# Supplementary material for: Modular access to chiral bridged piperidine-γ-butyrolactones via catalytic asymmetric allylation/aza-Prins cyclization/lactonization sequences
Source: Nat Commun. 2024 Jan 2;15:127. doi: 10.1038/s41467-023-44336-2 (PMC10762176; doi:10.1038/s41467-023-44336-2)
Supplement: Supplementary file 1 — Revised_Supplementary Information [file 41467_2023_44336_MOESM1_ESM.pdf]

Supplementary Information for

Modular Access to Chiral Bridged Piperidine-γ-butyrolactones *via* Catalytic Asymmetric Allylation/*aza*-Prins Cyclization/Lactonization Sequences

Cong Fu,[1,2,4] Ling He,[1,4] Hui Xu,[3,4] Zongpeng Zhang,[1] Xin Chang,[1] Yanfeng Dang,[3]* Xiu-Qin Dong,[1]* and Chun-Jiang Wang[1,2]*

[1]College of Chemistry and Molecular Sciences, Wuhan University, Wuhan 430072, China; [2]State Key Laboratory of Elemento-organic Chemistry, Nankai University, Tianjin, 300071, China; [3]Tianjin Key Laboratory of Molecular Optoelectronic Sciences, Department of Chemistry, Tianjin University, Tianjin 300072, China; [4]These two authors contributed equally

E-mail: yanfeng.dang@tju.edu.cn (Y.D.); xiuqindong@whu.edu.cn (X.Q.D.); cjwang@whu.edu.cn (C.J.W.)

Table of Contents

1. Supplementary Methods........S2

1.1 General remarks........S2

1.2 Additional optimization data........S3

1.3 General procedure for synergistic Cu/Ir-catalyzed synthesis of enantioenriched α-allyl α-amino acid derivatives and *aza*-Prins cyclization/lactonization........S4

1.4 Gram-scale synthesis and synthetic transformation........S18

2. Supplementary Discussion........S21

2.1 X-ray structure of (1*S*,3*S*,5*R*)-**4a** and (1*R*,3*R*,5*S*)-**4a**........S21

2.2 Biological activity study........S21

2.3 Computational method........S22

3. Supplementary Figures........S26

3.1 ^{1}H NMR, ^{13}C NMR, and F ^{19}NMR spectra........S26

3.2 HPLC spectra........S55

4. Supplementary Reference........S111

1. Supplementary Methods

1.1 General remarks

^{1}H NMR spectra were recorded on a Bruker 400 MHz spectrometer in $CDCl_3$. Chemical shifts are reported in ppm with the internal TMS signal at 0.0 ppm as a standard. The data are reported as (s = single, d = double, t = triple, q = quarte, m = multiple or unresolved, brs = broad single, coupling constant(s) in Hz, integration). ^{13}C NMR spectra were recorded on a Bruker 100 MHz spectrometer in $CDCl_3$. Chemical shifts are reported in ppm with the internal chloroform signal at 77.0 ppm as a standard. Commercially obtained reagents were used without further purification. Solvents were purified prior to use according to the standard methods. Unless otherwise noted, all reactions were carried out under argon atmosphere. Enantiomeric ratios were determined by chiral-phase HPLC analysis in comparison with authentic racemic materials using a chiralpak AD-H, AS-H, IA, IC or chiralcel OD-H column with hexane and *i*-PrOH as solvents. High resolution mass spectra were recorded using ESI-TOF technique. The aldimine esters **1**,[1,2] allylcarbonates,[3,4] **L1**[5] and **L5**[6] were prepared according to the literature procedure. All calculations were performed with Gaussian 09[7] at the M06-L[8,9] level of density functional theory. The M06-L functional and a mixed basis set of SDD[10,11] for Cu, Fe, and Ir and 6-31G(d) for other atoms were used for geometry optimizations and frequency calculations. Frequency outcomes were examined to confirm the stationary points as minima (no imaginary frequencies) or transition states (only one imaginary frequency). Single-point energies were then calculated with M06-L functional and the def2-TZVP[12] basis set for all atoms, in which the solvation effects were modeled by SMD.[13] Free energies (in kcal/mol) in solution (concentration: 1 mol/L) were utilized in the discussions. The optimized geometries were represented by using CYLView.[14]

1.2 Additional optimization of reaction condition

Supplementary Table 1. Optimization of Acids[a]

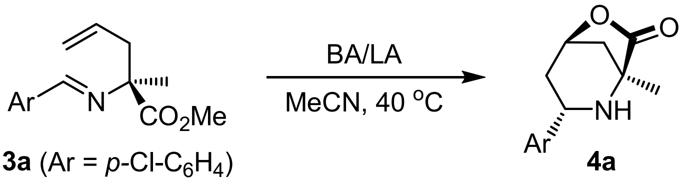

entry	**BA or LA** (3.5 eq.)	dr[b]	yield (%)[c]	ee (%)[d]
1	$Cu(OTf)_2$	-	no reaction	NA
2	$Zn(OTf)_2$	-	no reaction	NA
3	TMSOTf	>20:1	85	95
4	TBSOTf	>20:1	60	88
5	TfOH	>20:1	96	94
6	TFA	-	decomposed	NA
7	$MeSO_3H$	-	ND	NA
8	$TsOH·H_2O$	-	ND	NA
9[e]	TfOH	>20:1	85	94

[a] **3a** (0.15 mmol) was dissolved in MeCN (1.5 mL), follow by addition of the acid (3.5 eq.). The reaction mixture was stirred at 40 °C for 3 days, neutralized with excessive triethylamine and purified by column chromatography. [b] Dr was determined by the crude ^{1}H NMR analysis. [c] Yields refer to the isolated products after chromatographic purification. [d] Ee was determined by chiral HPLC analysis. [e] 2.0 eq. TfOH was used.

Supplementary Table 2. Optimization of the Solvent[a]

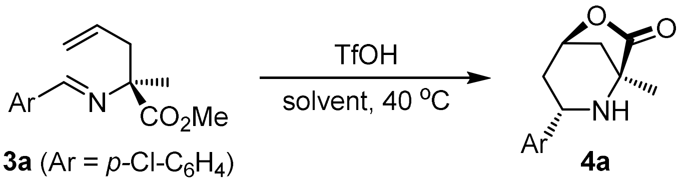

entry	**solvent**	dr[b]	yield (%)[c]	ee (%)[d]
1	MeCN	>20:1	96	94
2	toluene	>20:1	77	95
3	DMF	-	decomposed	NA
4	MeOH	-	decomposed	NA
5	THF	-	trace	NA
6	acetone	-	complex mixture	NA
7	DMSO	-	complex mixture	NA

[a] **3a** (0.15 mmol) was dissolved in the solvent (1.5 mL), follow by addition of TfOH (3.5 eq.). The reaction mixture was stirred at 40 °C for 3 days, neutralized with excessive triethylamine and purified by column chromatography. [b] Dr was determined by the crude ^{1}H NMR analysis. [c] Yields refer to the isolated products after chromatographic purification. [d] Ee was determined by chiral HPLC analysis.

Supplementary Table 3. Optimization of the Temperature[a]

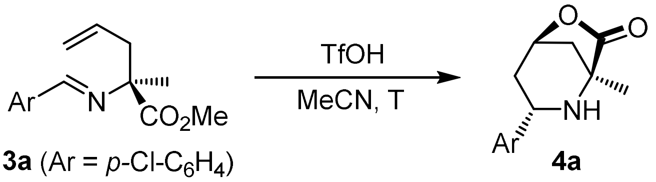

entry	**T** (°C)	dr[b]	yield (%)[c]	ee (%)[d]
1	30	-	low conversion	-
2	40	>20:1	96	94
3	50	>20:1	91	91
4	reflux	>20:1	69	20

[a] **3a** (0.15 mmol) was dissolved in the solvent (1.5 mL), follow by addition of TfOH (3.5 eq.). The reaction mixture was stirred at a certain temperature for 3 days, neutralized with excessive triethylamine and purified by column chromatography. [b] Dr was determined by the crude ^{1}H NMR analysis. [c] Yields refer to the isolated products after chromatographic purification. [d] Ee was determined by chiral HPLC analysis.

1.3 General procedure for synergistic Cu/Ir-catalyzed synthesis of enantioenriched α-allyl α-amino acid derivatives and *aza*-Prins cyclization/lactonization

A flame dried Schlenk tube was cooled to room temperature and filled with Ar. To this flask were added $[Ir(COD)Cl]_2$ (0.003 mmol, 1.5 mol %), phosphoramidite ligand (*S,S,S*)-**L5** (0.006 mmol, 3 mol %), degassed THF (0.5 mL) and degassed *n*-propylamine (0.5 mL). The reaction mixture was stirred at 50 °C for 30 min and then the volatile solvents were removed in vacuum to give a pale yellow solid. Meanwhile, in a separated Schlenk tube, (*S,S$_p$*)-*i*Pr-Phosferrox-**L1** (0.011 mmol, 5.5 mol %) and $Cu(MeCN)_4BF_4$ (0.01 mmol, 5 mol %) were dissolved in DCM (0.5 mL), and stirred at room temperature for about 0.5 h. A solution of aldimine ester **1** (0.30 mmol) in CH_2Cl_2 (0.5 mL) was added, followed by Cs_2CO_3 (98 mg, 0.30 mmol), allylic carbonate **4** (0.20 mmol) in DCM (0.5 mL) and pre-prepared Ir-complex in DCM (0.5 mL). The reaction mixture was stirred at 25 °C for 10-14 h. The organic solvent was removed by rotary evaporation. The residue can be used directly in ensuing acid-promoted *aza*-Prins cylization/lactonization or be purified by column chromatography (1-5% of EtOAc and 1% of Et_3N in PE) to afford the allylation product. To a solution of the obtained crude allylation product in MeCN (1.5 mL) was added TfOH (3.5 eq.), the reaction mixture was stirred at 40 °C for 2-3 days (Note: 50 °C was needed for some electron-enriched substrates, including the cases of **4i**, **4l**, **4m**, **4p** and **4r**), basified with Et_3N (0.1 mL) and then concentrated in vacuum. The residue was purified by column chromatography on silica gel (PE: EtOAc = 6: 1)

afforded the bridged-heterocyclic product **4**. The dr value was determined by ^{1}H NMR spectrum of the product, and the enantiomeric excess was recorded by HPLC analysis in comparison with the racemic sample.

methyl (*S*)-2-((4-chlorobenzylidene)amino)-2-methylpent-4-enoate

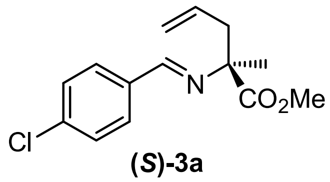

(*S*)-3a

Yield (94%); colorless oil; $[\alpha]^{20}_{D}$ = -17.5 (*c* 1.0, acetone); ^{1}H NMR (400 MHz, $CDCl_3$) δ 8.20 (s, 1H), 7.75 – 7.64 (m, 2H), 7.43 – 7.34 (m, 2H), 5.85 – 5.70 (m, 1H), 5.16 – 5.04 (m, 2H), 3.74 (s, 3H), 2.67 (d, *J* = 7.3 Hz, 2H), 1.49 (s, 3H). ^{13}C NMR (100 MHz, $CDCl_3$) δ 174.0, 158.1, 136.8, 134.7, 133.1, 129.5, 128.8, 118.9, 68.5, 52.2, 44.1, 22.7. HRMS (ESI-TOF) Calcd. For $C_{14}H_{17}ClNO_2^+$ ($[M+H]^+$): 266.0942, found: 266.0939. The product was analyzed by HPLC to determine the enantiomeric excess: >99% ee (Chiralpak AS-H, *i*-propanol/hexane = 5/95, flow rate 1.0 mL/min, λ = 254 nm); t_r = 4.9 and 5.6 min.

(1*S*,3*S*,5*R*)-3-(4-chlorophenyl)-1-methyl-6-oxa-2-azabicyclo[3.2.1]octan-7-one

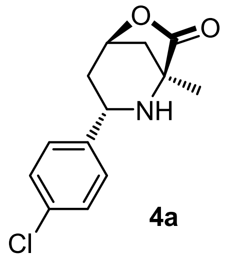

4a

Yield (90%); white solid; $[\alpha]^{20}_{D}$ = -12.4 (*c* 0.5, acetone); mp 118–120 °C; ^{1}H NMR (400 MHz, $CDCl_3$) δ 7.38 – 7.28 (m, 4H), 4.84 (t, *J* = 5.3 Hz, 1H), 4.11 (dd, *J* = 11.2, 4.8 Hz, 1H), 2.25 – 2.14 (m, 2H), 2.02 (d, *J* = 11.2 Hz, 1H), 1.82 (brs, 1H), 1.63 – 1.58 (m, 1H), 1.38 (s, 3H). ^{13}C NMR (100 MHz, $CDCl_3$) δ 178.0, 141.2, 133.3, 128.7, 128.3, 75.0, 58.3, 54.7, 42.8, 37.4, 20.0. HRMS (ESI-TOF) Calcd. For $C_{13}H_{15}ClNO_2^+$ ($[M+H]^+$): 252.0786, found: 252.0785. >20:1 dr. The product was analyzed by HPLC to determine the enantiomeric excess: 94% ee (Chiralpak AD-H, *i*-propanol/hexane = 15/85, flow rate 1.0 mL/min, λ = 210 nm); t_r = 6.9 and 8.2 min.

(1*S*,3*S*,5*R*)-1-methyl-3-(4-(trifluoromethyl)phenyl)-6-oxa-2-azabicyclo[3.2.1]octan-7-one

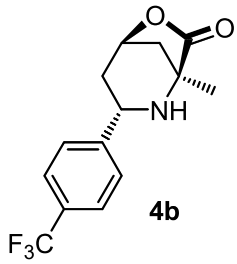

4b

Yield (73%); white solid; $[\alpha]^{20}_{D}$ = -7.2 (*c* 0.5, acetone); mp 100-102 °C; ^{1}H NMR (400 MHz, $CDCl_3$) δ 7.67 – 7.45 (m, 4H), 4.86 (t, *J* = 5.3 Hz, 1H), 4.20 (dd, *J* = 11.1, 4.8 Hz, 1H), 2.27 – 2.18 (m, 2H), 2.04 (d, *J* = 11.4 Hz, 1H), 1.83 (brs, 1H), 1.64 – 1.58 (m, 1H), 1.40 (s, 3H). ^{13}C NMR (100 MHz, $CDCl_3$) δ 177.9, 146.7, 130.0 (q, *J* = 32.4 Hz), 127.3, 125.5 (q, *J* = 3.8 Hz), 124.0 (q, *J* = 271.0 Hz), 74.9, 58.3, 55.0, 42.8, 37.4, 20.0. ^{19}F NMR (376 MHz, $CDCl_3$) δ -62.5. HRMS (ESI-TOF) Calcd. For $C_{14}H_{15}F_3NO_2^+$ ($[M+H]^+$): 286.1049, found: 286.1048. >20:1 dr. The product was analyzed by HPLC to determine the enantiomeric excess: 95% ee (Chiralpak AD-H, *i*-propanol/hexane = 15/85, flow rate 1.0 mL/min, λ = 210 nm); t_r = 6.5 and 7.8 min.

4-((1*S*,3*S*,5*R*)-1-methyl-7-oxo-6-oxa-2-azabicyclo[3.2.1]octan-3-yl)benzonitrile

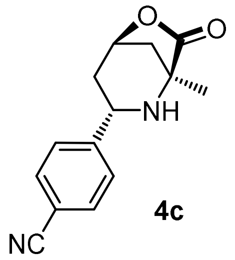

4c

Yield (93%); white solid; $[\alpha]^{20}_{D}$ = -15.2 (*c* 0.5, acetone); mp 180–182 °C; ^{1}H NMR (400 MHz, $CDCl_3$) δ 7.74 – 7.42 (m, 4H), 4.86 (t, *J* = 5.3 Hz, 1H), 4.20 (dd, *J* = 11.1, 4.8 Hz, 1H), 2.26 – 2.19 (m, 2H), 2.03 (d, *J* = 11.6 Hz, 1H), 1.87 (s, 1H), 1.61 – 1.54 (m, 1H), 1.40 (s, 3H). ^{13}C NMR (100 MHz, $CDCl_3$) δ 177.7, 148.1, 132.4, 127.6, 118.6, 111.5, 74.8, 58.2, 55.0, 42.7, 37.3, 20.0. HRMS (ESI-TOF) Calcd. For $C_{14}H_{15}N_2O_2^+$ ($[M+H]^+$): 243.1128, found: 243.1128. >20:1 dr. The product was analyzed by HPLC to determine the enantiomeric excess: 95% ee (Chiralpak AD-H, *i*-propanol/hexane = 25/75, flow rate 1.0 mL/min, λ = 210 nm); t_r = 9.1 and 10.0 min.

methyl 4-((1*S*,3*S*,5*R*)-1-methyl-7-oxo-6-oxa-2-azabicyclo[3.2.1]octan-3-yl)benzoate

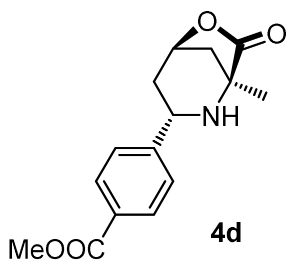

4d

Yield (96%); white solid; $[\alpha]^{20}_{D}$ = -13.8 (*c* 0.5, acetone); mp 160-161 °C; ^{1}H NMR (400 MHz, $CDCl_3$) δ 8.00 (d, *J* = 8.3 Hz, 2H), 7.48 (d, *J* = 8.2 Hz, 2H), 4.85 (t, *J* = 5.3 Hz, 1H), 4.20 (dd, *J* = 11.1, 4.8 Hz, 1H), 3.91 (s, 3H), 2.30 – 2.15 (m, 2H), 2.04 (d, *J* = 11.4 Hz, 1H), 1.87 (brs, 1H), 1.64 – 1.58 (m, 1H), 1.40 (s, 3H). ^{13}C NMR (100 MHz, $CDCl_3$) δ 177.9, 166.8, 147.8, 129.9, 129.6, 126.9, 75.0, 58.3, 55.1, 52.1, 42.8, 37.3, 20.0. HRMS (ESI-TOF) Calcd. For $C_{15}H_{18}NO_4^+$ ($[M+H]^+$): 276.1230, found: 276.1229. >20:1 dr. The product was analyzed by HPLC to determine the enantiomeric excess: 96% ee (Chiralcel OD-H, *i*-propanol/hexane = 10/90, flow rate 1.0 mL/min, λ = 210 nm); t_r = 19.8 and 23.7 min.

(1*S*,3*S*,5*R*)-1-methyl-3-(3-nitrophenyl)-6-oxa-2-azabicyclo[3.2.1]octan-7-one

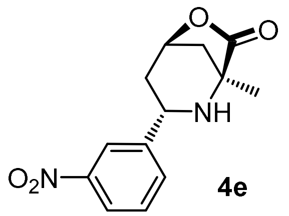

4e

Yield (87%); white solid; $[\alpha]^{20}_{D}$ = -16.4 (*c* 0.5, acetone); mp 116–118 °C; ^{1}H NMR (400 MHz, $CDCl_3$) δ 8.37 – 8.36 (m, 1H), 8.15 – 8.13 (m, 1H), 7.70 (d, *J* = 7.7 Hz, 1H), 7.53 – 7.49 (m, 1H), 4.87 (t, *J* = 5.3 Hz, 1H), 4.26 (dd, *J* = 11.1, 4.9 Hz, 1H), 2.33 – 2.18 (m, 2H), 2.07 (d, *J* = 11.5 Hz, 1H), 1.91 (brs, 1H), 1.65 – 1.59 (m, 1H), 1.42 (s, 3H). ^{13}C NMR (100 MHz, $CDCl_3$) δ 177.7, 148.5, 145.0, 133.2, 129.5, 122.8, 121.9, 74.8, 58.3, 54.7, 42.6, 37.4, 20.0. HRMS (ESI-TOF) Calcd. For $C_{13}H_{15}N_2O_4^+$ ($[M+H]^+$): 263.1026, found: 263.1025. >20:1 dr. The product was analyzed by HPLC to determine the enantiomeric excess: 96% ee (Chiralpak AD-H, *i*-propanol/hexane = 25/75, flow rate 1.0 mL/min, λ = 210 nm); t_r = 8.5 and 10.1 min.

(1*S*,3*S*,5*R*)-1-methyl-3-(3-(methylsulfonyl)phenyl)-6-oxa-2-azabicyclo[3.2.1]octan-7-one

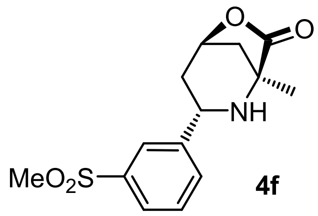

4f

Yield (85%); white solid; $[\alpha]^{20}_{D}$ = -14.4 (*c* 0.25, acetone); mp 198–200 °C; ^{1}H NMR (400 MHz, $CDCl_3$) δ 7.87 (d, *J* = 8.3 Hz, 2H), 7.61 (d, *J* = 8.3 Hz, 2H), 4.84 (t, *J* = 5.3 Hz, 1H), 4.21 (dd, *J* = 11.1, 4.9 Hz, 1H), 3.02 (s, 3H), 2.29 – 2.12 (m, 2H), 2.02 (d, *J* = 11.5 Hz, 1H), 1.88 (brs, 1H), 1.60 – 1.54 (m, 1H), 1.37 (s, 3H). ^{13}C NMR (100 MHz, $CDCl_3$) δ 177.7, 149.1, 139.7, 127.8, 127.6, 74.8, 58.2, 54.8, 44.4, 42.6, 37.3, 19.9. HRMS (ESI-TOF) Calcd. For $C_{14}H_{18}NO_4S^+$ ($[M+H]^+$): 296.0951, found: 296.0951. >20:1 dr. The product was analyzed by HPLC to determine the enantiomeric excess: 96% ee (Chiralpak IA, *i*-propanol/hexane = 40/60, flow rate 1.0 mL/min, λ = 210 nm); t_r = 11.2 and 13.3 min.

(1*S*,3*S*,5*R*)-3-(2-chlorophenyl)-1-methyl-6-oxa-2-azabicyclo[3.2.1]octan-7-one

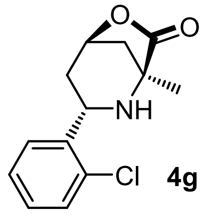

4g

Yield (67%); white solid; $[\alpha]^{20}_{D}$ = -18.6 (*c* 0.5, acetone); mp 100–101 °C; ^{1}H NMR (400 MHz, $CDCl_3$) δ 7.77 (dd, *J* = 7.8, 1.6 Hz, 1H), 7.34 – 7.25 (m, 2H), 7.21 – 7.17 (m, 1H), 4.86 (t, *J* = 5.3 Hz, 1H), 4.63 (dd, *J* = 11.0, 4.9 Hz, 1H), 2.48 – 2.41 (m, 1H), 2.21 – 2.17 (m, 1H), 2.02 (d, *J* = 11.5 Hz, 1H), 1.81 (s, 1H), 1.48 (dd, *J* = 13.5, 11.1 Hz, 1H), 1.39 (s, 3H). ^{13}C NMR (100 MHz, $CDCl_3$) δ 178.0, 139.9, 132.6, 129.5, 128.5, 128.3, 127.2, 75.0, 58.2, 51.2, 42.8, 34.7, 20.2. HRMS (ESI-TOF) Calcd. For $C_{13}H_{15}ClNO_2^+$ ($[M+H]^+$): 252.0786, found: 252.0785. >20:1 dr. The product was analyzed by HPLC to determine the enantiomeric excess: 96% ee (Chiralpak AD-H, *i*-propanol/hexane = 15/85, flow rate 1.0 mL/min, λ = 210 nm); t_r = 5.6 and 7.1 min.

(1*S*,3*S*,5*R*)-1-methyl-3-phenyl-6-oxa-2-azabicyclo[3.2.1]octan-7-one

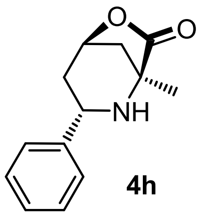

4h

Yield (86%); white solid; $[\alpha]^{20}_{D}$ = -1.2 (*c* 0.5, acetone); mp 108–110 °C; ^{1}H NMR (400 MHz, $CDCl_3$) δ 7.44 – 7.37 (m, 2H), 7.35 – 7.31 (m, 2H), 7.30 – 7.25 (m, 1H), 4.84 (t, *J* = 5.3 Hz, 1H), 4.13 (dd, *J* = 11.1, 4.8 Hz, 1H), 2.25 – 2.16 (m, 2H), 2.04 (d, *J* = 11.4 Hz, 1H), 1.84 (brs, 1H), 1.66 (dd, *J* = 13.4, 11.3 Hz, 1H), 1.38 (s, 3H). ^{13}C NMR (100 MHz, $CDCl_3$) δ 178.2, 142.6, 128.5, 127.8, 127.0, 75.2, 58.4, 55.4, 42.9, 37.3, 20.1. HRMS (ESI-TOF) Calcd. For $C_{13}H_{16}NO_2^+$ ($[M+H]^+$): 218.1776, found: 218.1774. >20:1 dr. The product was analyzed by HPLC to determine the enantiomeric excess: 95% ee (Chiralpak AD-H, *i*-propanol/hexane = 10/90, flow rate 1.0 mL/min, λ = 210 nm); t_r = 7.5 and 8.2 min.

(1*S*,3*S*,5*R*)-1-methyl-3-(p-tolyl)-6-oxa-2-azabicyclo[3.2.1]octan-7-one

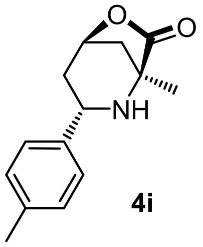

4i

Yield (49%); white solid; $[\alpha]^{20}_{D}$ = -3.6 (*c* 0.5, acetone); mp 115–117 °C; ^{1}H NMR (400 MHz, $CDCl_3$) δ 7.29 (d, *J* = 8.0 Hz, 2H), 7.14 (d, *J* = 8.0 Hz, 2H), 4.84 (t, *J* = 5.3 Hz, 1H), 4.10 (dd, *J* = 11.2, 4.7 Hz, 1H), 2.33 (s, 3H), 2.23 – 2.14 (m, 2H), 2.04 (d, *J* = 11.5 Hz, 1H), 1.85 (brs, 1H), 1.66 (dd, *J* = 13.5, 11.4 Hz, 1H), 1.37 (s, 3H). ^{13}C NMR (100 MHz, $CDCl_3$) δ 178.3, 139.6, 137.5, 129.2, 126.9, 75.3, 58.5, 55.1, 42.9, 37.3, 21.1, 20.1. HRMS (ESI-TOF) Calcd. For $C_{14}H_{18}NO_2^+$ ($[M+H]^+$): 232.1332, found: 232.1331. >20:1 dr. The product was analyzed by HPLC to determine the enantiomeric excess: 97% ee (Chiralpak AD-H, *i*-propanol/hexane = 15/85, flow rate 1.0 mL/min, λ = 210 nm); t_r = 5.7 and 6.9 min.

(1*S*,3*S*,5*R*)-1-methyl-3-(m-tolyl)-6-oxa-2-azabicyclo[3.2.1]octan-7-one

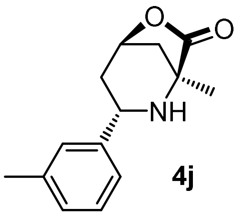

4j

Yield (76%); white solid; $[\alpha]^{20}_{D}$ = -17.2 (*c* 0.5, acetone); mp 118–120 °C; ^{1}H NMR (400 MHz, $CDCl_3$) δ 7.24 – 7.17 (m, 3H), 7.09 (d, *J* = 6.8 Hz, 1H), 4.84 (t, *J* = 5.3 Hz, 1H), 4.09 (dd, *J* = 11.2, 4.7 Hz, 1H), 2.35 (s, 3H), 2.25 – 2.14 (m, 2H), 2.04 (d, *J* = 11.5 Hz, 1H), 1.82 (brs, 1H), 1.66 (dd, *J* = 13.5, 11.4 Hz, 1H), 1.38 (s, 3H). ^{13}C NMR (100 MHz, $CDCl_3$) δ 178.2, 142.5, 138.2, 128.5, 128.4, 127.7, 124.1, 75.2, 58.4, 55.3, 42.9, 37.2, 21.4, 20.1. HRMS (ESI-TOF) Calcd. For $C_{14}H_{18}NO_2^+$ ($[M+H]^+$): 232.1332, found: 232.1331. >20:1 dr. The product was analyzed by HPLC to determine the enantiomeric excess: 95% ee (Chiralpak AD-H, *i*-propanol/hexane = 15/85, flow rate 1.0 mL/min, λ = 210 nm); t_r = 5.2 and 5.9 min.

(1*S*,3*S*,5*R*)-1-methyl-3-(o-tolyl)-6-oxa-2-azabicyclo[3.2.1]octan-7-one

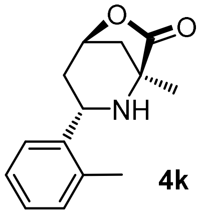

4k

Yield (70%); white solid; $[\alpha]^{20}_{D}$ = -12.4 (*c* 0.5, acetone); mp 123–125 °C; ^{1}H NMR (400 MHz, $CDCl_3$) δ 7.68 (d, *J* = 7.5 Hz, 1H), 7.23 – 7.19 (m, 1H), 7.18 – 7.13 (m, 1H), 7.12 – 7.10 (m, 1H), 4.85 (t, *J* = 5.3 Hz, 1H), 4.39 (dd, *J* = 11.1, 4.8 Hz, 1H), 2.33 (s, 3H), 2.27 – 2.15 (m, 2H), 2.05 (d, *J* = 11.3 Hz, 1H), 1.72 (brs, 1H), 1.56 (dd, *J* = 13.5, 11.2 Hz, 1H), 1.38 (s, 3H). ^{13}C NMR (100 MHz, $CDCl_3$) δ 178.3, 140.5, 135.0, 130.4, 127.2, 126.9, 126.4, 75.2, 58.4, 51.1, 42.9, 35.6, 20.1, 19.0. HRMS (ESI-TOF) Calcd. For $C_{14}H_{18}NO_2^+$ ($[M+H]^+$): 232.1332, found: 232.1332. >20:1 dr. The product was analyzed by HPLC to determine the enantiomeric excess: 88% ee (Chiralpak AD-H, *i*-propanol/hexane = 15/85, flow rate 1.0 mL/min, λ = 210 nm); t_r = 4.8 and 5.7 min.

(1*S*,3*S*,5*R*)-3-(4-isobutylphenyl)-1-methyl-6-oxa-2-azabicyclo[3.2.1]octan-7-one

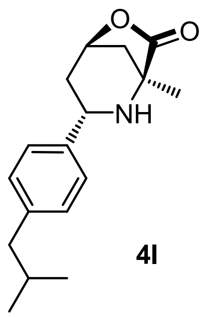

4l

Yield (41%); colorless gum; $[\alpha]^{20}_{D}$ = -12.0 (*c* 0.2, acetone); ^{1}H NMR (400 MHz, $CDCl_3$) δ 7.29 (d, *J* = 8.0 Hz, 2H), 7.10 (d, *J* = 8.0 Hz, 2H), 4.84 (t, *J* = 5.3 Hz, 1H), 4.10 (dd, *J* = 11.2, 4.7 Hz, 1H), 2.45 (d, *J* = 7.2 Hz, 2H), 2.25 – 2.14 (m, 2H), 2.03 (d, *J* = 11.4 Hz, 1H), 1.89 – 1.79 (m, 2H), 1.67 (dd, *J* = 13.6, 11.4 Hz, 1H), 1.37 (s, 3H), 0.89 (d, *J* = 6.6 Hz, 6H). ^{13}C NMR (100 MHz, $CDCl_3$) δ 178.3, 141.3, 139.8, 129.3, 126.8, 75.3, 58.5, 55.1, 45.0, 43.0, 37.3, 30.2, 22.4, 22.3, 20.1. HRMS (ESI-TOF) Calcd. For $C_{17}H_{24}NO_2^+$ ($[M+H]^+$): 274.1802, found: 274.1798. >20:1 dr. The product was analyzed by HPLC to determine the enantiomeric excess: 97% ee (Chiralpak AD-H, *i*-propanol/hexane = 15/85, flow rate 1.0 mL/min, λ = 210 nm); t_r = 5.0 and 6.3 min.

(1*S*,3*S*,5*R*)-3-(3-methoxyphenyl)-1-methyl-6-oxa-2-azabicyclo[3.2.1]octan-7-one

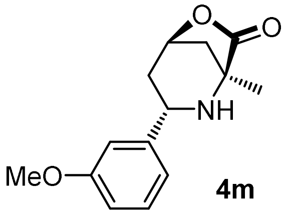

4m

Yield (71%); white solid; $[\alpha]^{20}_{D}$ = +3.4 (*c* 0.5, acetone); mp 90–91 °C; ^{1}H NMR (400 MHz, $CDCl_3$) δ 7.26 – 7.22 (m, 1H), 7.01 – 6.93 (m, 2H), 6.84 – 6.77 (m, 1H), 4.84 (t, *J* = 5.3 Hz, 1H), 4.10 (dd, *J* = 11.1, 4.8 Hz, 1H), 3.80 (s, 3H), 2.28 – 2.12 (m, 2H), 2.02 (d, *J* = 11.3 Hz, 1H), 1.84 (brs, 1H), 1.64 (dd, *J* = 13.4, 11.3 Hz, 1H), 1.37 (s, 3H). ^{13}C NMR (100 MHz, $CDCl_3$) δ 178.2, 159.7, 144.3, 129.5, 119.2, 113.0, 112.6, 75.2, 58.3, 55.2, 55.2, 42.8, 37.2, 20.0. HRMS (ESI-TOF) Calcd. For $C_{14}H_{18}NO_3^+$ ($[M+H]^+$): 248.1281, found: 248.1280. >20:1 dr. The product was analyzed by HPLC to determine the enantiomeric excess: 95% ee (Chiralpak AD-H, *i*-propanol/hexane = 15/85, flow rate 1.0 mL/min, λ = 210 nm); t_r = 7.1 and 8.6 min.

(1*S*,3*S*,5*R*)-1-methyl-3-(naphthalen-1-yl)-6-oxa-2-azabicyclo[3.2.1]octan-7-one

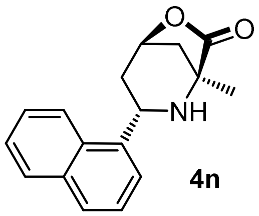

4n

Yield (63%); white solid; $[\alpha]^{20}_{D}$ = -2.6 (*c* 0.5, acetone); mp 200–202 °C; ^{1}H NMR (400 MHz, $CDCl_3$) δ 8.16 (d, *J* = 8.0 Hz, 1H), 7.88 – 7.85 (m, 2H), 7.77 (d, *J* = 8.2 Hz, 1H), 7.58 – 7.41 (m, 3H), 4.97 (dd, *J* = 11.0, 4.3 Hz, 1H), 4.90 (t, *J* = 5.3 Hz, 1H), 2.53 – 2.43 (m, 1H), 2.26 – 2.22 (m, 1H), 2.12 (d, *J* = 11.5 Hz, 1H), 1.89 (brs, 1H), 1.75 (dd, *J* = 13.4, 11.6 Hz, 1H), 1.44 (s, 3H). ^{13}C NMR (100 MHz, $CDCl_3$) δ 178.3, 138.2, 133.8, 130.6, 129.0, 128.0, 126.2, 125.61, 125.59, 124.1, 122.6, 75.4, 58.6, 51.2, 42.9, 36.2, 20.2. HRMS (ESI-TOF) Calcd. For $C_{17}H_{18}NO_2^+$ ($[M+H]^+$): 268.1332, found: 268.1333. >20:1 dr. The product was analyzed by HPLC to determine the enantiomeric excess: 87% ee (Chiralpak AD-H, *i*-propanol/hexane = 15/85, flow rate 1.0 mL/min, λ = 210 nm); t_r = 6.2 and 6.9 min.

(1*S*,3*S*,5*R*)-1-methyl-3-(naphthalen-2-yl)-6-oxa-2-azabicyclo[3.2.1]octan-7-one

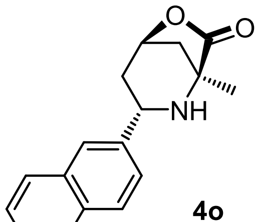

4o

Yield (85%); white solid; $[\alpha]^{20}_{D}$ = -2.8 (*c* 0.5, acetone); mp 130–132 °C; ^{1}H NMR (400 MHz, $CDCl_3$) δ 7.89 – 7.75 (m, 4H), 7.56 – 7.51 (m, 1H), 7.50 – 7.42 (m, 2H), 4.87 (t, *J* = 5.3 Hz, 1H), 4.30 (dd, *J* = 11.1, 4.8 Hz, 1H), 2.33 – 2.17 (m, 2H), 2.09 (d, *J* = 11.5 Hz, 1H), 1.95 (brs, 1H), 1.74 (dd, *J* = 13.4, 11.4 Hz, 1H), 1.41 (s, 3H). ^{13}C NMR (100 MHz, $CDCl_3$) δ 178.2, 139.9, 133.3, 133.1, 128.3, 127.8, 127.6, 126.2, 125.9, 125.7, 125.0, 75.2, 58.5, 55.5, 42.9, 37.3, 20.1. HRMS (ESI-TOF) Calcd. For $C_{17}H_{18}NO_2^+$ ($[M+H]^+$): 268.1332, found: 268.1329. >20:1 dr. The product was analyzed by HPLC to determine the enantiomeric excess: 96% ee (Chiralpak AD-H, *i*-propanol/hexane = 15/85, flow rate 1.0 mL/min, λ = 210 nm); t_r = 7.7 and 9.1 min.

(1*S*,3*S*,5*R*)-1-methyl-3-(thiophen-2-yl)-6-oxa-2-azabicyclo[3.2.1]octan-7-one

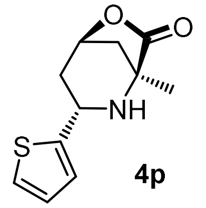

4p

Yield (49%); yellow solid; $[\alpha]^{20}_{D}$ = +9.2 (*c* 0.5, acetone); mp 114–116 °C; ^{1}H NMR (400 MHz, $CDCl_3$) δ 7.23 (dd, *J* = 5.0, 1.2 Hz, 1H), 6.99 – 6.91 (m, 2H), 4.84 (t, *J* = 5.3 Hz, 1H), 4.47 (dd, *J* = 11.0, 4.9 Hz, 1H), 2.34 – 2.28 (m, 1H), 2.19 – 2.14 (m, 1H), 2.06 (d, *J* = 11.5 Hz, 1H), 1.78 (dd, *J* = 13.5, 11.1 Hz, 1H), 1.38 (s, 3H). ^{13}C NMR (100 MHz, $CDCl_3$) δ 177.3, 146.1, 126.3, 124.5, 123.9, 74.6, 58.3, 50.8, 42.3, 37.7, 19.5. HRMS (ESI-TOF) Calcd. For $C_{11}H_{14}NO_2S^+$ ($[M+H]^+$): 224.0740, found: 224.0739. >20:1 dr. The product was analyzed by HPLC to determine the enantiomeric excess: 96% ee (Chiralpak IC, *i*-propanol/hexane = 10/90, flow rate 1.0 mL/min, λ = 210 nm); t_r = 15.5 and 18.0 min.

(1*S*,3*S*,5*R*)-3-(2-bromopyridin-3-yl)-1-methyl-6-oxa-2-azabicyclo[3.2.1]octan-7-one

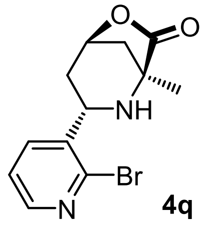

4q

Yield (65%); light yellow solid; $[\alpha]^{20}_{D}$ = -32.0 (*c* 0.5, acetone); mp 122–124 °C; ^{1}H NMR (400 MHz, $CDCl_3$) δ 8.29-8.27 (m, 1H), 8.11-8.09 (m, 1H), 7.31-7.27 (m, 1H), 4.88 (t, *J* = 5.3 Hz, 1H), 4.52 (dd, *J* = 10.9, 4.9 Hz, 1H), 2.62 – 2.50 (m, 1H), 2.24 – 2.19 (m, 1H), 2.01 (d, *J* = 11.5 Hz, 1H), 1.86 (brs, 1H), 1.41 (s, 3H). ^{13}C NMR (100 MHz, $CDCl_3$) δ 177.6, 149.1, 142.4, 139.2, 137.3, 123.3, 74.6, 58.2, 53.2, 42.7, 34.6, 20.1. HRMS (ESI-TOF) Calcd. For $C_{12}H_{14}BrN_2O_2^+$ ($[M+H]^+$): 297.0233, found: 297.0230. >20:1 dr. The product was analyzed by HPLC to determine the enantiomeric excess: 89% ee (Chiralpak AD-H, *i*-propanol/hexane = 25/75, flow rate 1.0 mL/min, λ = 210 nm); t_r = 6.9 and 9.7 min.

(1*S*,3*S*,5*R*)-1-methyl-3-(1-tosyl-1H-indol-3-yl)-6-oxa-2-azabicyclo[3.2.1]octan-7-one

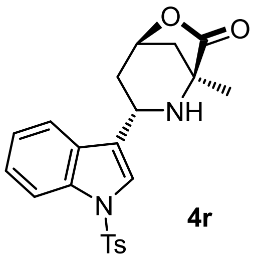

4r

Yield (45%); white solid; $[\alpha]^{20}_{D}$ = +1.2 (*c* 0.25, acetone); mp 181–183 °C; ^{1}H NMR (400 MHz, $CDCl_3$) δ 7.97 (d, *J* = 8.3 Hz, 1H), 7.76 (d, *J* = 8.4 Hz, 2H), 7.63 (d, *J* = 7.8 Hz, 1H), 7.57 (s, 1H), 7.35 – 7.28 (m, 1H), 7.25 – 7.15 (m, 3H), 4.86 (t, *J* = 5.3 Hz, 1H), 4.41 (dd, *J* = 11.1, 4.8 Hz, 1H), 2.36 – 2.30 (m, 1H), 2.34 (s, 3H), 2.23 – 2.19 (m, 1H), 2.07 (d, *J* = 11.5 Hz, 1H), 1.88 (brs, 1H), 1.78 (dd, *J* = 13.5, 11.3 Hz, 1H), 1.39 (s, 3H). ^{13}C NMR (100 MHz, $CDCl_3$) δ 178.1, 145.0, 135.5, 135.2, 129.9, 128.8, 126.8, 124.9, 124.1, 123.2, 123.1, 120.1, 113.9, 75.1, 58.5, 48.0, 42.8, 35.6, 21.6, 20.1. HRMS (ESI-TOF) Calcd. For $C_{22}H_{23}N_2O_4S^+$ ($[M+H]^+$): 411.1373, found: 411.1374. >20:1 dr. The product was analyzed by HPLC to determine the enantiomeric excess: 94% ee (Chiralpak AD-H, *i*-propanol/hexane = 75/25, flow rate 1.0 mL/min, λ = 210 nm); t_r = 11.4 and 15.2 min.

(1*S*,3*S*,5*R*)-3-cyclohexyl-1-methyl-6-oxa-2-azabicyclo[3.2.1]octan-7-one

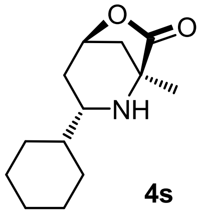

4s

Yield (40%); light yellow oil; $[\alpha]^{30}_{D}$ = +19.4 (*c* 1.0, acetone); ^{1}H NMR (400 MHz, $CDCl_3$) δ 4.78 (t, *J* = 5.3 Hz, 1H), 2.81 – 2.67 (m, 1H), 2.16 – 2.03 (m, 2H), 1.82 (d, *J* = 11.3 Hz, 1H), 1.79 – 1.62 (m, 6H), 1.38 – 1.32 (m, 1H), 1.32 (s, 3H), 1.29 – 1.06 (m, 4H), 0.97 – 0.85 (m, 2H). ^{13}C NMR (100 MHz, $CDCl_3$) δ 178.5, 75.2, 57.8, 55.0, 43.3, 42.8, 32.1, 29.0, 28.7, 26.3, 26.0, 25.9, 20.2. HRMS (ESI-TOF) Calcd. For $C_{13}H_{22}NO_2^+$ ($[M+H]^+$): 224.1645, found: 224.1647. >20:1 dr. The product was analyzed by GC to determine the enantiomeric excess: 87% ee (Gamma DEX 225, 30m x 0.25mm x 0.25um, column temperature: 180 °C, carrier gas: N_2, 1.0 mL/min, tr = 50.3 and 51.2 min).

(1*S*,3*S*,5*R*)-3-(4-chlorophenyl)-1-propyl-6-oxa-2-azabicyclo[3.2.1]octan-7-one

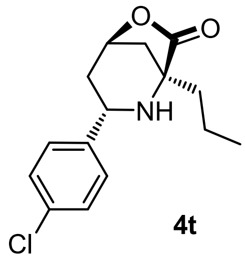

4t

Yield (69%); colorless gum; $[\alpha]^{20}_{D}$ = -15.6 (*c* 0.5, acetone); ^{1}H NMR (400 MHz, $CDCl_3$) δ 7.38 – 7.27 (m, 4H), 4.84 (t, *J* = 5.3 Hz, 1H), 4.11 (dd, *J* = 11.1, 4.8 Hz, 1H), 2.29 – 2.13 (m, 2H), 1.94 (d, *J* = 11.4 Hz, 1H), 1.77 – 1.49 (m, 5H), 1.44 – 1.29 (m, 1H), 0.98 (t, *J* = 7.1 Hz, 3H). ^{13}C NMR (100 MHz, $CDCl_3$) δ 177.7, 141.4, 133.3, 128.7, 128.3, 75.0, 61.6, 54.7, 40.1, 37.9, 35.8, 17.1, 14.4. HRMS (ESI-TOF) Calcd. For $C_{15}H_{19}ClNO_2^+$ ($[M+H]^+$): 280.1099, found: 280.1096. >20:1 dr. The product was analyzed by HPLC to determine the enantiomeric excess: 91% ee (Chiralpak AD-H, *i*-propanol/hexane = 15/85, flow rate 1.0 mL/min, λ = 210 nm); t_r = 7.2 and 10.9 min.

(1*S*,3*S*,5*R*)-3-(4-chlorophenyl)-1-isobutyl-6-oxa-2-azabicyclo[3.2.1]octan-7-one

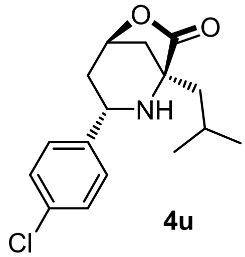

4u

Yield (80%); colorless gum; $[\alpha]^{20}_{D}$ = -15.6 (*c* 0.5, acetone); ^{1}H NMR (400 MHz, $CDCl_3$) δ 7.37 – 7.27 (m, 4H), 4.84 (t, *J* = 5.4 Hz, 1H), 4.09 (dd, *J* = 11.1, 4.9 Hz, 1H), 2.28 – 2.14 (m, 2H), 2.04 (d, *J* = 11.4 Hz, 1H), 1.91 – 1.81 (m, 1H), 1.73 – 1.62 (m, 2H), 1.61 – 1.52 (m, 2H), 1.02 (d, *J* = 6.6 Hz, 3H), 0.97 (d, *J* = 6.6 Hz, 3H). ^{13}C NMR (100 MHz, $CDCl_3$) δ 177.9, 141.5, 133.3, 128.7, 128.3, 75.0, 61.4, 54.7, 42.1, 40.3, 38.0, 24.5, 24.3, 23.6. HRMS (ESI-TOF) Calcd. For $C_{16}H_{21}ClNO_2^+$ ($[M+H]^+$): 294.1255, found: 294.1251. >20:1 dr. The product was analyzed by HPLC to determine the enantiomeric excess: 90% ee (Chiralpak AD-H, *i*-propanol/hexane = 15/85, flow rate 1.0 mL/min, λ = 210 nm); t_r = 7.8 and 8.5 min.

(1*S*,3*S*,5*R*)-1-benzyl-3-(4-chlorophenyl)-6-oxa-2-azabicyclo[3.2.1]octan-7-one

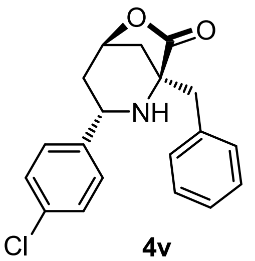

4v

Yield (61%); white solid; $[\alpha]^{20}_D$ = +23.4 (*c* 0.5, acetone); mp 120–122 °C; ^{1}H NMR (400 MHz, $CDCl_3$) δ 7.37 – 7.22 (m, 9H), 4.75 (t, *J* = 5.3 Hz, 1H), 4.13 (dd, *J* = 11.1, 4.9 Hz, 1H), 3.14 (d, *J* = 13.7 Hz, 1H), 2.93 (d, *J* = 13.7 Hz, 1H), 2.28 – 2.13 (m, 1H), 2.08 – 2.03 (m, 1H), 1.93 (brs, 1H), 1.80 (d, *J* = 11.5 Hz, 1H), 1.55 (dd, *J* = 13.4, 11.4 Hz, 1H). ^{13}C NMR (100 MHz, $CDCl_3$) δ 177.2, 141.2, 135.3, 133.4, 130.4, 128.7, 128.4, 128.3, 126.9, 75.1, 62.7, 54.7, 39.63, 39.56, 37.9. HRMS (ESI-TOF) Calcd. For $C_{19}H_{19}ClNO_2^+$ ($[M+H]^+$): 328.1099, found: 328.1092. >20:1 dr. The product was analyzed by HPLC to determine the enantiomeric excess: 95% ee (Chiralpak AD-H, *i*-propanol/hexane = 15/85, flow rate 1.0 mL/min, λ = 210 nm); t_r = 8.6 and 13.0 min.

(1*R*,3*S*,5*R*)-3-(4-chlorophenyl)-1-phenyl-6-oxa-2-azabicyclo[3.2.1]octan-7-one

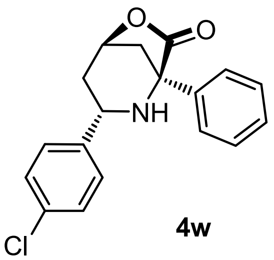

4w

Yield (89%); colorless gum; $[\alpha]^{20}_D$ = -33.6 (*c* 0.5, acetone); ^{1}H NMR (400 MHz, $CDCl_3$) δ 7.66 – 7.57 (m, 2H), 7.46 – 7.37 (m, 4H), 7.37 – 7.29 (m, 3H), 4.93 (t, *J* = 5.3 Hz, 1H), 4.28 (dd, *J* = 11.1, 4.9 Hz, 1H), 2.61 – 2.48 (m, 1H), 2.40 – 2.16 (m, 3H), 1.69 (dd, *J* = 13.6, 11.3 Hz, 1H). ^{13}C NMR (100 MHz, $CDCl_3$) δ 176.0, 141.1, 138.1, 133.5, 128.7, 128.6, 128.4, 128.3, 126.3, 74.7, 64.2, 55.0, 44.8, 37.6. HRMS (ESI-TOF) Calcd. For $C_{18}H_{17}ClNO_2^+$ ($[M+H]^+$): 314.0942, found: 314.0941. >20:1 dr. The product was analyzed by HPLC to determine the enantiomeric excess: 88% ee (Chiralpak AD-H, *i*-propanol/hexane = 15/85, flow rate 1.0 mL/min, λ = 210 nm); t_r = 9.1 and 10.6 min.

methyl 3-((1*S*,3*S*,5*R*)-3-(4-chlorophenyl)-7-oxo-6-oxa-2-azabicyclo[3.2.1]octan-1-yl)propanoate

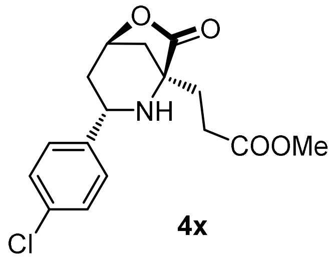

4x

Yield (77%); light yellow gum; $[\alpha]^{20}_{D}$ = -12.2 (*c* 0.5, acetone); ^{1}H NMR (400 MHz, $CDCl_3$) δ 7.36 – 7.27 (m, 4H), 4.86 (t, *J* = 5.3 Hz, 1H), 4.11 (dd, *J* = 11.0, 4.8 Hz, 1H), 3.67 (s, 3H), 2.64 – 2.39 (m, 2H), 2.28 – 2.10 (m, 3H), 2.01 – 1.88 (m, 2H), 1.77 (brs, 1H), 1.54 (dd, *J* = 13.4, 11.4 Hz, 1H). ^{13}C NMR (100 MHz, $CDCl_3$) δ 176.9, 173.3, 141.2, 133.4, 128.7, 128.2, 75.1, 60.9, 54.5, 51.8, 40.1, 37.7, 28.8, 28.6. HRMS (ESI-TOF) Calcd. For $C_{16}H_{19}ClNO_4^+$ ([M+H]$^+$): 324.0997, found: 324.0992. >20:1 dr. The product was analyzed by HPLC to determine the enantiomeric excess: 95% ee (Chiralpak AD-H, *i*-propanol/hexane = 25/75, flow rate 1.0 mL/min, λ = 210 nm); t_r = 9.3 and 14.2 min.

(1*R*,3*S*,5*R*)-3-(4-chlorophenyl)-1-(2-(methylthio)ethyl)-6-oxa-2-azabicyclo[3.2.1]octan-7-one

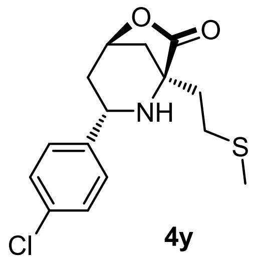

4y

Yield (45%); yellow gum; $[\alpha]^{20}_{D}$ = -8.0 (*c* 0.5, acetone); ^{1}H NMR (400 MHz, $CDCl_3$) δ 7.35 – 7.29 (m, 4H), 4.87 (t, *J* = 5.3 Hz, 1H), 4.12 (dd, *J* = 11.1, 4.8 Hz, 1H), 2.83 – 2.50 (m, 2H), 2.30 – 2.18 (m, 2H), 2.14 (s, 3H), 2.09 – 1.92 (m, 3H), 1.80 (brs, 1H), 1.58 (dd, *J* = 13.4, 11.3 Hz, 1H). ^{13}C NMR (100 MHz, $CDCl_3$) δ 177.1, 141.1, 133.4, 128.7, 128.3, 75.1, 61.2, 54.7, 40.3, 37.8, 33.4, 28.4, 15.6. HRMS (ESI-TOF) Calcd. For $C_{15}H_{19}ClNO_2S^+$ ([M+H]$^+$): 312.0820, found: 312.0816. >20:1 dr. The product was analyzed by HPLC to determine the enantiomeric excess: 89% ee (Chiralpak AD-H, *i*-propanol/hexane = 15/85, flow rate 1.0 mL/min, λ = 210 nm); t_r = 10.7 and 14.3 min.

1.4 Gram-scale synthesis and synthetic transformation

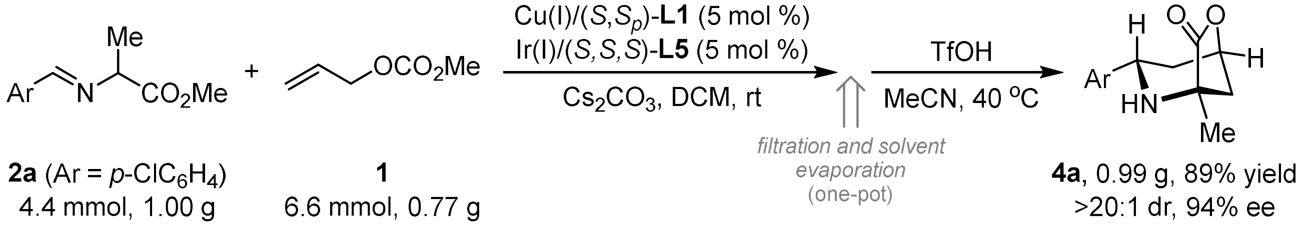

Supplementary Figure 1. Procedure for one-pot synthesis of compound **4a** on gram-scale

Procedure for one-pot synthesis of compound 4a on gram-scale: A flame dried Schlenk tube was cooled to rt and filled with Ar. To this flask were added $[Ir(COD)Cl]_2$ (0.066 mmol, 1.5 mol %), phosphoramidite ligand (*S,S,S*)-**L5** (0.132 mmol, 3 mol %), degassed THF (10 mL) and degassed *n*-propylamine (10 mL). The reaction mixture was stirred at 50 °C for 30 min and then the volatile solvents were removed in vacuum to give a pale yellow solid. Meanwhile, in a separated Schlenk tube, (S,S_p)-*i*Pr-Phosferrox-**L1** (0.24 mmol, 5.5 mol %) and $Cu(MeCN)_4BF_4$ (0.22 mmol, 5 mol %) were dissolved in DCM (10 mL), and stirred at room temperature for about 0.5 h. A solution of aldimine ester **1** (1.00 g, 4.42 mmol) in DCM (10 mL) was added, and then Cs_2CO_3 (2.20 g, 6.75 mmol), allylic carbonate **4** (6.60 mmol) in DCM (10 mL) and pre-prepared Ir-complex in DCM (5 mL) were added successively. The reaction mixture was stirred at 25 °C for 14 h before filtration. The filtrate was concentrated in vacuum and refilled with MeCN (44 mL). TfOH (1.4 mL, 15.87 mmol) was added drop-wise. The reaction mixture was stirred at 40 °C for 3 days, basified with Et_3N (3 mL) and then concentrated in vacuum. Column chromatography on silica gel (PE: EtOAc = 6: 1) afforded the lactone **4a** (0.99 g, 89% overall yield, >20: 1 dr, 94% ee) as a white solid.

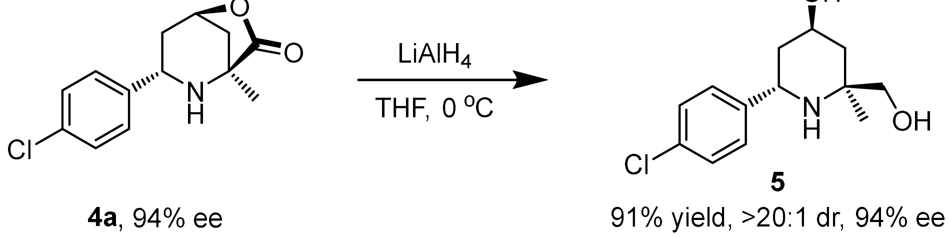

Supplementary Figure 2. Procedure for synthesis of compound **5**

Procedure for synthesis of compound 5: A flame dried Schlenk tube was cooled to rt and filled with Ar. To this flask were added $LiAlH_4$ (23 mg, 0.6 mmol, 3 eq.) and dry THF (1 mL). The suspension was cooled to 0 °C, and then **4a** (50 mg, 0.2 mmol) in THF (1 mL) was added drop-wise. The reaction mixture was stirred at 0 °C for 2 h, and then water (50 μL), 15% NaOH (aq., 50 μL) and water (100 μL) were added successively. The resulting mixture was purified by column chromatography on silica gel (PE: EtOAc = 5: 1 to 2: 1) to afford the diol **5** (46 mg, 91% yield, >20: 1 dr, 94% ee) as a white solid. mp 142–143 °C; $[\alpha]^{20}_{D}$ = -27.8 (*c* 0.5, acetone); ^1H NMR (400 MHz, CD_3OD) δ 7.42 – 7.38 (m, 2H), 7.33 – 7.30 (m, 2H), 4.32 (d, *J* = 11.3 Hz, 1H), 4.23 (dd, *J* = 11.7, 2.5 Hz, 1H), 4.20 – 4.14 (m, 1H), 3.35 (d, *J* = 11.3 Hz, 1H), 1.86 – 1.78 (m, 1H), 1.77 – 1.67 (m, 2H), 1.59 – 1.54 (m, 1H), 1.09 (s, 3H). ^{13}C NMR (100 MHz, MeOD) δ 144.4, 133.8, 129.7, 129.5, 66.3, 66.1, 55.3, 50.3, 40.6, 40.2, 27.9. HRMS (ESI-TOF) Calcd. For $C_{13}H_{19}ClNO_2^+$ ($[M+H]^+$): 256.1099, found: 256.1096. >20:1 dr. The product was analyzed by HPLC to determine the enantiomeric excess: 94% ee (Chiralpak AD-H, *i*-propanol/hexane = 25/75, flow rate 1.0 mL/min, λ = 210 nm); t_r = 4.4 and 4.7 min.

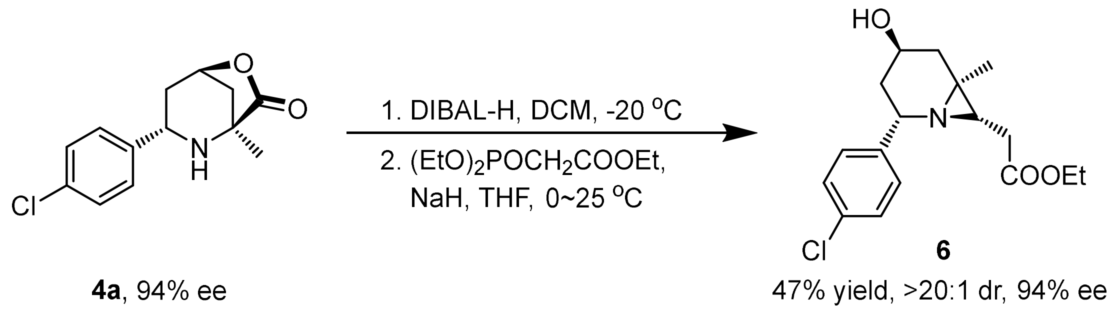

Supplementary Figure 3. Procedure for synthesis of compound **6**

Procedure for synthesis of compound 6: A flame dried Schlenk tube was cooled to rt and filled with Ar. To this flask were added **4a** (50 mg, 0.2 mmol) and dry DCM (2 mL). The solution was cooled to -20 °C, and then DIBAL-H (1.3 M in toluene, 0.4 mL, 0.5 mmol, 2.5 eq.) was added drop-wise. The reaction mixture was stirred at -20 °C for 1 h and quenched by sat. potassium sodium tartrate (3 mL). The resulting mixture was stirred at 30 °C for 0.5 h until two clear phases formed. The mixture was extracted with DCM (3 mL × 3). The combined organic phase was washed with brine (5 mL), dried over $MgSO_4$, filtered and concentrated in vacuum to afford a white solid, which

was used directly for the next step.

To another Schlenk tube filled with Ar was added NaH (60% in mineral oil, 10 mg, 0.25 mmol) and THF (1 mL). The mixture was cooled to 0 ºC, $(EtO)_2POCH_2COOEt$ (70 mg, 0.31 mmol) was added. After stirred at 25 ºC for 0.5 h, the pre-prepared hemiacetal in THF (1 mL) was added drop-wise. The reaction mixture was stirred at 25 ºC for 3 h, and then quenched by sat. NH_4Cl (5 mL). The mixture was extracted with EtOAc (3 mL × 3). The combined organic phase was washed with brine (5 mL), dried over Na_2SO_4, filtered and concentrated in vacuum. The residue was purified by column chromatography on silica gel (PE: EtOAc = 5: 1 to 3: 1) to afford the product **6** (30 mg, 46% yield over 2 steps, >20: 1 dr, 94% ee) as a yellow oil. $[\alpha]^{20}_{D}$ = -27.0 (*c* 0.5, acetone); ^{1}H NMR (400 MHz, $CDCl_3$) δ 7.40 – 7.31 (m, 2H), 7.29 – 7.24 (m, 2H), 4.52 (t, *J* = 5.7 Hz, 1H), 4.39 (dd, *J* = 10.5, 5.3 Hz, 1H), 4.23 – 4.17 (m, 3H), 2.80 (dd, *J* = 15.6, 8.8 Hz, 1H), 2.55 (dd, *J* = 15.6, 4.2 Hz, 1H), 2.11 – 2.06 (m, 1H), 2.03 – 1.96 (m, 1H), 1.73 (d, *J* = 11.0 Hz, 1H), 1.35 (dd, *J* = 13.1, 10.5 Hz, 1H), 1.29 (t, *J* = 7.1 Hz, 3H), 1.16 (s, 3H). ^{13}C NMR (100 MHz, $CDCl_3$) δ 171.6, 143.4, 132.5, 128.4, 128.0, 82.4, 74.7, 60.7, 59.7, 53.6, 45.1, 40.2, 33.3, 21.6, 14.1. HRMS (ESI-TOF) Calcd. For $C_{17}H_{23}ClNO_3^+$ ($[M+H]^+$): 324.1361, found: 324.1359. >20:1 dr. The product was analyzed by HPLC to determine the enantiomeric excess: 94% ee (Chiralpak IC, *i*-propanol/hexane = 10/90, flow rate 1.0 mL/min, λ = 210 nm); t_r = 10.7 and 16.5 min.

2. Supplementary Discussion

2.1 Absolute configuration determination of (1*S*,3*S*,5*R*)-4a and (1*R*,3*R*,5*S*)-4a

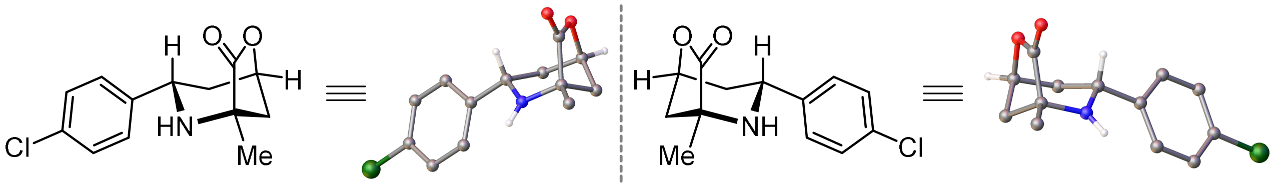

(1*S*,3*S*,5*R*)-**4a** (CCDC 2256426) (1*R*,3*R*,5*S*)-**4a** (CCDC 2256427)

Supplementary Figure 4. ORTEP representation of (1*S*,3*S*,5*R*)-**4a** and (1*R*,3*R*,5*S*)-**4a**

Crystal data for (1*S*,3*S*,5*R*)-**4a**: $C_{13}H_{14}ClNO_2$, M_r = 251.71, T = 285 K, monoclinic, space group P 1 21 1, a= 8.2011(6), b= 7.4032(5), c= 11.0356(6) Å, α = 90, β = 107.450(7), γ = 90, V = 639.18(7) Å^3, Z = 2, 1935 unique reflections, final R_1 = 0.0429 and wR_2 = 0.1303 for 2238 observed [I>2σ(I)] reflections, Flack χ= -0.001(12).

Crystal data for (1*R*,3*R*,5*S*)-**4a**: $C_{13}H_{14}ClNO_2$, M_r = 251.71, T = 284 K, monoclinic, space group P 1 21 1, a= 8.1957(6), b= 7.4165(4), c= 11.0268(7) Å, α = 90, β = 107.491(7), γ = 90, V = 639.26(7) Å^3, Z = 2, 1977 unique reflections, final R_1 = 0.0396 and wR_2 = 0.1131 for 2227 observed [I>2σ(I)] reflections, Flack χ= 0.021(13). CCDC 2256426 ((1*S*,3*S*,5*R*)-**4a**) and 2256427 ((1*R*,3*R*,5*S*)-**4a**) contains the supplementary crystallographic data for this paper.

These data can be obtained free of charge via www.ccdc.cam.ac.uk/conts/retrieving.html (or from the Cambridge Crystallographic Data Centre, 12, Union Road, Cambridge CB21EZ, UK; fax: (+44) 1223-336-033; or deposit@ccdc.cam.ac.uk).

2.2 Biological activity study

(1) Cell lines and cell culture conditions

The human tumor cell lines HeLa-60, A549, SMMC-7721, MDA-MB-231, and SW480 were used in the cytotoxic assay. These cell lines were obtained from ATCC (Manassas, VA, USA) and BeNa Culture Collection. Cells were cultured in RMPI-1640 or DMEM medium (Biological Industries, Kibbutz Beit-Haemek, Israel) supplemented with 10% fetal bovine serum (FBS, Biological Industries) at 37 °C in a humidified atmosphere with 5% CO_2.

(2) Cytotoxicity assays

The cytotoxicity assay was evaluated by the 3-(4,5-dimethylthiazol-2-yl)-5-(3-carboxymethoxy-phenyl)-2-(4-sulfophenyl)-2H-tetrazolium, inner salt (MTS) (Promega, Madison, WI, USA) assay. Briefly, cells were seeded into each well of a 96-well cell culture plate. After 12 ~24 h of incubation at 37 °C, the test compound (40 μM) was added. After incubated for 48 h, cells were subjected to the MTS assay. Compounds with a growth inhibition rate of 50% were further evaluated at concentrations of 0.064, 0.32, 1.6, 8, and 40 μM in triplicate, with cisplatin (Sigma, St. Louis, MO, USA) as positive control. The IC_{50} value of each compound was calculated with Reed and Muench's method.

2.3 Computational methods

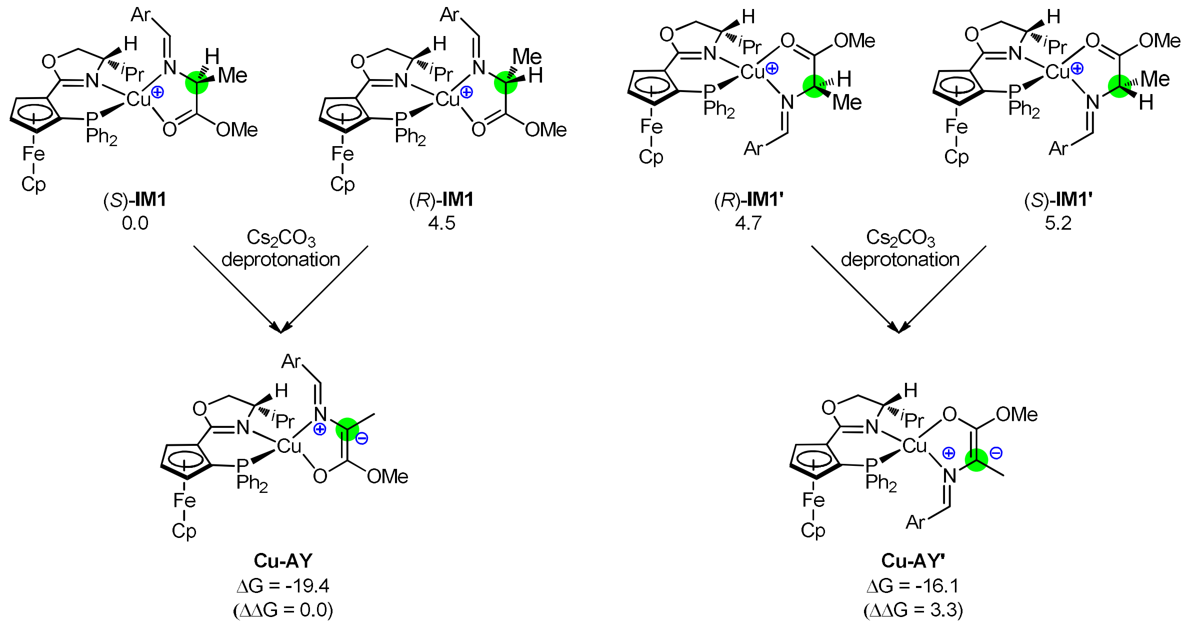

Supplementary Figure 5. The generation of Cu-ylides **Cu-AY** and **Cu-AY'**. Free energies are given in kcal/mol.

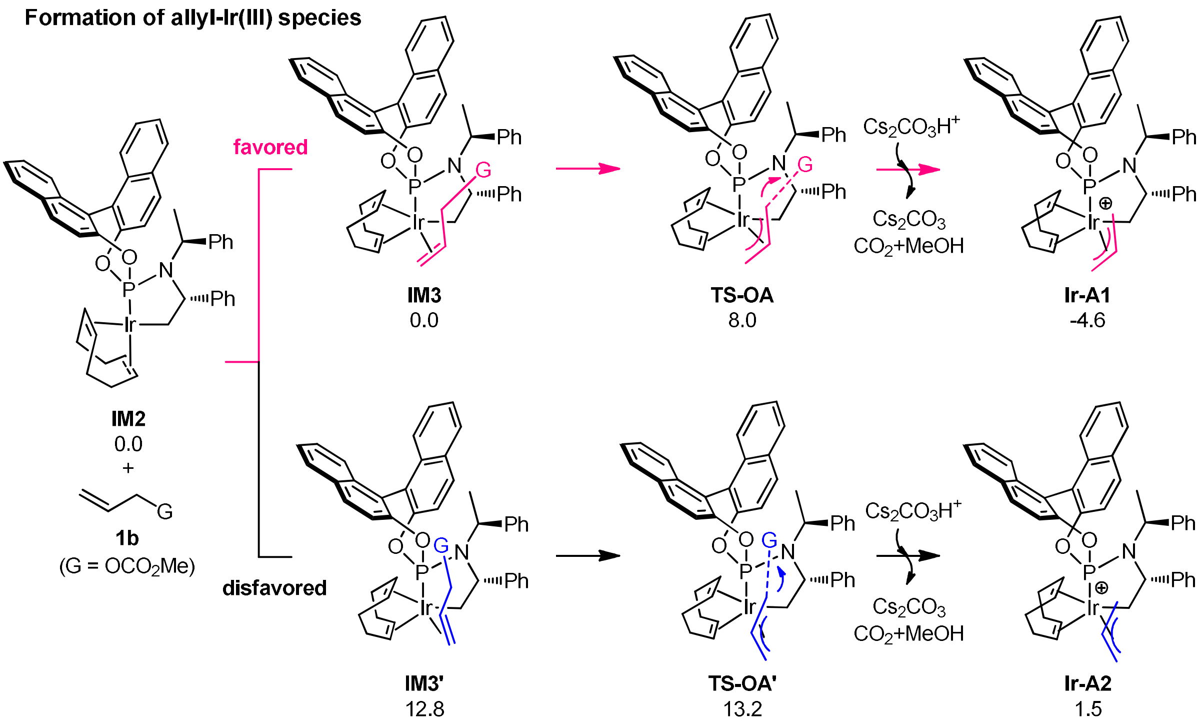

Supplementary Figure 6. The formation of allyl-Ir(III) species **Ir-A1** and **Ir-A2**. Free energies are given in kcal/mol.

Supplementary Table 4. Analysis of Distortion/Interaction Energies for **TS1** and **TS1'**

	ΔE_{dis}	ΔE_{dis}(Ir)	ΔE_{dis}(Cu)	ΔE_{int}	$\Delta E^{\neq}$
**TS1**	17.0	15.3	1.7	-23.3	-6.3
**TS1'**	21.0	16.9	4.1	-21.0	0.0

To further unveil the energy difference between **TS1** and **TS1'**, the distortion/interaction analysis has been carried out. As shown in Supplementary Table 4, in the energy decomposition analysis, the transition state is divided into two fragments, which originates from the Cu(I)-ylide and π-allyl-Ir(III). ΔE_{dist}(**Cu**) and ΔE_{dist}(**Ir**) are the distortion energies of the nucleophile and electrophile (ΔE_{dist}, required to distort the two active catalysts into the TS geometry), respectively. ΔE_{int} (gained upon allowing the distorted fragments to interact) corresponds to the energy difference between the total distortion energy and the activation energy ($\Delta E^{\neq}$). Distortion/interaction energies show that **TS1** experiences less structural distortions in each catalyst and greater interaction energies between the

two catalysts than that of **TS1'**.

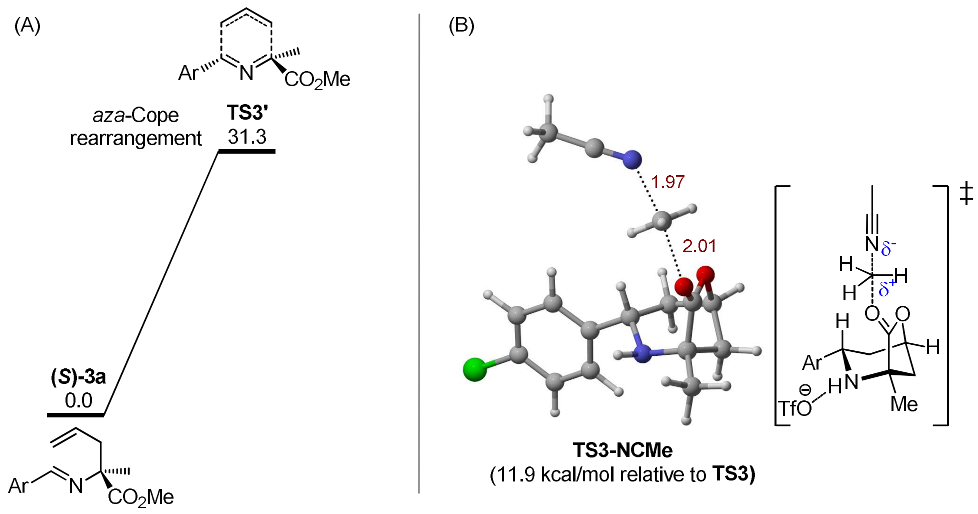

Supplementary Figure 7. (A) Free energy profiles for the direct *aza*-Cope rearrangement of (*S*)-**3a**. (B) Transition states for the leaving of Me^{+} group aided by MeCN. The TfO^{-} moiety was omitted for clarity in the optimized structure of **TS3-CNMe**.

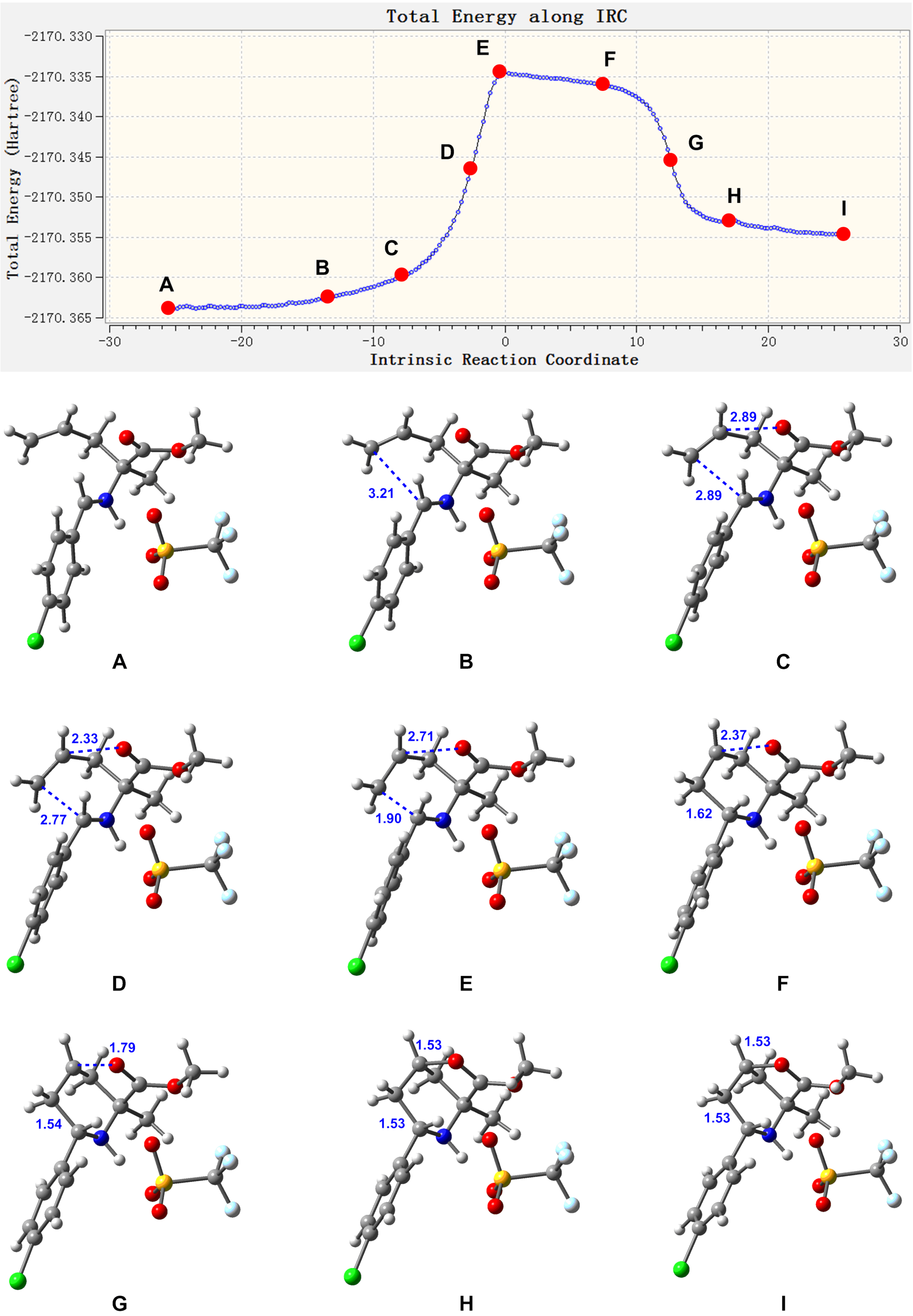

Supplementary Figure 8. Intrinsic reaction coordinate (IRC) analysis for the cyclization transition state **TS2**, with selected C···C and C···O bond distances given in Å.

3. Supplementary Figures

3.1 ^{1}H, ^{13}C, and ^{19}F NMR Spectra of New Compounds

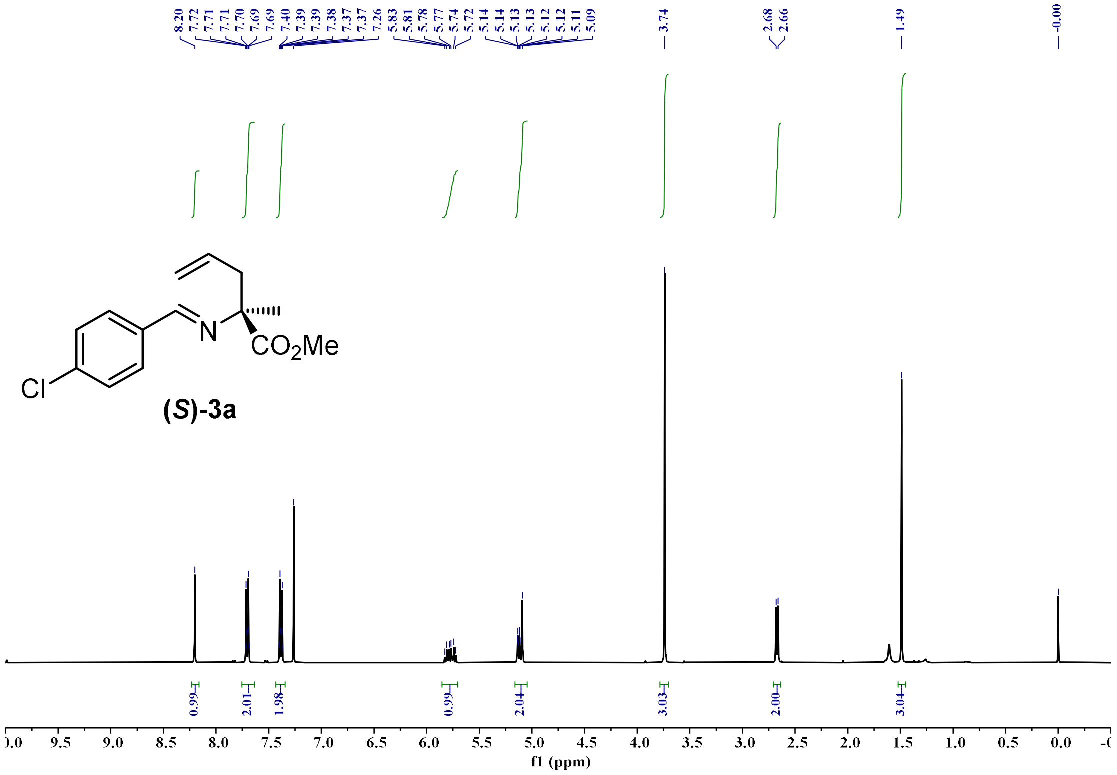

Supplementary Figure 9. ^{1}H NMR spectrum (400 MHz, $CDCl_3$) of (*S*)-**3a**

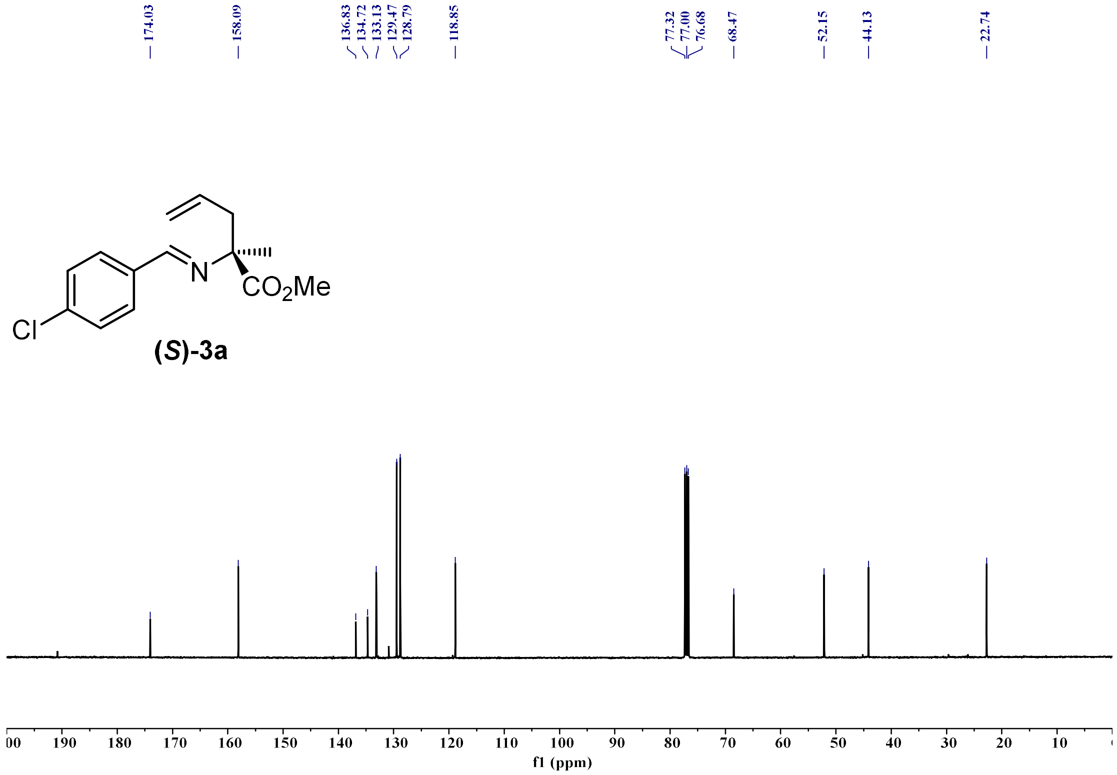

Supplementary Figure 10. ^{13}C NMR spectrum (100 MHz, $CDCl_3$) of (*S*)-**3a**

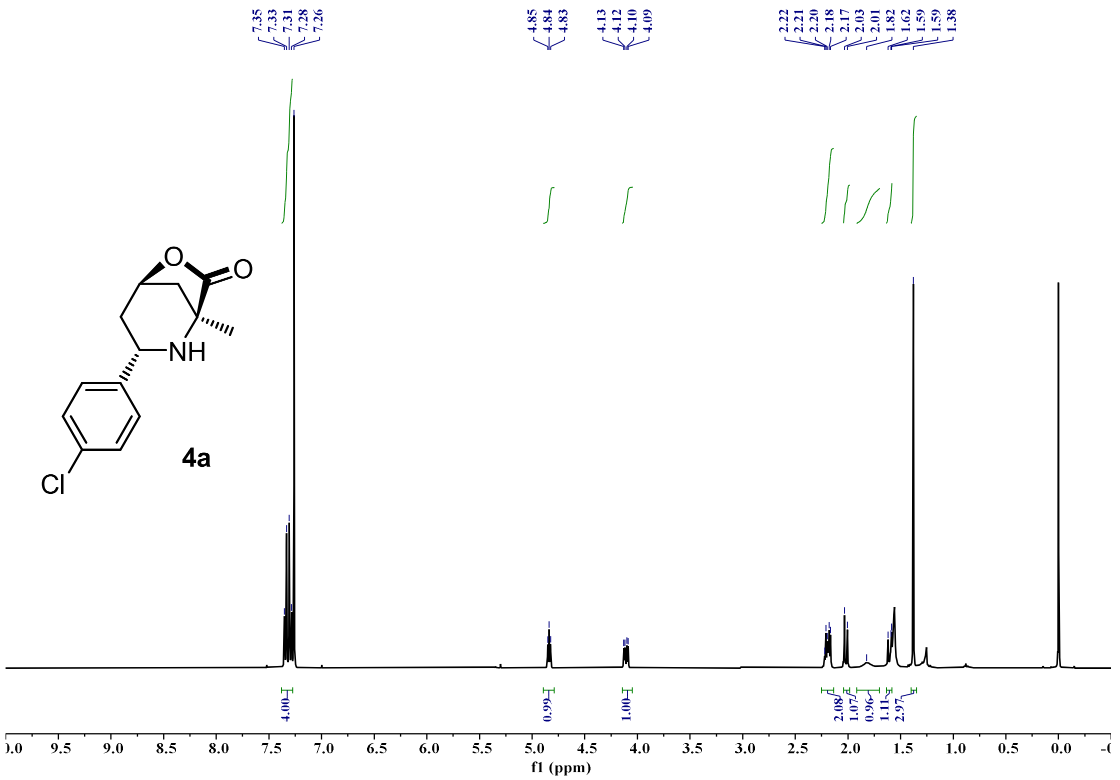

Supplementary Figure 11. ^{1}H NMR spectrum (400 MHz, $CDCl_3$) of **4a**

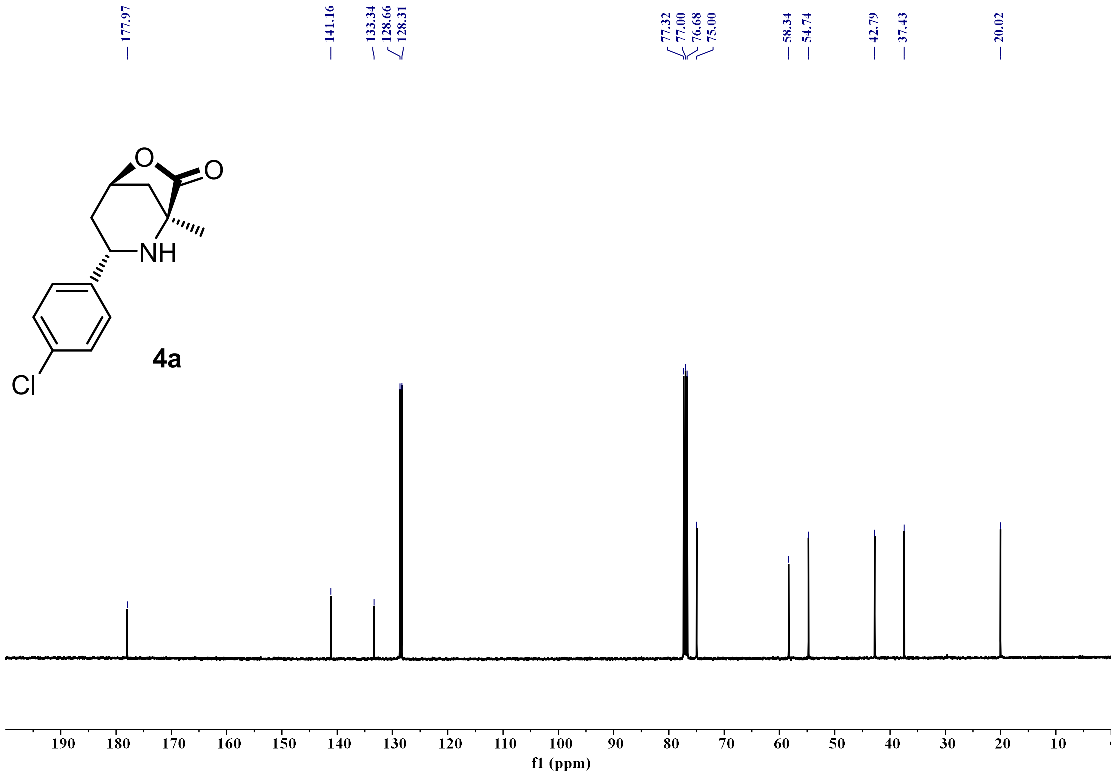

Supplementary Figure 12. ^{13}C NMR spectrum (100 MHz, $CDCl_3$) of **4a**

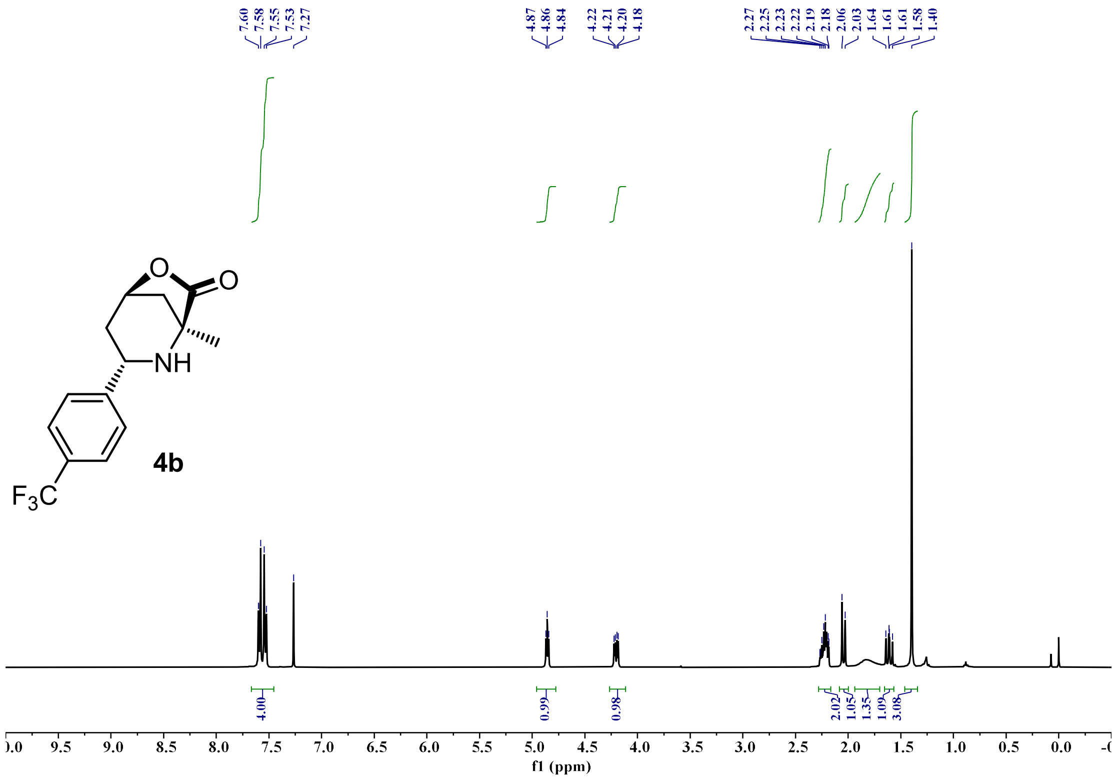

Supplementary Figure 13. ^{1}H NMR spectrum (400 MHz, $CDCl_3$) of **4b**

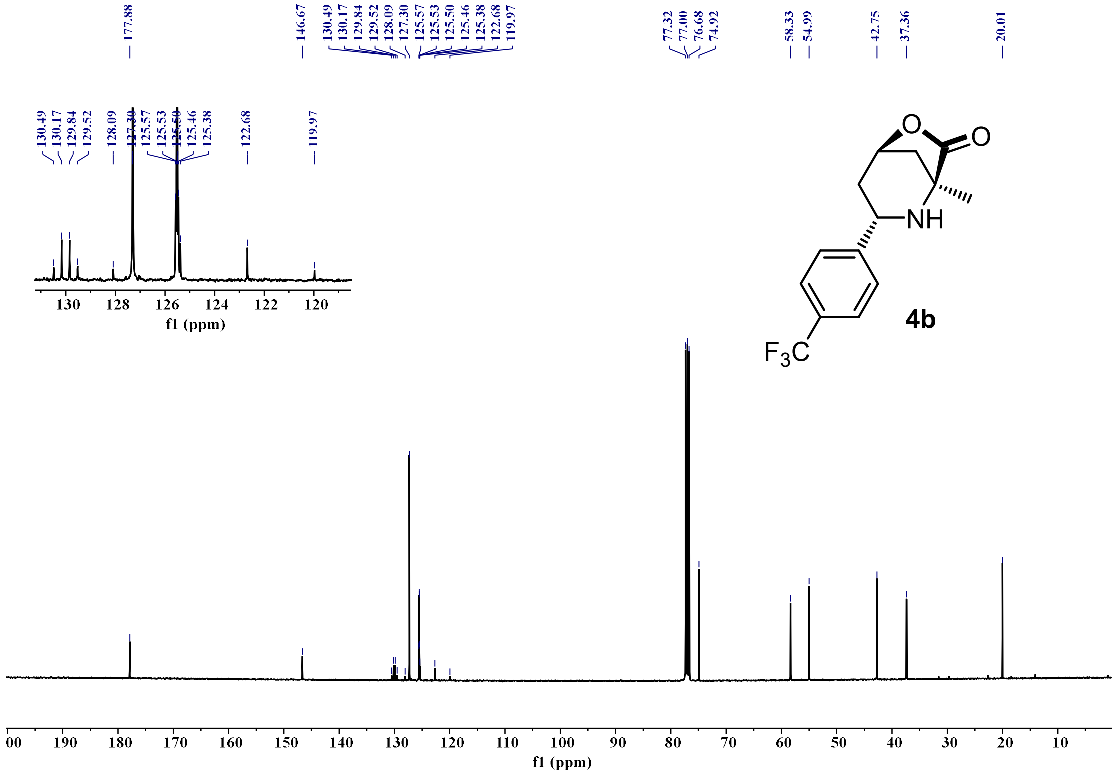

Supplementary Figure 14. ^{13}C NMR spectrum (100 MHz, $CDCl_3$) of **4b**

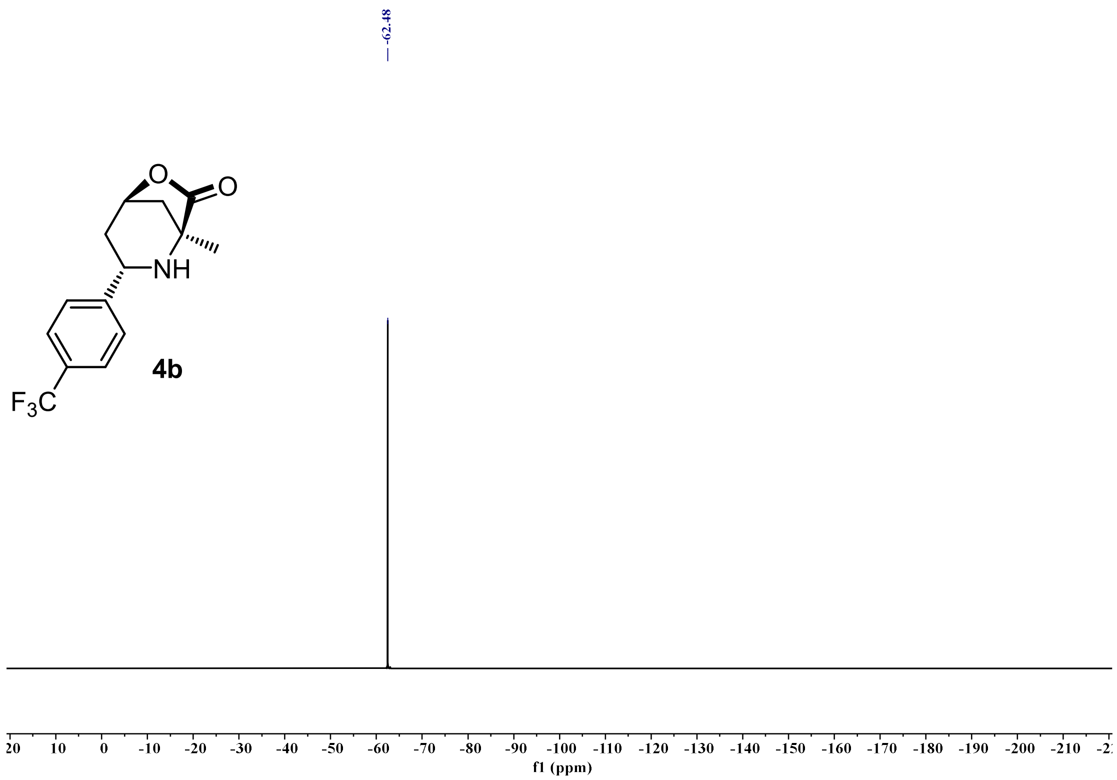

Supplementary Figure 15. ^{19}F NMR (376 MHz, $CDCl_3$) of **4b**

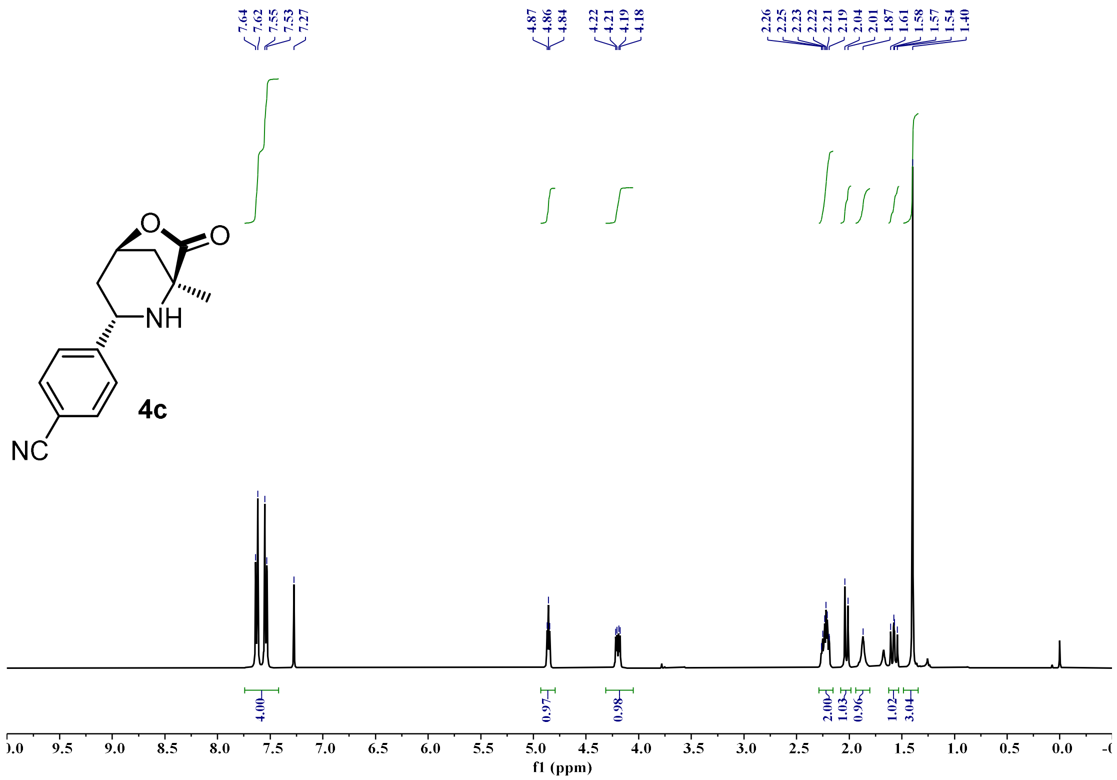

Supplementary Figure 16. ^{1}H NMR spectrum (400 MHz, $CDCl_3$) of **4c**

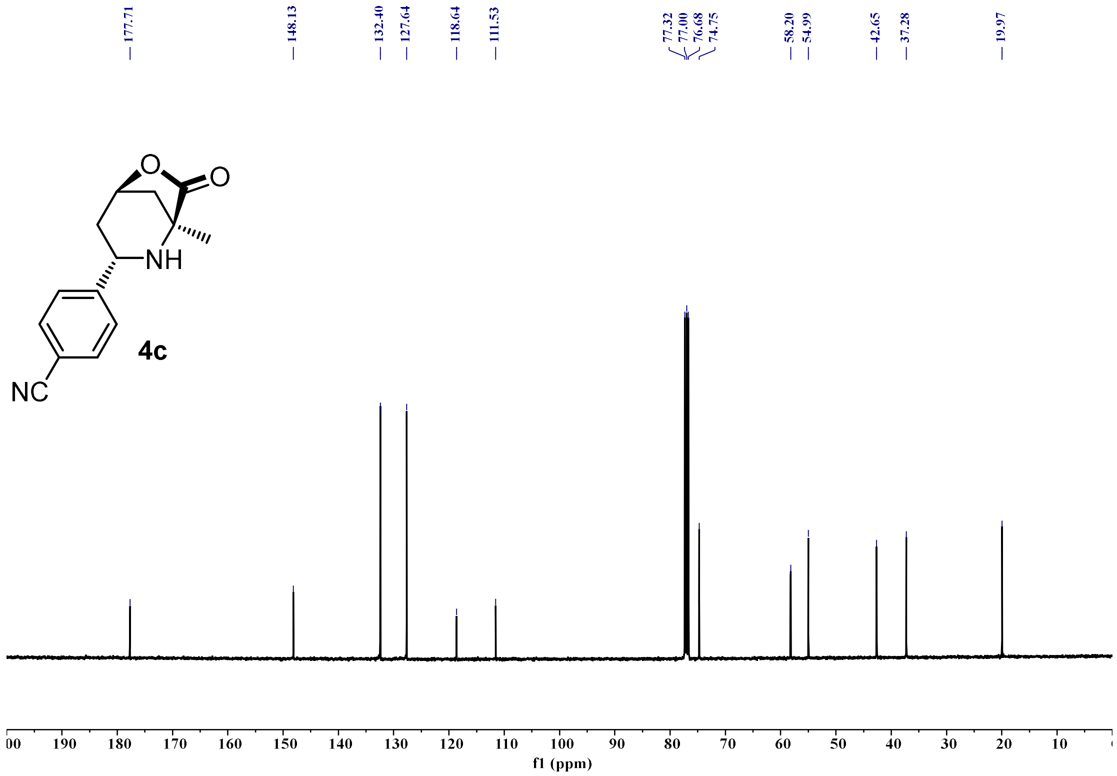

Supplementary Figure 17. ^{13}C NMR spectrum (100 MHz, $CDCl_3$) of **4c**

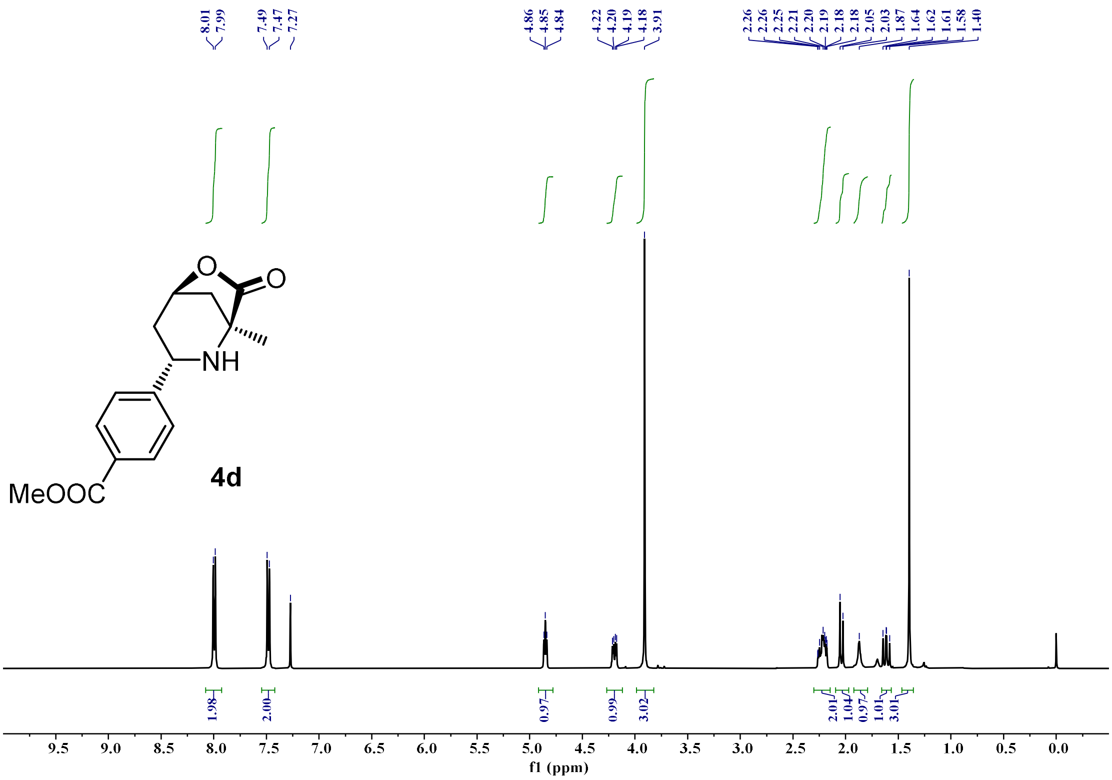

Supplementary Figure 18. ^{1}H NMR spectrum (400 MHz, $CDCl_3$) of **4d**

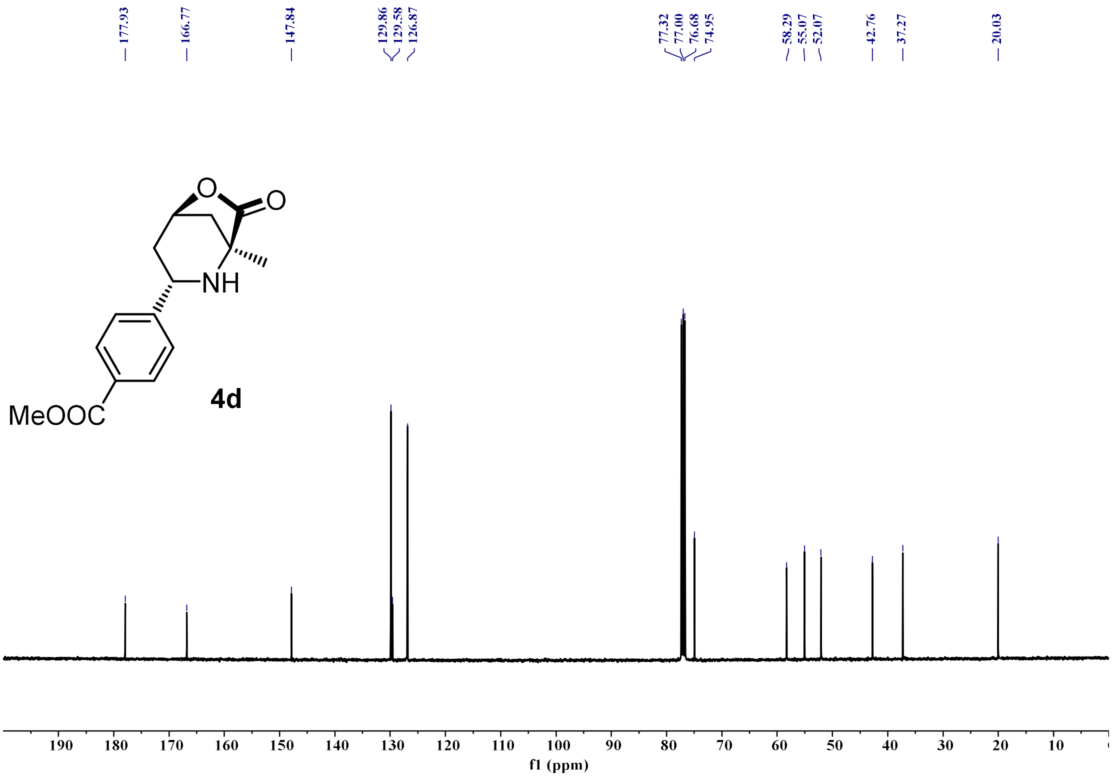

Supplementary Figure 19. ^{13}C NMR spectrum (100 MHz, $CDCl_3$) of **4d**

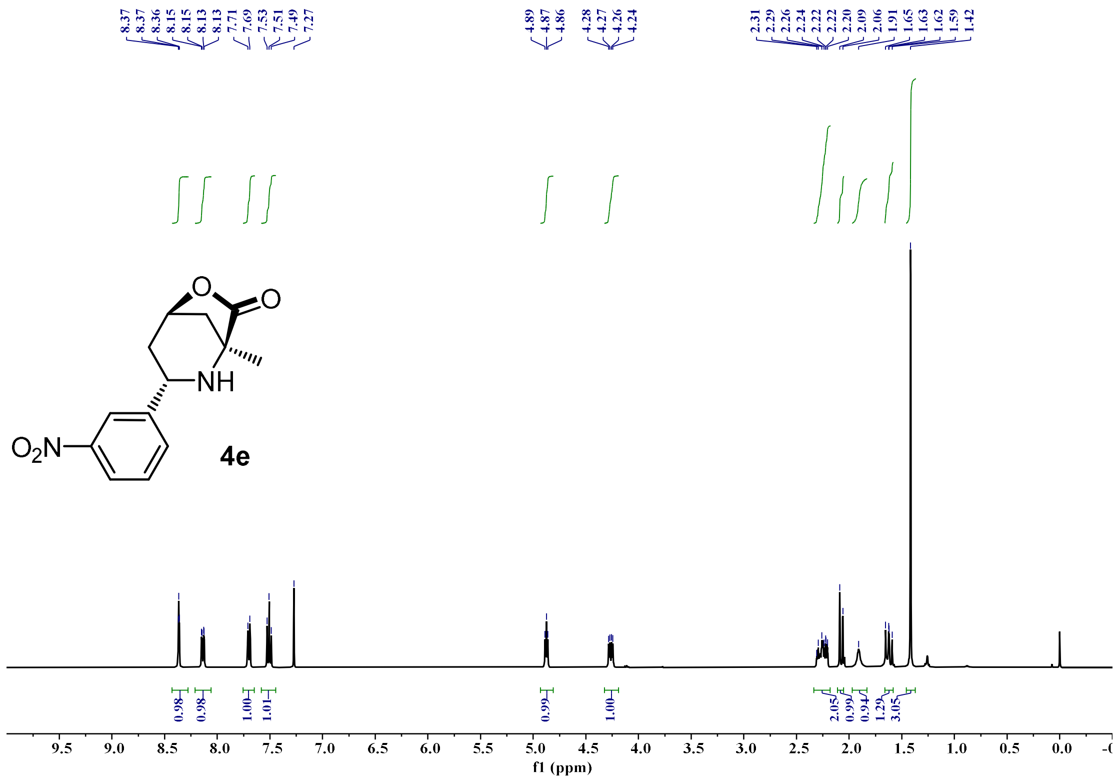

Supplementary Figure 20. ^{1}H NMR spectrum (400 MHz, $CDCl_3$) of **4e**

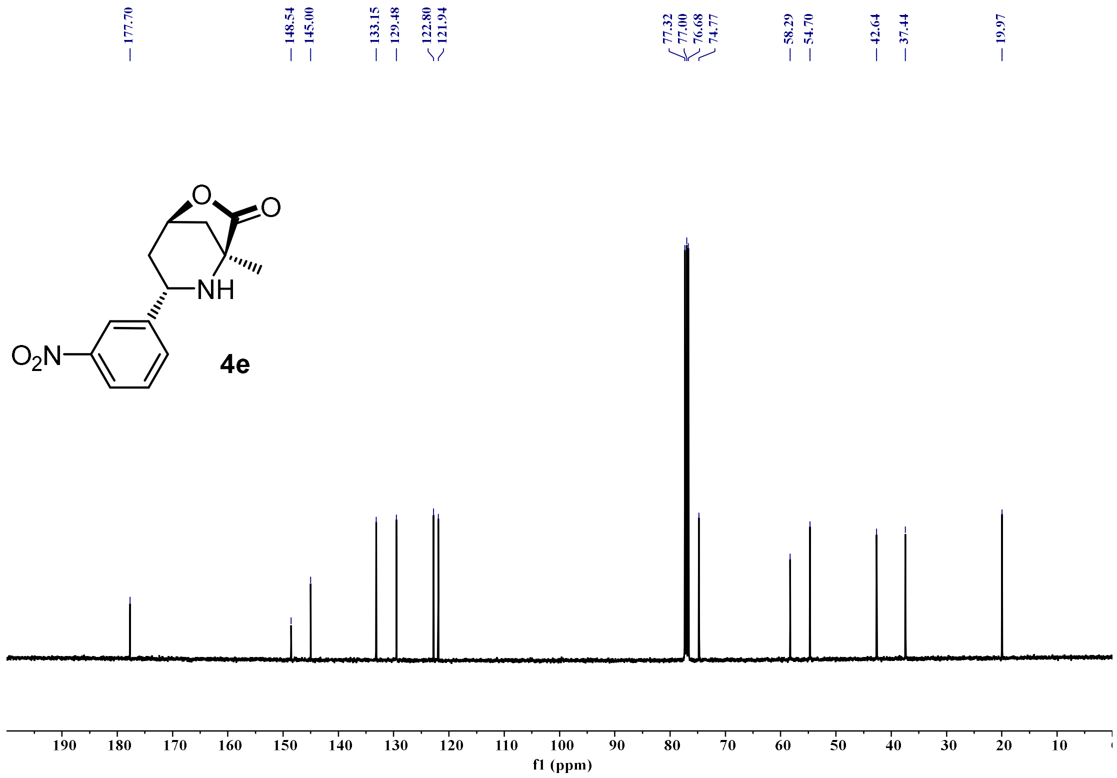

Supplementary Figure 21. ^{13}C NMR spectrum (100 MHz, $CDCl_3$) of **4e**

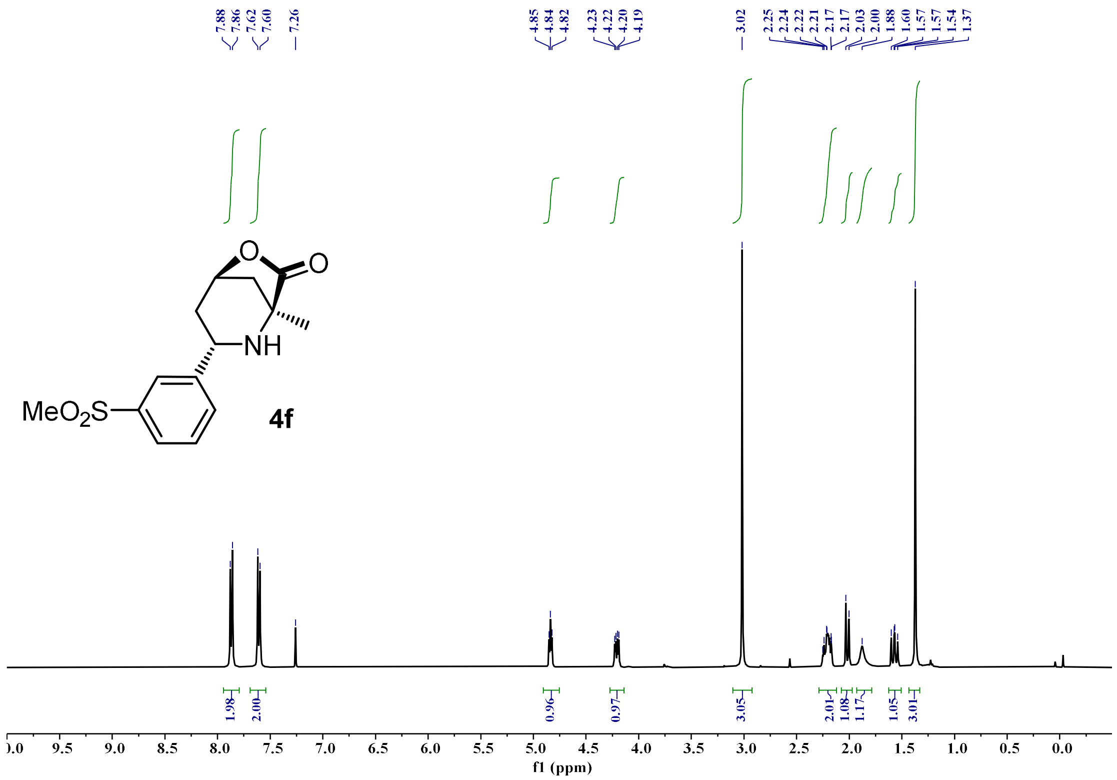

Supplementary Figure 22. ^{1}H NMR spectrum (400 MHz, $CDCl_3$) of **4f**

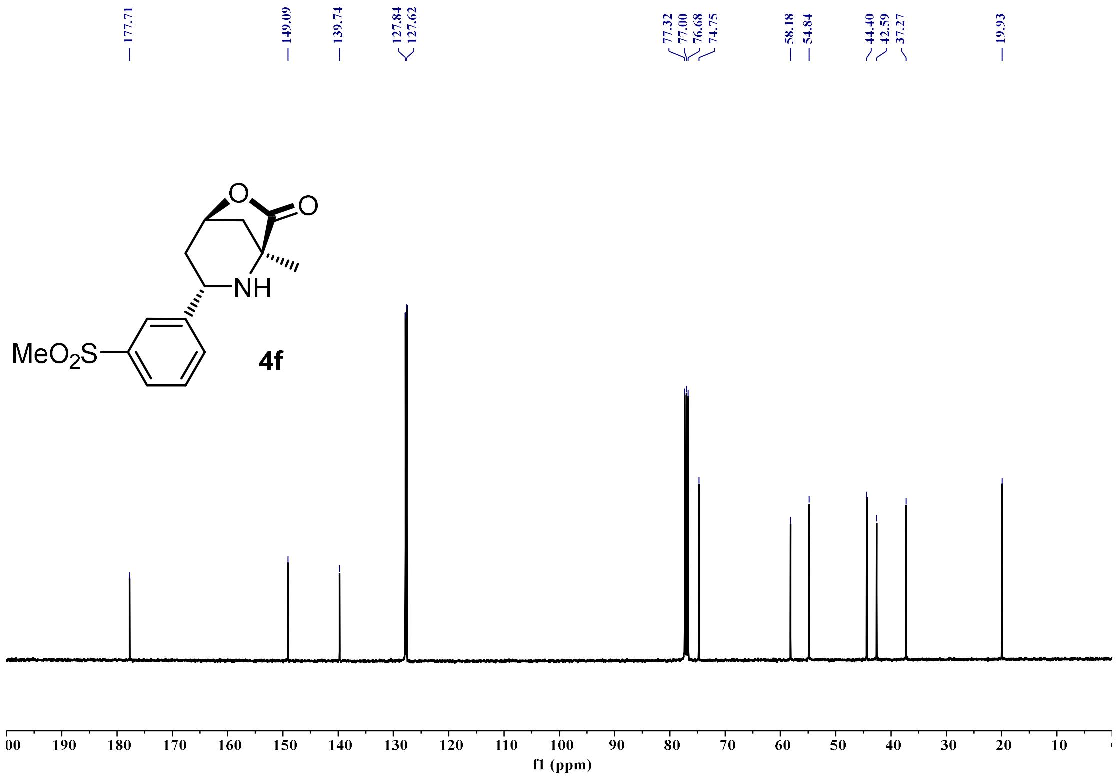

Supplementary Figure 23. ^{13}C NMR spectrum (100 MHz, $CDCl_3$) of **4f**

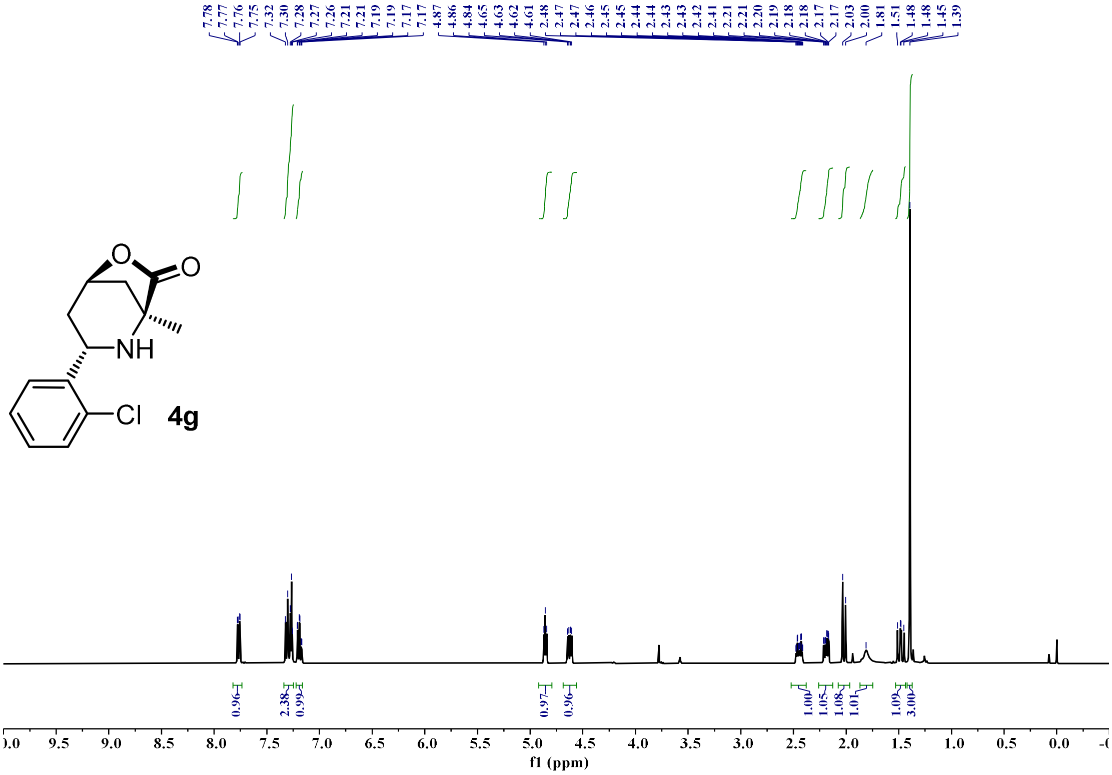

Supplementary Figure 24. ^{1}H NMR spectrum (400 MHz, $CDCl_3$) of **4g**

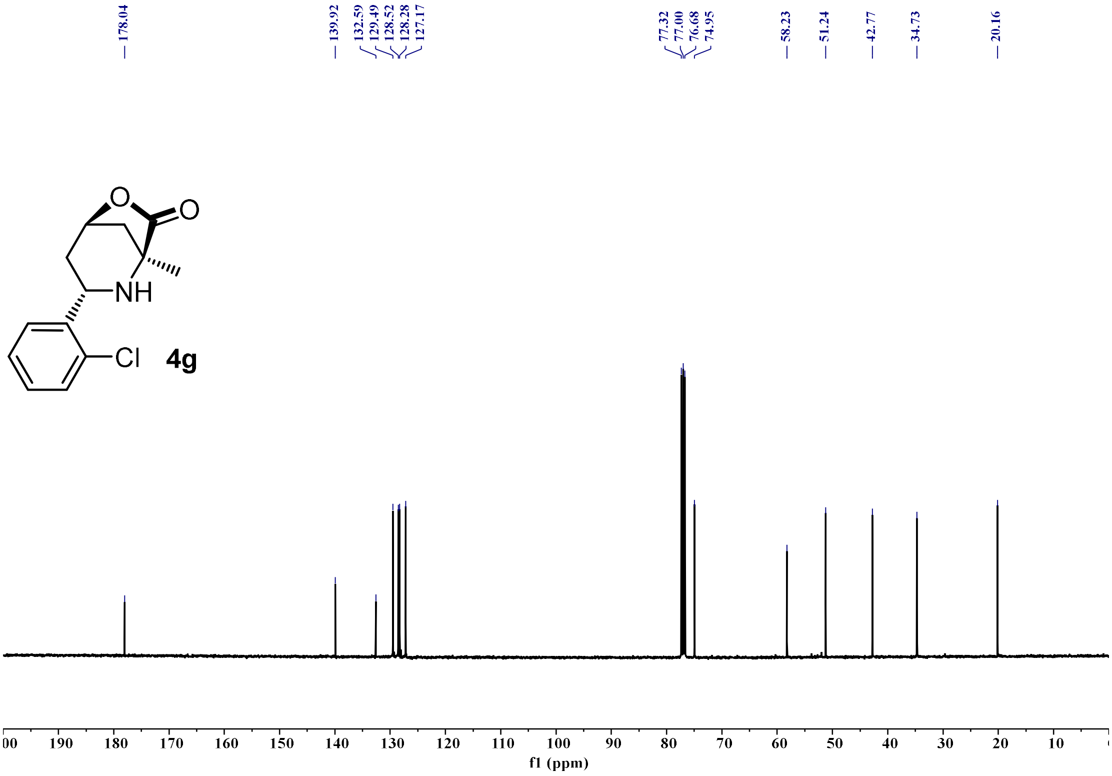

Supplementary Figure 25. ^{13}C NMR spectrum (100 MHz, $CDCl_3$) of **4g**

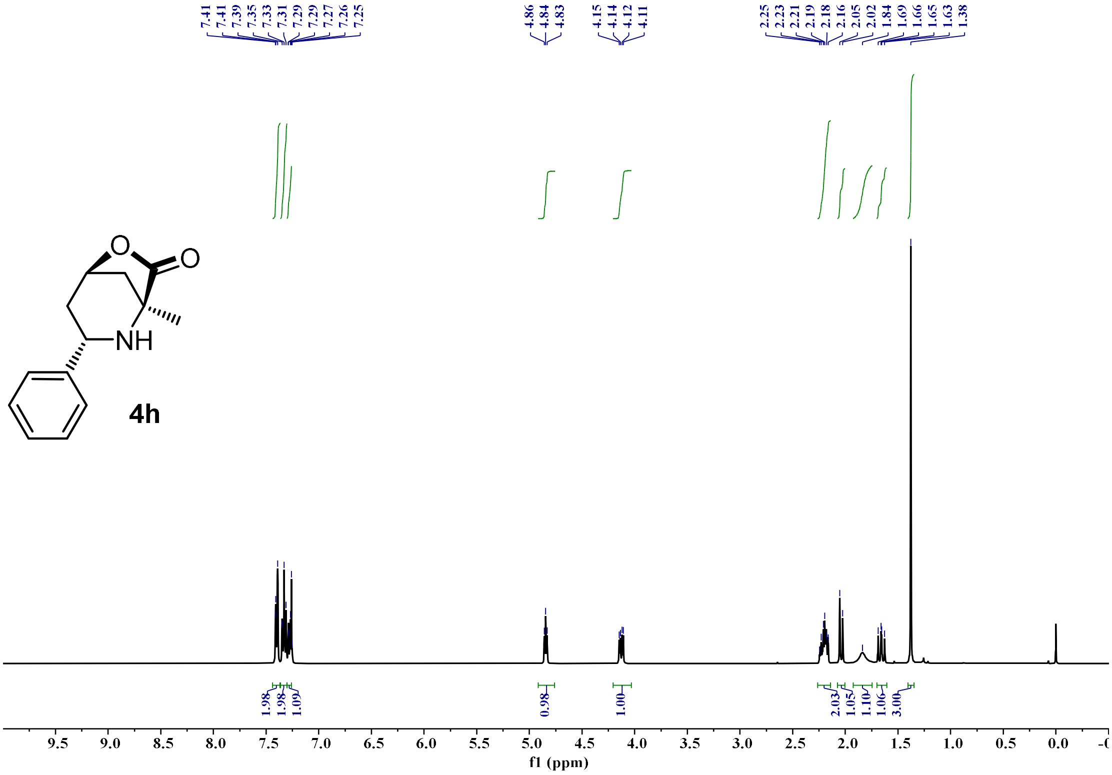

Supplementary Figure 26. ^{1}H NMR spectrum (400 MHz, $CDCl_3$) of **4h**

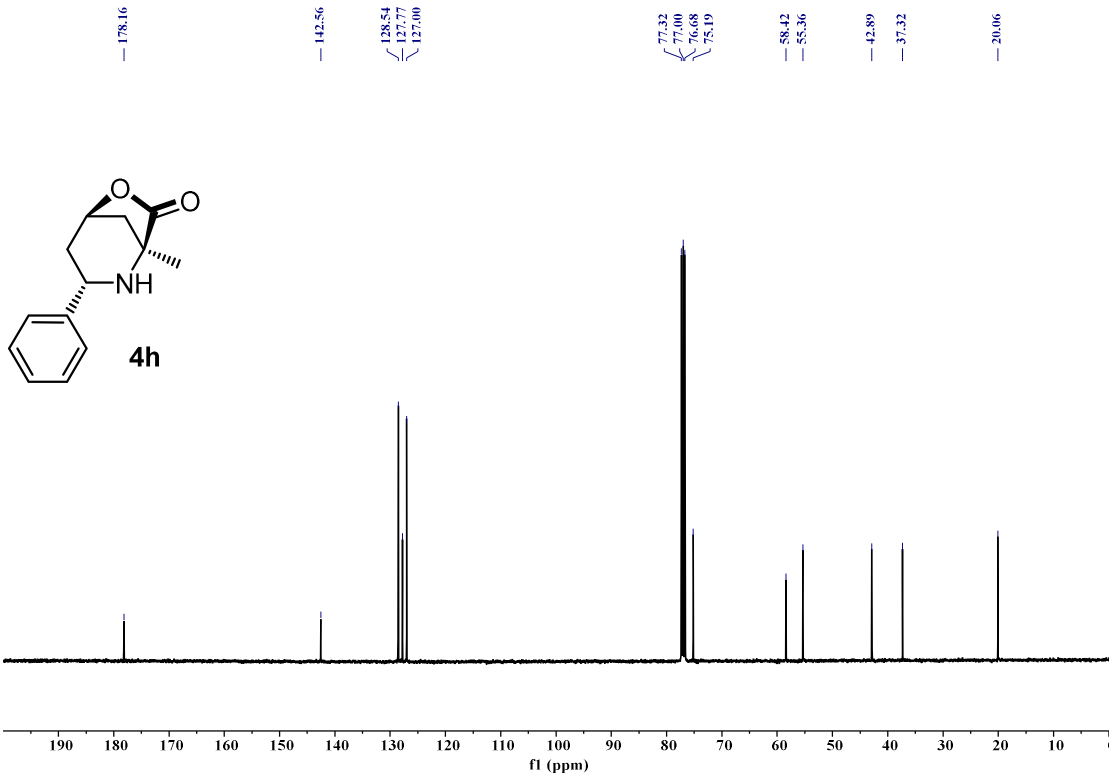

Supplementary Figure 27. ^{13}C NMR spectrum (100 MHz, $CDCl_3$) of **4h**

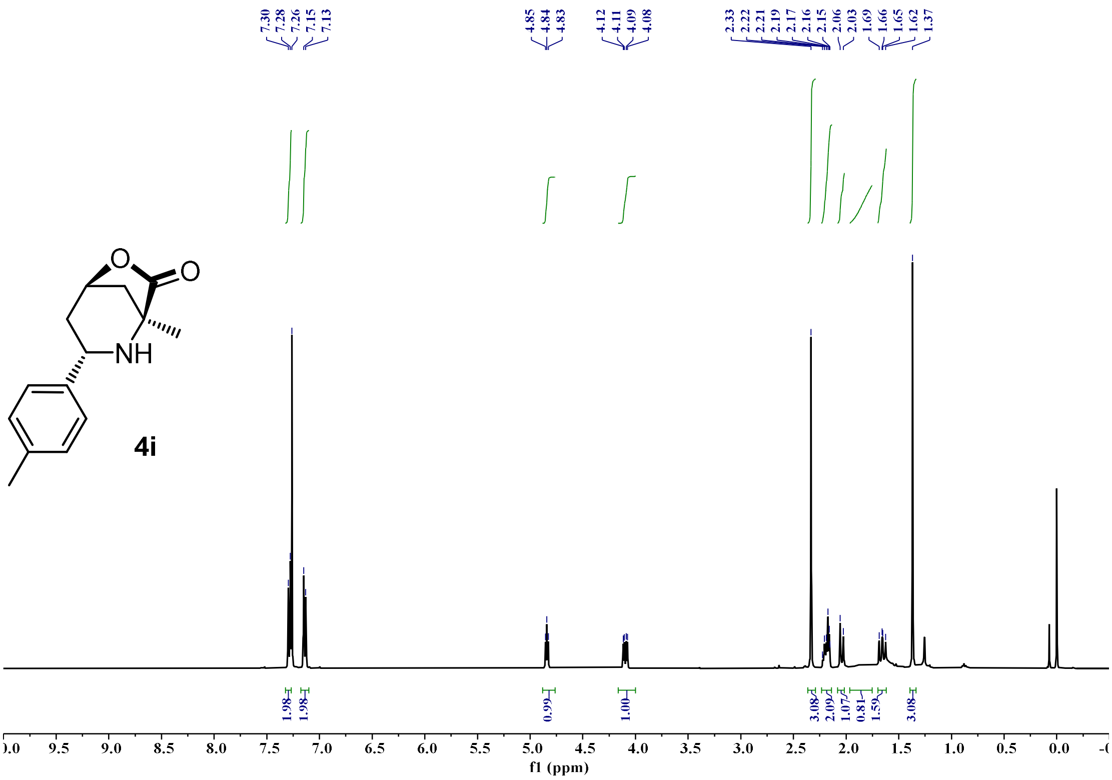

Supplementary Figure 28. ^{1}H NMR spectrum (400 MHz, $CDCl_3$) of **4i**

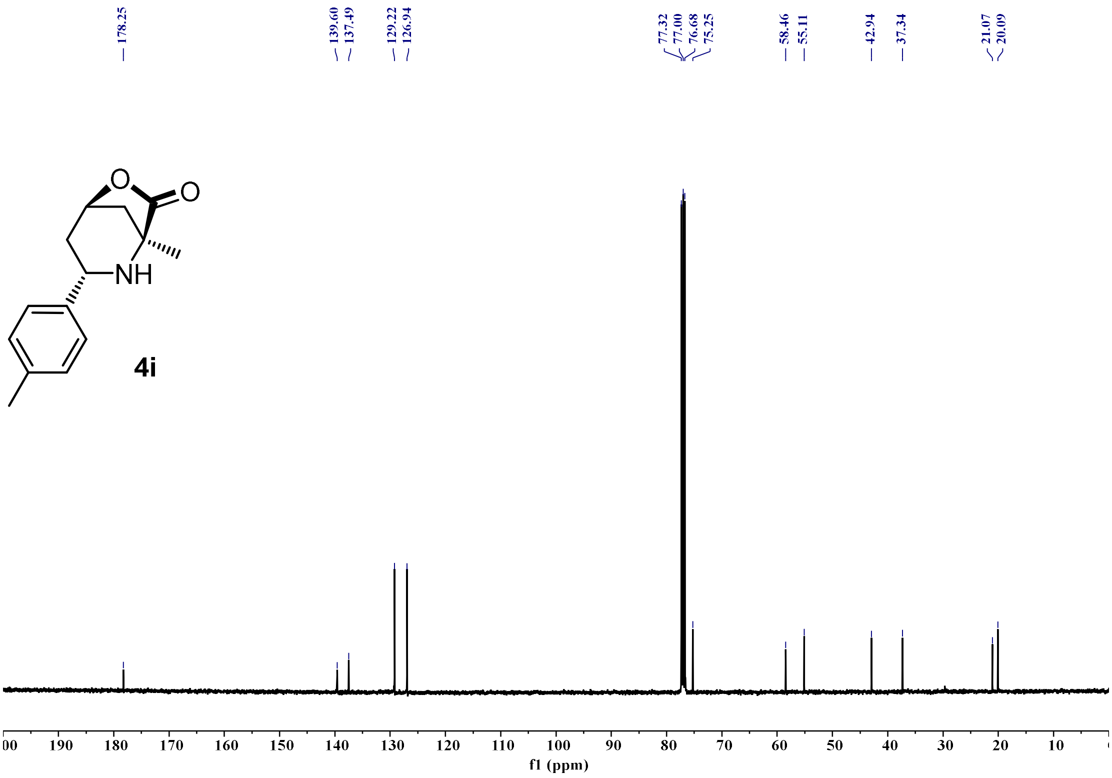

Supplementary Figure 29. ^{13}C NMR spectrum (100 MHz, $CDCl_3$) of **4i**

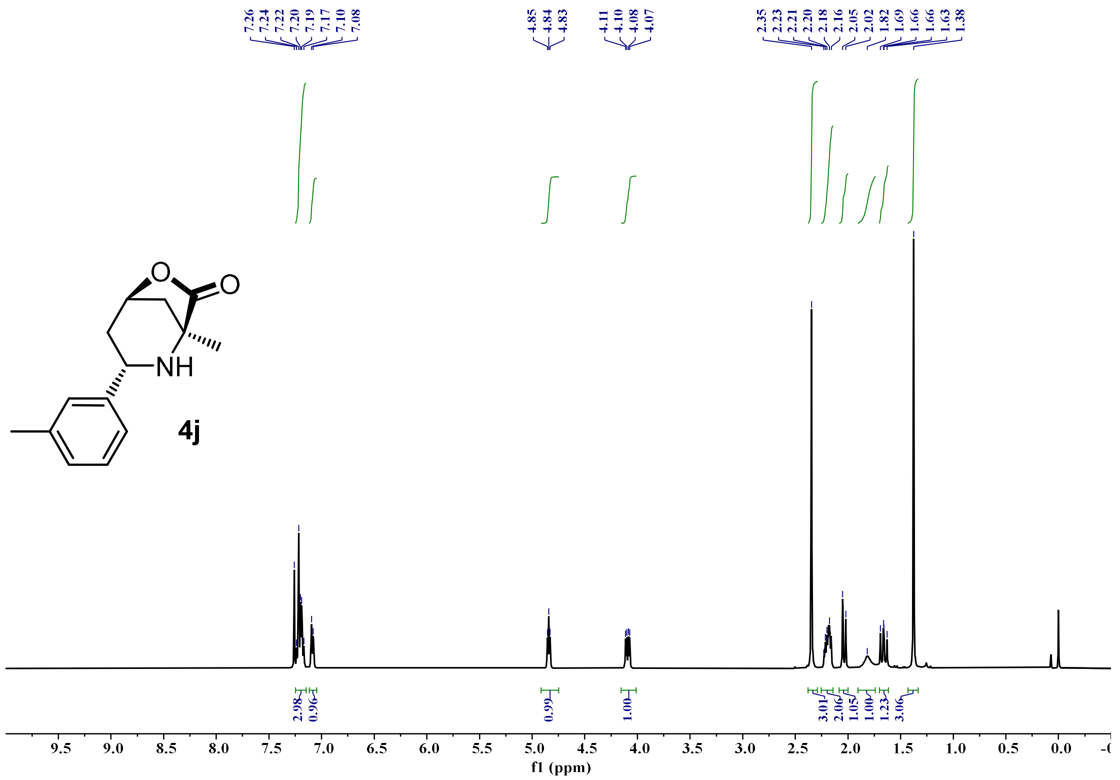

Supplementary Figure 30. ^{1}H NMR spectrum (400 MHz, $CDCl_3$) of **4j**

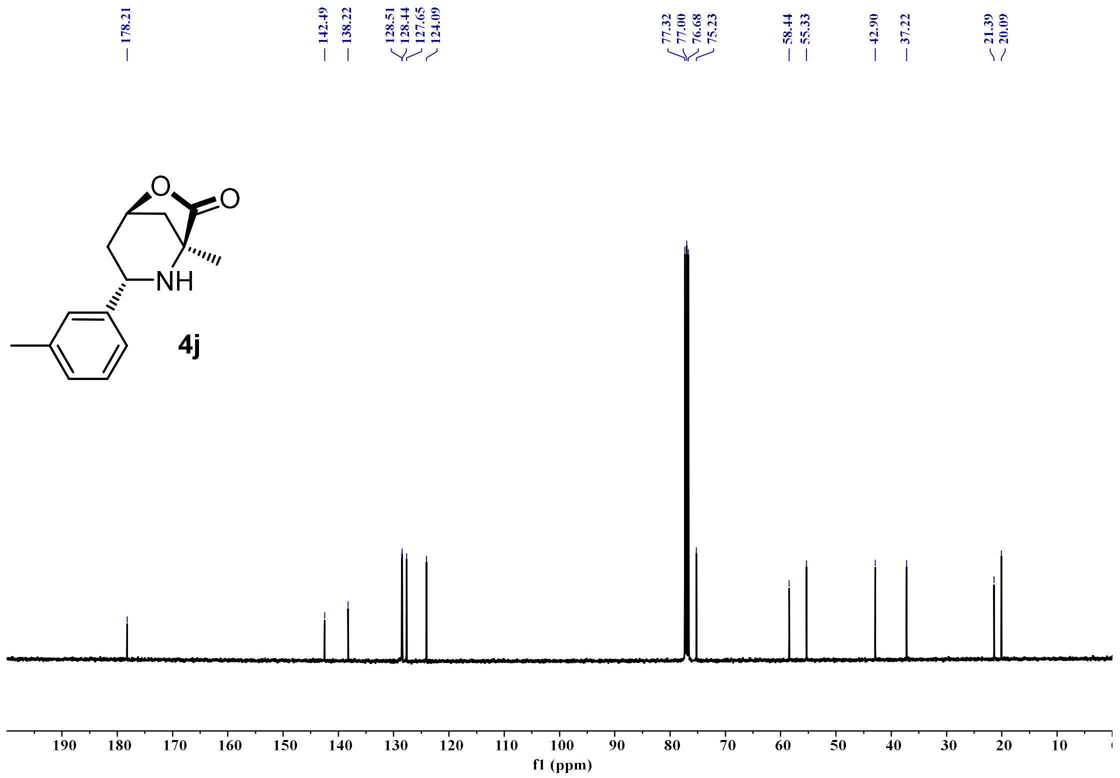

Supplementary Figure 31. ^{13}C NMR spectrum (100 MHz, $CDCl_3$) of **4j**

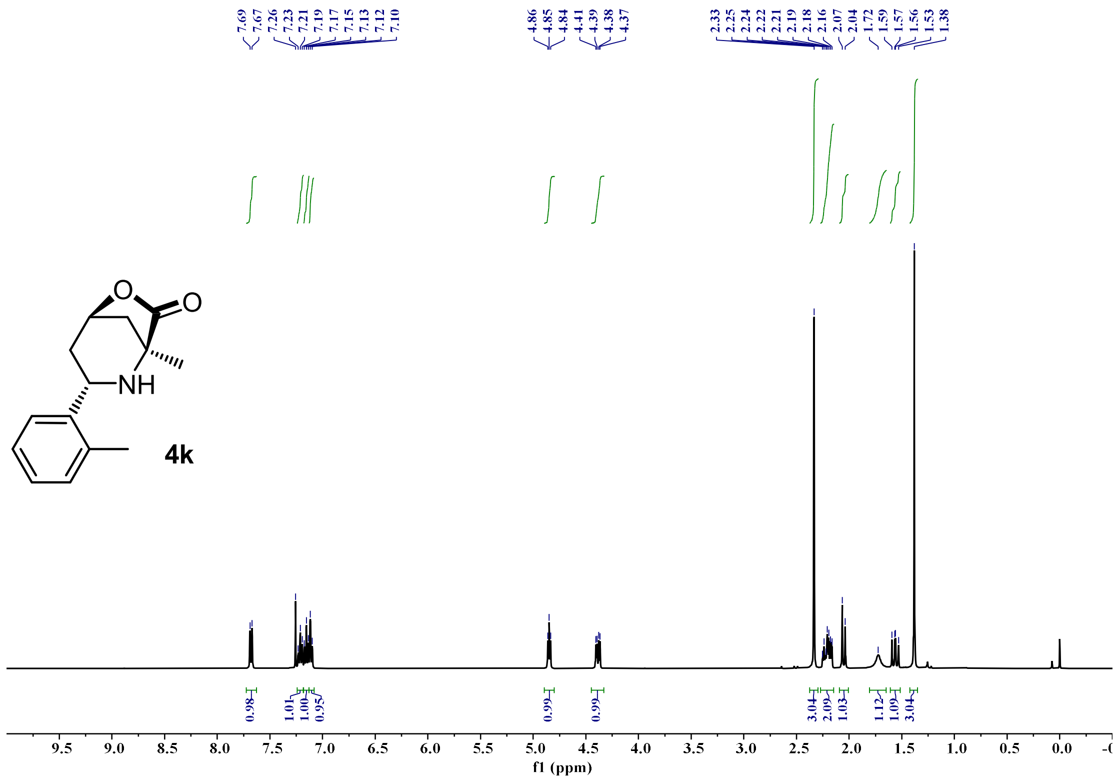

Supplementary Figure 32. ^{1}H NMR spectrum (400 MHz, $CDCl_3$) of **4k**

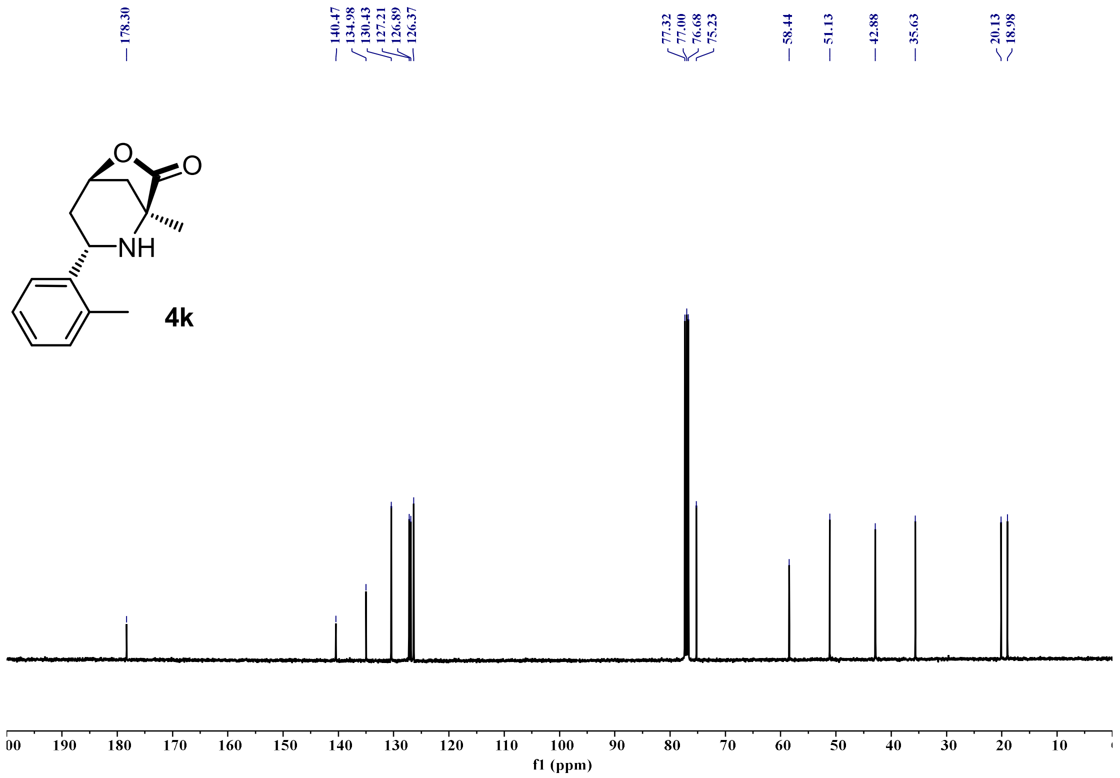

Supplementary Figure 33. ^{13}C NMR spectrum (100 MHz, $CDCl_3$) of **4k**

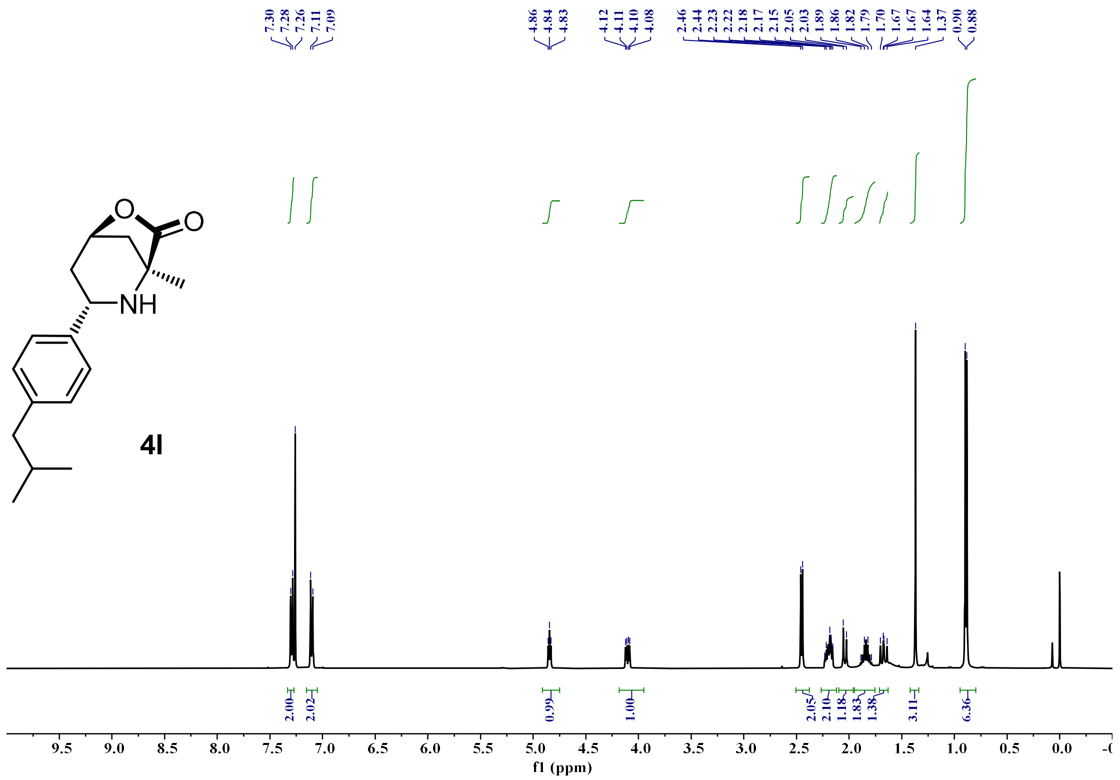

Supplementary Figure 34. ^{1}H NMR spectrum (400 MHz, $CDCl_3$) of **4l**

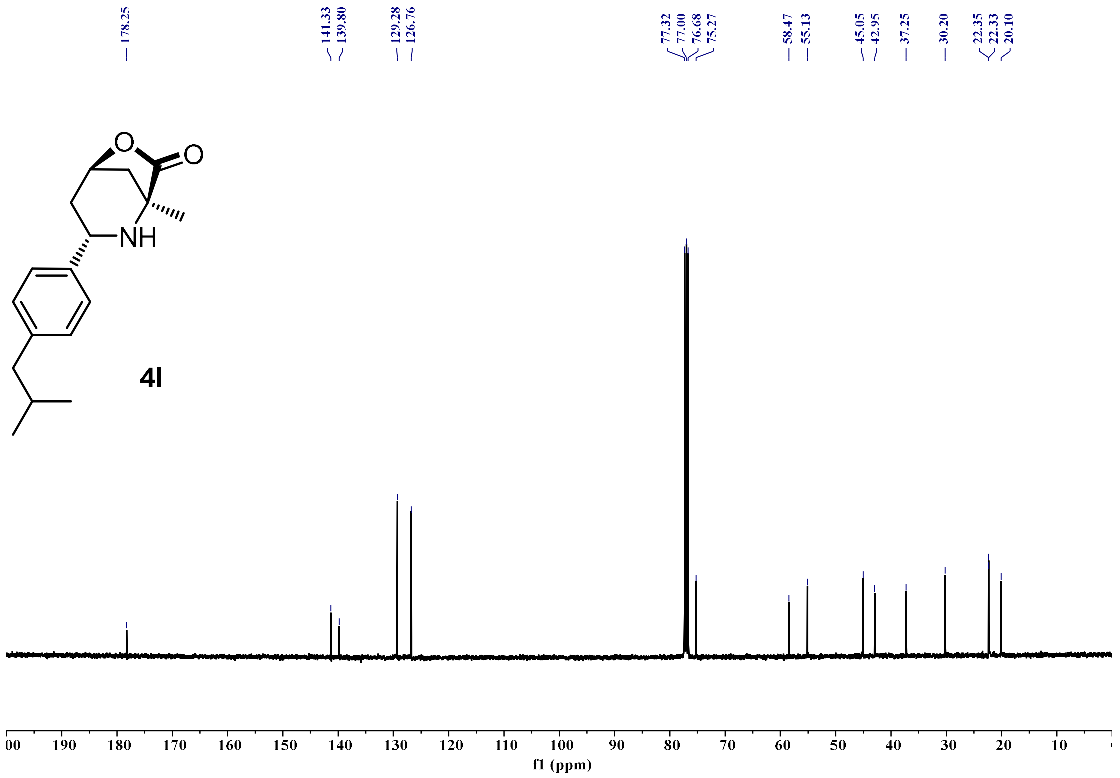

Supplementary Figure 35. ^{13}C NMR spectrum (100 MHz, $CDCl_3$) of **4l**

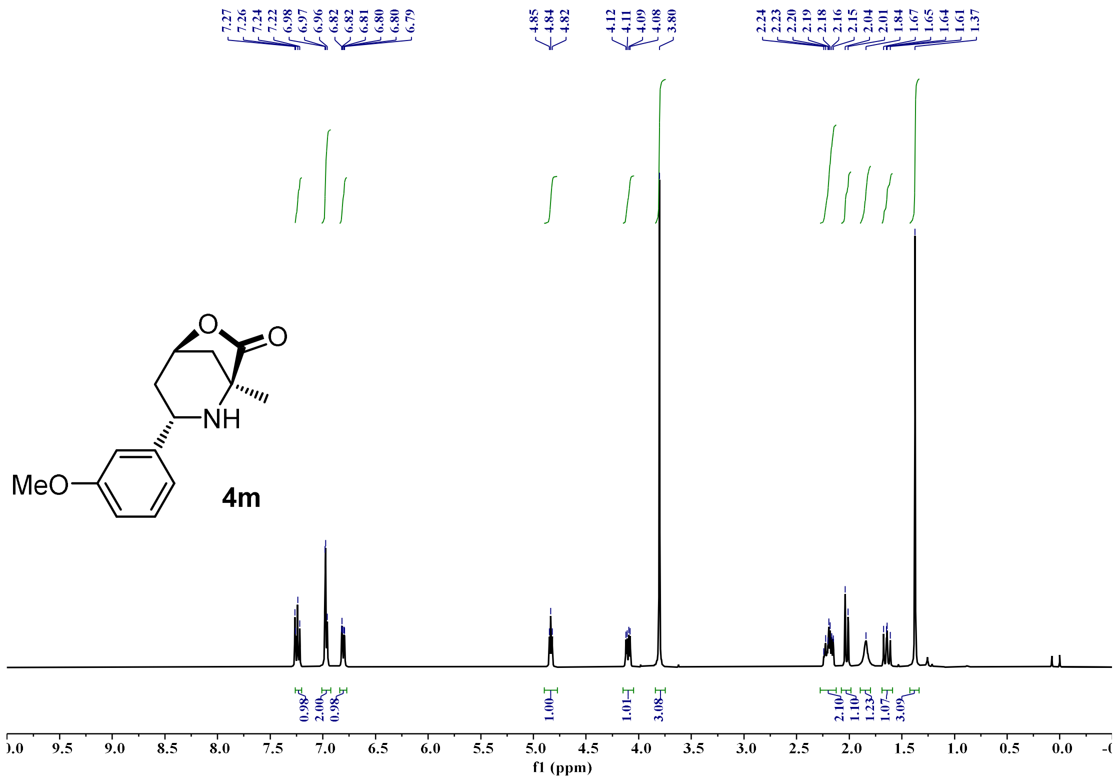

Supplementary Figure 36. ^{1}H NMR spectrum (400 MHz, $CDCl_3$) of **4m**

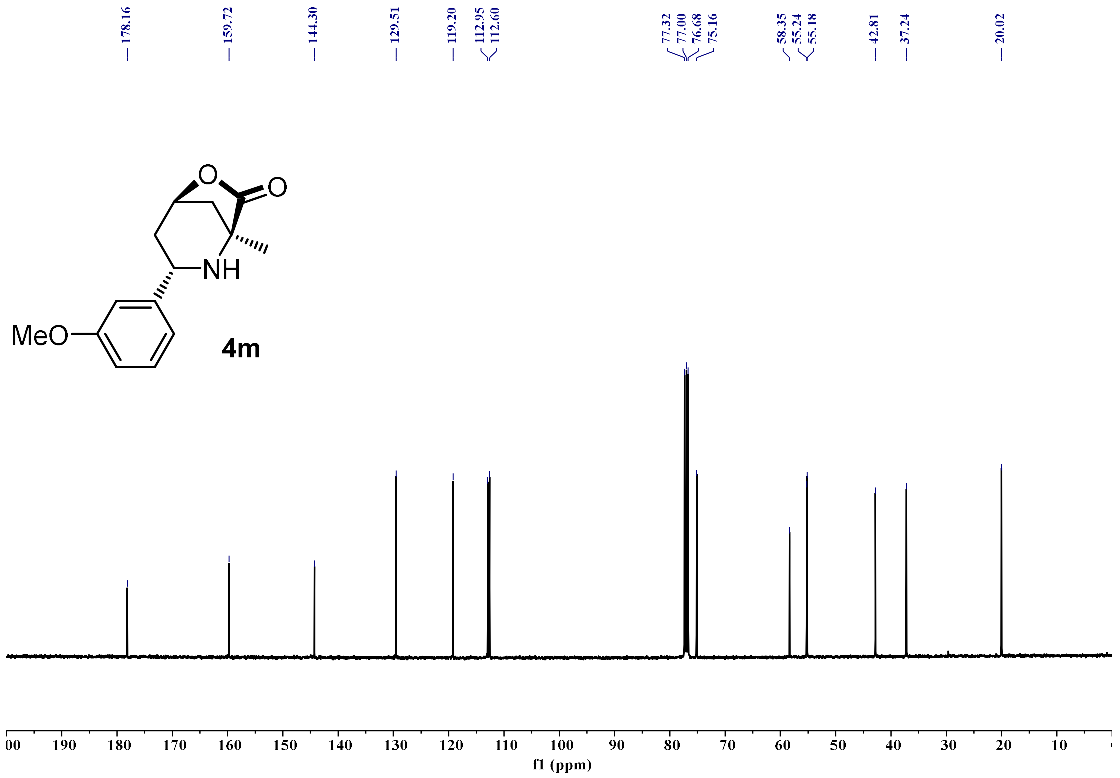

Supplementary Figure 37. ^{13}C NMR spectrum (100 MHz, $CDCl_3$) of **4m**

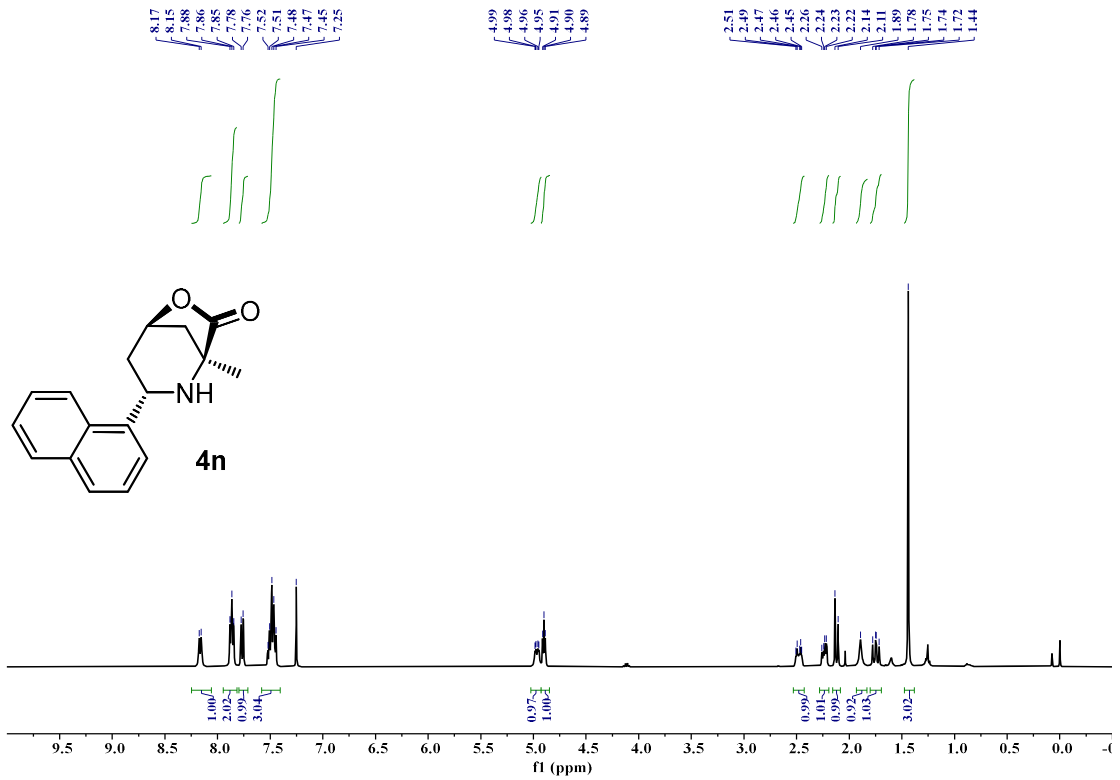

Supplementary Figure 38. ^{1}H NMR spectrum (400 MHz, $CDCl_3$) of **4n**

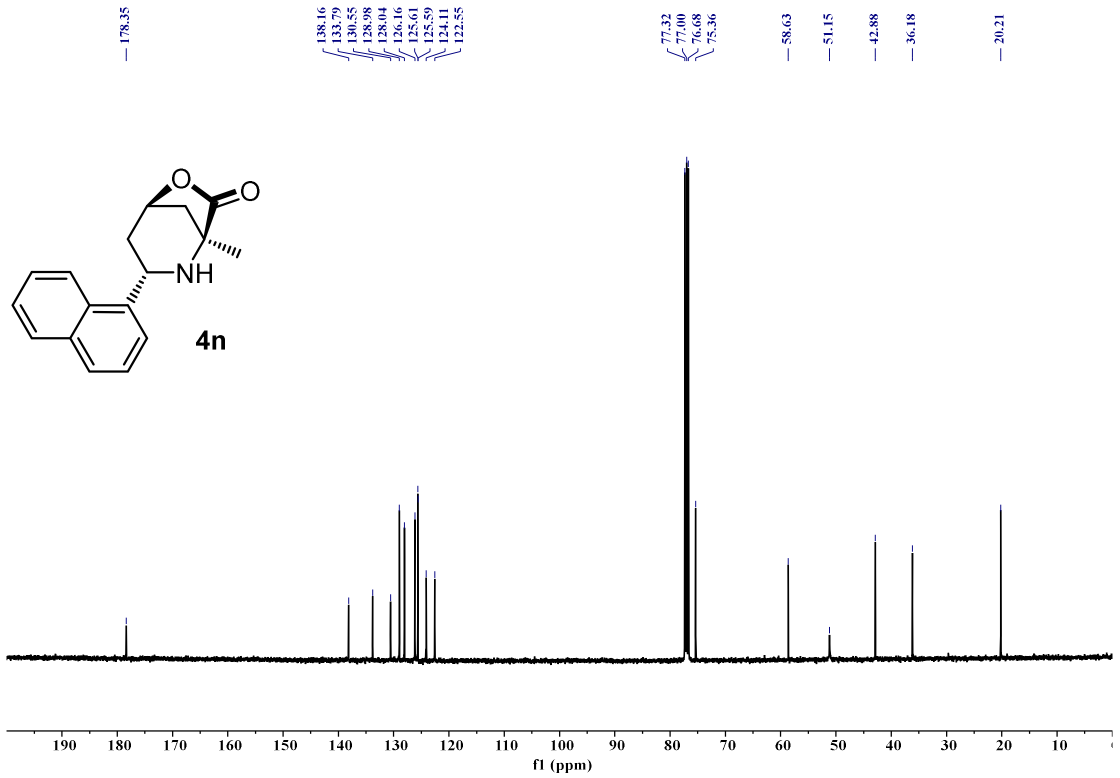

Supplementary Figure 39. ^{13}C NMR spectrum (100 MHz, $CDCl_3$) of **4n**

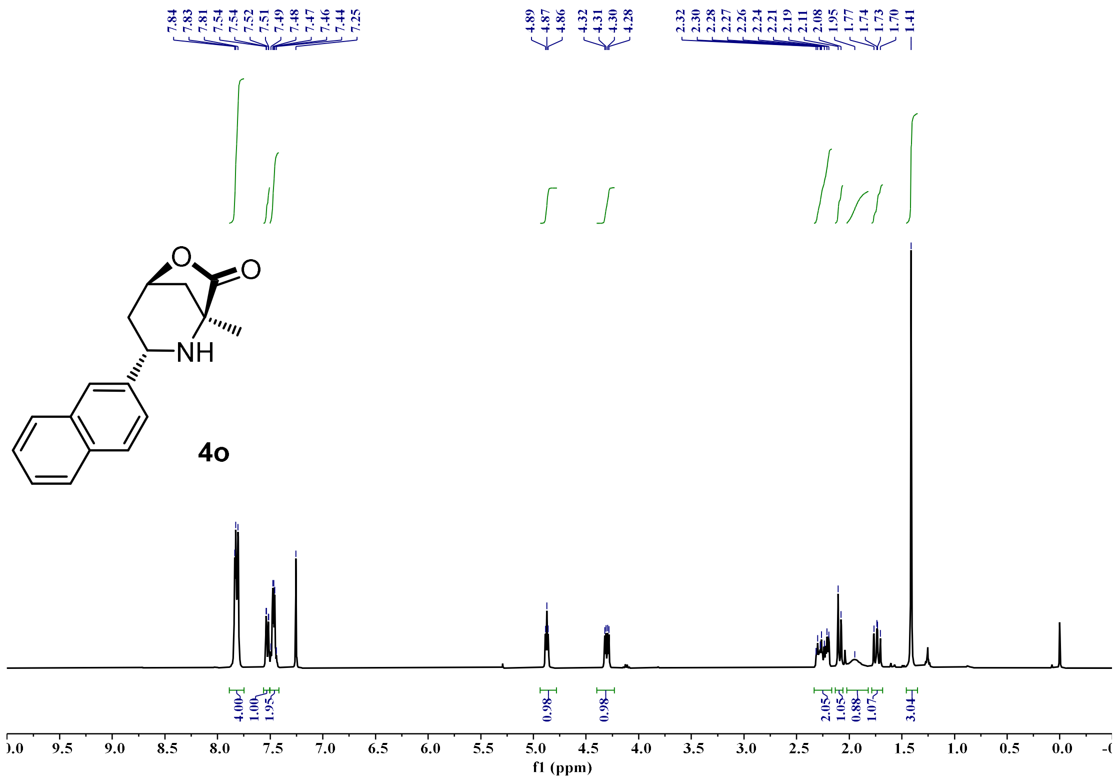

Supplementary Figure 40. ^{1}H NMR spectrum (400 MHz, $CDCl_3$) of **4o**

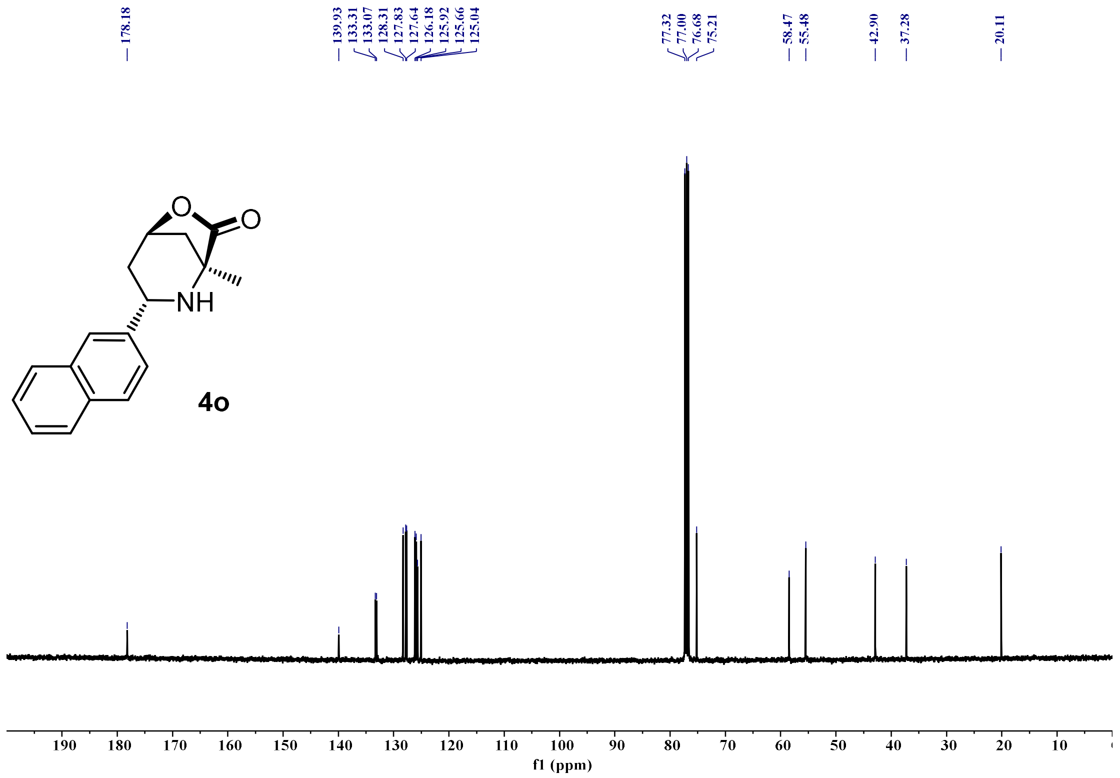

Supplementary Figure 41. ^{13}C NMR spectrum (100 MHz, $CDCl_3$) of **4o**

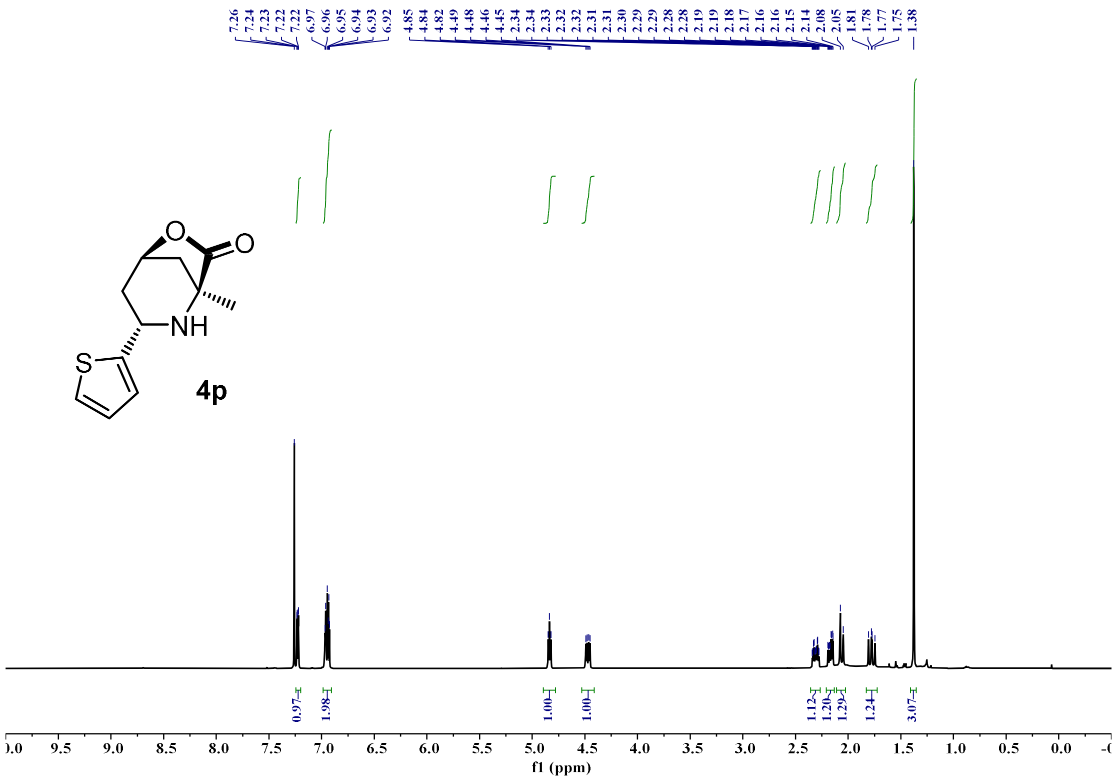

Supplementary Figure 42. ^{1}H NMR spectrum (400 MHz, $CDCl_3$) of **4p**

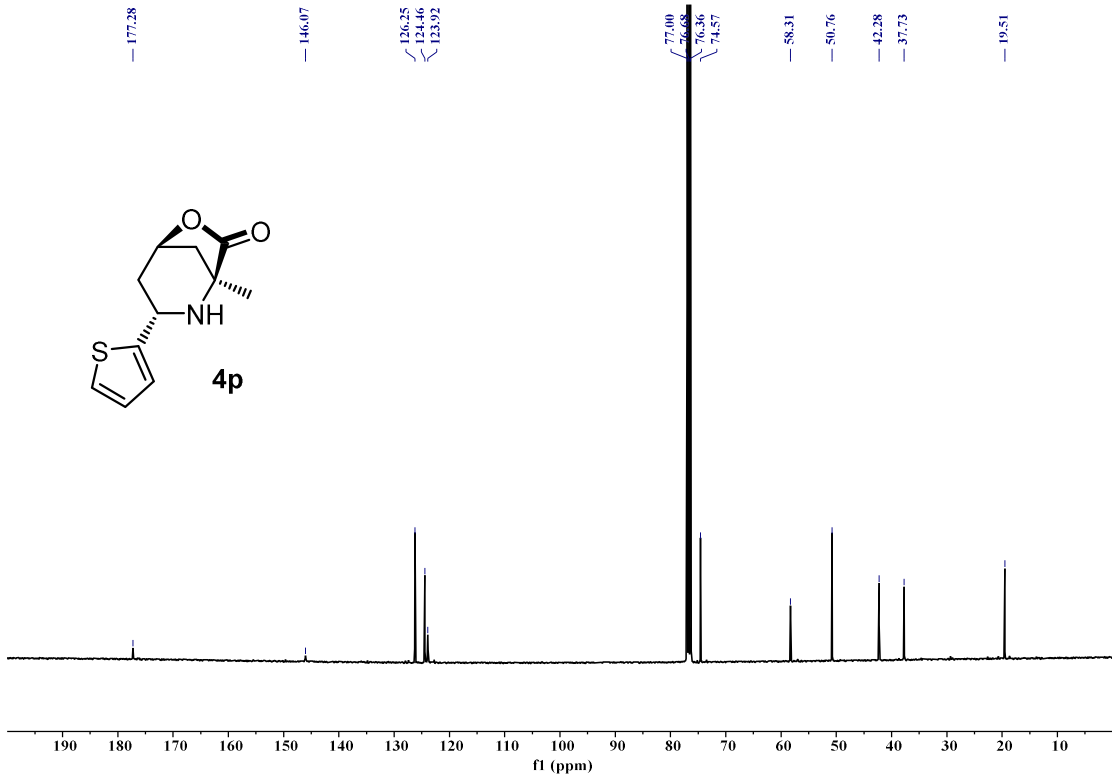

Supplementary Figure 43. ^{13}C NMR spectrum (100 MHz, $CDCl_3$) of **4p**

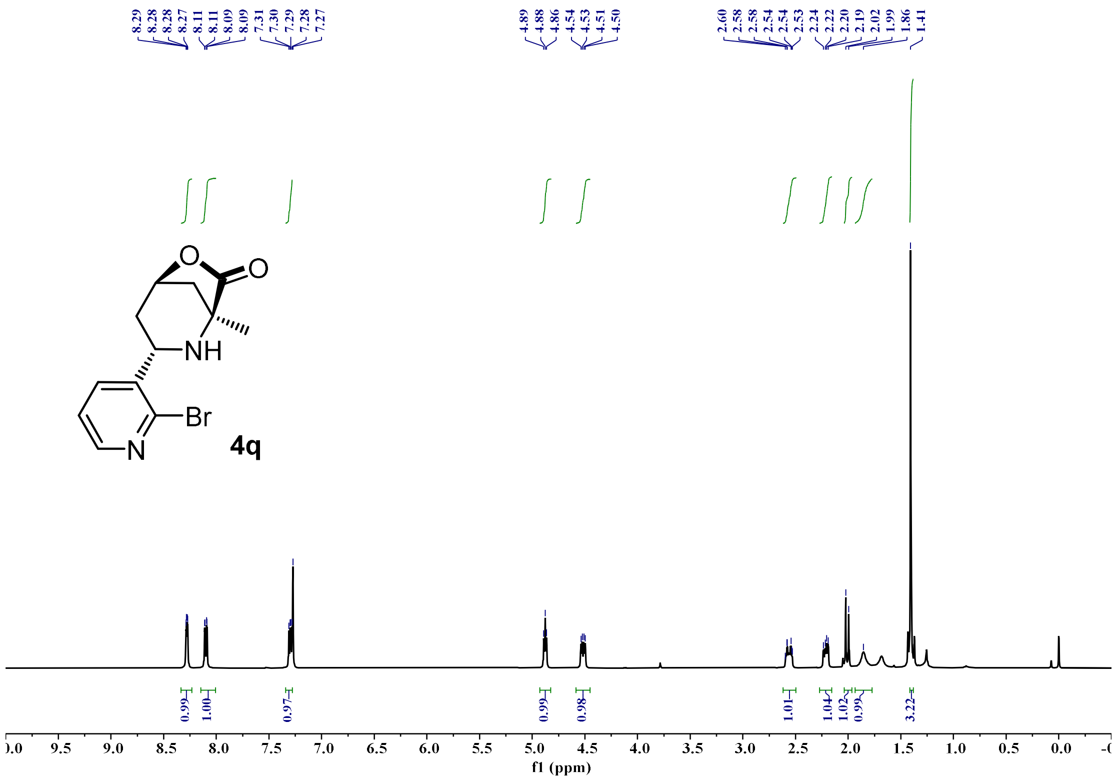

Supplementary Figure 44. ^{1}H NMR spectrum (400 MHz, $CDCl_3$) of **4q**

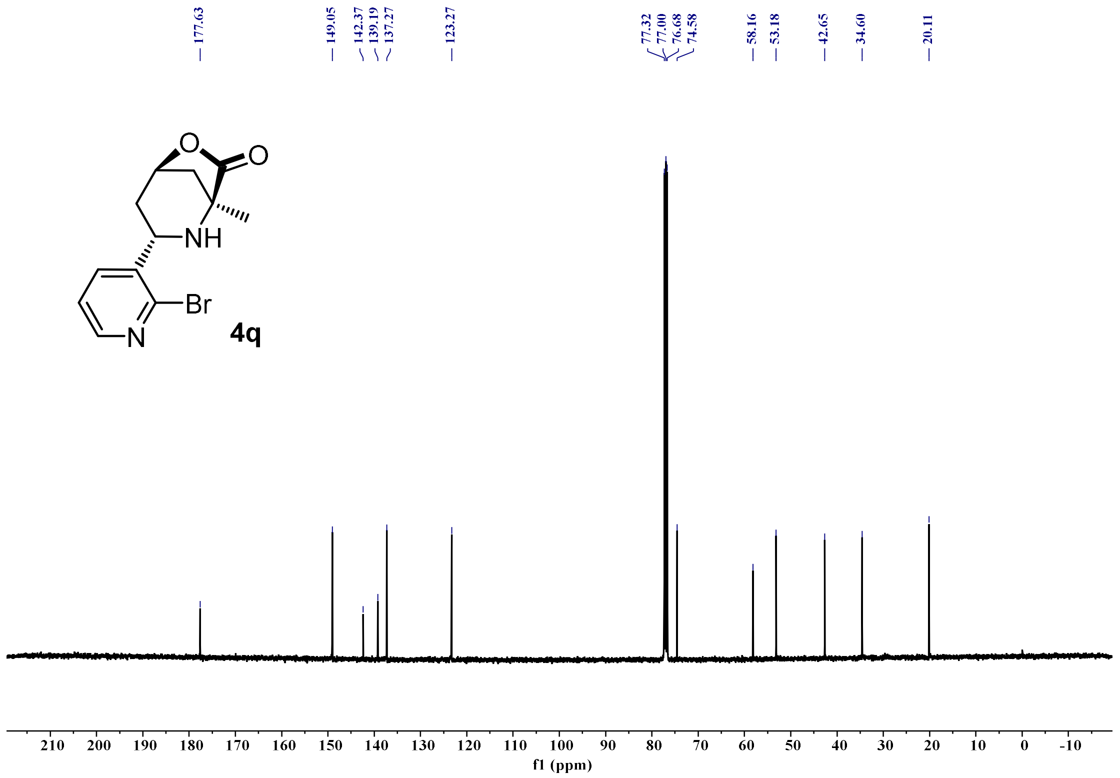

Supplementary Figure 45. ^{13}C NMR spectrum (100 MHz, $CDCl_3$) of **4q**

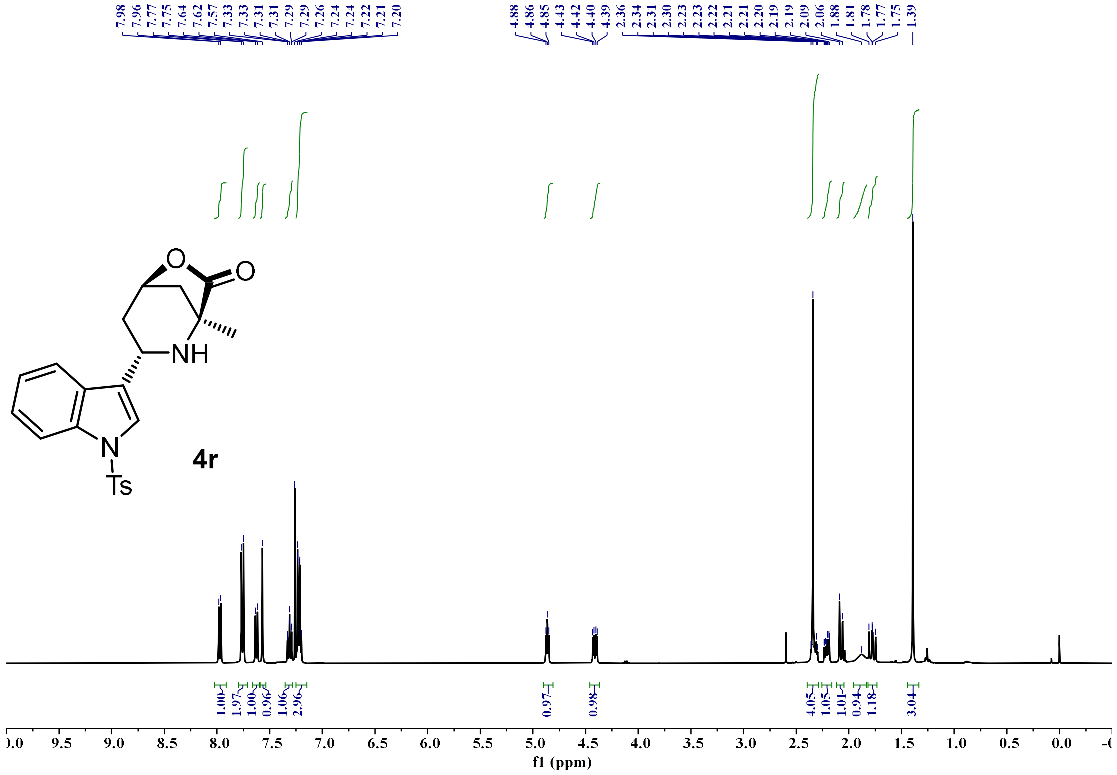

Supplementary Figure 46. ^{1}H NMR spectrum (400 MHz, $CDCl_3$) of **4r**

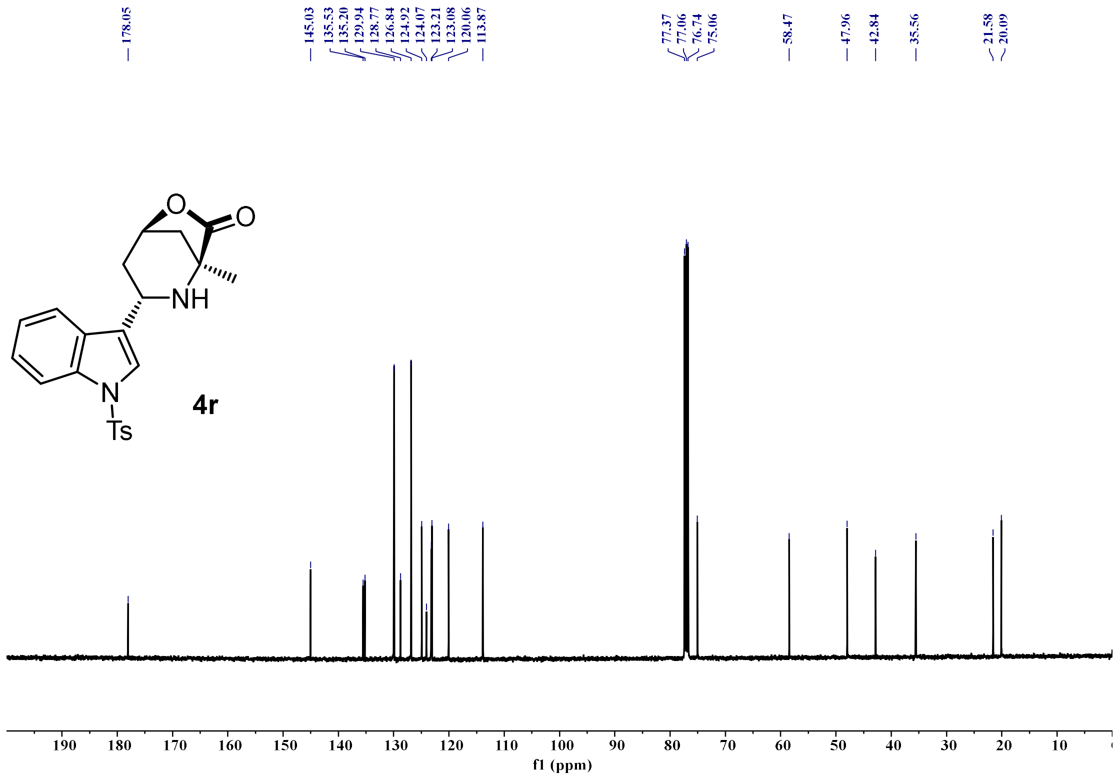

Supplementary Figure 47. ^{13}C NMR spectrum (100 MHz, $CDCl_3$) of **4r**

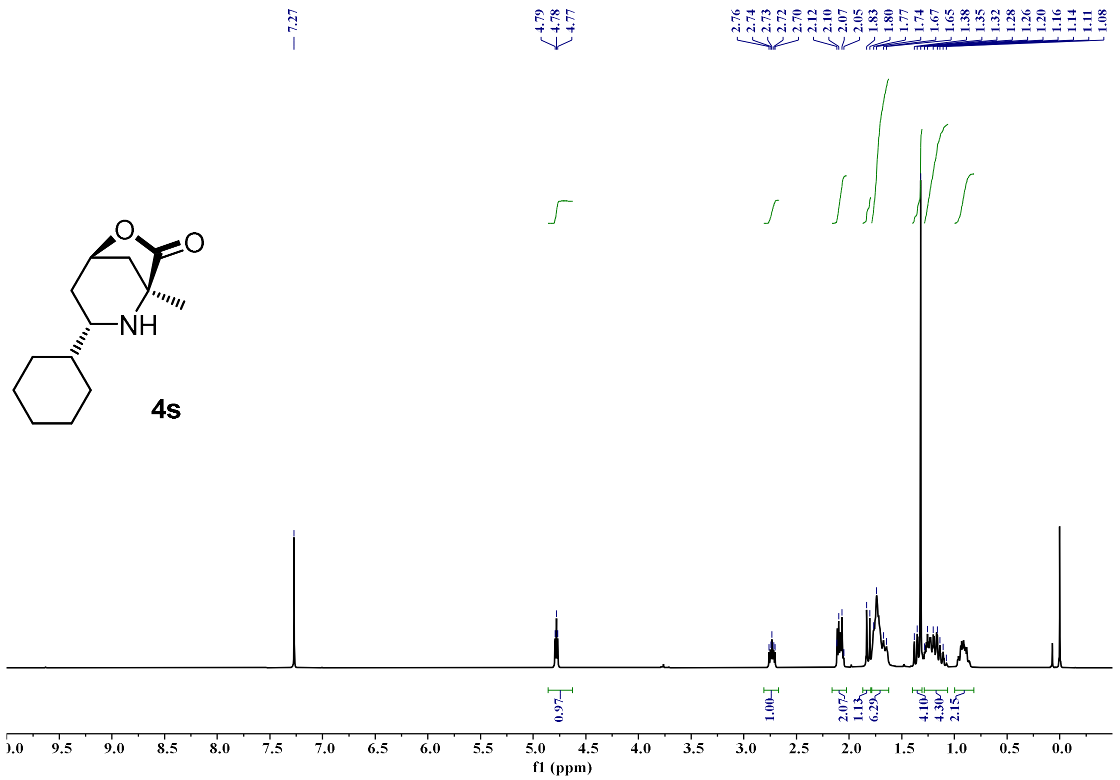

Supplementary Figure 48. ^{1}H NMR spectrum (400 MHz, $CDCl_3$) of **4s**

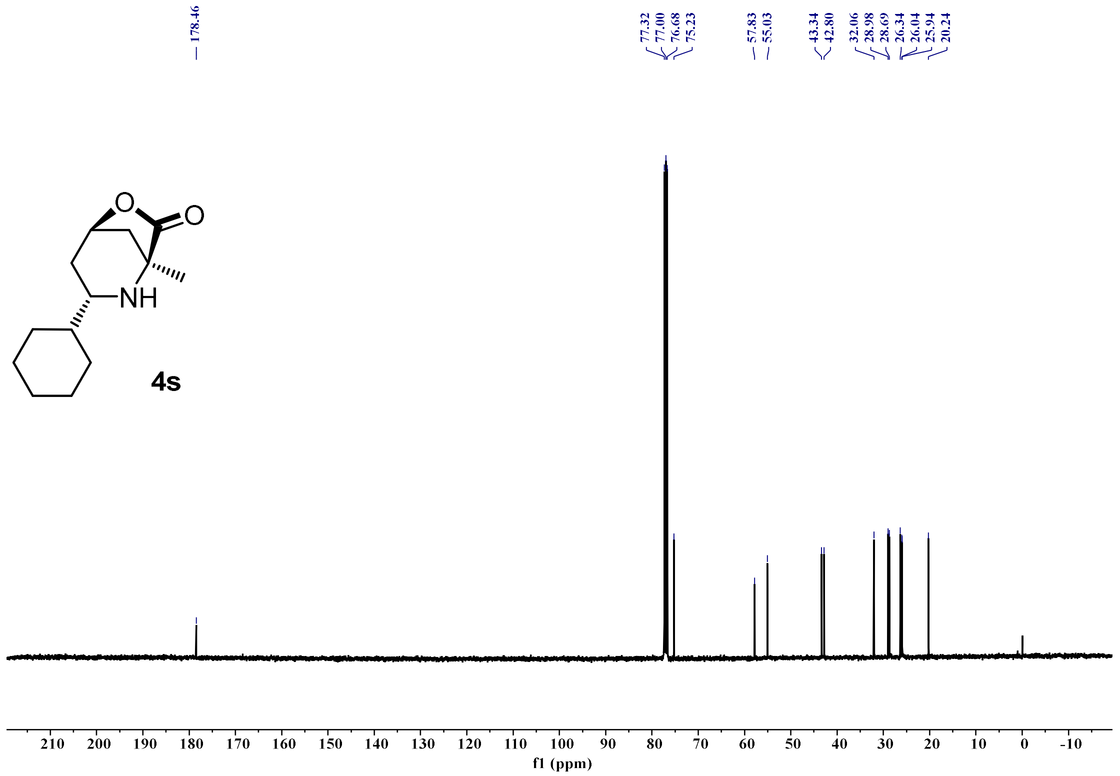

Supplementary Figure 49. ^{13}C NMR spectrum (100 MHz, $CDCl_3$) of **4s**

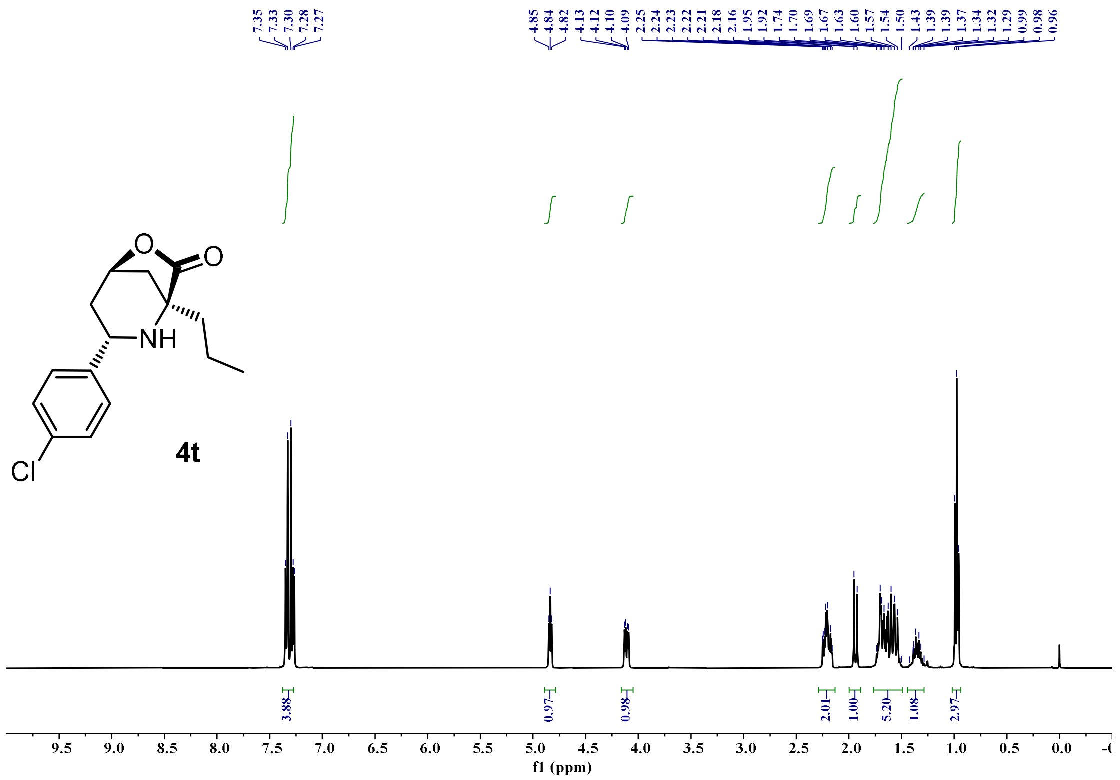

Supplementary Figure 50. ^{1}H NMR spectrum (400 MHz, $CDCl_3$) of **4t**

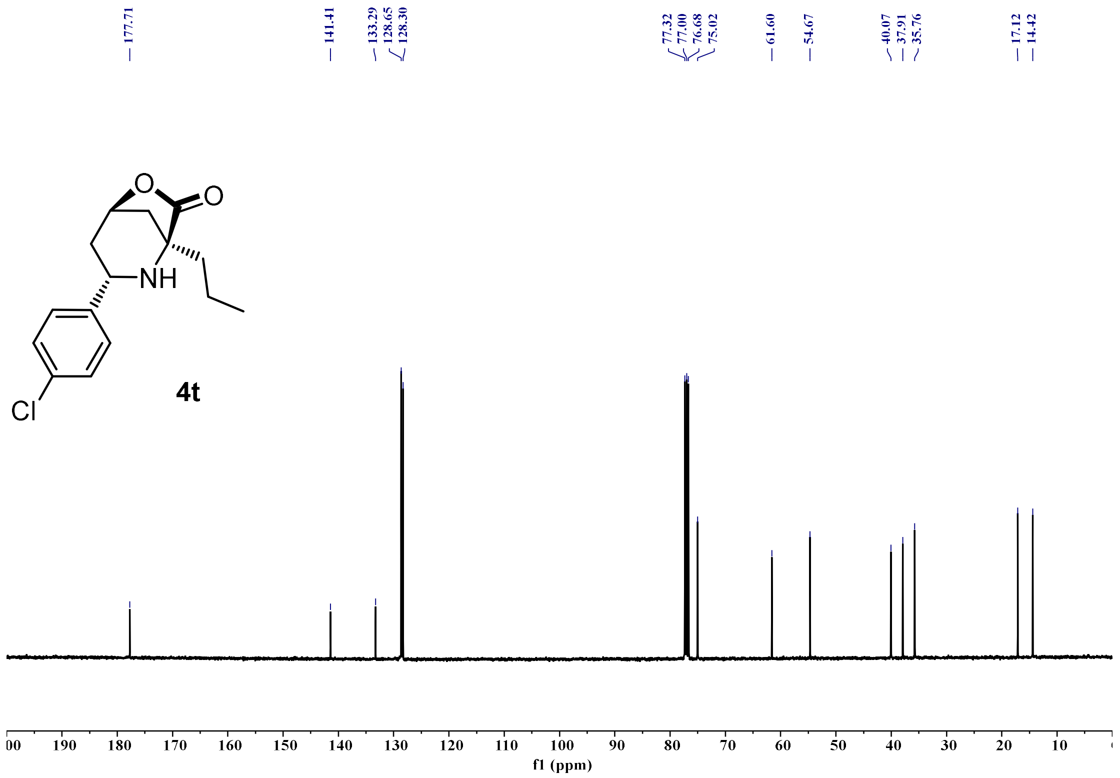

Supplementary Figure 51. ^{13}C NMR spectrum (100 MHz, $CDCl_3$) of **4t**

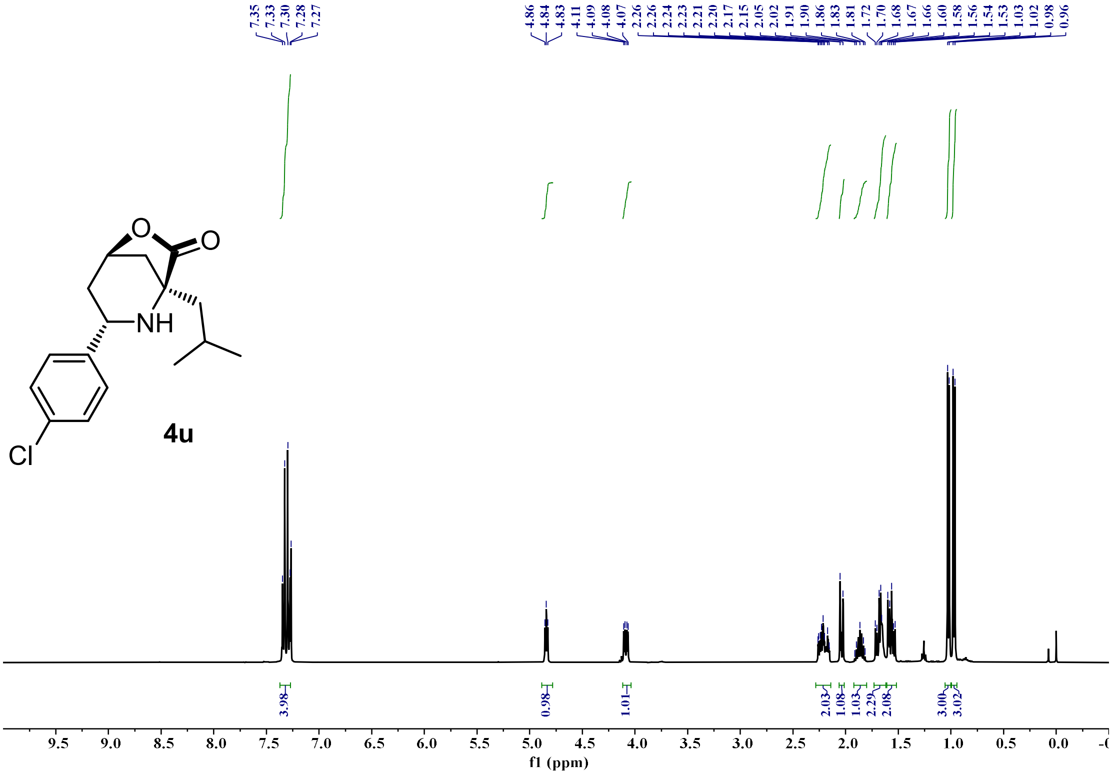

Supplementary Figure 52. ^{1}H NMR spectrum (400 MHz, $CDCl_3$) of **4u**

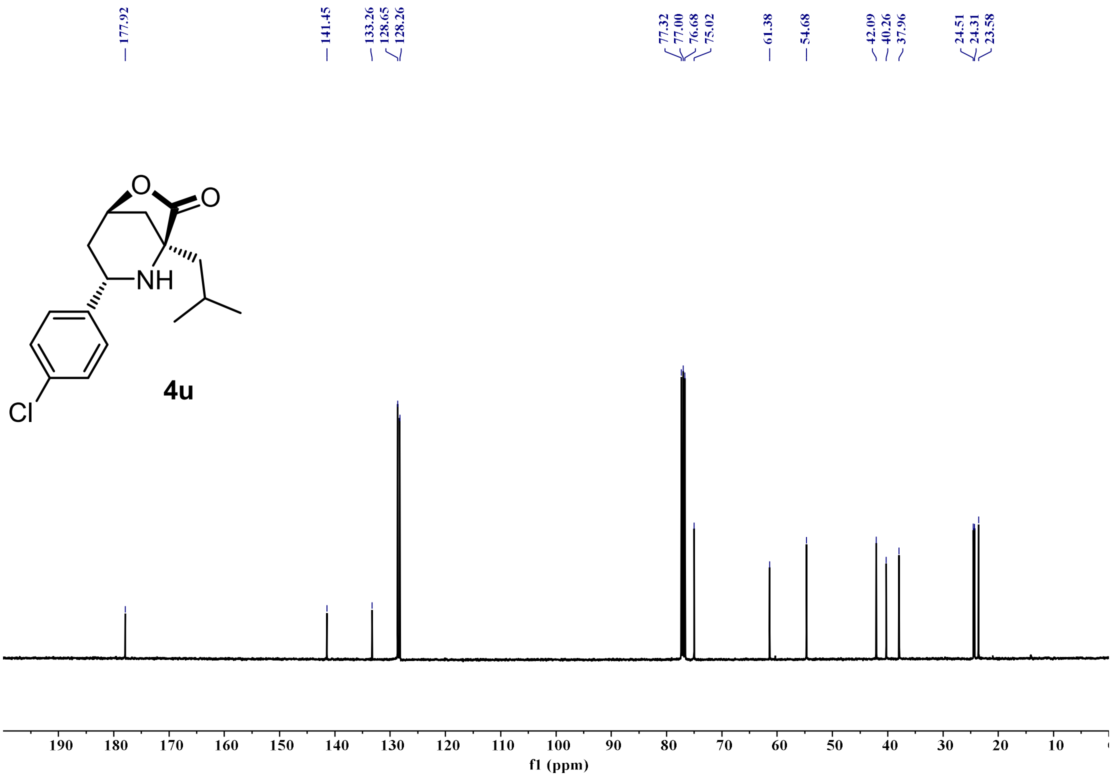

Supplementary Figure 53. ^{13}C NMR spectrum (100 MHz, $CDCl_3$) of **4u**

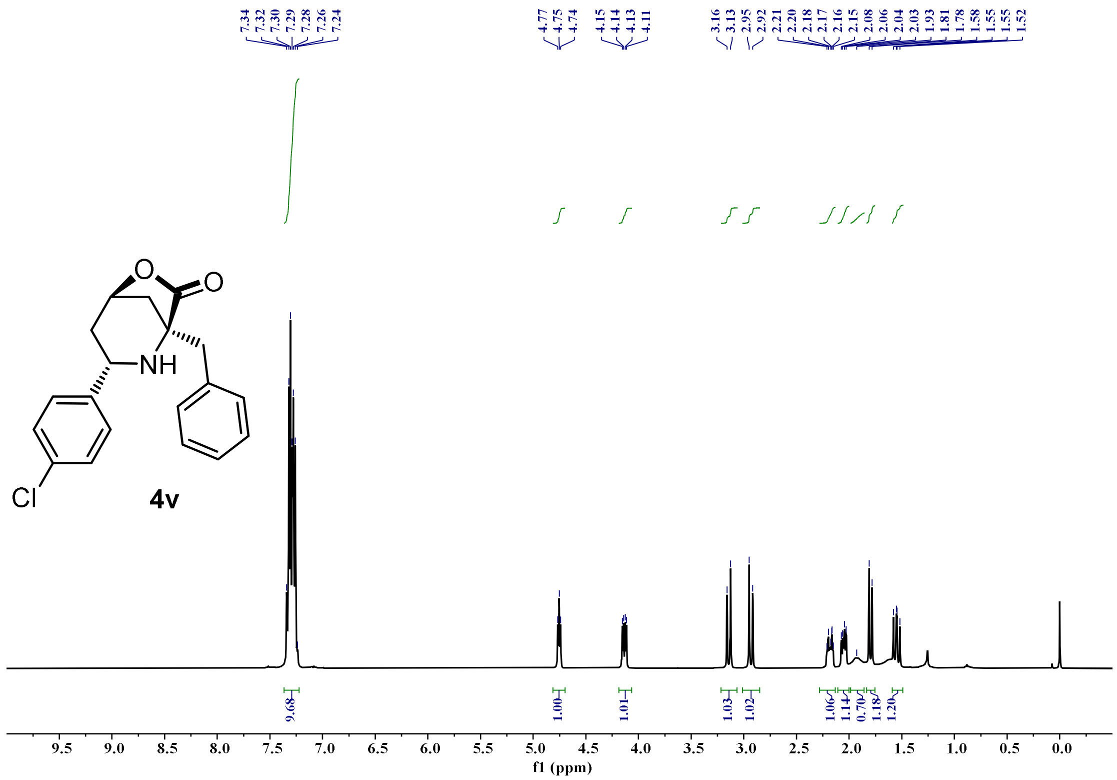

Supplementary Figure 54. ^{1}H NMR spectrum (400 MHz, $CDCl_3$) of **4v**

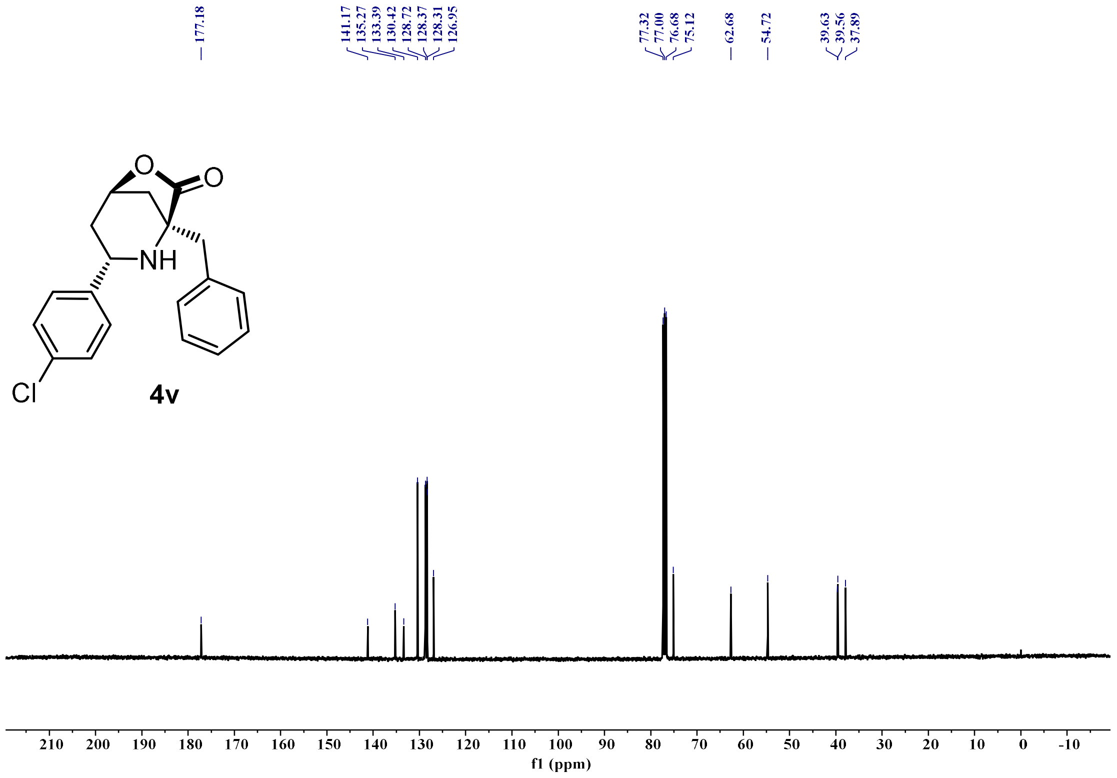

Supplementary Figure 55. ^{13}C NMR spectrum (100 MHz, $CDCl_3$) of **4v**

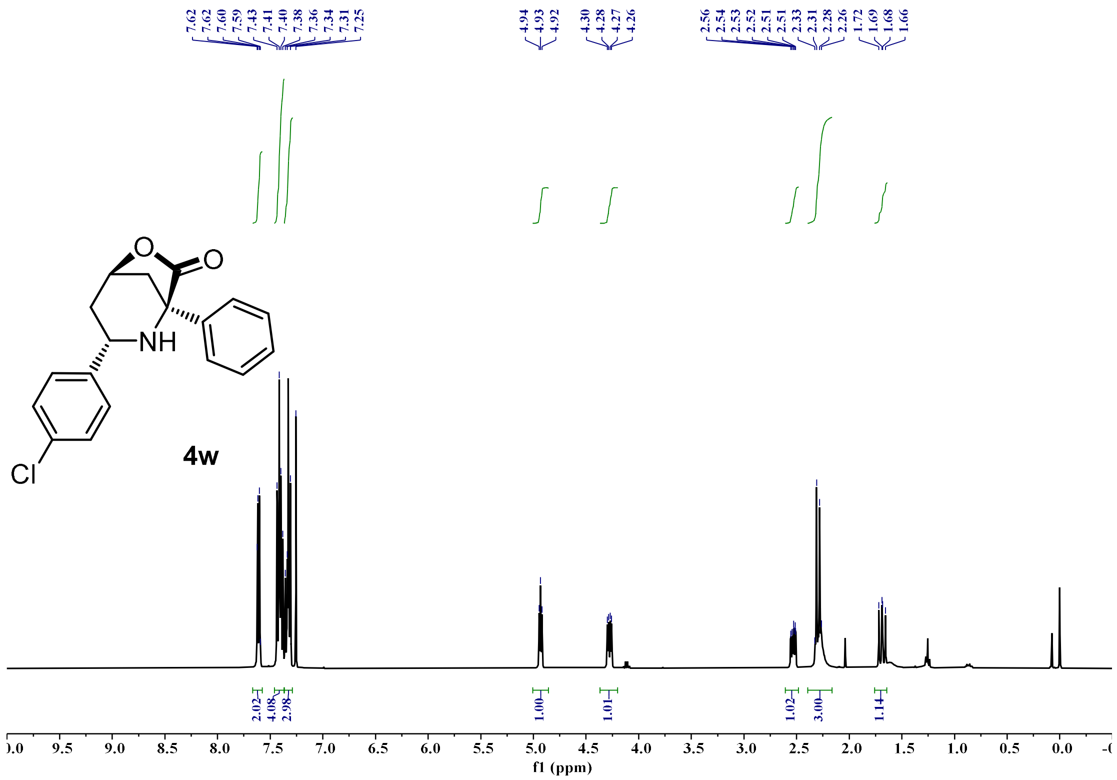

Supplementary Figure 56. ^{1}H NMR spectrum (400 MHz, $CDCl_3$) of **4w**

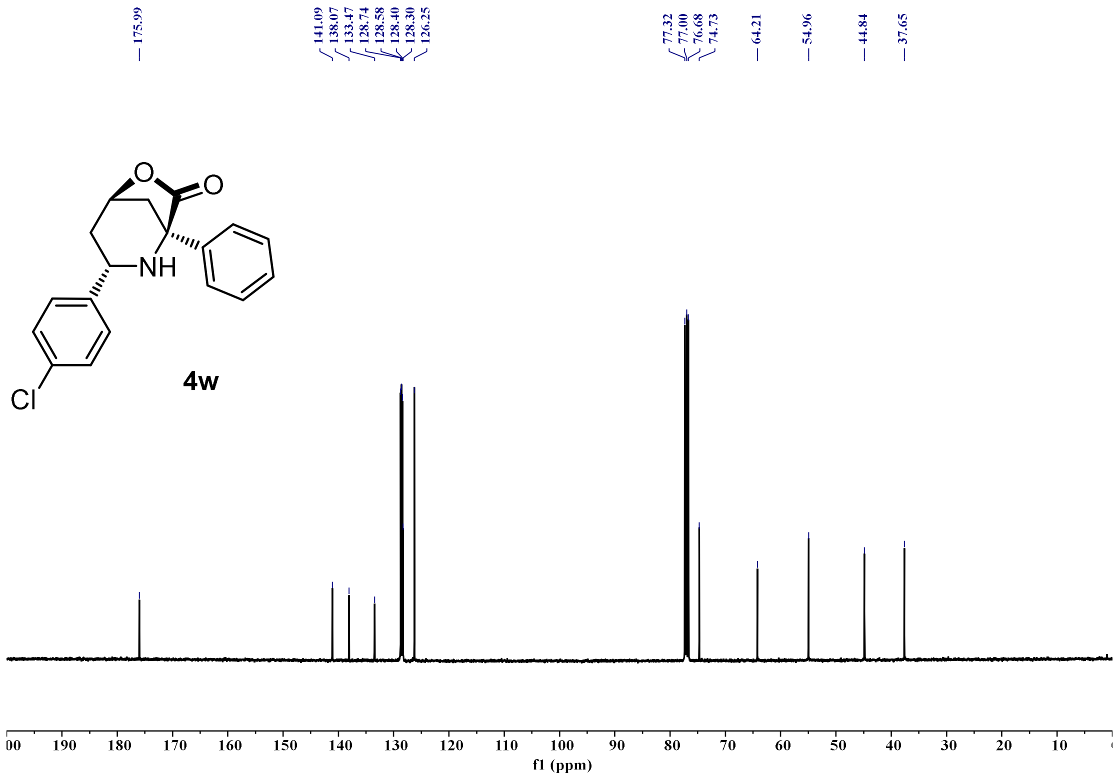

Supplementary Figure 57. ^{13}C NMR spectrum (100 MHz, $CDCl_3$) of **4w**

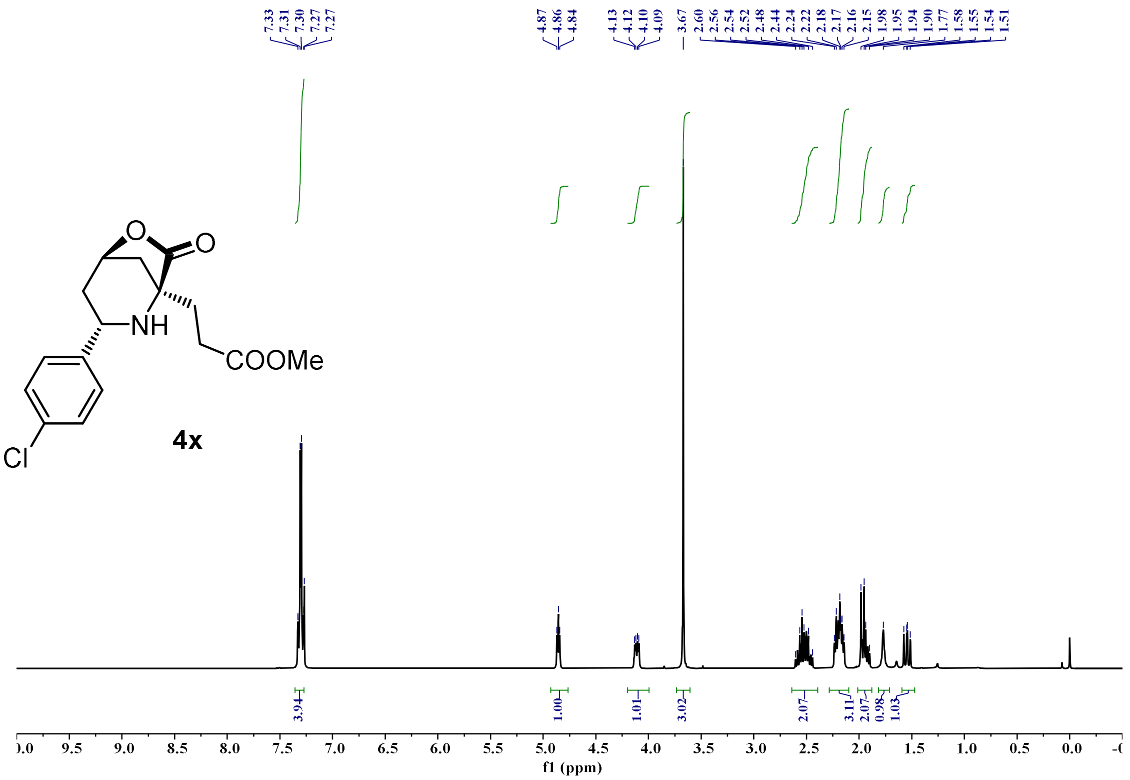

Supplementary Figure 58. ^{1}H NMR spectrum (400 MHz, $CDCl_3$) of **4x**

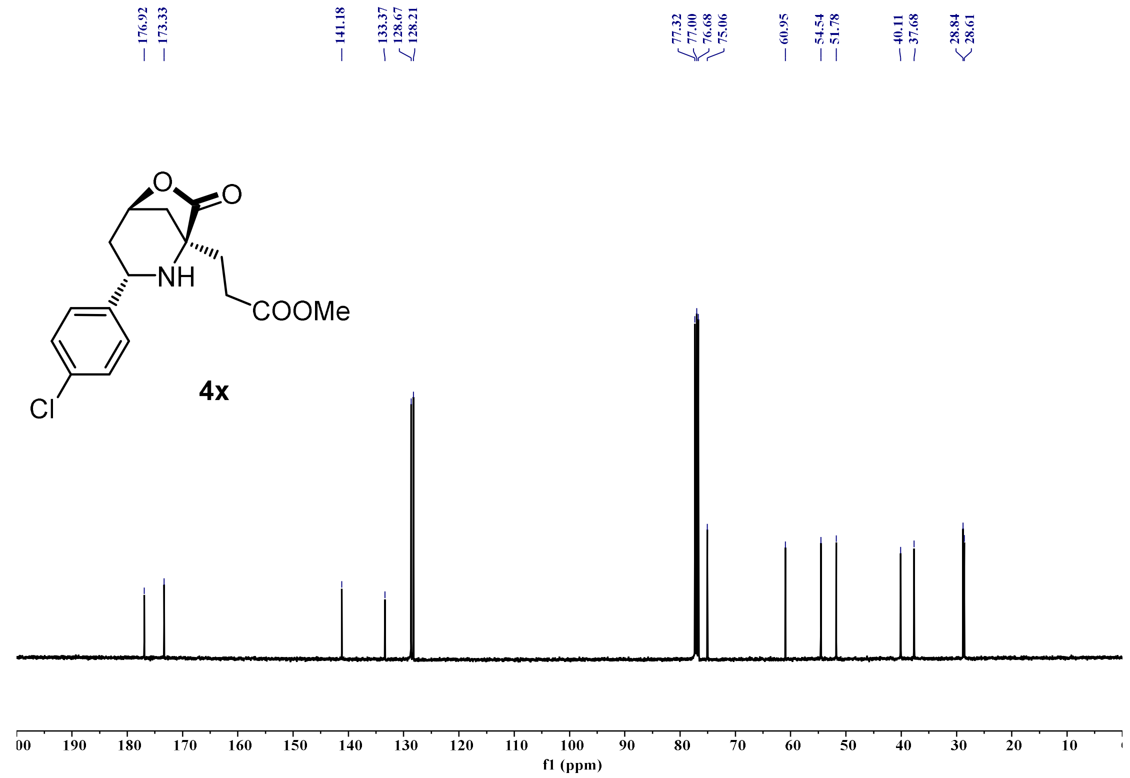

Supplementary Figure 59. ^{13}C NMR spectrum (100 MHz, $CDCl_3$) of **4x**

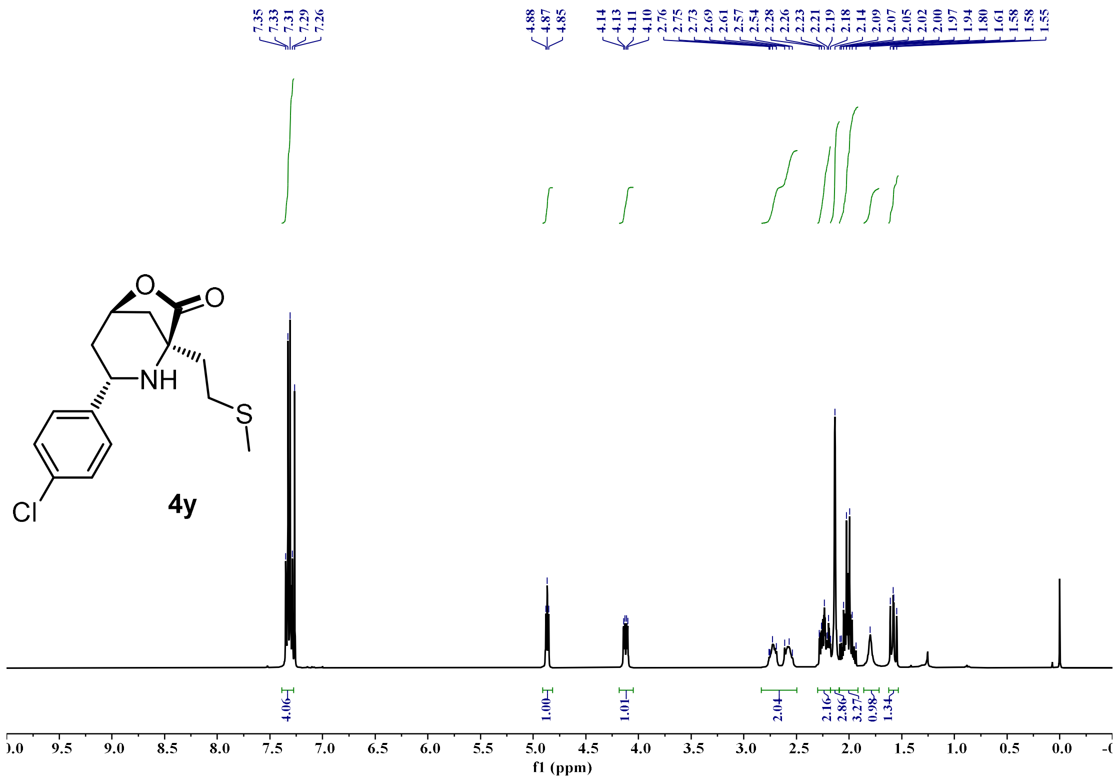

Supplementary Figure 60. ^{1}H NMR spectrum (400 MHz, $CDCl_3$) of **4y**

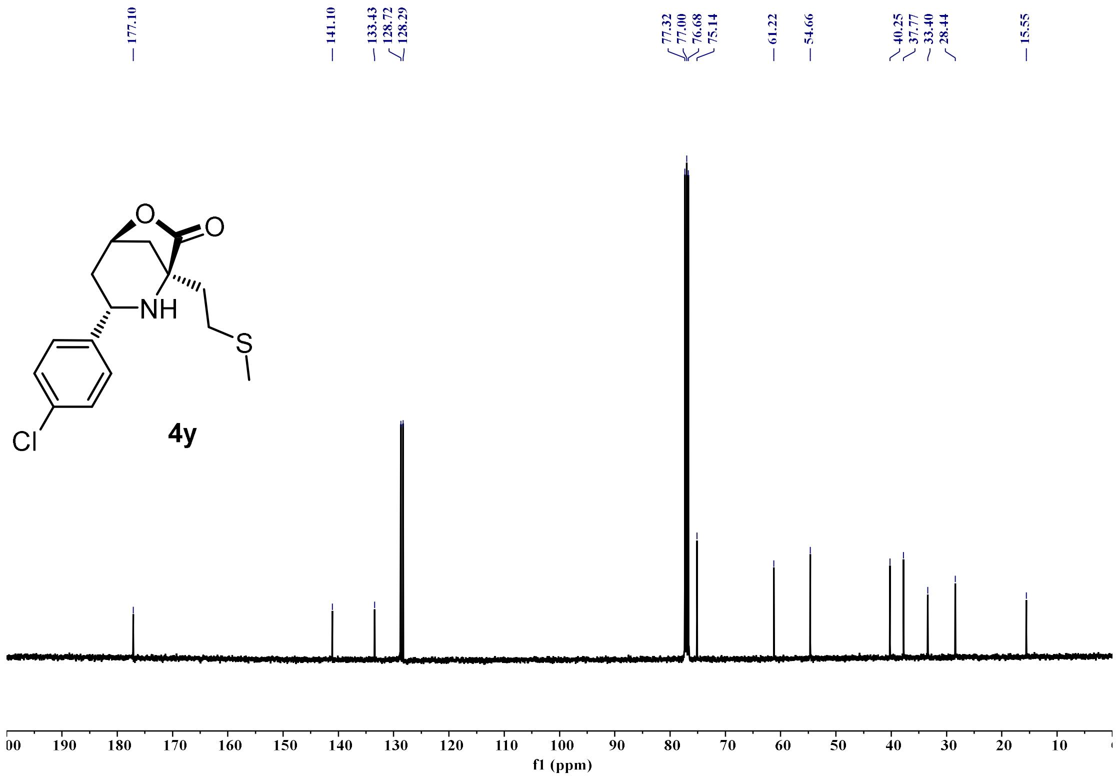

Supplementary Figure 61. ^{13}C NMR spectrum (100 MHz, $CDCl_3$) of **4y**

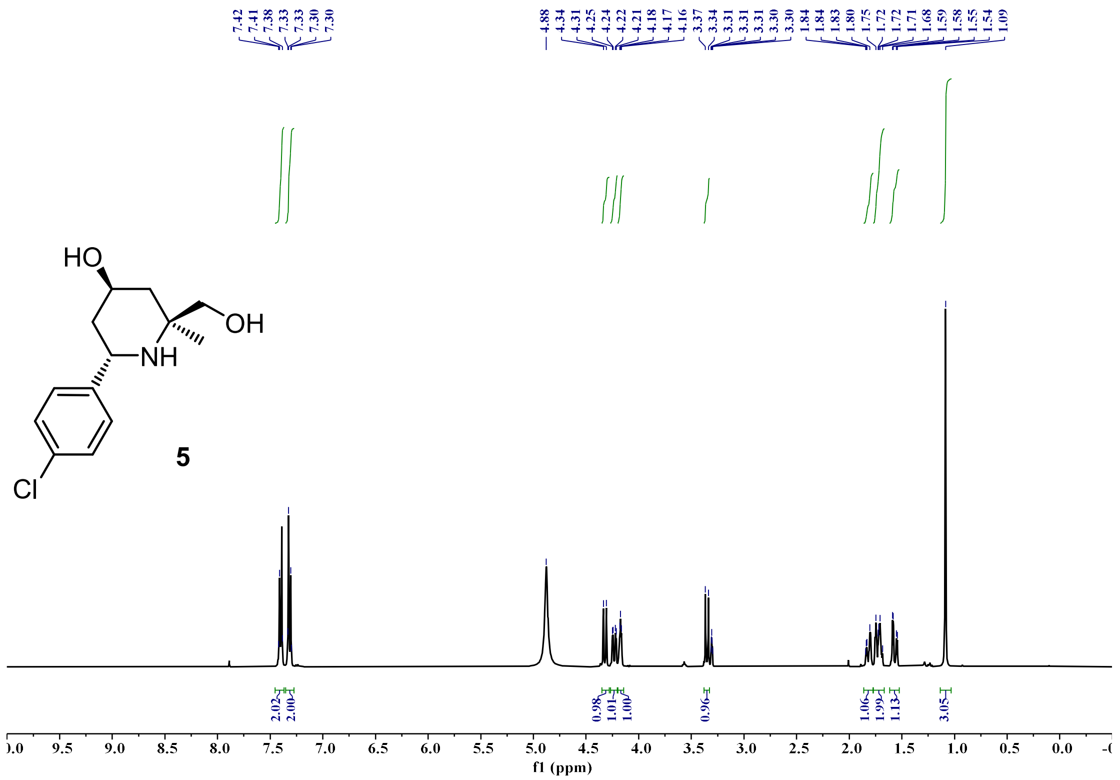

Supplementary Figure 62. ^{1}H NMR spectrum (400 MHz, CD_3OD) of **5**

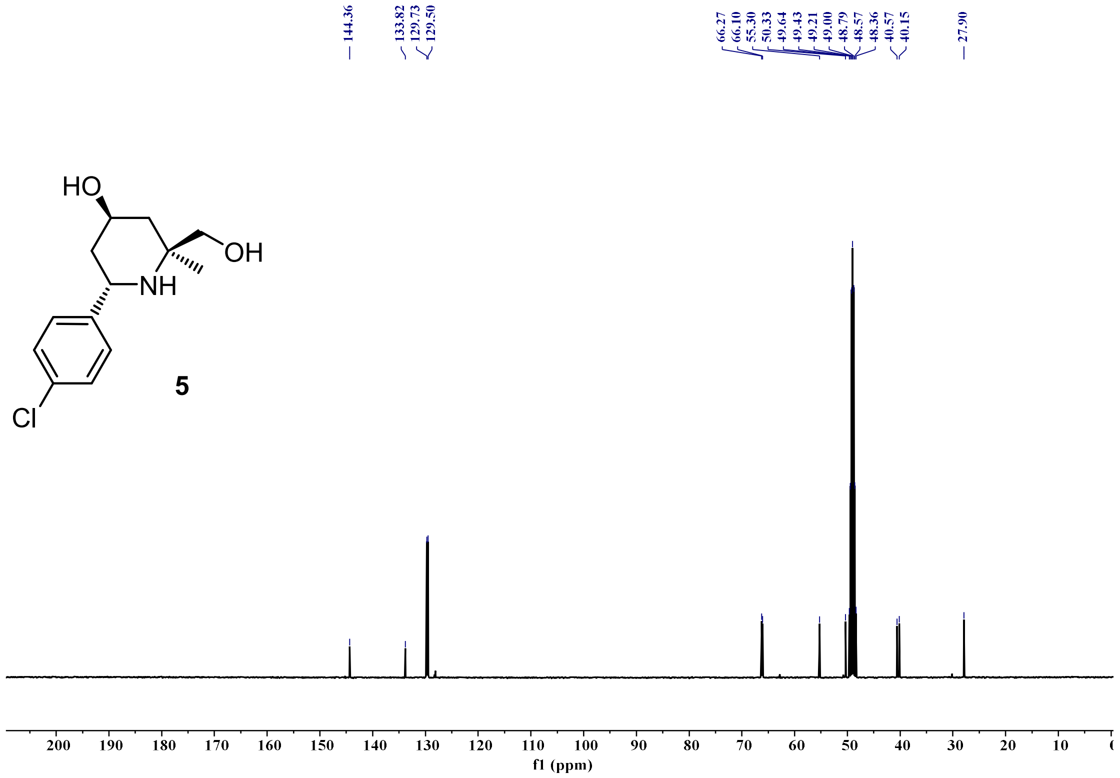

Supplementary Figure 63. ^{13}C NMR spectrum (100 MHz, CD_3OD) of **5**

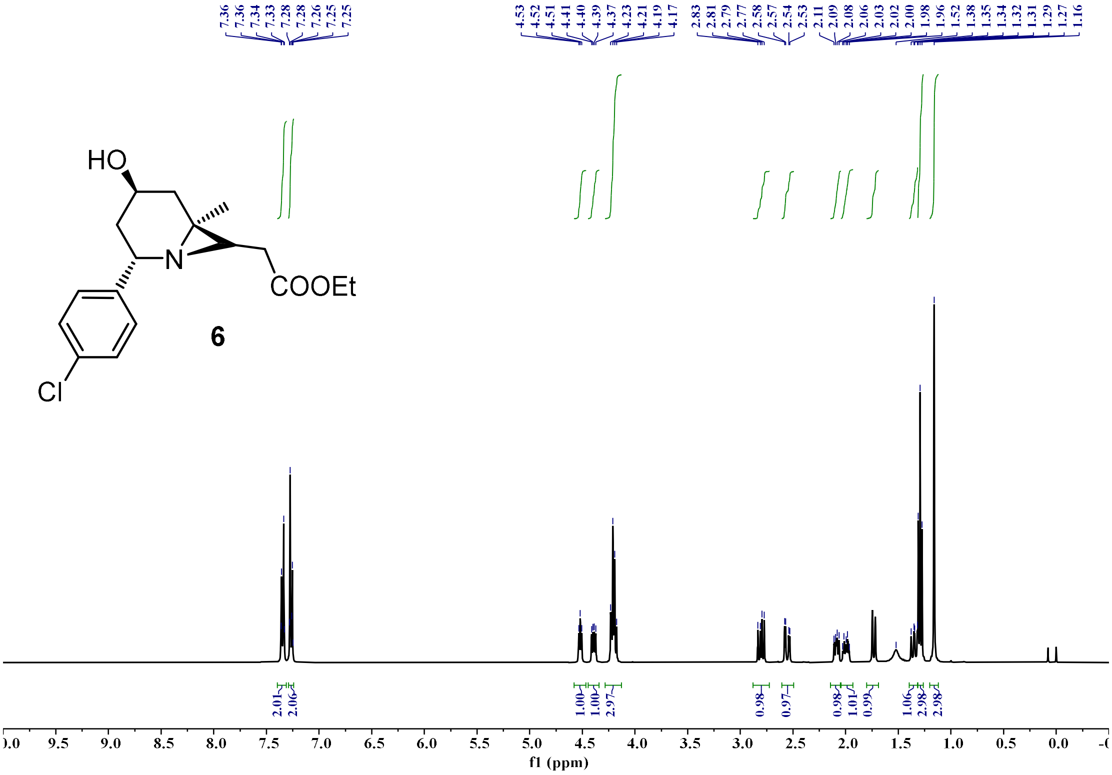

Supplementary Figure 64. ^{1}H NMR spectrum (400 MHz, $CDCl_3$) of **6**

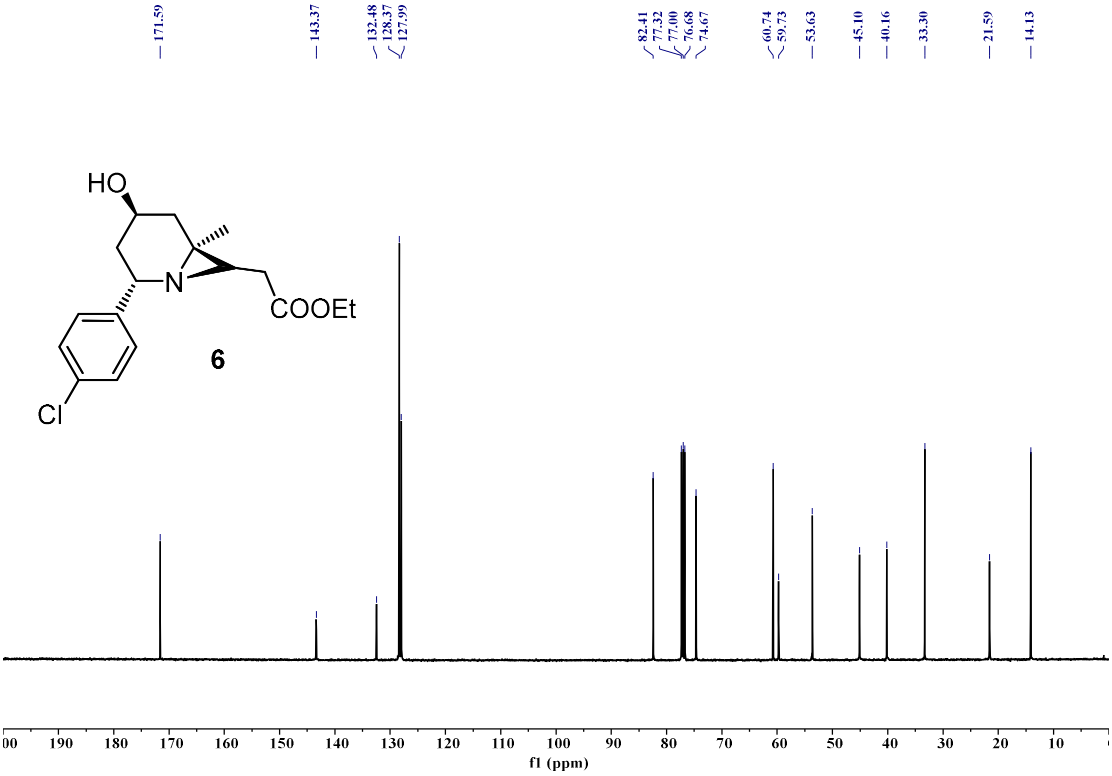

Supplementary Figure 65. ^{13}C NMR spectrum (100 MHz, $CDCl_3$) of **6**

3.2 HPLC spectra

Data File D:\HPLC\DATA\20220505\C 2022-05-05 16-04-56\C.D
Sample Name: FC03-390-a-Me-allyl-rac-1-1

```
=====================================================================
Acq. Operator   : SYSTEM                        Seq. Line :   1
Acq. Instrument : 1260                           Location :   41
Injection Date  : 5/5/2022 4:06:23 PM                 Inj :   1
                                              Inj Volume : 2.000 µl
Acq. Method     : D:\HPLC\Data\20220505\C 2022-05-05 16-04-56\FC-AS-H-95-5-DAD-1ML-10MIN.M
Last changed    : 5/5/2022 4:04:56 PM by SYSTEM
Analysis Method : D:\HPLC\Data\20220505\C 2022-05-05 16-04-56\FC-AS-H-95-5-DAD-1ML-10MIN.M (
                  Sequence Method)
Last changed    : 6/6/2022 3:34:29 PM by SYSTEM
                  (modified after loading)
Additional Info : Peak(s) manually integrated
```

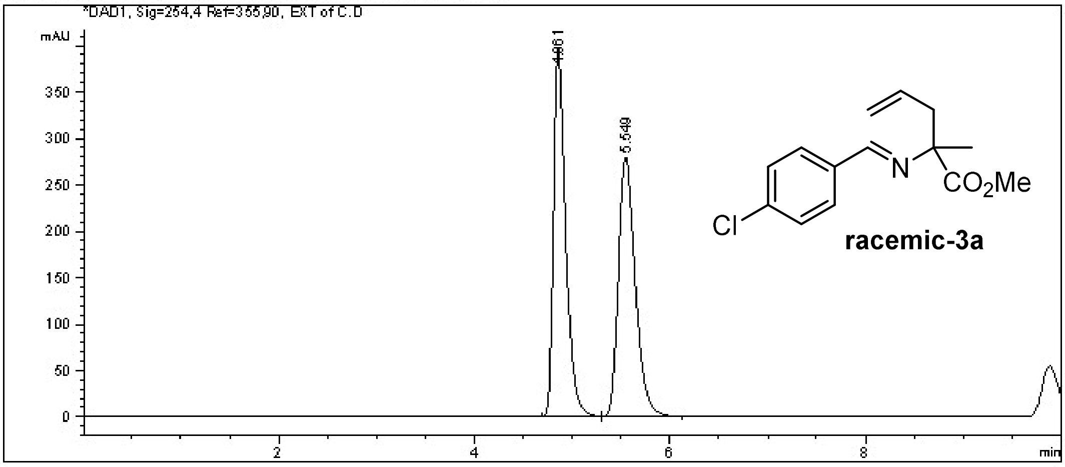


```
=====================================================================
                        Area Percent Report
=====================================================================

Sorted By             :      Signal
Multiplier            :      1.0000
Dilution              :      1.0000
Do not use Multiplier & Dilution Factor with ISTDs


Signal 1: DAD1, Sig=254,4 Ref=355,90, EXT
 Signal has been modified after loading from rawdata file!
```

Peak #	RetTime [min]	Type	Width [min]	Area [mAU*s]	Height [mAU]	Area %
1	4.861	BB	0.1288	3403.88184	398.04025	50.4470
2	5.549	BB	0.1826	3343.55640	279.36945	49.5530
Totals :				6747.43823	677.40970	

```
=====================================================================
                        *** End of Report ***
```

1260 6/6/2022 3:34:38 PM SYSTEM   Page 1 of 1

Supplementary Figure 66. HPLC spectrum of racemic-**3a**

Data File D:\HPLC\Data\20220505\B 2022-05-05 15-16-42\B1.D
Sample Name: FC03-390-a-Me-allyl-SS

===

Acq. Operator : SYSTEM   Seq. Line : 2
Acq. Instrument : 1260   Location : 42
Injection Date : 5/5/2022 3:29:02 PM   Inj : 1
Inj Volume : 2.000 µl
Acq. Method : D:\HPLC\Data\20220505\B 2022-05-05 15-16-42\FC-AS-H-95-5-DAD-1ML-15MIN.M
Last changed : 5/5/2022 3:16:42 PM by SYSTEM
Analysis Method : D:\HPLC\Data\20220505\B 2022-05-05 15-16-42\FC-AS-H-95-5-DAD-1ML-15MIN.M (Sequence Method)
Last changed : 5/11/2022 11:34:36 AM by SYSTEM
(modified after loading)
Additional Info : Peak(s) manually integrated

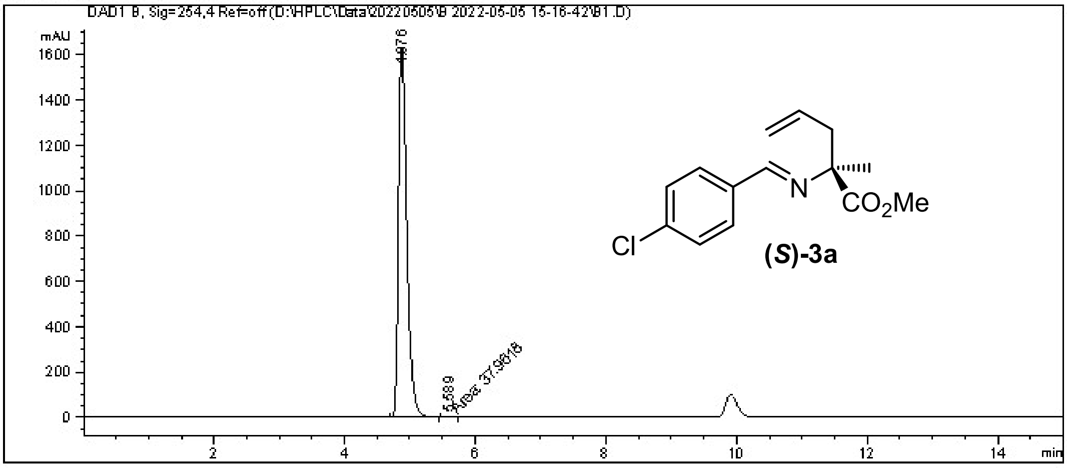

===

Area Percent Report

===

Sorted By : Signal
Multiplier : 1.0000
Dilution : 1.0000
Do not use Multiplier & Dilution Factor with ISTDs

Signal 1: DAD1 B, Sig=254,4 Ref=off

Peak #	RetTime [min]	Type	Width [min]	Area [mAU*s]	Height [mAU]	Area %
1	4.876	BB	0.1329	1.43877e4	1630.70117	99.7368
2	5.589	MM	0.1948	37.96180	3.24845	0.2632

Totals : 1.44257e4 1633.94963

===

*** End of Report ***

1260 5/11/2022 11:35:59 AM SYSTEM   Page 1 of 1

Supplementary Figure 67. HPLC spectrum of (*S*)-**3a**

Data File D:\HPLC\Data\20220523\B 2022-05-23 15-17-50\B4.D
Sample Name: FC-pCl-Me-lac-1-1

===

```
Acq. Operator   : SYSTEM                        Seq. Line :    5
Sample Operator : SYSTEM
Acq. Instrument : 1260                           Location :    1
Injection Date  : 5/23/2022 4:45:18 PM                Inj :    1
                                              Inj Volume : 2.000 µl
Acq. Method     : D:\HPLC\Data\20220523\B 2022-05-23 15-17-50\AD-85-15-210NM-1ML-2uL-20MIN.M
Last changed    : 5/23/2022 11:25:12 AM by SYSTEM
Analysis Method : D:\HPLC\Data\20220523\B 2022-05-23 15-17-50\AD-85-15-210NM-1ML-2uL-20MIN.M
                  (Sequence Method)
Last changed    : 6/2/2022 11:42:26 AM by SYSTEM
                  (modified after loading)
Additional Info : Peak(s) manually integrated
```

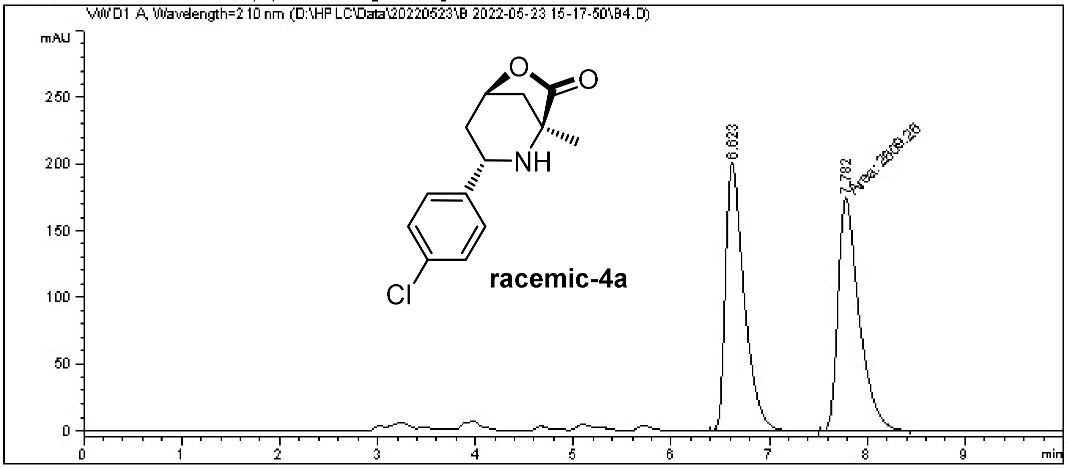

===

Area Percent Report

===

```
Sorted By            :      Signal
Multiplier           :      1.0000
Dilution             :      1.0000
Do not use Multiplier & Dilution Factor with ISTDs
```

Signal 1: VWD1 A, Wavelength=210 nm

Peak #	RetTime [min]	Type	Width [min]	Area [mAU*s]	Height [mAU]	Area %
1	6.623	BB	0.1955	2638.74072	200.54469	50.2808
2	7.782	MF	0.2489	2609.26465	174.72408	49.7192

Totals : 5248.00537 375.26877

===

*** End of Report ***

1260 6/2/2022 11:42:34 AM SYSTEM   Page 1 of 1

Supplementary Figure 68. HPLC spectrum of racemic-**4a**

```
Data File D:\LC\DATA...MeCN-40-S 2021-09-30 15-39-38\FC03-411-Me-lactone-TfOH-MeCN-40-S1.D
Sample Name: FC03-411-Me-lactone-TfOH-MeCN-40-S

=====================================================================
Acq. Operator   : 系统                         Seq. Line :    2
Sample Operator : 系统
Acq. Instrument : 1200                          Location :    1
Injection Date  : 9/30/2021 3:51:59 PM               Inj :    1
                                              Inj Volume : 2.000 µl
Acq. Method     : D:\LC\DATA\FC\FC-1\FC03-411-Me-lactone-TfOH-MeCN-40-S 2021-09-30 15-39-38
                  \ADH-85-15-1ML-2uL-10MIN-210nm.M
Last changed    : 9/10/2021 8:35:24 PM by 系统
Analysis Method : D:\LC\DATA\FC\FC-1\FC03-411-Me-lactone-TfOH-MeCN-40-S 2021-09-30 15-39-38
                  \ADH-85-15-1ML-2uL-10MIN-210nm.M (Sequence Method)
Last changed    : 5/5/2022 8:14:25 PM by 系统
                  (modified after loading)
Additional Info : Peak(s) manually integrated
```

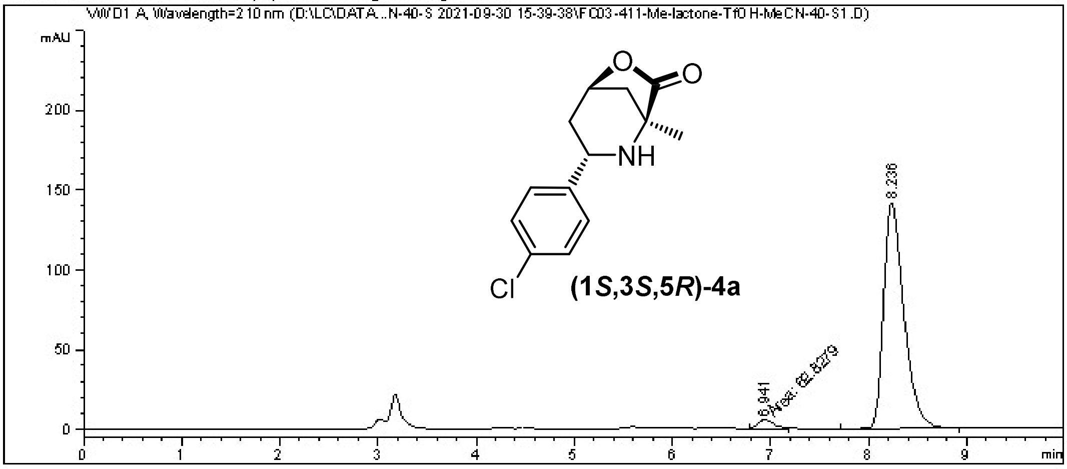


```
=====================================================================
                         Area Percent Report
=====================================================================

Sorted By             :      Signal
Multiplier            :      1.0000
Dilution              :      1.0000
Do not use Multiplier & Dilution Factor with ISTDs


Signal 1: VWD1 A, Wavelength=210 nm
```

Peak #	RetTime [min]	Type	Width [min]	Area [mAU*s]	Height [mAU]	Area %
1	6.941	MM	0.1964	62.82786	5.33284	2.9971
2	8.236	VB R	0.2188	2033.44617	141.36374	97.0029
Totals :				2096.27402	146.69658	

```
=====================================================================
                         *** End of Report ***
```

1200 5/5/2022 8:17:00 PM 系统 Page 1 of 1

Supplementary Figure 69. HPLC spectrum of (1*S*,3*S*,5*R*)-**4a**

Data File D:\LC\DATA...tone-rac-02 2021-10-15 19-50-22\FC03-324-CF3-BrPy-lactone-rac-021.D
Sample Name: FC03-424-CF3-lactone-rac

===

Acq. Operator : 系统 Seq. Line : 2
Sample Operator : 系统
Acq. Instrument : 1200 Location : 43
Injection Date : 10/15/2021 8:02:41 PM Inj : 1
Inj Volume : 2.000 µl
Acq. Method : D:\LC\DATA\FC\FC-1\FC03-424-CF3-BrPy-lactone-rac-02 2021-10-15 19-50-22\ADH-85-15-1ML-2uL-10MIN-210nm.M
Last changed : 9/10/2021 8:35:24 PM by 系统
Analysis Method : D:\LC\DATA\FC\FC-1\FC03-424-CF3-BrPy-lactone-rac-02 2021-10-15 19-50-22\ADH-85-15-1ML-2uL-10MIN-210nm.M (Sequence Method)
Last changed : 5/5/2022 8:20:46 PM by 系统
(modified after loading)
Additional Info : Peak(s) manually integrated

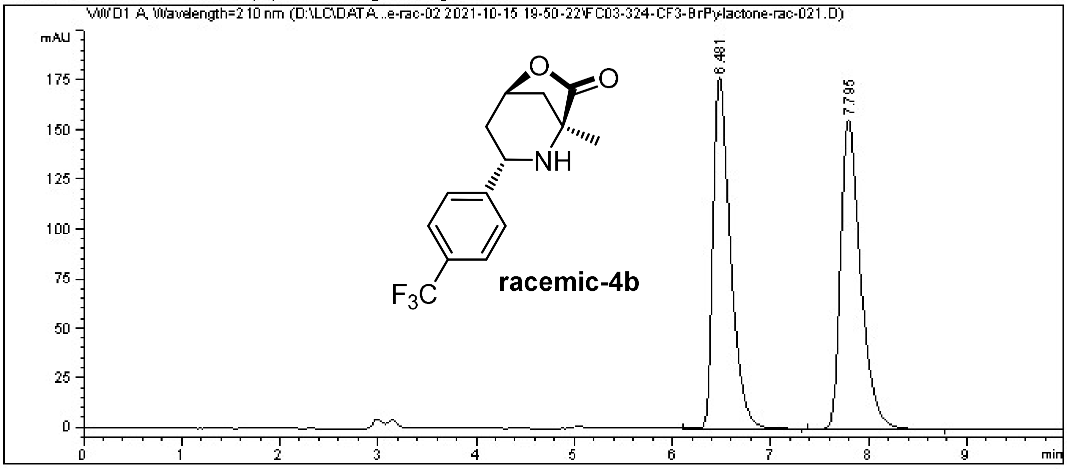

===

Area Percent Report

===

Sorted By : Signal
Multiplier : 1.0000
Dilution : 1.0000
Do not use Multiplier & Dilution Factor with ISTDs

Signal 1: VWD1 A, Wavelength=210 nm

Peak #	RetTime [min]	Type	Width [min]	Area [mAU*s]	Height [mAU]	Area %
1	6.481	VV R	0.1857	2173.13843	176.71045	49.7513
2	7.795	VV R	0.2127	2194.86865	155.54457	50.2487

Totals : 4368.00708 332.25502

===

*** End of Report ***

1200 5/5/2022 8:20:48 PM 系统 Page 1 of 1

Supplementary Figure 70. HPLC spectrum of racemic-**4b**

Data File D:\LC\DATA...Py-lactone-R-S 2021-10-15 17-36-29\FC03-324-CF3-BrPy-lactone-R-S3.D
Sample Name: FC03-424-CF3-lactone-S

===

Acq. Operator : 系统 Seq. Line : 4
Sample Operator : 系统
Acq. Instrument : 1200 Location : 44
Injection Date : 10/15/2021 6:11:04 PM Inj : 1
Inj Volume : 2.000 µl
Acq. Method : D:\LC\DATA\FC\FC-1\FC03-424-CF3-BrPy-lactone-R-S 2021-10-15 17-36-29\ADH-85-15-1ML-2uL-10MIN-210nm.M
Last changed : 9/10/2021 8:35:24 PM by 系统
Analysis Method : D:\LC\DATA\FC\FC-1\FC03-424-CF3-BrPy-lactone-R-S 2021-10-15 17-36-29\ADH-85-15-1ML-2uL-10MIN-210nm.M (Sequence Method)
Last changed : 5/5/2022 8:22:55 PM by 系统
(modified after loading)
Additional Info : Peak(s) manually integrated

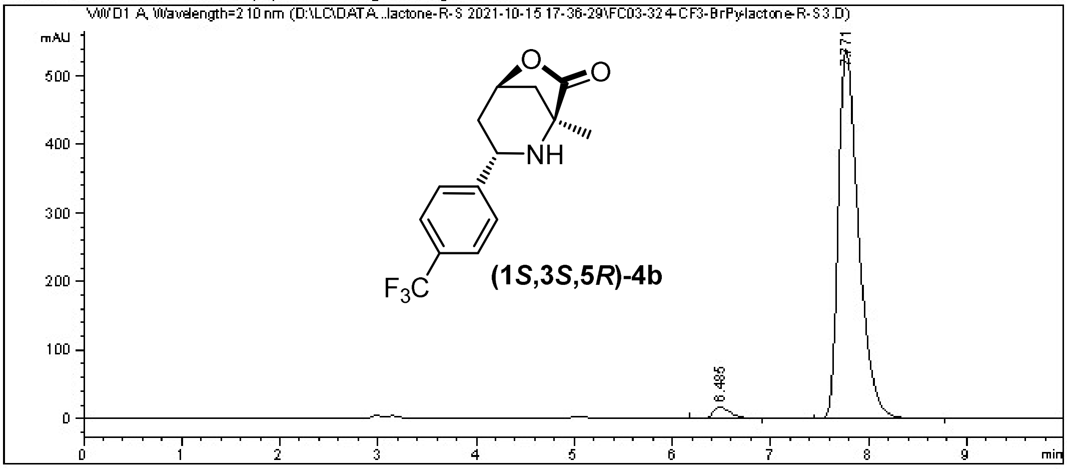

===

Area Percent Report

===

Sorted By : Signal
Multiplier : 1.0000
Dilution : 1.0000
Do not use Multiplier & Dilution Factor with ISTDs

Signal 1: VWD1 A, Wavelength=210 nm

Peak #	RetTime [min]	Type	Width [min]	Area [mAU*s]	Height [mAU]	Area %
1	6.485	VB R	0.1797	205.02260	17.21422	2.5824
2	7.771	BV R	0.2165	7734.11572	540.23621	97.4176

Totals : 7939.13832 557.45043

===

*** End of Report ***

1200 5/5/2022 8:23:31 PM 系统 Page 1 of 1

Supplementary Figure 71. HPLC spectrum of (1*S*,3*S*,5*R*)-**4b**

Data File D:\LC\DATA...CN-lactone-rac-02 2021-10-22 19-35-17\FC03-434-CN-lactone-rac-021.D
Sample Name: FC03-434-CN-lactone-rac

===
Acq. Operator : 系统 Seq. Line : 2
Sample Operator : 系统
Acq. Instrument : 1200 Location : 4
Injection Date : 10/22/2021 7:47:32 PM Inj : 1
Inj Volume : 2.000 µl
Acq. Method : D:\LC\DATA\FC\FC-1\FC03-434-CN-lactone-rac-02 2021-10-22 19-35-17\ADH-75-25-1ML-2uL-20MIN-210 nm.M
Last changed : 10/15/2021 3:27:40 PM by 系统
Analysis Method : D:\LC\DATA\FC\FC-1\FC03-434-CN-lactone-rac-02 2021-10-22 19-35-17\ADH-75-25-1ML-2uL-20MIN-210 nm.M (Sequence Method)
Last changed : 5/5/2022 8:27:22 PM by 系统
(modified after loading)
Additional Info : Peak(s) manually integrated

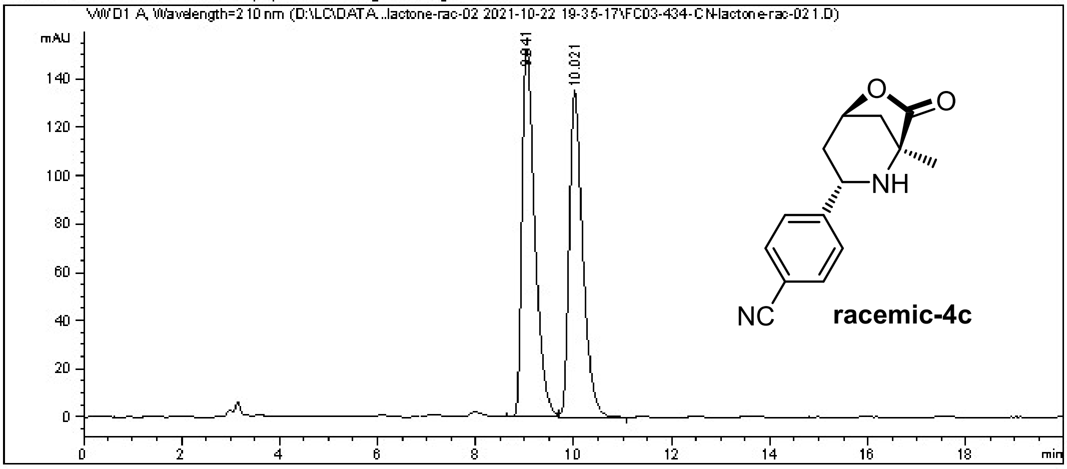

===
Area Percent Report
===

Sorted By : Signal
Multiplier : 1.0000
Dilution : 1.0000
Do not use Multiplier & Dilution Factor with ISTDs

Signal 1: VWD1 A, Wavelength=210 nm

Peak #	RetTime [min]	Type	Width [min]	Area [mAU*s]	Height [mAU]	Area %
1	9.041	VV R	0.2785	2797.90454	152.20767	51.8585
2	10.021	VV R	0.2869	2597.36670	135.11874	48.1415
Totals :				5395.27124	287.32642	

===
*** End of Report ***

Supplementary Figure 72. HPLC spectrum of racemic-**4c**

Data File D:\LC\DATA...3-434-CN-lactone-R-S 2021-10-22 17-36-20\FC03-434-CN-lactone-R-S2.D
Sample Name: FC03-434-CN-lactone-S

===

Acq. Operator : 系统 Seq. Line : 3
Sample Operator : 系统
Acq. Instrument : 1200 Location : 5
Injection Date : 10/22/2021 6:09:35 PM Inj : 1
Inj Volume : 2.000 µl
Acq. Method : D:\LC\DATA\FC\FC-1\FC03-434-CN-lactone-R-S 2021-10-22 17-36-20\ADH-75-25-1ML-2uL-20MIN-210 nm.M
Last changed : 10/15/2021 3:27:40 PM by 系统
Analysis Method : D:\LC\DATA\FC\FC-1\FC03-434-CN-lactone-R-S 2021-10-22 17-36-20\ADH-75-25-1ML-2uL-20MIN-210 nm.M (Sequence Method)
Last changed : 5/6/2022 10:07:33 AM by 系统
(modified after loading)
Additional Info : Peak(s) manually integrated

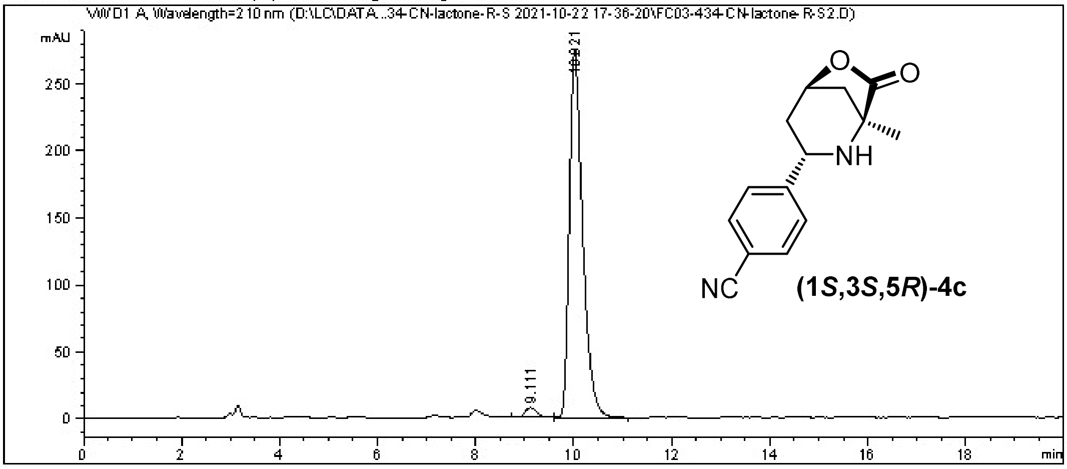

===

Area Percent Report

===

Sorted By : Signal
Multiplier : 1.0000
Dilution : 1.0000
Do not use Multiplier & Dilution Factor with ISTDs

Signal 1: VWD1 A, Wavelength=210 nm

Peak #	RetTime [min]	Type	Width [min]	Area [mAU*s]	Height [mAU]	Area %
1	9.111	BV R	0.2523	123.52682	7.21122	2.3393
2	10.021	BV R	0.2811	5157.03467	275.34158	97.6607

Totals : 5280.56149 282.55280

===

*** End of Report ***

1200 5/6/2022 10:07:35 AM 系统 Page 1 of 1

Supplementary Figure 73. HPLC spectrum of (1*S*,3*S*,5*R*)-**4c**

Data File E:\DATA\FC...CTONE-RAC-TRY 2021-10-23 21-43-04\FC03-434-COOMe-lactone-rac-try6.D
Sample Name: FC03-434-COOMe-lactone-rac

===

```
Acq. Operator   : SYSTEM                        Seq. Line :   7
Acq. Instrument : 1260                           Location :   6
Injection Date  : 10/24/2021 1:48:48 AM               Inj :   1
                                               Inj Volume : 5.000 µl
Acq. Method     : E:\DATA\FC-Prins\FC03-434-COOMe-lactone-rac-try 2021-10-23 21-43-04\FC-OD-H
                  -90-10-DAD-1ML-45MIN.M
Last changed    : 10/23/2021 9:43:04 PM by SYSTEM
Analysis Method : E:\DATA\FC-Prins\FC03-434-COOMe-lactone-rac-try 2021-10-23 21-43-04\FC-OD-H
                  -90-10-DAD-1ML-45MIN.M (Sequence Method)
Last changed    : 5/6/2022 11:01:56 AM by SYSTEM
                  (modified after loading)
Additional Info : Peak(s) manually integrated
```

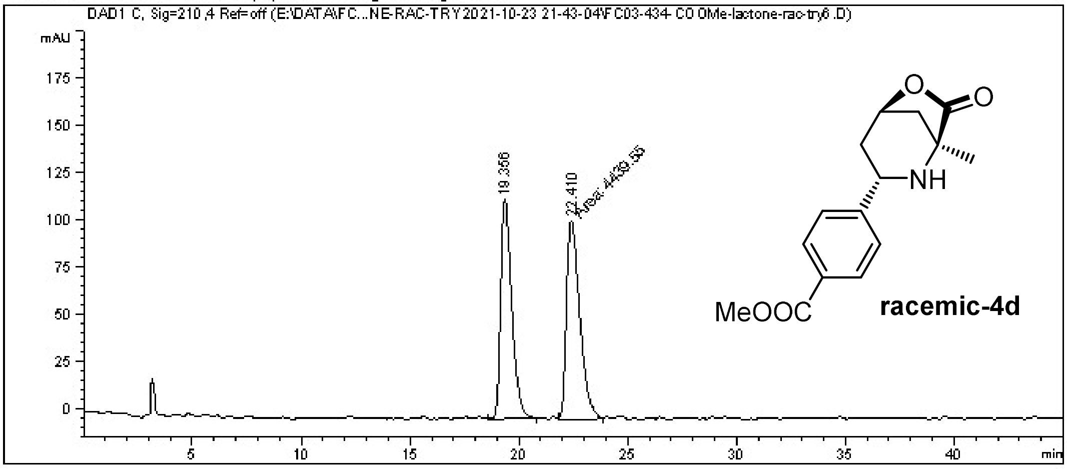

===
Area Percent Report
===

Sorted By : Signal
Multiplier : 1.0000
Dilution : 1.0000
Do not use Multiplier & Dilution Factor with ISTDs

Signal 1: DAD1 C, Sig=210,4 Ref=off

Peak #	RetTime [min]	Type	Width [min]	Area [mAU*s]	Height [mAU]	Area %
1	19.356	BB	0.5169	4117.09229	116.04630	48.1157
2	22.410	FM	0.7041	4439.55371	105.09027	51.8843

Totals : 8556.64600 221.13657

===
*** End of Report ***

Supplementary Figure 74. HPLC spectrum of racemic-**4d**

Data File E:\DATA\FC...COOMe-lactone-R-S 2021-10-24 17-12-51\FC03-434-COOMe-lactone-R-S3.D
Sample Name: FC03-434-COOMe-lactone-S

===
Acq. Operator : SYSTEM Seq. Line : 4
Acq. Instrument : 1260 Location : 26
Injection Date : 10/24/2021 6:23:40 PM Inj : 1
Inj Volume : 5.000 µl
Acq. Method : E:\DATA\FC-Prins\FC03-434-COOMe-lactone-R-S 2021-10-24 17-12-51\FC-OD-H-90-10-DAD-1ML-45MIN.M
Last changed : 10/24/2021 5:12:51 PM by SYSTEM
Analysis Method : E:\DATA\FC-Prins\FC03-434-COOMe-lactone-R-S 2021-10-24 17-12-51\FC-OD-H-90-10-DAD-1ML-45MIN.M (Sequence Method)
Last changed : 5/6/2022 11:06:02 AM by SYSTEM
(modified after loading)
Additional Info : Peak(s) manually integrated

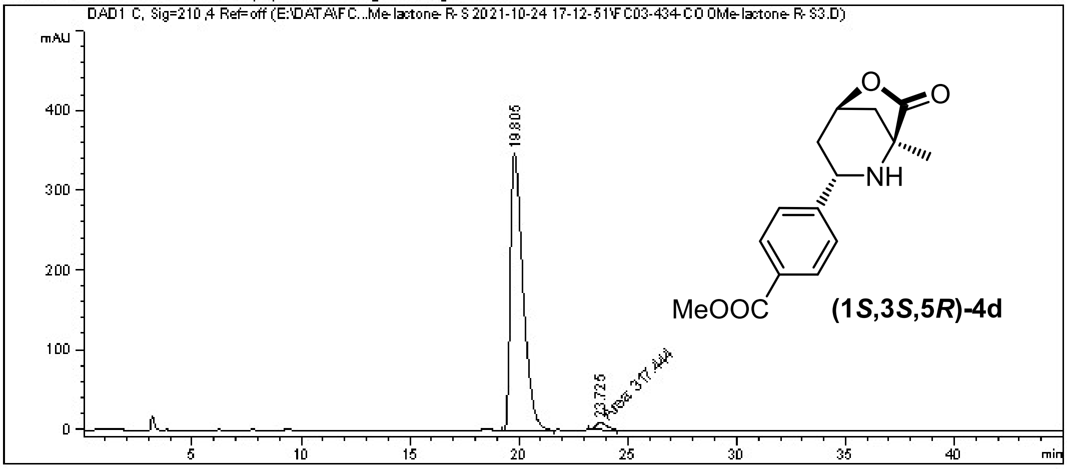

===
Area Percent Report
===

Sorted By : Signal
Multiplier : 1.0000
Dilution : 1.0000
Do not use Multiplier & Dilution Factor with ISTDs

Signal 1: DAD1 C, Sig=210,4 Ref=off

Peak #	RetTime [min]	Type	Width [min]	Area [mAU*s]	Height [mAU]	Area %
1	19.805	BB	0.6094	1.39528e4	347.56320	97.7755
2	23.725	MF	0.5938	317.44400	8.90989	2.2245

Totals : 1.42703e4 356.47309

===
*** End of Report ***

1260 5/6/2022 11:06:04 AM SYSTEM Page 1 of 1

Supplementary Figure 75. HPLC spectrum of (1*S*,3*S*,5*R*)-**4d**

Data File D:\LC\DATA...NO2-lactone-rac-01 2021-10-19 16-07-20\FC03-429-mNO2-lactone-rac1.D
Sample Name: FC03-429-mNO2-lactone-rac

===

Acq. Operator : 系统 Seq. Line : 2
Sample Operator : 系统
Acq. Instrument : 1200 Location : 41
Injection Date : 10/19/2021 4:21:11 PM Inj : 1
Inj Volume : 2.000 µl
Acq. Method : D:\LC\DATA\FC\FC-1\FC03-429-mNO2-lactone-rac-01 2021-10-19 16-07-20\ADH-75-25-1ML-2uL-20MIN-210 nm.M
Last changed : 10/15/2021 3:27:40 PM by 系统
Analysis Method : D:\LC\DATA\FC\FC-1\FC03-429-mNO2-lactone-rac-01 2021-10-19 16-07-20\ADH-75-25-1ML-2uL-20MIN-210 nm.M (Sequence Method)
Last changed : 5/5/2022 8:25:22 PM by 系统
(modified after loading)
Additional Info : Peak(s) manually integrated

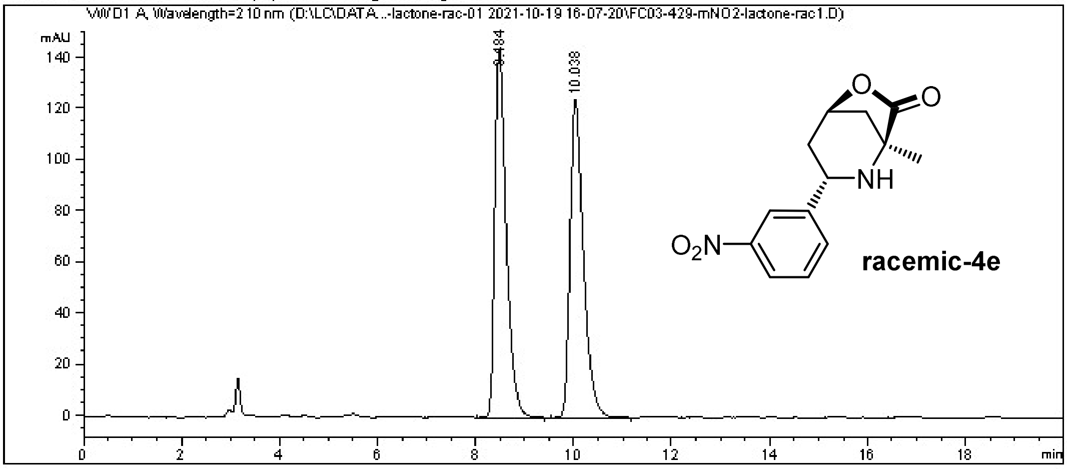

===

Area Percent Report

===

Sorted By : Signal
Multiplier : 1.0000
Dilution : 1.0000
Do not use Multiplier & Dilution Factor with ISTDs

Signal 1: VWD1 A, Wavelength=210 nm

Peak #	RetTime [min]	Type	Width [min]	Area [mAU*s]	Height [mAU]	Area %
1	8.484	VV R	0.2433	2341.53784	144.20670	49.7866
2	10.038	VV R	0.2820	2361.60620	124.76067	50.2134

Totals : 4703.14404 268.96736

===

*** End of Report ***

1200 5/5/2022 8:25:24 PM 系统 Page 1 of 1

Supplementary Figure 76. HPLC spectrum of racemic-**4e**

Data File D:\LC\DATA...9-mNO2-lactone-R-S 2021-10-19 17-54-53\FC03-429-mNO2-lactone-R-S2.D
Sample Name: FC03-429-mNO2-lactone-S

===

Acq. Operator : 系统 Seq. Line : 3
Sample Operator : 系统
Acq. Instrument : 1200 Location : 43
Injection Date : 10/19/2021 6:28:14 PM Inj : 1
Inj Volume : 2.000 µl
Acq. Method : D:\LC\DATA\FC\FC-1\FC03-429-mNO2-lactone-R-S 2021-10-19 17-54-53\ADH-75-25-1ML-2uL-20MIN-210 nm.M
Last changed : 10/15/2021 3:27:40 PM by 系统
Analysis Method : D:\LC\DATA\FC\FC-1\FC03-429-mNO2-lactone-R-S 2021-10-19 17-54-53\ADH-75-25-1ML-2uL-20MIN-210 nm.M (Sequence Method)
Last changed : 5/5/2022 8:26:05 PM by 系统
(modified after loading)
Additional Info : Peak(s) manually integrated

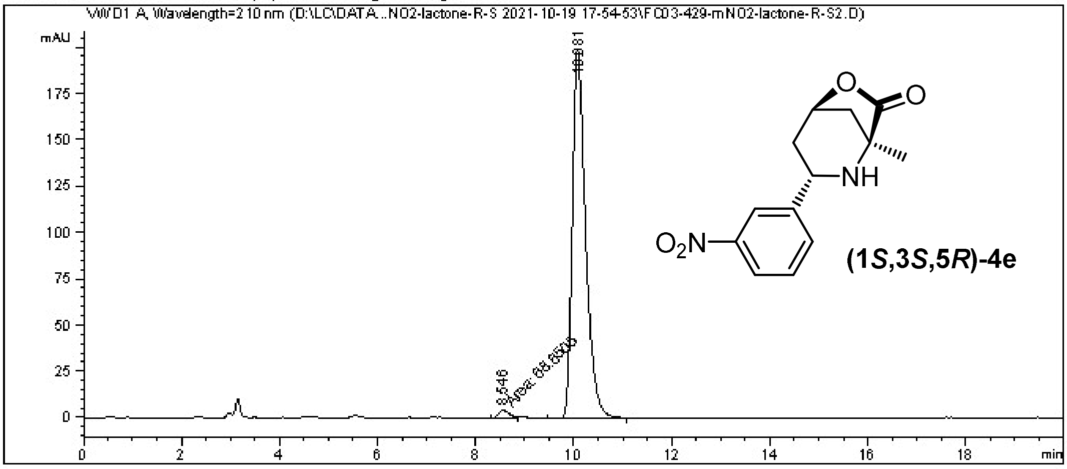

===

Area Percent Report

===

Sorted By : Signal
Multiplier : 1.0000
Dilution : 1.0000
Do not use Multiplier & Dilution Factor with ISTDs

Signal 1: VWD1 A, Wavelength=210 nm

Peak #	RetTime [min]	Type	Width [min]	Area [mAU*s]	Height [mAU]	Area %
1	8.546	MF	0.2604	68.65054	4.39366	1.8029
2	10.081	BV R	0.2799	3739.18408	199.45021	98.1971

Totals : 3807.83462 203.84387

===

*** End of Report ***

1200 5/5/2022 8:26:41 PM 系统 Page 1 of 1

Supplementary Figure 77. HPLC spectrum of (1*S*,3*S*,5*R*)-**4e**

Data File D:\HPLC\Data\20220504\G 2022-05-04 21-34-11\G.D
Sample Name: FC03-437-MeSO2-lac-rac-1-1

===
Acq. Operator : SYSTEM Seq. Line : 1
Acq. Instrument : 1260 Location : 41
Injection Date : 5/4/2022 9:35:10 PM Inj : 1
Inj Volume : 5.000 µl
Acq. Method : D:\HPLC\Data\20220504\G 2022-05-04 21-34-11\IA-60-40-1ML-5uL-20MIN-ALL.M
Last changed : 5/4/2022 9:34:11 PM by SYSTEM
Analysis Method : D:\HPLC\Data\20220504\G 2022-05-04 21-34-11\IA-60-40-1ML-5uL-20MIN-ALL.M (Sequence Method)
Last changed : 6/2/2022 11:25:26 AM by SYSTEM
(modified after loading)
Additional Info : Peak(s) manually integrated

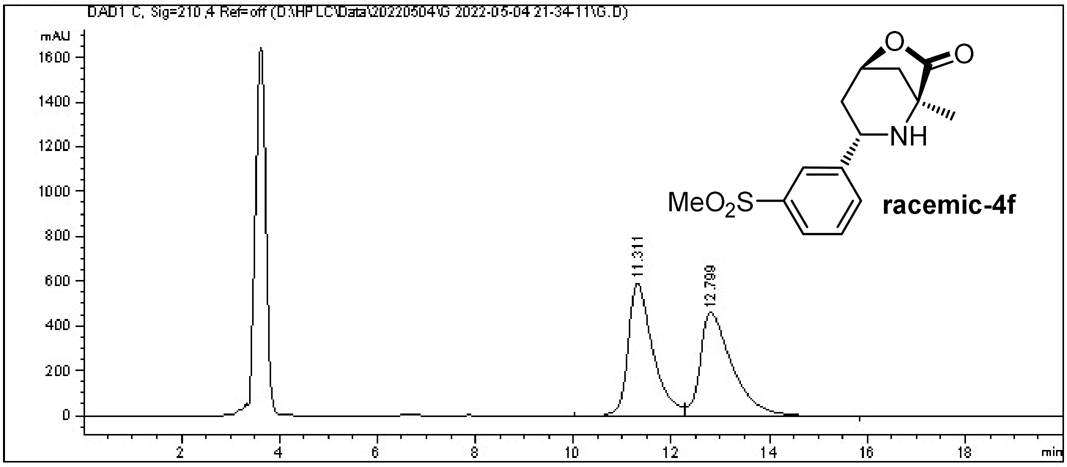

===
Area Percent Report
===

Sorted By : Signal
Multiplier : 1.0000
Dilution : 1.0000
Do not use Multiplier & Dilution Factor with ISTDs

Signal 1: DAD1 C, Sig=210,4 Ref=off

Peak #	RetTime [min]	Type	Width [min]	Area [mAU*s]	Height [mAU]	Area %
1	11.311	BV	0.5203	2.12034e4	597.07129	49.5591
2	12.799	VB	0.6716	2.15807e4	466.62387	50.4409

Totals : 4.27842e4 1063.69516

===
*** End of Report ***

1260 6/2/2022 11:25:28 AM SYSTEM Page 1 of 1

Supplementary Figure 78. HPLC spectrum of racemic-**4f**

Data File D:\HPLC\Data\20220504\F 2022-05-04 20-38-01\F1.D
Sample Name: FC03-437-MeSO2-lac-SS

===

Acq. Operator : SYSTEM Seq. Line : 2
Acq. Instrument : 1260 Location : 42
Injection Date : 5/4/2022 8:44:56 PM Inj : 1
Inj Volume : 5.000 µl
Acq. Method : D:\HPLC\Data\20220504\F 2022-05-04 20-38-01\IA-60-40-1ML-5uL-20MIN-ALL.M
Last changed : 5/4/2022 8:38:01 PM by SYSTEM
Analysis Method : D:\HPLC\Data\20220504\F 2022-05-04 20-38-01\IA-60-40-1ML-5uL-20MIN-ALL.M (Sequence Method)
Last changed : 6/2/2022 11:21:57 AM by SYSTEM
(modified after loading)
Additional Info : Peak(s) manually integrated

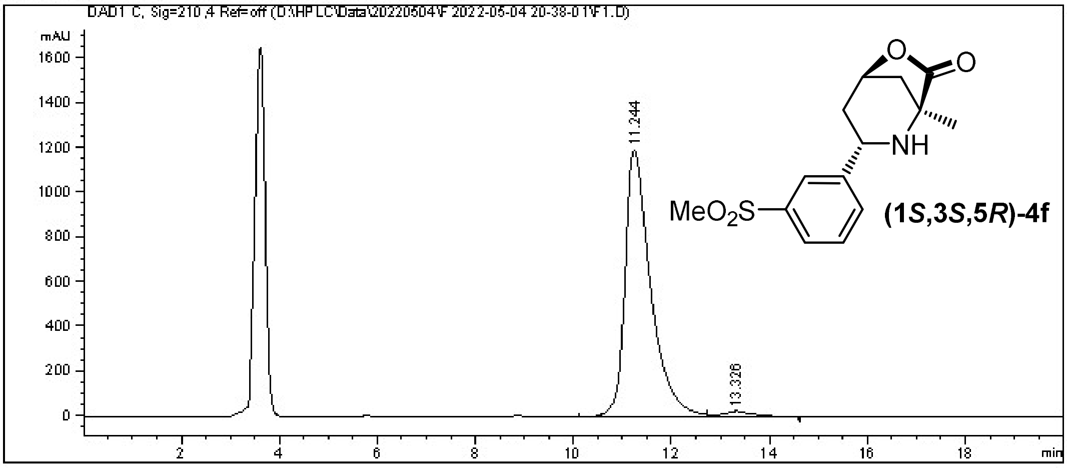

===

Area Percent Report

===

Sorted By : Signal
Multiplier : 1.0000
Dilution : 1.0000
Do not use Multiplier & Dilution Factor with ISTDs

Signal 1: DAD1 C, Sig=210,4 Ref=off

Peak #	RetTime [min]	Type	Width [min]	Area [mAU*s]	Height [mAU]	Area %
1	11.244	BV R	0.5368	4.40017e4	1197.08728	97.9916
2	13.326	VB E	0.5602	901.82245	22.76401	2.0084

Totals : 4.49036e4 1219.85129

===

*** End of Report ***

1260 6/2/2022 11:22:19 AM SYSTEM Page 1 of 1

Supplementary Figure 79. HPLC spectrum of (1*S*,3*S*,5*R*)-**4f**

Data File D:\LC\DATA\20211223\20211223A 2021-12-23 08-49-26\A20.D
Sample Name: FC04-517-oCl-lac-rac

===

Acq. Operator : 系统 Seq. Line : 21
Sample Operator : 系统
Acq. Instrument : 1200 Location : 3
Injection Date : 12/23/2021 4:31:18 PM Inj : 1
Inj Volume : 10.000 µl
Acq. Method : D:\LC\DATA\20211223\20211223A 2021-12-23 08-49-26\ADH-85-15-1ML-10uL-20MIN-210nm.M
Last changed : 12/17/2021 8:49:14 PM by 系统
Analysis Method : D:\LC\DATA\20211223\20211223A 2021-12-23 08-49-26\ADH-85-15-1ML-10uL-20MIN-210nm.M (Sequence Method)
Last changed : 5/11/2022 11:01:56 AM by 系统
(modified after loading)
Additional Info : Peak(s) manually integrated

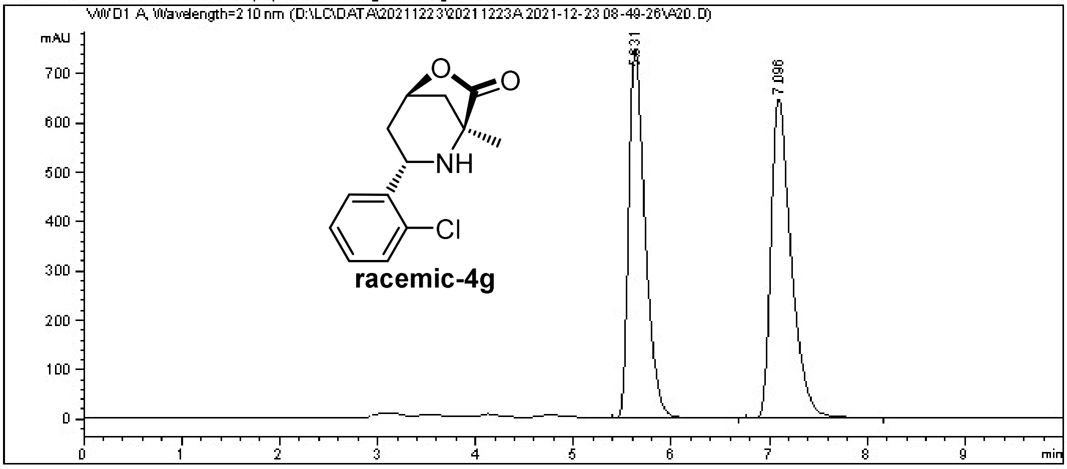

===

Area Percent Report

===

Sorted By : Signal
Multiplier : 1.0000
Dilution : 1.0000
Do not use Multiplier & Dilution Factor with ISTDs

Signal 1: VWD1 A, Wavelength=210 nm

Peak #	RetTime [min]	Type	Width [min]	Area [mAU*s]	Height [mAU]	Area %
1	5.631	BB	0.1753	8725.97949	748.59528	48.4356
2	7.096	BB	0.2138	9289.66113	648.05359	51.5644

Totals : 1.80156e4 1396.64886

===

*** End of Report ***

1200 5/11/2022 11:03:57 AM 系统 Page 1 of 1

Supplementary Figure 80. HPLC spectrum of racemic-**4g**

Data File D:\LC\DATA\20211223\20211223A 2021-12-23 08-49-26\A18.D
Sample Name: FC04-517-oCl-lac-S

===

Acq. Operator : 系统 Seq. Line : 19
Sample Operator : 系统
Acq. Instrument : 1200 Location : 2
Injection Date : 12/23/2021 3:48:47 PM Inj : 1
Inj Volume : 10.000 µl
Acq. Method : D:\LC\DATA\20211223\20211223A 2021-12-23 08-49-26\ADH-85-15-1ML-10uL-20MIN-210nm.M
Last changed : 12/17/2021 8:49:14 PM by 系统
Analysis Method : D:\LC\DATA\20211223\20211223A 2021-12-23 08-49-26\ADH-85-15-1ML-10uL-20MIN-210nm.M (Sequence Method)
Last changed : 5/11/2022 11:01:56 AM by 系统
(modified after loading)
Additional Info : Peak(s) manually integrated

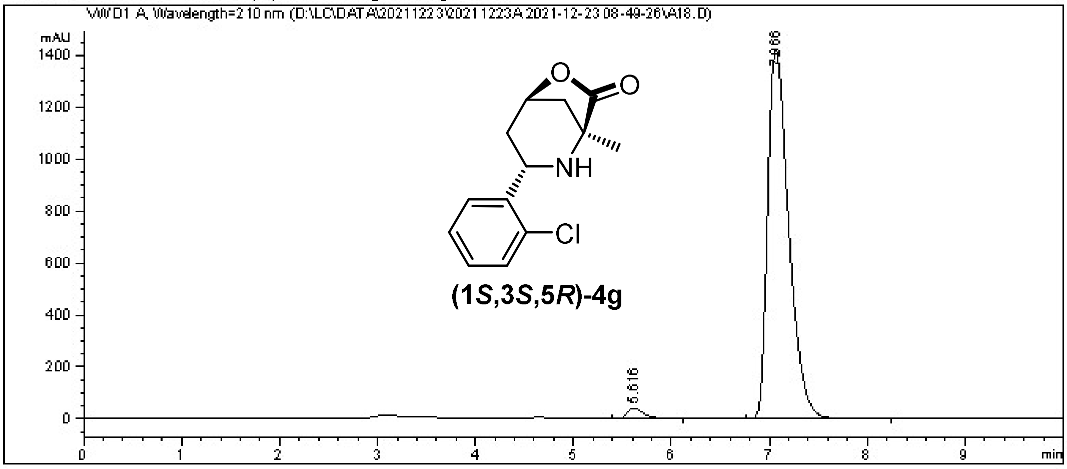

===

Area Percent Report

===

Sorted By : Signal
Multiplier : 1.0000
Dilution : 1.0000
Do not use Multiplier & Dilution Factor with ISTDs

Signal 1: VWD1 A, Wavelength=210 nm

Peak #	RetTime [min]	Type	Width [min]	Area [mAU*s]	Height [mAU]	Area %
1	5.616	BB	0.1715	463.85449	40.48661	2.1369
2	7.066	BV R	0.2278	2.12432e4	1424.77808	97.8631

Totals : 2.17070e4 1465.26469

===

*** End of Report ***

1200 5/11/2022 11:03:20 AM 系统 Page 1 of 1

Supplementary Figure 81. HPLC spectrum of (1*S*,3*S*,5*R*)-**4g**

Data File D:\LC\DATA...e-rac 2021-11-25 15-00-18\FC04-459-Ph-CH2CH2COOMe-lactone-rac-021.D
Sample Name: FC04-459-Ph-lactone-rac

===

Acq. Operator : 系统 Seq. Line : 2
Sample Operator : 系统
Acq. Instrument : 1200 Location : 1
Injection Date : 11/25/2021 3:12:29 PM Inj : 1
Inj Volume : 2.000 µl
Acq. Method : D:\LC\DATA\FC\FC-1\FC04-459-Ph-CH2CH2COOMe-lactone-rac 2021-11-25 15-00-18\ADH-90-10-1ML-2uL-20MIN-210nm.M
Last changed : 9/10/2021 11:11:47 AM by 系统
Analysis Method : D:\LC\DATA\FC\FC-1\FC04-459-Ph-CH2CH2COOMe-lactone-rac 2021-11-25 15-00-18\ADH-90-10-1ML-2uL-20MIN-210nm.M (Sequence Method)
Last changed : 5/6/2022 11:31:43 AM by 系统
(modified after loading)
Additional Info : Peak(s) manually integrated

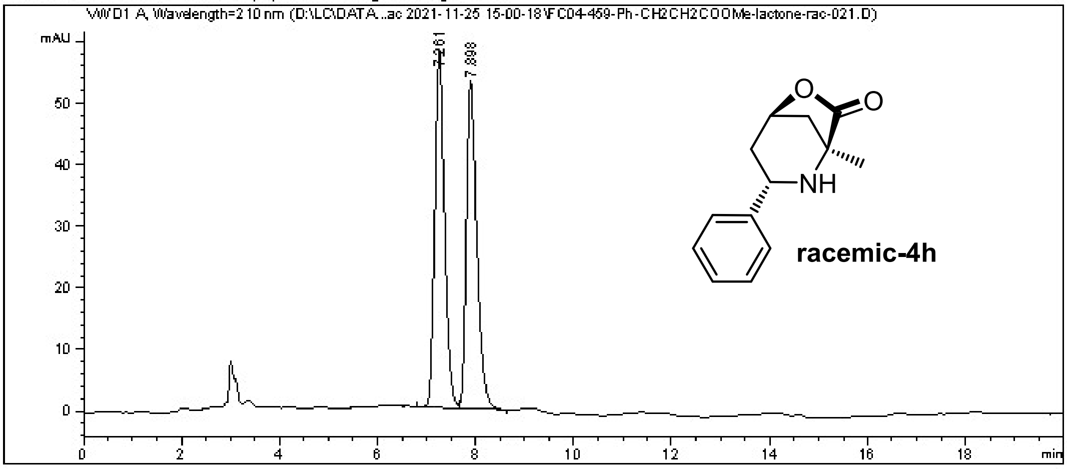

===

Area Percent Report

===

Sorted By : Signal
Multiplier : 1.0000
Dilution : 1.0000
Do not use Multiplier & Dilution Factor with ISTDs

Signal 1: VWD1 A, Wavelength=210 nm

Peak #	RetTime [min]	Type	Width [min]	Area [mAU*s]	Height [mAU]	Area %
1	7.261	BV	0.2002	795.35626	58.21394	50.4597
2	7.898	VB	0.2223	780.86353	53.16894	49.5403

Totals : 1576.21979 111.38288

===

*** End of Report ***

1200 5/6/2022 11:31:45 AM 系统 Page 1 of 1

Supplementary Figure 82. HPLC spectrum of racemic-**4h**

Data File D:\LC\DATA...-R-S 2021-11-25 11-30-57\FC04-459-Ph-CH2CH2COOMe-pMePh-lactone-R3.D
Sample Name: FC04-459-Ph-lactone-S

===

Acq. Operator : 系統 Seq. Line : 4
Sample Operator : 系統
Acq. Instrument : 1200 Location : 2
Injection Date : 11/25/2021 12:15:08 PM Inj : 1
Inj Volume : 2.000 µl
Acq. Method : D:\LC\DATA\FC\FC-1\FC04-459-Ph-CH2CH2COOMe-pMePh-lactone-R-S 2021-11-25 11-30-57\ADH-90-10-1ML-2uL-20MIN-210nm.M
Last changed : 9/10/2021 11:11:47 AM by 系統
Analysis Method : D:\LC\DATA\FC\FC-1\FC04-459-Ph-CH2CH2COOMe-pMePh-lactone-R-S 2021-11-25 11-30-57\ADH-90-10-1ML-2uL-20MIN-210nm.M (Sequence Method)
Last changed : 5/6/2022 11:32:42 AM by 系統
(modified after loading)
Additional Info : Peak(s) manually integrated

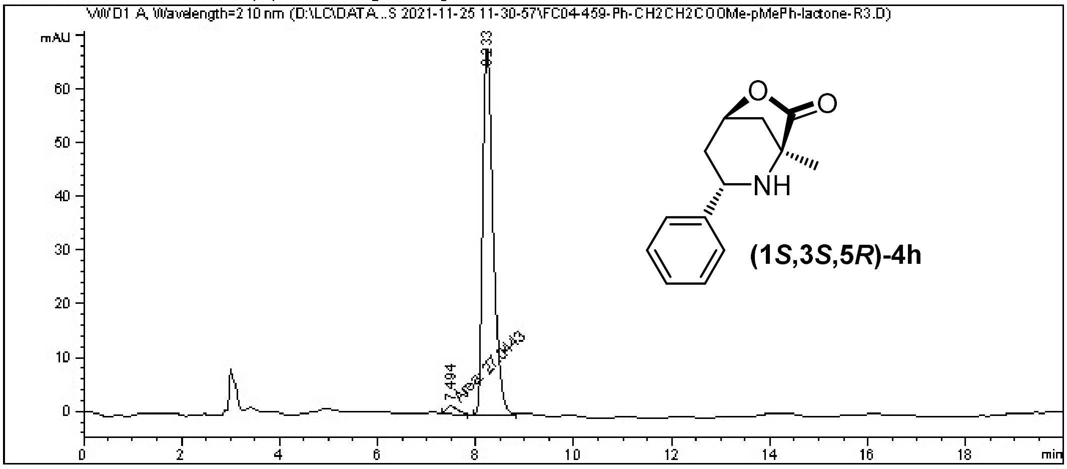

===

Area Percent Report

===

Sorted By : Signal
Multiplier : 1.0000
Dilution : 1.0000
Do not use Multiplier & Dilution Factor with ISTDs

Signal 1: VWD1 A, Wavelength=210 nm

Peak #	RetTime [min]	Type	Width [min]	Area [mAU*s]	Height [mAU]	Area %
1	7.494	MM	0.2747	27.04429	1.64088	2.6221
2	8.233	BB	0.2235	1004.36444	67.93081	97.3779

Totals : 1031.40874 69.57169

===

*** End of Report ***

1200 5/6/2022 11:33:32 AM 系統 Page 1 of 1

Supplementary Figure 83. HPLC spectrum of (1*S*,3*S*,5*R*)-**4h**

Data File D:\LC\DATA...Ph-lactone-rac-02 2021-11-27 08-50-18\FC04-473-pMePh-lactone-rac1.D
Sample Name: FC04-473-pMePh-lactone-rac

===

Acq. Operator : 系統 Seq. Line : 2
Sample Operator : 系統
Acq. Instrument : 1200 Location : 1
Injection Date : 11/27/2021 9:02:56 AM Inj : 1
Inj Volume : 2.000 µl
Acq. Method : D:\LC\DATA\FC\FC-1\FC04-473-pMePh-lactone-rac-02 2021-11-27 08-50-18\ADH-85-15-1ML-2uL-20MIN-210nm.M
Last changed : 9/10/2021 8:34:32 PM by 系統
Analysis Method : D:\LC\DATA\FC\FC-1\FC04-473-pMePh-lactone-rac-02 2021-11-27 08-50-18\ADH-85-15-1ML-2uL-20MIN-210nm.M (Sequence Method)
Last changed : 5/6/2022 11:37:11 AM by 系統
(modified after loading)
Additional Info : Peak(s) manually integrated

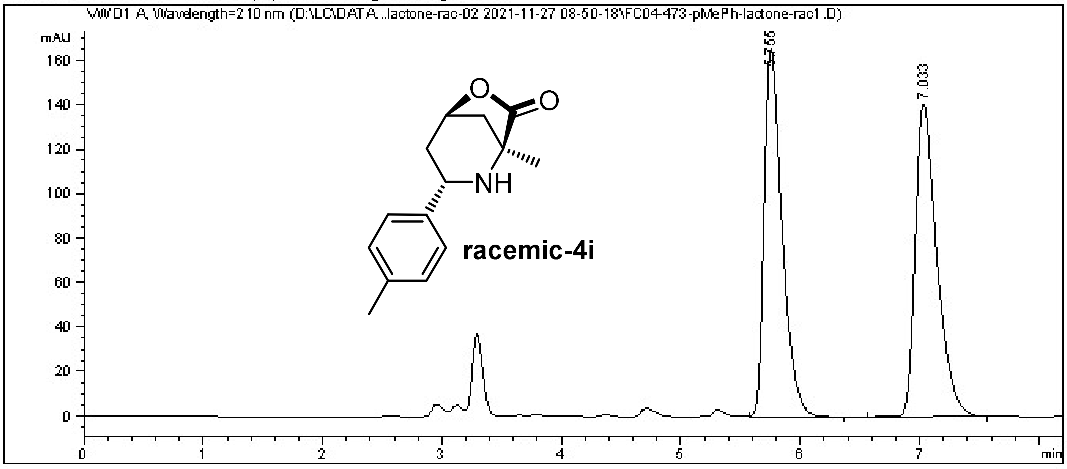

===

Area Percent Report

===

Sorted By : Signal
Multiplier : 1.0000
Dilution : 1.0000
Do not use Multiplier & Dilution Factor with ISTDs

Signal 1: VWD1 A, Wavelength=210 nm

Peak #	RetTime [min]	Type	Width [min]	Area [mAU*s]	Height [mAU]	Area %
1	5.755	VB	0.1576	1746.57764	166.07718	49.6020
2	7.033	VB R	0.1891	1774.60303	140.97704	50.3980

Totals : 3521.18066 307.05421

===

*** End of Report ***

1200 5/6/2022 11:37:14 AM 系統 Page 1 of 1

Supplementary Figure 84. HPLC spectrum of racemic-**4i**

Data File D:\LC\DATA...pMePh-lactone-R-S 2021-11-25 16-28-20\FC04-473-pMePh-lactone-R-S3.D
Sample Name: FC04-473-pMePh-lactone-S

===

Acq. Operator : 系統   Seq. Line : 4
Sample Operator : 系統
Acq. Instrument : 1200   Location : 2
Injection Date : 11/25/2021 5:02:26 PM   Inj : 1
Inj Volume : 2.000 µl
Acq. Method : D:\LC\DATA\FC\FC-1\FC04-473-pMePh-lactone-R-S 2021-11-25 16-28-20\ADH-85-15-1ML-2uL-10MIN-210nm.M
Last changed : 9/10/2021 8:35:24 PM by 系統
Analysis Method : D:\LC\DATA\FC\FC-1\FC04-473-pMePh-lactone-R-S 2021-11-25 16-28-20\ADH-85-15-1ML-2uL-10MIN-210nm.M (Sequence Method)
Last changed : 5/6/2022 11:38:04 AM by 系統
(modified after loading)
Additional Info : Peak(s) manually integrated

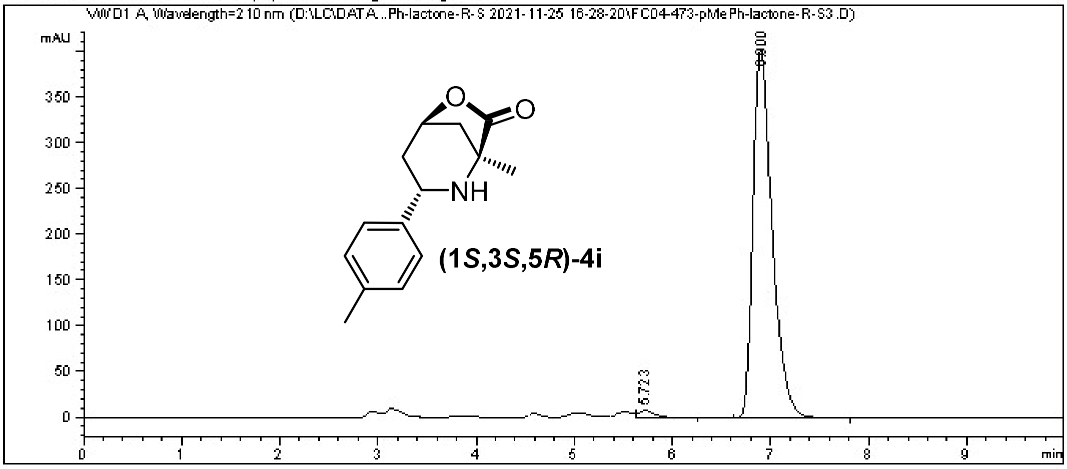

===

Area Percent Report

===

Sorted By : Signal
Multiplier : 1.0000
Dilution : 1.0000
Do not use Multiplier & Dilution Factor with ISTDs

Signal 1: VWD1 A, Wavelength=210 nm

Peak #	RetTime [min]	Type	Width [min]	Area [mAU*s]	Height [mAU]	Area %
1	5.723	VB	0.1636	97.63725	8.65813	1.7504
2	6.900	BB	0.2053	5480.31445	402.91684	98.2496

Totals : 5577.95171 411.57497

===

*** End of Report ***

1200 5/6/2022 11:38:39 AM 系統   Page 1 of 1

Supplementary Figure 85. HPLC spectrum of (1*S*,3*S*,5*R*)-**4i**

Data File D:\LC\DATA...n-PhNH-4-Ar-Bz 2021-12-13 10-30-08\CC-15-39B-S-down-PhNH-4-Ar-Bz6.D
Sample Name: FC04-491-mMe-lac-rac-1-1

```
=====================================================================
Acq. Operator   : 系统                        Seq. Line :   7
Sample Operator : 系统
Acq. Instrument : 1200                          Location :   4
Injection Date  : 12/13/2021 1:06:51 PM              Inj :   1
                                             Inj Volume : 2.000 µl
Acq. Method     : D:\LC\DATA\CC\CC-15-39B-S-down-PhNH-4-Ar-Bz\CC-15-39B-S-down-PhNH-4-Ar-Bz
                  2021-12-13 10-30-08\ADH-85-15-1ML-2uL-20MIN-210nm.M
Last changed    : 9/10/2021 8:34:32 PM by 系统
Analysis Method : D:\LC\DATA\CC\CC-15-39B-S-down-PhNH-4-Ar-Bz\CC-15-39B-S-down-PhNH-4-Ar-Bz
                  2021-12-13 10-30-08\ADH-85-15-1ML-2uL-20MIN-210nm.M (Sequence Method)
Last changed    : 5/11/2022 10:47:39 AM by 系统
                  (modified after loading)
Additional Info : Peak(s) manually integrated
```

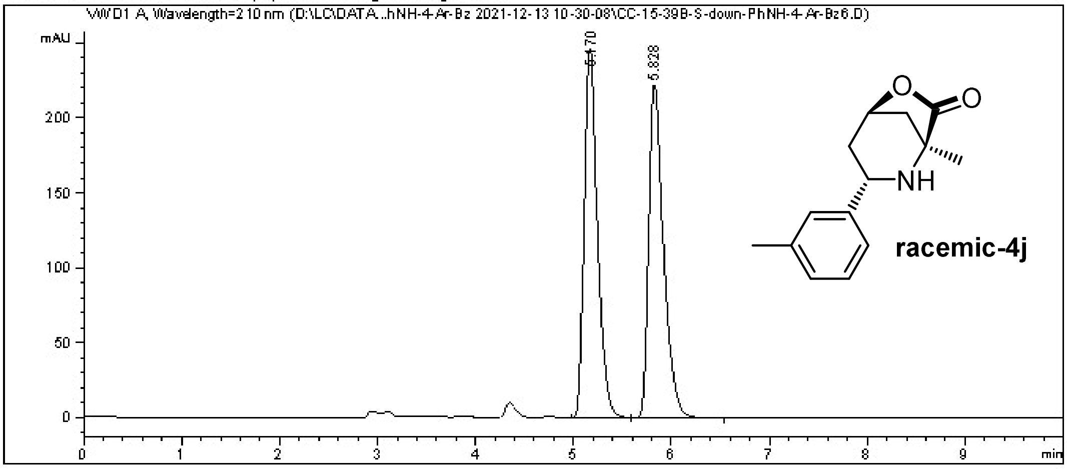


```
=====================================================================
                         Area Percent Report
=====================================================================

Sorted By             :      Signal
Multiplier            :      1.0000
Dilution              :      1.0000
Do not use Multiplier & Dilution Factor with ISTDs


Signal 1: VWD1 A, Wavelength=210 nm
```

Peak #	RetTime [min]	Type	Width [min]	Area [mAU*s]	Height [mAU]	Area %
1	5.170	VB	0.1410	2297.29150	245.86736	49.6167
2	5.828	BB	0.1574	2332.78809	222.19489	50.3833

Totals :	4630.07959	468.06224

```
=====================================================================
                          *** End of Report ***
```

1200 5/11/2022 10:47:41 AM 系统 Page 1 of 1

Supplementary Figure 86. HPLC spectrum of racemic-**4j**

Data File D:\LC\DATA\20211210\E 2021-12-10 16-19-34\E14.D
Sample Name: FC04-491-mMe-lac-S

===
Acq. Operator   : 系統   Seq. Line : 15
Sample Operator : 系統
Acq. Instrument : 1200   Location :   6
Injection Date : 12/10/2021 10:24:49 PM   Inj :   1
                                            Inj Volume : 2.000 µl
Acq. Method   : D:\LC\DATA\20211210\E 2021-12-10 16-19-34\ADH-85-15-1ML-2uL-20MIN-210nm.M
Last changed   : 9/10/2021 8:34:32 PM by 系統
Analysis Method : D:\LC\DATA\20211210\E 2021-12-10 16-19-34\ADH-85-15-1ML-2uL-20MIN-210nm.M (
                  Sequence Method)
Last changed   : 5/11/2022 10:28:10 AM by 系統
                  (modified after loading)
Additional Info : Peak(s) manually integrated

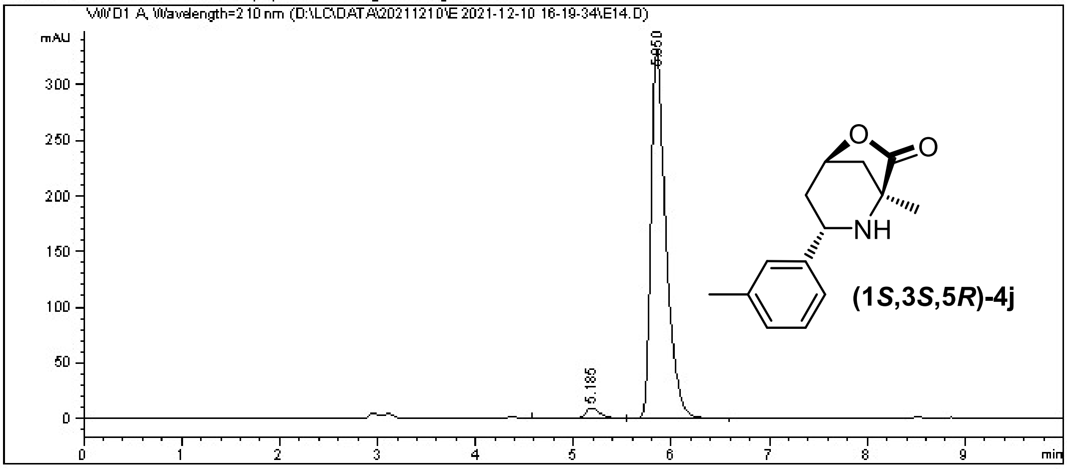

===
Area Percent Report
===

Sorted By   :   Signal
Multiplier   :   1.0000
Dilution   :   1.0000
Do not use Multiplier & Dilution Factor with ISTDs

Signal 1: VWD1 A, Wavelength=210 nm

Peak #	RetTime [min]	Type	Width [min]	Area [mAU*s]	Height [mAU]	Area %
1	5.185	VV R	0.1580	93.22444	8.83866	2.6098
2	5.850	VB	0.1558	3478.81274	331.76486	97.3902

Totals :   3572.03719   340.60352

===
*** End of Report ***

1200 5/11/2022 10:31:56 AM 系統   Page 1 of 1

Supplementary Figure 87. HPLC spectrum of (1*S*,3*S*,5*R*)-**4j**

Data File D:\LC\DATA...oMe-SO2-rac-try 2021-10-30 09-10-17\FC03-442-Nap-oMe-SO2-rac-try2.D
Sample Name: FC03-442-oMe-lactone-rac

```
=====================================================================
Acq. Operator   : 系統                          Seq. Line :   3
Sample Operator : 系統
Acq. Instrument : 1200                           Location :   12
Injection Date  : 10/30/2021 9:43:38 AM               Inj :   1
                                             Inj Volume : 2.000 µl
Acq. Method     : D:\LC\DATA\FC\FC-1\FC03-442-Nap-oMe-SO2-rac-try 2021-10-30 09-10-17\ADH-85-
                  15-1ML-2uL-20MIN-210nm.M
Last changed    : 9/10/2021 8:34:32 PM by 系統
Analysis Method : D:\LC\DATA\FC\FC-1\FC03-442-Nap-oMe-SO2-rac-try 2021-10-30 09-10-17\ADH-85-
                  15-1ML-2uL-20MIN-210nm.M (Sequence Method)
Last changed    : 5/6/2022 10:39:28 AM by 系統
                  (modified after loading)
Additional Info : Peak(s) manually integrated
```

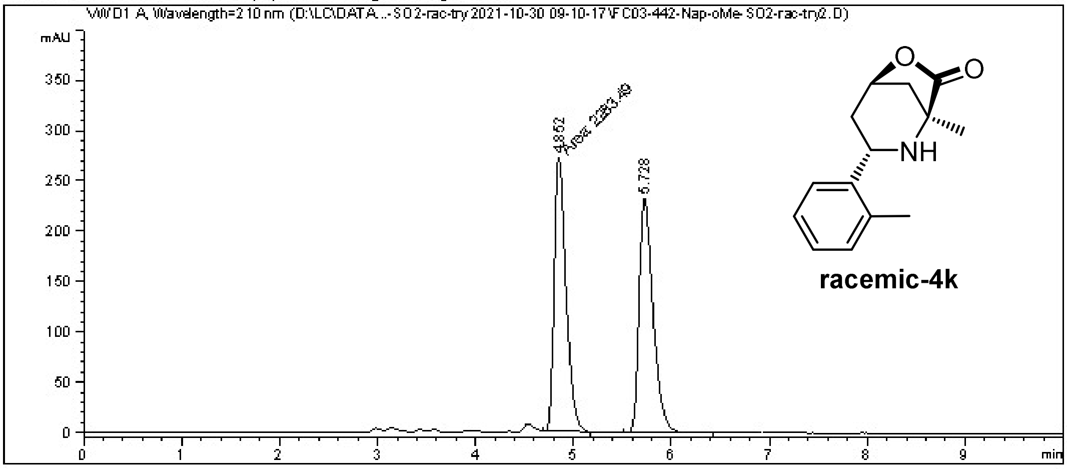


```
=====================================================================
                       Area Percent Report
=====================================================================

Sorted By            :      Signal
Multiplier           :      1.0000
Dilution             :      1.0000
Do not use Multiplier & Dilution Factor with ISTDs


Signal 1: VWD1 A, Wavelength=210 nm
```

Peak #	RetTime [min]	Type	Width [min]	Area [mAU*s]	Height [mAU]	Area %
1	4.852	MM	0.1397	2283.49219	272.33163	49.6730
2	5.728	BV R	0.1490	2313.55957	233.76357	50.3270

Totals : 4597.05176 506.09520

```
=====================================================================
                       *** End of Report ***
```

1200 5/6/2022 10:39:37 AM 系統 Page 1 of 1

Supplementary Figure 88. HPLC spectrum of racemic-**4k**

Data File D:\LC\DATA...oMe-lactone-R-S 2021-10-31 19-08-47\FC03-442-Nap-oMe-lactone-R-S6.D
Sample Name: FC03-442-2-oMe-lactone-S

```
=====================================================================
Acq. Operator   : 系统                          Seq. Line :   7
Sample Operator : 系统
Acq. Instrument : 1200                           Location :   4
Injection Date  : 10/31/2021 8:46:06 PM               Inj :   1
                                              Inj Volume : 2.000 µl
Acq. Method     : D:\LC\DATA\FC\FC-1\FC03-442-2-Nap-oMe-lactone-R-S 2021-10-31 19-08-47\ADH-
                  85-15-1ML-2uL-20MIN-210nm.M
Last changed    : 9/10/2021 8:34:32 PM by 系统
Analysis Method : D:\LC\DATA\FC\FC-1\FC03-442-2-Nap-oMe-lactone-R-S 2021-10-31 19-08-47\ADH-
                  85-15-1ML-2uL-20MIN-210nm.M (Sequence Method)
Last changed    : 5/6/2022 10:35:00 AM by 系统
                  (modified after loading)
Additional Info : Peak(s) manually integrated
```

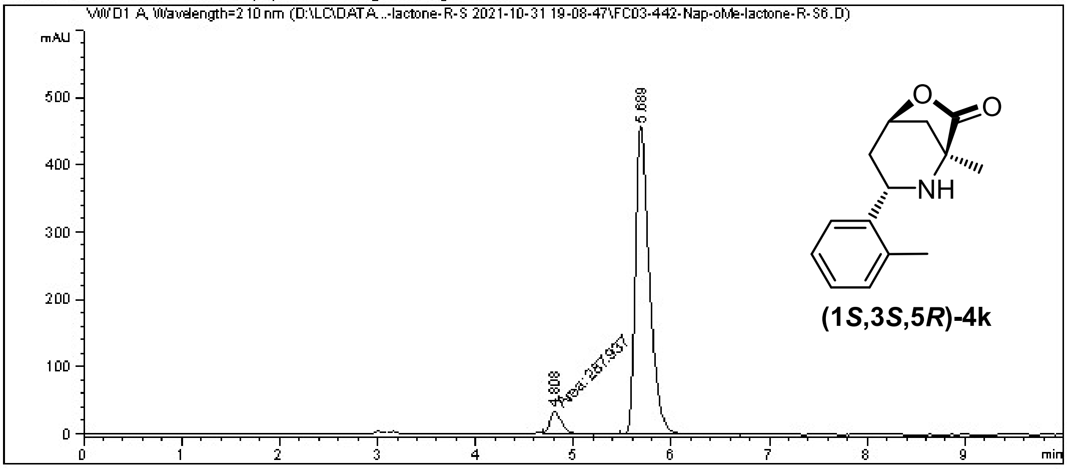


```
=====================================================================
                         Area Percent Report
=====================================================================

Sorted By             :      Signal
Multiplier            :      1.0000
Dilution              :      1.0000
Do not use Multiplier & Dilution Factor with ISTDs


Signal 1: VWD1 A, Wavelength=210 nm

Peak RetTime Type  Width     Area      Height     Area
  #   [min]        [min]   [mAU*s]     [mAU]        %
----|-------|----|-------|----------|----------|--------|
   1   4.808 FM     0.1395  287.93695   34.38921   5.9243
   2   5.689 BV R   0.1501 4572.32617  457.40448  94.0757

Totals :                   4860.26312  491.79369

=====================================================================
                          *** End of Report ***
```

1200 5/6/2022 10:35:05 AM 系统 Page 1 of 1

Supplementary Figure 89. HPLC spectrum of (1*S*,3*S*,5*R*)-**4k**

Data File D:\LC\DATA\20211213\20211213A 2021-12-13 14-36-47\A6.D
Sample Name: FC03-446-p-iBu-lac-rac-1-1

===

Acq. Operator : 系統 Seq. Line : 7
Sample Operator : 系統
Acq. Instrument : 1200 Location : 3
Injection Date : 12/13/2021 6:15:00 PM Inj : 1
Inj Volume : 10.000 µl
Acq. Method : D:\LC\DATA\20211213\20211213A 2021-12-13 14-36-47\ADH-85-15-1ML-10uL-10MIN-210nm.M
Last changed : 9/19/2021 9:53:09 PM by 系統
Analysis Method : D:\LC\DATA\20211213\20211213A 2021-12-13 14-36-47\ADH-85-15-1ML-10uL-10MIN-210nm.M (Sequence Method)
Last changed : 5/11/2022 10:33:24 AM by 系統
(modified after loading)
Additional Info : Peak(s) manually integrated

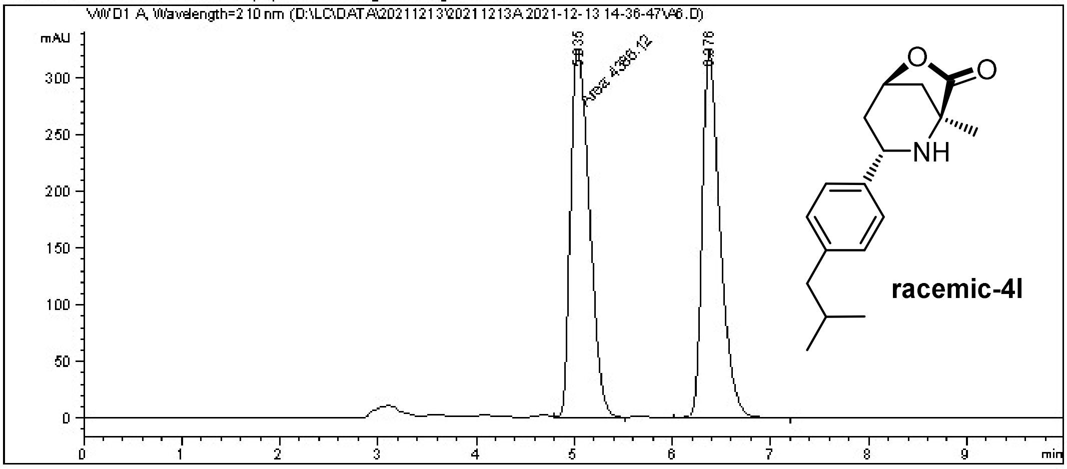

===

Area Percent Report

===

Sorted By : Signal
Multiplier : 1.0000
Dilution : 1.0000
Do not use Multiplier & Dilution Factor with ISTDs

Signal 1: VWD1 A, Wavelength=210 nm

Peak #	RetTime [min]	Type	Width [min]	Area [mAU*s]	Height [mAU]	Area %
1	5.035	MF	0.2247	4386.12451	325.27286	51.4795
2	6.376	BB	0.1889	4134.01709	325.62140	48.5205

Totals : 8520.14160 650.89426

===

*** End of Report ***

1200 5/11/2022 10:33:26 AM 系統 Page 1 of 1

Supplementary Figure 90. HPLC spectrum of racemic-**4l**

Data File D:\LC\DATA\20211210\E 2021-12-10 16-19-34\E12.D
Sample Name: FC03-446-p-iBu-lac-S

===

Acq. Operator : 系統 Seq. Line : 13
Sample Operator : 系統
Acq. Instrument : 1200 Location : 4
Injection Date : 12/10/2021 9:42:23 PM Inj : 1
Inj Volume : 2.000 µl
Acq. Method : D:\LC\DATA\20211210\E 2021-12-10 16-19-34\ADH-85-15-1ML-2uL-20MIN-210nm.M
Last changed : 9/10/2021 8:34:32 PM by 系統
Analysis Method : D:\LC\DATA\20211210\E 2021-12-10 16-19-34\ADH-85-15-1ML-2uL-20MIN-210nm.M (Sequence Method)
Last changed : 5/11/2022 10:28:10 AM by 系統
(modified after loading)
Additional Info : Peak(s) manually integrated

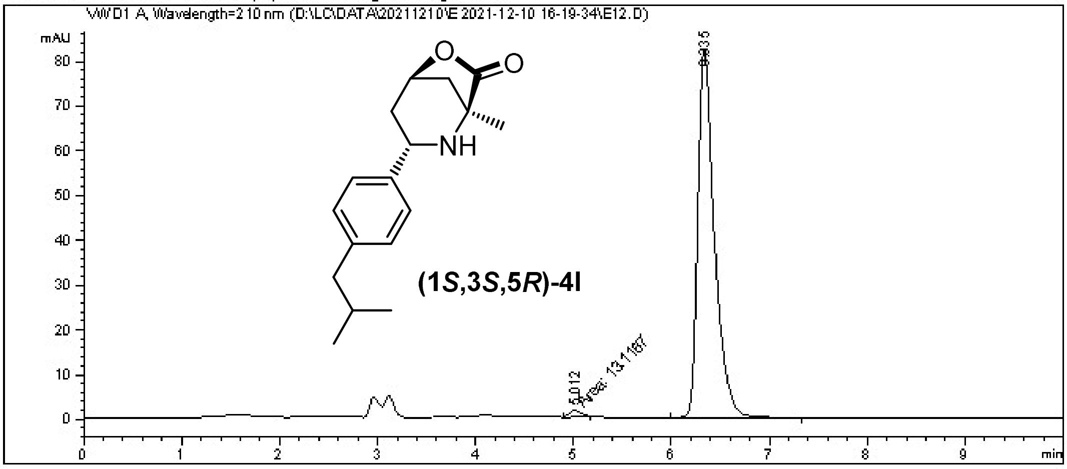

===

Area Percent Report

===

Sorted By : Signal
Multiplier : 1.0000
Dilution : 1.0000
Do not use Multiplier & Dilution Factor with ISTDs

Signal 1: VWD1 A, Wavelength=210 nm

Peak #	RetTime [min]	Type	Width [min]	Area [mAU*s]	Height [mAU]	Area %
1	5.012	MM	0.1410	13.11674	1.55024	1.3263
2	6.335	BB	0.1745	975.85016	82.42980	98.6737

Totals : 988.96690 83.98005

===

*** End of Report ***

1200 5/11/2022 10:30:17 AM 系統 Page 1 of 1

Supplementary Figure 91. HPLC spectrum of (1*S*,3*S*,5*R*)-**4l**

Data File D:\HPLC\Data\20220606\C 2022-06-06 15-31-07\C2.D
Sample Name: FC03-434-mMeO-lac-rac-1-1

===

Acq. Operator : SYSTEM Seq. Line : 3
Sample Operator : SYSTEM
Acq. Instrument : 1260 Location : 12
Injection Date : 6/6/2022 4:17:33 PM Inj : 1
Inj Volume : 2.000 µl
Acq. Method : D:\HPLC\Data\20220606\C 2022-06-06 15-31-07\AD-85-15-210NM-1ML-2uL-20MIN.M
Last changed : 5/23/2022 11:25:12 AM by SYSTEM
Analysis Method : D:\HPLC\Data\20220606\C 2022-06-06 15-31-07\AD-85-15-210NM-1ML-2uL-20MIN.M
(Sequence Method)
Last changed : 6/6/2022 4:58:39 PM by SYSTEM
(modified after loading)
Additional Info : Peak(s) manually integrated

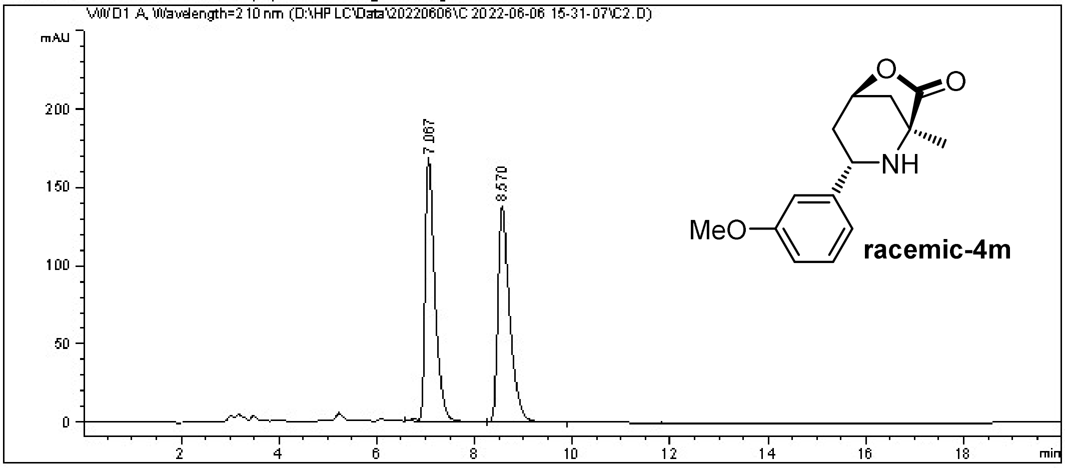

===

Area Percent Report

===

Sorted By : Signal
Multiplier : 1.0000
Dilution : 1.0000
Do not use Multiplier & Dilution Factor with ISTDs

Signal 1: VWD1 A, Wavelength=210 nm

Peak #	RetTime [min]	Type	Width [min]	Area [mAU*s]	Height [mAU]	Area %
1	7.067	VB R	0.2035	2325.77930	168.03516	50.2226
2	8.570	BB	0.2473	2305.16064	137.67819	49.7774

Totals : 4630.93994 305.71335

===

*** End of Report ***

1260 6/6/2022 4:58:42 PM SYSTEM Page 1 of 1

Supplementary Figure 92. HPLC spectrum of racemic-**4m**

Data File D:\HPLC\Data\20220606\A 2022-06-06 10-46-08\A1.D
Sample Name: FC03-434-mMeO-lac-S

===
Acq. Operator : SYSTEM Seq. Line : 2
Sample Operator : SYSTEM
Acq. Instrument : 1260 Location : 13
Injection Date : 6/6/2022 10:57:42 AM Inj : 1
Inj Volume : 2.000 µl
Method : D:\HPLC\Data\20220606\A 2022-06-06 10-46-08\AD-85-15-210NM-1ML-2uL-20MIN.M
(Sequence Method)
Last changed : 5/23/2022 11:25:12 AM by SYSTEM
Additional Info : Peak(s) manually integrated

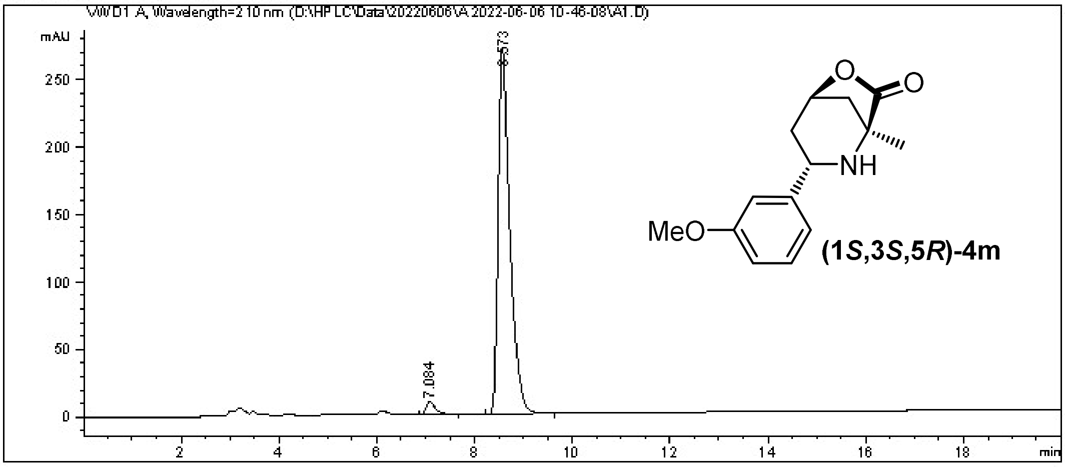

===
Area Percent Report
===

Sorted By : Signal
Multiplier : 1.0000
Dilution : 1.0000
Do not use Multiplier & Dilution Factor with ISTDs

Signal 1: VWD1 A, Wavelength=210 nm

Peak #	RetTime [min]	Type	Width [min]	Area [mAU*s]	Height [mAU]	Area %
1	7.084	BB	0.2006	124.98883	9.19260	2.6745
2	8.573	BB	0.2486	4548.34033	271.30383	97.3255

Totals : 4673.32916 280.49643

===
*** End of Report ***

Supplementary Figure 93. HPLC spectrum of (1*S*,3*S*,5*R*)-**4m**

Data File D:\LC\DATA...-Nap-lactone-rac 2021-11-01 09-00-49\FC03-442-Nap-lactone-rac-021.D
Sample Name: FC03-442-2-Nap-lactone-rac

==
Acq. Operator : 系统 Seq. Line : 2
Sample Operator : 系统
Acq. Instrument : 1200 Location : 1
Injection Date : 11/1/2021 9:12:47 AM Inj : 1
Inj Volume : 2.000 µl
Acq. Method : D:\LC\DATA\FC\FC-1\FC03-442-2-Nap-lactone-rac 2021-11-01 09-00-49\ADH-85-15-1ML-2uL-20MIN-210nm.M
Last changed : 9/10/2021 8:34:32 PM by 系统
Analysis Method : D:\LC\DATA\FC\FC-1\FC03-442-2-Nap-lactone-rac 2021-11-01 09-00-49\ADH-85-15-1ML-2uL-20MIN-210nm.M (Sequence Method)
Last changed : 5/6/2022 10:23:53 AM by 系统
(modified after loading)
Additional Info : Peak(s) manually integrated

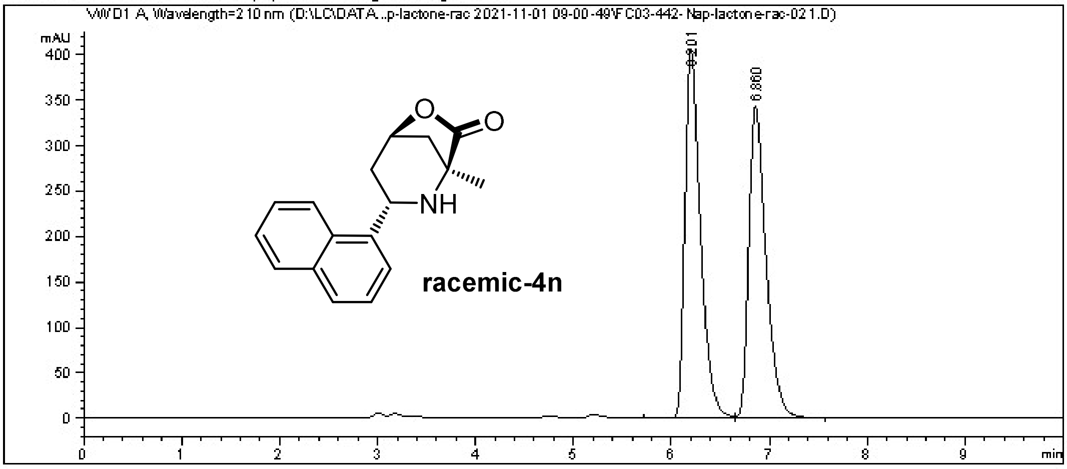

==
Area Percent Report
==

Sorted By : Signal
Multiplier : 1.0000
Dilution : 1.0000
Do not use Multiplier & Dilution Factor with ISTDs

Signal 1: VWD1 A, Wavelength=210 nm

Peak #	RetTime [min]	Type	Width [min]	Area [mAU*s]	Height [mAU]	Area %
1	6.201	VV R	0.1664	4474.07715	406.20853	51.5959
2	6.860	VB	0.1834	4197.30127	343.39914	48.4041

Totals : 8671.37842 749.60767

==
*** End of Report ***

1200 5/6/2022 10:23:55 AM 系统 Page 1 of 1

Supplementary Figure 94. HPLC spectrum of racemic-**4n**

Data File D:\LC\DATA...oMe-lactone-R-S 2021-10-31 19-08-47\FC03-442-Nap-oMe-lactone-R-S3.D
Sample Name: FC03-442-2-Nap-lactone-S

```
=====================================================================
Acq. Operator   : 系統                          Seq. Line :    4
Sample Operator : 系統
Acq. Instrument : 1200                            Location :    2
Injection Date  : 10/31/2021 7:52:51 PM                Inj :    1
                                                Inj Volume : 2.000 µl
Acq. Method     : D:\LC\DATA\FC\FC-1\FC03-442-2-Nap-oMe-lactone-R-S 2021-10-31 19-08-47\ADH-
                  85-15-1ML-2uL-20MIN-210nm.M
Last changed    : 9/10/2021 8:34:32 PM by 系統
Analysis Method : D:\LC\DATA\FC\FC-1\FC03-442-2-Nap-oMe-lactone-R-S 2021-10-31 19-08-47\ADH-
                  85-15-1ML-2uL-20MIN-210nm.M (Sequence Method)
Last changed    : 5/6/2022 10:25:50 AM by 系統
                  (modified after loading)
Additional Info : Peak(s) manually integrated
```

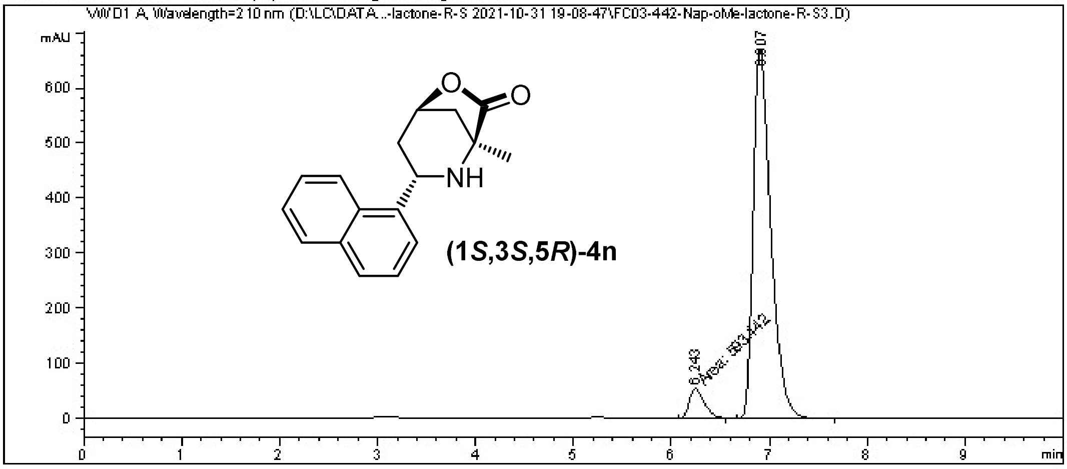


```
=====================================================================
                     Area Percent Report
=====================================================================

Sorted By             :      Signal
Multiplier            :      1.0000
Dilution              :      1.0000
Do not use Multiplier & Dilution Factor with ISTDs


Signal 1: VWD1 A, Wavelength=210 nm
```

Peak #	RetTime [min]	Type	Width [min]	Area [mAU*s]	Height [mAU]	Area %
1	6.243	MF	0.1819	593.44177	54.38683	6.6359
2	6.907	VB	0.1874	8349.42676	670.98920	93.3641

Totals : 8942.86853 725.37602

```
=====================================================================
                     *** End of Report ***
```

1200 5/6/2022 10:25:56 AM 系統 Page 1 of 1

Supplementary Figure 95. HPLC spectrum of (1*S*,3*S*,5*R*)-**4n**

Data File D:\LC\DATA...3-2Nap-lactone-rac 2021-11-11 10-32-23\FC04-453-2Nap-lactone-rac1.D
Sample Name: FC04-453-2Nap-lactone-rac

===

Acq. Operator : 系统 Seq. Line : 2
Sample Operator : 系统
Acq. Instrument : 1200 Location : 2
Injection Date : 11/11/2021 10:44:37 AM Inj : 1
Inj Volume : 2.000 µl
Acq. Method : D:\LC\DATA\FC\FC-1\FC03-453-2Nap-lactone-rac 2021-11-11 10-32-23\ADH-85-15-1ML-2uL-20MIN-210nm.M
Last changed : 9/10/2021 8:34:32 PM by 系统
Analysis Method : D:\LC\DATA\FC\FC-1\FC03-453-2Nap-lactone-rac 2021-11-11 10-32-23\ADH-85-15-1ML-2uL-20MIN-210nm.M (Sequence Method)
Last changed : 5/6/2022 10:41:09 AM by 系统
(modified after loading)
Additional Info : Peak(s) manually integrated

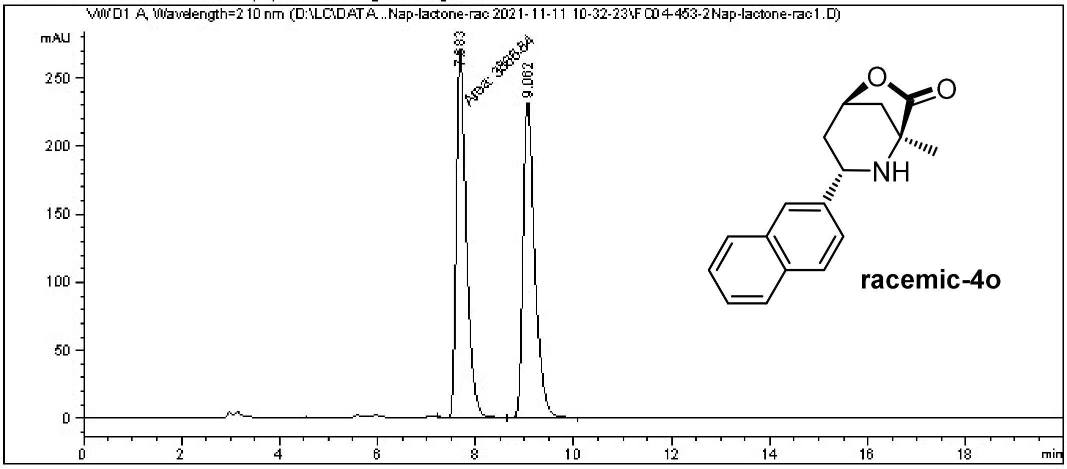

===

Area Percent Report

===

Sorted By : Signal
Multiplier : 1.0000
Dilution : 1.0000
Do not use Multiplier & Dilution Factor with ISTDs

Signal 1: VWD1 A, Wavelength=210 nm

Peak #	RetTime [min]	Type	Width [min]	Area [mAU*s]	Height [mAU]	Area %
1	7.683	FM	0.2379	3866.83740	270.92062	50.0827
2	9.062	BB	0.2492	3854.06323	231.95480	49.9173

Totals : 7720.90063 502.87543

===

*** End of Report ***

1200 5/6/2022 10:41:20 AM 系统 Page 1 of 1

Supplementary Figure 96. HPLC spectrum of racemic-**4o**

Data File D:\LC\DATA...3-2Nap-lactone-R-S 2021-11-10 21-01-48\FC04-453-2Nap-lactone-R-S3.D
Sample Name: FC04-453-2Nap-lactone-S

===

Acq. Operator : 系統 Seq. Line : 4
Sample Operator : 系統
Acq. Instrument : 1200 Location : 36
Injection Date : 11/10/2021 9:46:03 PM Inj : 1
Inj Volume : 2.000 µl
Acq. Method : D:\LC\DATA\FC\FC-1\FC04-453-2Nap-lactone-R-S 2021-11-10 21-01-48\ADH-85-15-1ML-2uL-20MIN-210nm.M
Last changed : 9/10/2021 8:34:32 PM by 系統
Analysis Method : D:\LC\DATA\FC\FC-1\FC04-453-2Nap-lactone-R-S 2021-11-10 21-01-48\ADH-85-15-1ML-2uL-20MIN-210nm.M (Sequence Method)
Last changed : 5/6/2022 10:42:24 AM by 系統
(modified after loading)
Additional Info : Peak(s) manually integrated

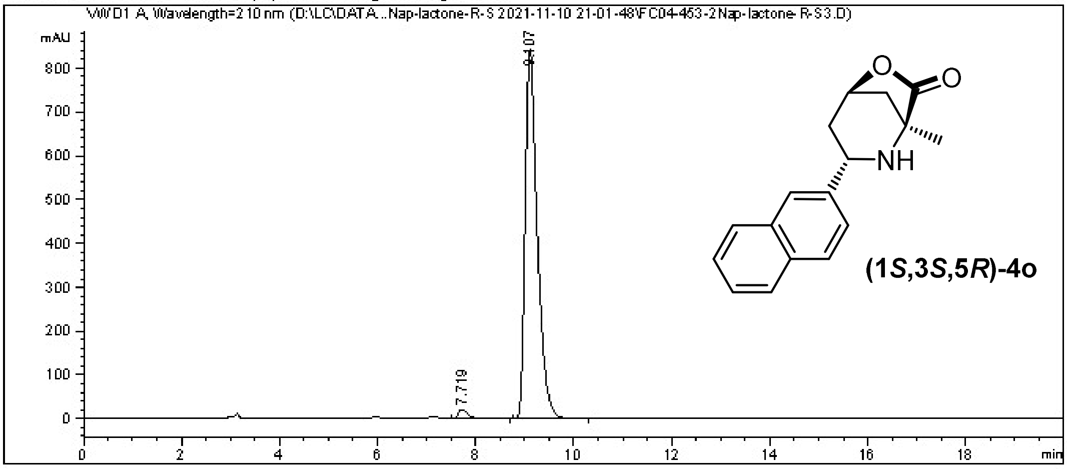

===

Area Percent Report

===

Sorted By : Signal
Multiplier : 1.0000
Dilution : 1.0000
Do not use Multiplier & Dilution Factor with ISTDs

Signal 1: VWD1 A, Wavelength=210 nm

Peak #	RetTime [min]	Type	Width [min]	Area [mAU*s]	Height [mAU]	Area %
1	7.719	VV R	0.2197	312.72479	21.07804	2.1092
2	9.107	BB	0.2588	1.45140e4	844.15302	97.8908

Totals : 1.48267e4 865.23106

===

*** End of Report ***

1200 5/6/2022 10:43:00 AM 系統 Page 1 of 1

Supplementary Figure 97. HPLC spectrum of (1*S*,3*S*,5*R*)-**4o**

Data File D:\LC\DATA...thio-lactone-rac-02 2021-12-02 16-58-10\FC03-439-thio-lactone-rac.D
Sample Name: FC03-439-thio-lactone-rac

===

Acq. Operator : 系统 Seq. Line : 1
Sample Operator : 系统
Acq. Instrument : 1200 Location : 2
Injection Date : 12/2/2021 4:59:08 PM Inj : 1
Inj Volume : 10.000 µl
Acq. Method : D:\LC\DATA\FC\FC-1\FC03-439-thio-lactone-rac-02 2021-12-02 16-58-10\P2-IC-90-10-1ML-5uL-30MIN-210NM-2uL.M
Last changed : 12/2/2021 3:20:46 PM by 系统
Analysis Method : D:\LC\DATA\FC\FC-1\FC03-439-thio-lactone-rac-02 2021-12-02 16-58-10\P2-IC-90-10-1ML-5uL-30MIN-210NM-2uL.M (Sequence Method)
Last changed : 5/6/2022 10:15:36 AM by 系统
(modified after loading)
Additional Info : Peak(s) manually integrated

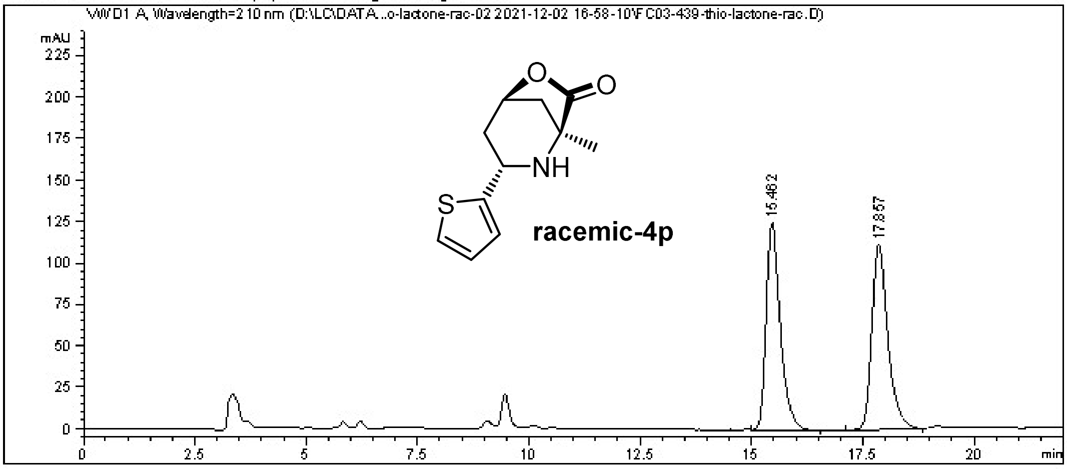

===

Area Percent Report

===

Sorted By : Signal
Multiplier : 1.0000
Dilution : 1.0000
Do not use Multiplier & Dilution Factor with ISTDs

Signal 1: VWD1 A, Wavelength=210 nm

Peak #	RetTime [min]	Type	Width [min]	Area [mAU*s]	Height [mAU]	Area %
1	15.462	VB	0.3343	2775.93018	125.23184	49.7212
2	17.857	BB	0.3808	2807.06421	111.59377	50.2788

Totals : 5582.99438 236.82561

===

*** End of Report ***

1200 5/6/2022 10:15:39 AM 系统 Page 1 of 1

Supplementary Figure 98. HPLC spectrum of racemic-**4p**

Data File D:\LC\DATA...9-thio-lactone-R-S 2021-12-02 15-23-08\FC03-439-thio-lactone-R-S3.D
Sample Name: FC03-439-thio-lactone-S

===

Acq. Operator : 系統 Seq. Line : 4
Sample Operator : 系統
Acq. Instrument : 1200 Location : 3
Injection Date : 12/2/2021 4:16:30 PM Inj : 1
Inj Volume : 10.000 µl
Acq. Method : D:\LC\DATA\FC\FC-1\FC03-439-thio-lactone-R-S 2021-12-02 15-23-08\P2-IC-90-10-1ML-5uL-30MIN-210NM-2uL.M
Last changed : 12/2/2021 3:20:46 PM by 系統
Analysis Method : D:\LC\DATA\FC\FC-1\FC03-439-thio-lactone-R-S 2021-12-02 15-23-08\P2-IC-90-10-1ML-5uL-30MIN-210NM-2uL.M (Sequence Method)
Last changed : 5/6/2022 10:17:32 AM by 系統
(modified after loading)
Additional Info : Peak(s) manually integrated

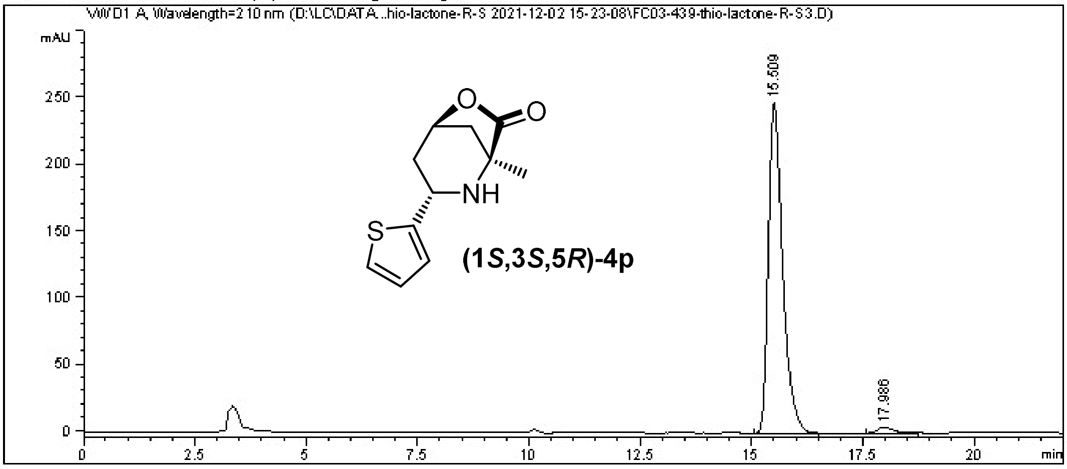

===

Area Percent Report

===

Sorted By : Signal
Multiplier : 1.0000
Dilution : 1.0000
Do not use Multiplier & Dilution Factor with ISTDs

Signal 1: VWD1 A, Wavelength=210 nm

Peak #	RetTime [min]	Type	Width [min]	Area [mAU*s]	Height [mAU]	Area %
1	15.509	BB	0.3392	5551.19580	248.54587	97.9228
2	17.986	BB	0.3499	117.75767	4.61833	2.0772

Totals : 5668.95347 253.16419

===

*** End of Report ***

1200 5/6/2022 10:17:42 AM 系統 Page 1 of 1

Supplementary Figure 99. HPLC spectrum of (1*S*,3*S*,5*R*)-**4p**

Data File D:\LC\DATA...tone-rac-02 2021-10-15 19-50-22\FC03-324-CF3-BrPy-lactone-rac-023.D
Sample Name: FC03-424-BrPy-lactone-rac

===
Acq. Operator : 系统 Seq. Line : 4
Sample Operator : 系统
Acq. Instrument : 1200 Location : 44
Injection Date : 10/15/2021 8:24:47 PM Inj : 1
Inj Volume : 2.000 µl
Acq. Method : D:\LC\DATA\FC\FC-1\FC03-424-CF3-BrPy-lactone-rac-02 2021-10-15 19-50-22\ADH-75-25-1ML-2uL-20MIN-210 nm.M
Last changed : 10/15/2021 3:27:40 PM by 系统
Analysis Method : D:\LC\DATA\FC\FC-1\FC03-424-CF3-BrPy-lactone-rac-02 2021-10-15 19-50-22\ADH-75-25-1ML-2uL-20MIN-210 nm.M (Sequence Method)
Last changed : 5/5/2022 8:22:10 PM by 系统
(modified after loading)
Additional Info : Peak(s) manually integrated

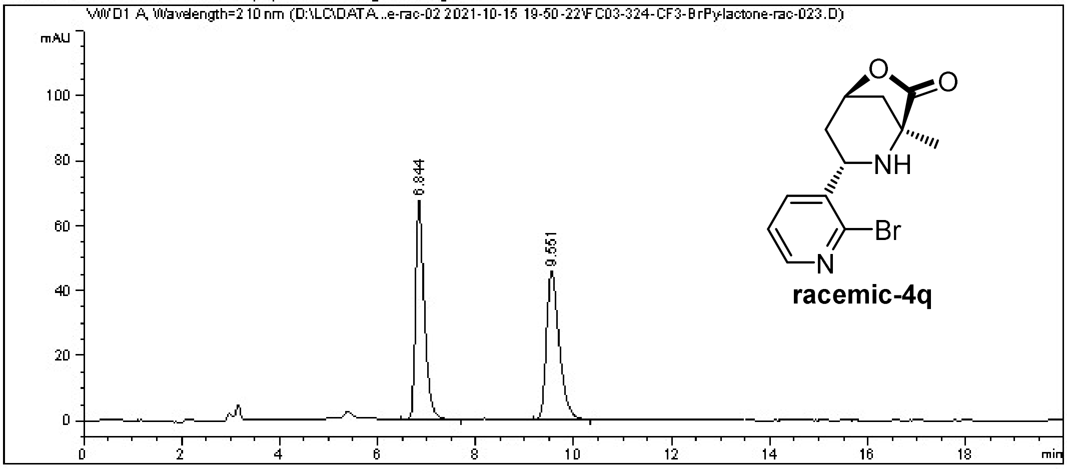

===
Area Percent Report
===

Sorted By : Signal
Multiplier : 1.0000
Dilution : 1.0000
Do not use Multiplier & Dilution Factor with ISTDs

Signal 1: VWD1 A, Wavelength=210 nm

Peak #	RetTime [min]	Type	Width [min]	Area [mAU*s]	Height [mAU]	Area %
1	6.844	VV R	0.1899	860.27710	67.97225	51.0800
2	9.551	BV R	0.2704	823.89856	45.93789	48.9200
Totals :				1684.17566	113.91014	

===
*** End of Report ***

1200 5/5/2022 8:22:12 PM 系统 Page 1 of 1

Supplementary Figure 100. HPLC spectrum of racemic-**4q**

Data File D:\LC\DATA...Py-lactone-R-S 2021-10-15 17-36-29\FC03-324-CF3-BrPy-lactone-R-S7.D
Sample Name: FC03-424-BrPy-lactone-S

===

Acq. Operator : 系统 Seq. Line : 8
Sample Operator : 系统
Acq. Instrument : 1200 Location : 46
Injection Date : 10/15/2021 7:05:20 PM Inj : 1
Inj Volume : 2.000 µl
Acq. Method : D:\LC\DATA\FC\FC-1\FC03-424-CF3-BrPy-lactone-R-S 2021-10-15 17-36-29\ADH-75-25-1ML-2uL-20MIN-210 nm.M
Last changed : 10/15/2021 3:27:40 PM by 系统
Analysis Method : D:\LC\DATA\FC\FC-1\FC03-424-CF3-BrPy-lactone-R-S 2021-10-15 17-36-29\ADH-75-25-1ML-2uL-20MIN-210 nm.M (Sequence Method)
Last changed : 5/5/2022 8:24:03 PM by 系统
(modified after loading)
Additional Info : Peak(s) manually integrated

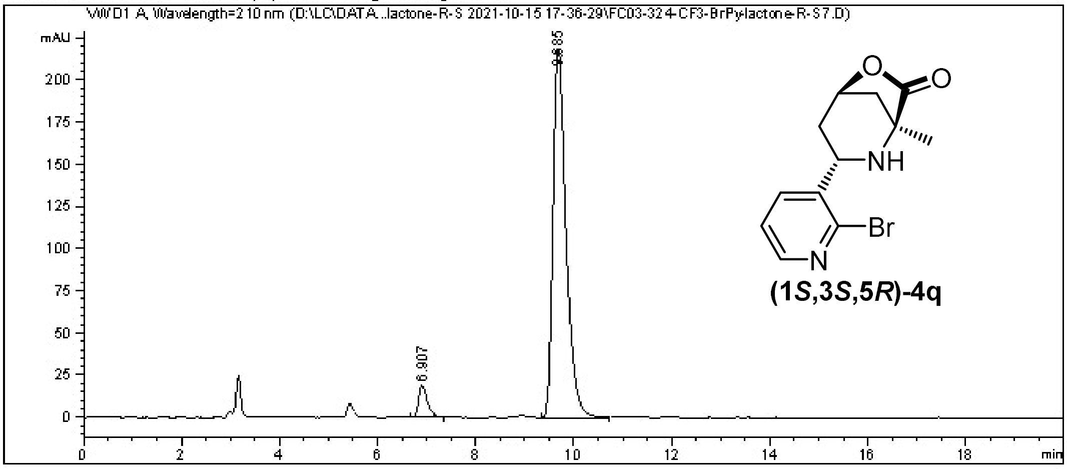

===

Area Percent Report

===

Sorted By : Signal
Multiplier : 1.0000
Dilution : 1.0000
Do not use Multiplier & Dilution Factor with ISTDs

Signal 1: VWD1 A, Wavelength=210 nm

Peak #	RetTime [min]	Type	Width [min]	Area [mAU*s]	Height [mAU]	Area %
1	6.907	BV R	0.1894	239.33774	18.96951	5.6088
2	9.685	BV R	0.2775	4027.87134	218.71835	94.3912

Totals : 4267.20908 237.68787

===

*** End of Report ***

1200 5/5/2022 8:24:38 PM 系统 Page 1 of 1

Supplementary Figure 101. HPLC spectrum of (1*S*,3*S*,5*R*)-**4q**

Data File D:\LC\DATA...-Indol-lactone-rac 2021-11-03 19-36-19\FC03-439-indol-lactone-rac.D
Sample Name: FC03-446-Indol-lactone-rac

===

Acq. Operator : 系统 Seq. Line : 1
Sample Operator : 系统
Acq. Instrument : 1200 Location : 21
Injection Date : 11/3/2021 7:37:51 PM Inj : 1
Inj Volume : 15.000 µl
Acq. Method : D:\LC\DATA\FC\FC-1\FC03-439-Indol-lactone-rac 2021-11-03 19-36-19\ADH-75-25-1ML-15uL-20MIN-210 nm.M
Last changed : 11/3/2021 3:16:22 PM by 系统
Analysis Method : D:\LC\DATA\FC\FC-1\FC03-439-Indol-lactone-rac 2021-11-03 19-36-19\ADH-75-25-1ML-15uL-20MIN-210 nm.M (Sequence Method)
Last changed : 5/6/2022 10:09:09 AM by 系统
(modified after loading)
Additional Info : Peak(s) manually integrated

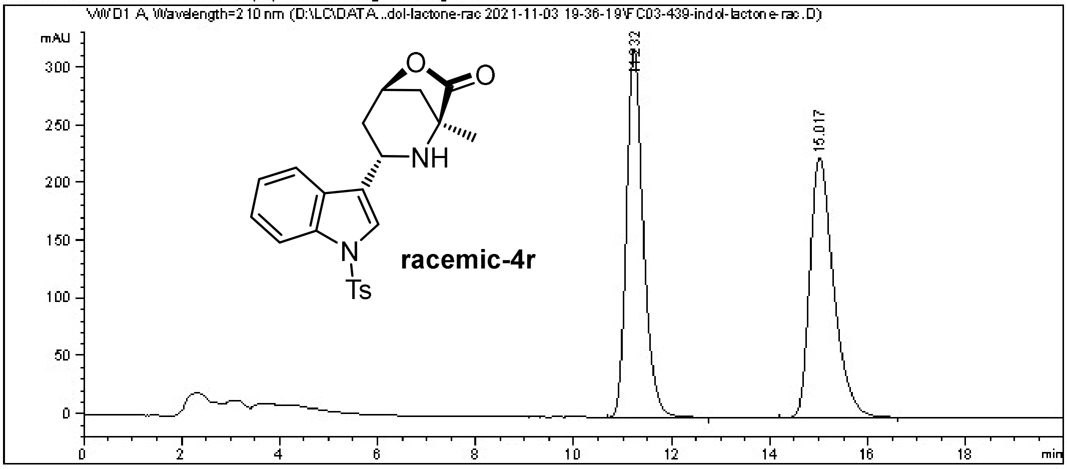

===

Area Percent Report

===

Sorted By : Signal
Multiplier : 1.0000
Dilution : 1.0000
Do not use Multiplier & Dilution Factor with ISTDs

Signal 1: VWD1 A, Wavelength=210 nm

Peak #	RetTime [min]	Type	Width [min]	Area [mAU*s]	Height [mAU]	Area %
1	11.232	BV R	0.3616	7655.76367	318.95007	50.1783
2	15.017	VV R	0.5102	7601.34521	225.23662	49.8217

Totals : 1.52571e4 544.18669

===

*** End of Report ***

1200 5/6/2022 10:09:13 AM 系统 Page 1 of 1

Supplementary Figure 102. HPLC spectrum of racemic-**4r**

Data File D:\LC\DATA...Indol-lactone-R-S 2021-11-03 17-33-53\FC03-439-indol-lactone-R-S3.D
Sample Name: FC03-446-Indol-lactone-S

===

Acq. Operator : 系統 Seq. Line : 4
Sample Operator : 系統
Acq. Instrument : 1200 Location : 22
Injection Date : 11/3/2021 6:18:24 PM Inj : 1
Inj Volume : 15.000 µl
Acq. Method : D:\LC\DATA\FC\FC-1\FC03-439-Indol-lactone-R-S 2021-11-03 17-33-53\ADH-75-25-1ML-15uL-20MIN-210 nm.M
Last changed : 11/3/2021 3:16:22 PM by 系統
Analysis Method : D:\LC\DATA\FC\FC-1\FC03-439-Indol-lactone-R-S 2021-11-03 17-33-53\ADH-75-25-1ML-15uL-20MIN-210 nm.M (Sequence Method)
Last changed : 5/6/2022 10:10:08 AM by 系統
(modified after loading)
Additional Info : Peak(s) manually integrated

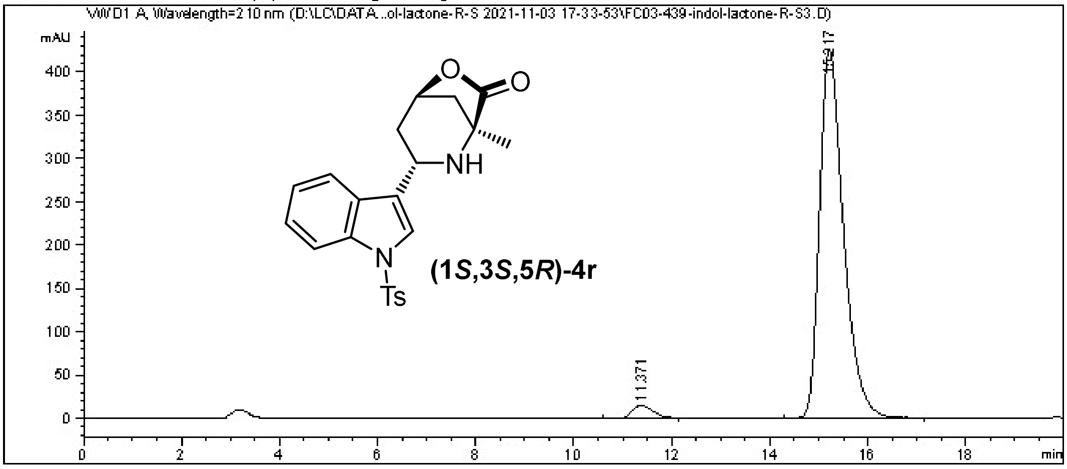

===

Area Percent Report

===

Sorted By : Signal
Multiplier : 1.0000
Dilution : 1.0000
Do not use Multiplier & Dilution Factor with ISTDs

Signal 1: VWD1 A, Wavelength=210 nm

Peak #	RetTime [min]	Type	Width [min]	Area [mAU*s]	Height [mAU]	Area %
1	11.371	VV R	0.3948	418.81448	14.45457	2.8023
2	15.217	BV R	0.5134	1.45264e4	426.96790	97.1977

Totals : 1.49453e4 441.42247

===

*** End of Report ***

1200 5/6/2022 10:10:41 AM 系統 Page 1 of 1

Supplementary Figure 103. HPLC spectrum of (1*S*,3*S*,5*R*)-**4r**

Data File D:\GC\DATA...-LAC-RAC-1-1\FC-CYCLOHEX-LAC-RAC-1-1 2022-04-30 14-49-24\101F0101.D
Sample Name: FC-cyclohex-lac-rac-1-1

===

Acq. Operator : Seq. Line : 1
Acq. Instrument : Instrument 1 Location : Vial 101
Injection Date : 4/30/2022 2:50:39 PM Inj : 1
Inj Volume : 5 µl
Acq. Method : D:\GC\DATA\FC\FC-CYCLOHEX-LAC-RAC-1-1\FC-CYCLOHEX-LAC-RAC-1-1 2022-04-30 14-49-24\FC-300-160-180(0.5)-100MIN-1ML.M
Last changed : 4/29/2022 7:19:00 PM
Analysis Method : D:\GC\METHOD\YZY\BETA-DEX-390-250-150C-20MIN-1ML.M
Last changed : 6/5/2022 7:21:39 PM
(modified after loading)
Additional Info : Peak(s) manually integrated

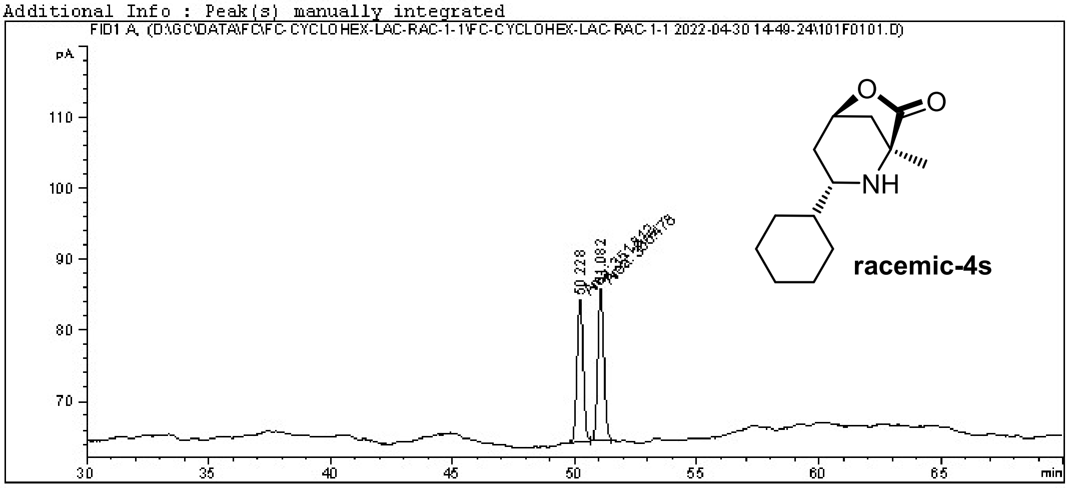

===

Area Percent Report

===

Sorted By : Signal
Multiplier : 1.0000
Dilution : 1.0000
Use Multiplier & Dilution Factor with ISTDs

Signal 1: FID1 A,

Peak #	RetTime [min]	Type	Width [min]	Area [pA*s]	Height [pA]	Area %
1	50.228	MM	0.2924	351.81165	20.05444	48.97911
2	51.082	MM	0.2866	366.47760	21.31496	51.02089
Totals :				718.28925	41.36941	

===

*** End of Report ***

Instrument 1 6/5/2022 7:21:57 PM Page 1 of 1

Supplementary Figure 104. HPLC spectrum of racemic-**4s**

Data File D:\GC\DATA...OHEX-LAC-RR-SS\FC-CYCLOHEX-LAC-RR-SS 2022-04-30 09-09-51\101F0101.D
Sample Name: FC-cyclohex-lac-RR

```
=====================================================================
Acq. Operator   :                                   Seq. Line :   1
Acq. Instrument : Instrument 1                       Location : Vial 101
Injection Date  : 4/30/2022 9:11:29 AM                    Inj :   1
                                                   Inj Volume : 5 µl
Acq. Method     : D:\GC\DATA\FC\FC-CYCLOHEX-LAC-RR-SS\FC-CYCLOHEX-LAC-RR-SS 2022-04-30 09-09-
                  51\FC-300-160-180(0.5)-100MIN-1ML.M
Last changed    : 4/29/2022 7:19:00 PM
Analysis Method : D:\GC\METHOD\YZY\BETA-DEX-390-250-150C-20MIN-1ML.M
Last changed    : 6/5/2022 7:20:27 PM
                  (modified after loading)
Additional Info : Peak(s) manually integrated
```

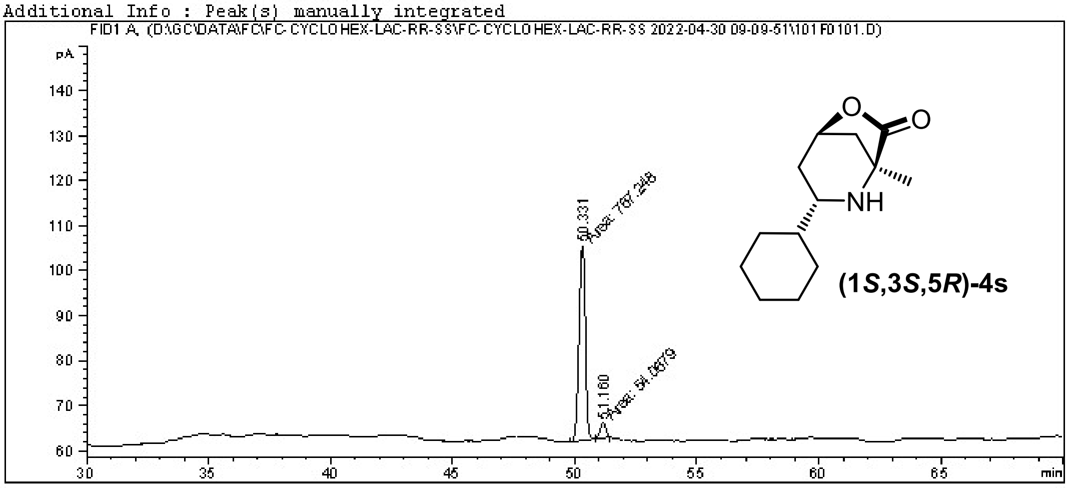


```
=====================================================================
                         Area Percent Report
=====================================================================

Sorted By             :      Signal
Multiplier            :      1.0000
Dilution              :      1.0000
Use Multiplier & Dilution Factor with ISTDs


Signal 1: FID1 A,
```

Peak #	RetTime [min]	Type	Width [min]	Area [pA*s]	Height [pA]	Area %
1	50.331	MM	0.2963	767.24768	43.15296	93.41691
2	51.160	MM	0.2713	54.06791	3.32205	6.58309

Totals : 821.31559 46.47501

```
=====================================================================
                          *** End of Report ***
```


Supplementary Figure 105. HPLC spectrum of (1*S*,3*S*,5*R*)-**4s**

Data File D:\LC\DATA...04-451-Pr-lactone-rac 2021-11-16 20-17-50\FC04-451-Pr-lactone-rac.D
Sample Name: FC04-451-Pr-lactone-rac

===

Acq. Operator : 系統 Seq. Line : 1
Sample Operator : 系統
Acq. Instrument : 1200 Location : 1
Injection Date : 11/16/2021 8:19:13 PM Inj : 1
Inj Volume : 2.000 µl
Acq. Method : D:\LC\DATA\FC\FC-1\FC04-451-Pr-lactone-rac 2021-11-16 20-17-50\ADH-85-15-1ML-2uL-20MIN-210nm.M
Last changed : 9/10/2021 8:34:32 PM by 系統
Analysis Method : D:\LC\DATA\FC\FC-1\FC04-451-Pr-lactone-rac 2021-11-16 20-17-50\ADH-85-15-1ML-2uL-20MIN-210nm.M (Sequence Method)
Last changed : 5/6/2022 11:27:18 AM by 系統
(modified after loading)
Additional Info : Peak(s) manually integrated

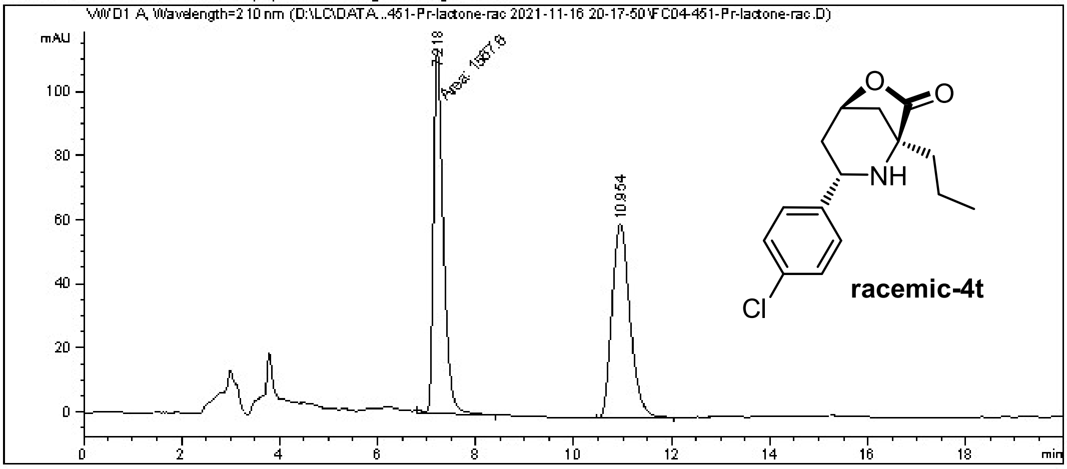

===

Area Percent Report

===

Sorted By : Signal
Multiplier : 1.0000
Dilution : 1.0000
Do not use Multiplier & Dilution Factor with ISTDs

Signal 1: VWD1 A, Wavelength=210 nm

Peak #	RetTime [min]	Type	Width [min]	Area [mAU*s]	Height [mAU]	Area %
1	7.218	FM	0.2299	1567.59705	113.66477	50.2449
2	10.954	BB	0.4025	1552.31726	60.55437	49.7551
Totals :				3119.91431	174.21915	

===

*** End of Report ***

1200 5/6/2022 11:27:20 AM 系統 Page 1 of 1

Supplementary Figure 106. HPLC spectrum of racemic-**4t**

Data File D:\LC\DATA...-EtS-lactone-R-S 2021-11-16 17-40-05\FC04-451-Pr-EtS-lactone-R-S4.D
Sample Name: FC04-451-Pr-lactone-S

===

Acq. Operator	: 系統	Seq. Line :	5
Sample Operator	: 系統		
Acq. Instrument	: 1200	Location :	2
Injection Date	: 11/16/2021 6:45:44 PM	Inj :	1
		Inj Volume :	2.000 µl

Acq. Method : D:\LC\DATA\FC\FC-1\FC04-451-Pr-EtS-lactone-R-S 2021-11-16 17-40-05\ADH-85-15-1ML-2uL-20MIN-210nm.M
Last changed : 9/10/2021 8:34:32 PM by 系統
Analysis Method : D:\LC\DATA\FC\FC-1\FC04-451-Pr-EtS-lactone-R-S 2021-11-16 17-40-05\ADH-85-15-1ML-2uL-20MIN-210nm.M (Sequence Method)
Last changed : 5/6/2022 11:28:18 AM by 系統
(modified after loading)
Additional Info : Peak(s) manually integrated

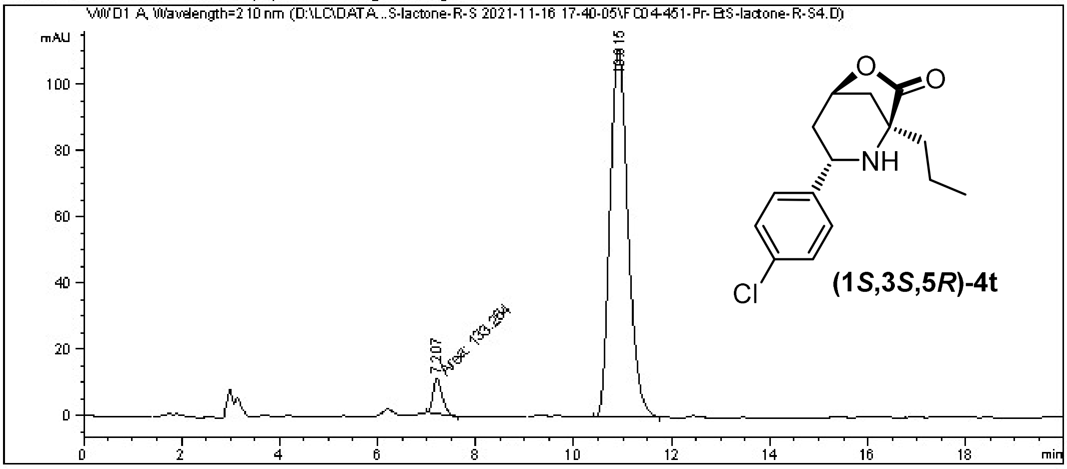

===
Area Percent Report
===

Sorted By	:	Signal
Multiplier	:	1.0000
Dilution	:	1.0000

Do not use Multiplier & Dilution Factor with ISTDs

Signal 1: VWD1 A, Wavelength=210 nm

Peak #	RetTime [min]	Type	Width [min]	Area [mAU*s]	Height [mAU]	Area %
1	7.207	MM	0.2088	133.26350	10.63519	4.4889
2	10.915	BB	0.3994	2835.46899	111.19030	95.5111
Totals :				2968.73250	121.82549	

===
*** End of Report ***

1200 5/6/2022 11:29:04 AM 系統 Page 1 of 1

Supplementary Figure 107. HPLC spectrum of (1*S*,3*S*,5*R*)-**4t**

Data File D:\LC\DATA\20211218\20211218A 2021-12-18 16-28-27\A1.D
Sample Name: FC04-511-a-iBu-lac-rac-1-1

===

Acq. Operator : 系统 Seq. Line : 2
Sample Operator : 系统
Acq. Instrument : 1200 Location : 1
Injection Date : 12/18/2021 4:40:23 PM Inj : 1
Inj Volume : 2.000 µl
Acq. Method : D:\LC\DATA\20211218\20211218A 2021-12-18 16-28-27\ADH-85-15-1ML-2uL-20MIN-210nm.M
Last changed : 12/17/2021 9:35:37 AM by 系统
Analysis Method : D:\LC\DATA\20211218\20211218A 2021-12-18 16-28-27\ADH-85-15-1ML-2uL-20MIN-210nm.M (Sequence Method)
Last changed : 5/11/2022 10:56:12 AM by 系统
(modified after loading)
Additional Info : Peak(s) manually integrated

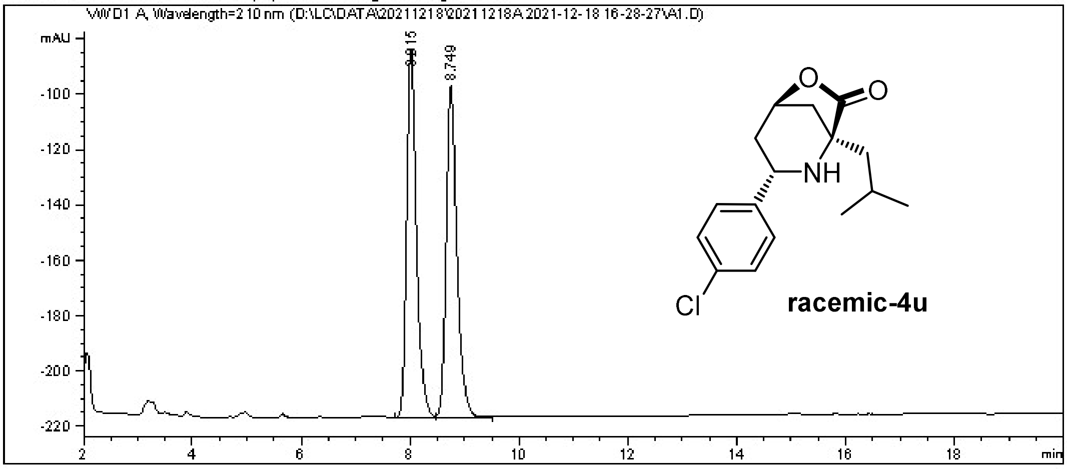

===

Area Percent Report

===

Sorted By : Signal
Multiplier : 1.0000
Dilution : 1.0000
Do not use Multiplier & Dilution Factor with ISTDs

Signal 1: VWD1 A, Wavelength=210 nm

Peak #	RetTime [min]	Type	Width [min]	Area [mAU*s]	Height [mAU]	Area %
1	8.015	BV	0.1871	1655.11743	133.26268	50.0811
2	8.749	VB	0.2085	1649.75696	119.94672	49.9189

Totals : 3304.87439 253.20940

===

*** End of Report ***

1200 5/11/2022 10:56:19 AM 系统 Page 1 of 1

Supplementary Figure 108. HPLC spectrum of racemic-**4u**

Data File D:\LC\DATA\20211217\20211217C 2021-12-17 19-50-54\C8.D
Sample Name: FC04-511-a-iBu-lac-S

===

Acq. Operator : 系统 Seq. Line : 9
Sample Operator : 系统
Acq. Instrument : 1200 Location : 4
Injection Date : 12/17/2021 10:50:00 PM Inj : 1
Inj Volume : 2.000 µl
Acq. Method : D:\LC\DATA\20211217\20211217C 2021-12-17 19-50-54\ADH-85-15-1ML-2uL-20MIN-210nm.M
Last changed : 12/17/2021 9:35:37 AM by 系统
Analysis Method : D:\LC\DATA\20211217\20211217C 2021-12-17 19-50-54\ADH-85-15-1ML-2uL-20MIN-210nm.M (Sequence Method)
Last changed : 5/11/2022 10:49:24 AM by 系统
(modified after loading)
Additional Info : Peak(s) manually integrated

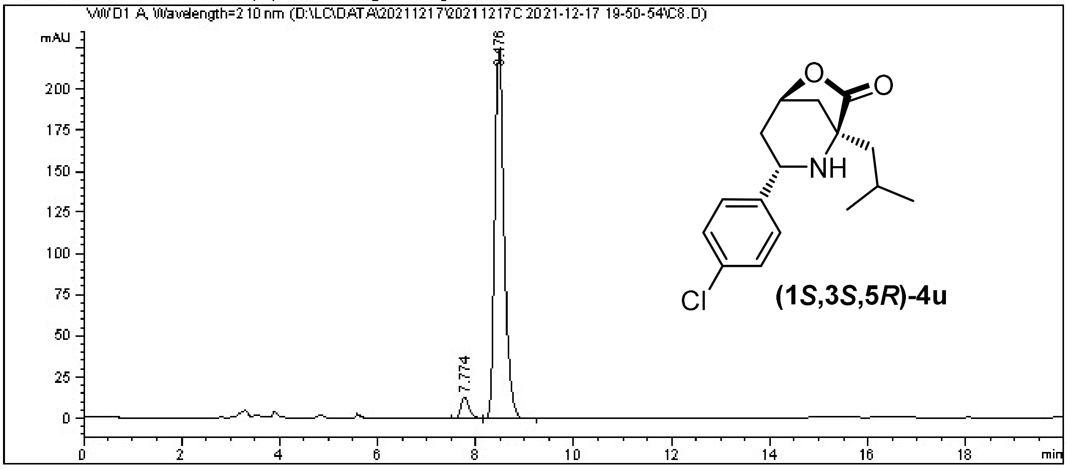

===

Area Percent Report

===

Sorted By : Signal
Multiplier : 1.0000
Dilution : 1.0000
Do not use Multiplier & Dilution Factor with ISTDs

Signal 1: VWD1 A, Wavelength=210 nm

Peak #	RetTime [min]	Type	Width [min]	Area [mAU*s]	Height [mAU]	Area %
1	7.774	BV	0.1811	154.96170	13.01833	4.9191
2	8.476	VB	0.2006	2995.24023	224.83531	95.0809

Totals : 3150.20193 237.85365

===

*** End of Report ***

1200 5/11/2022 10:51:31 AM 系统 Page 1 of 1

Supplementary Figure 109. HPLC spectrum of (1*S*,3*S*,5*R*)-**4u**

Data File D:\HPLC\Data\20220530\D 2022-05-30 21-50-22\D5.D
Sample Name: FC03-406-Bn-lac-rac-1-1

===

Acq. Operator : SYSTEM   Seq. Line : 6
Sample Operator : SYSTEM
Acq. Instrument : 1260   Location : 14
Injection Date : 5/30/2022 11:10:31 PM   Inj : 1
Inj Volume : 2.000 µl
Method : D:\HPLC\Data\20220530\D 2022-05-30 21-50-22\AD-85-15-210NM-1ML-2uL-20MIN.M (Sequence Method)
Last changed : 5/23/2022 11:25:12 AM by SYSTEM
Additional Info : Peak(s) manually integrated

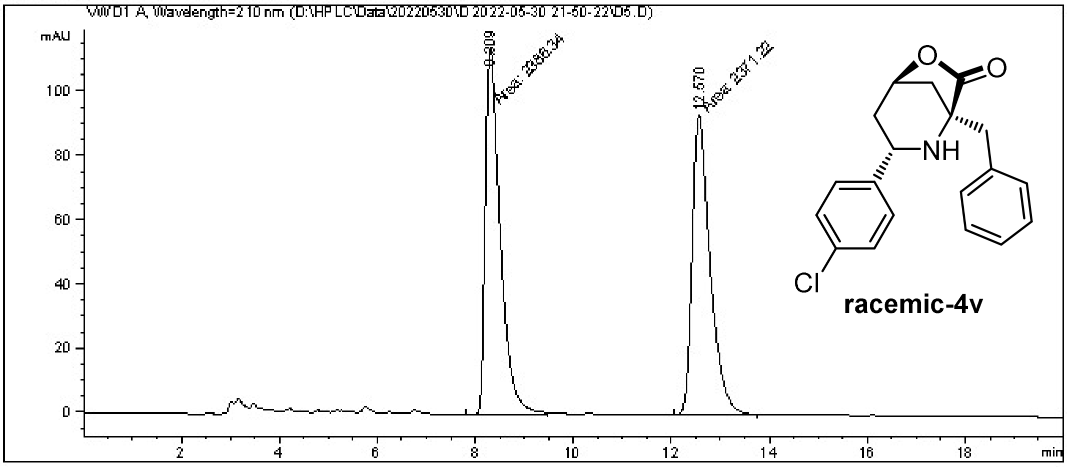

===

Area Percent Report

===

Sorted By : Signal
Multiplier : 1.0000
Dilution : 1.0000
Do not use Multiplier & Dilution Factor with ISTDs

Signal 1: VWD1 A, Wavelength=210 nm

Peak #	RetTime [min]	Type	Width [min]	Area [mAU*s]	Height [mAU]	Area %
1	8.309	MF	0.3488	2386.34180	114.01543	50.1589
2	12.570	MF	0.4243	2371.22241	93.13216	49.8411
Totals :				4757.56421	207.14759	

===

*** End of Report ***

1260 6/2/2022 11:31:31 AM SYSTEM   Page 1 of 1

Supplementary Figure 110. HPLC spectrum of racemic-**4v**

Data File D:\LC\DATA...-40o-S-04 2021-09-22 16-06-06\FC03-395-a-Bn-lactone-MeCN-40o-S-04.D
Sample Name: FC03-395-a-Bn-lactone-MeCN-40o-S-03

===

Acq. Operator : 系統 Seq. Line : 1
Sample Operator : 系統
Acq. Instrument : 1200 Location : 2
Injection Date : 9/22/2021 4:07:18 PM Inj : 1
Inj Volume : 2.000 µl
Acq. Method : D:\LC\DATA\FC\FC03-395-a-Bn-lactone-MeCN-40o-S-04 2021-09-22 16-06-06\ADH-85-15-1ML-2uL-20MIN-210nm.M
Last changed : 9/10/2021 8:34:32 PM by 系統
Analysis Method : D:\LC\DATA\FC\FC03-395-a-Bn-lactone-MeCN-40o-S-04 2021-09-22 16-06-06\ADH-85-15-1ML-2uL-20MIN-210nm.M (Sequence Method)
Last changed : 6/2/2022 11:40:02 AM by 系統
(modified after loading)
Additional Info : Peak(s) manually integrated

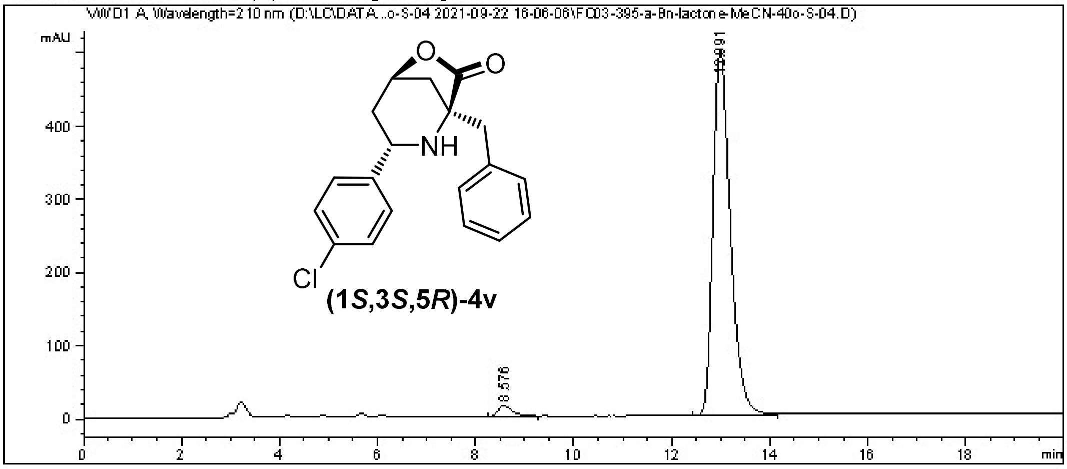

===

Area Percent Report

===

Sorted By : Signal
Multiplier : 1.0000
Dilution : 1.0000
Do not use Multiplier & Dilution Factor with ISTDs

Signal 1: VWD1 A, Wavelength=210 nm

Peak #	RetTime [min]	Type	Width [min]	Area [mAU*s]	Height [mAU]	Area %
1	8.576	BV R	0.3009	286.30438	14.45629	2.2783
2	12.991	BB	0.3765	1.22801e4	500.57867	97.7217

Totals : 1.25664e4 515.03496

===

*** End of Report ***

1200 6/2/2022 11:40:04 AM 系統 Page 1 of 1

Supplementary Figure 111. HPLC spectrum of (1*S*,3*S*,5*R*)-**4v**

```
Data File D:\LC\DATA\20211218\20211218A 2021-12-18 16-28-27\A2.D
Sample Name: FC04-511-a-Ph-lac-rac-1-1

=====================================================================
Acq. Operator   : 系统                         Seq. Line :   3
Sample Operator : 系统
Acq. Instrument : 1200                          Location :   2
Injection Date  : 12/18/2021 5:01:34 PM              Inj :   1
                                              Inj Volume : 2.000 µl
Acq. Method     : D:\LC\DATA\20211218\20211218A 2021-12-18 16-28-27\ADH-85-15-1ML-2uL-20MIN-
                  210nm.M
Last changed    : 12/17/2021 9:35:37 AM by 系统
Analysis Method : D:\LC\DATA\20211218\20211218A 2021-12-18 16-28-27\ADH-85-15-1ML-2uL-20MIN-
                  210nm.M (Sequence Method)
Last changed    : 5/11/2022 11:00:25 AM by 系统
                  (modified after loading)
Additional Info : Peak(s) manually integrated
```

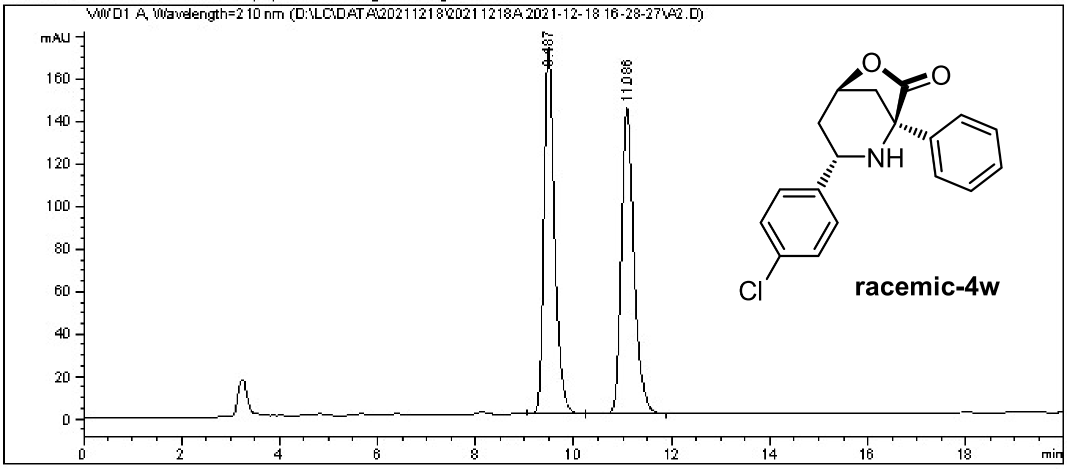


```
=====================================================================
                         Area Percent Report
=====================================================================

Sorted By             :      Signal
Multiplier            :      1.0000
Dilution              :      1.0000
Do not use Multiplier & Dilution Factor with ISTDs


Signal 1: VWD1 A, Wavelength=210 nm

Peak RetTime Type  Width     Area      Height     Area
  #   [min]        [min]   [mAU*s]     [mAU]        %
----|-------|----|-------|----------|----------|--------|
   1   9.487 BB    0.2284 2588.84546  171.66862  50.0967
   2  11.086 VB R  0.2716 2578.84717  143.96605  49.9033

Totals :                  5167.69263  315.63467

=====================================================================
                         *** End of Report ***
```

1200 5/11/2022 11:00:32 AM 系统 Page 1 of 1

Supplementary Figure 112. HPLC spectrum of racemic-**4w**

Data File D:\LC\DATA\20211217\20211217C 2021-12-17 19-50-54\C7.D
Sample Name: FC04-511-a-Ph-lac-S

===
Acq. Operator : 系統 Seq. Line : 8
Sample Operator : 系統
Acq. Instrument : 1200 Location : 2
Injection Date : 12/17/2021 10:28:50 PM Inj : 1
Inj Volume : 2.000 µl
Acq. Method : D:\LC\DATA\20211217\20211217C 2021-12-17 19-50-54\ADH-85-15-1ML-2uL-20MIN-210nm.M
Last changed : 12/17/2021 9:35:37 AM by 系統
Analysis Method : D:\LC\DATA\20211217\20211217C 2021-12-17 19-50-54\ADH-85-15-1ML-2uL-20MIN-210nm.M (Sequence Method)
Last changed : 5/11/2022 10:49:24 AM by 系統
(modified after loading)
Additional Info : Peak(s) manually integrated

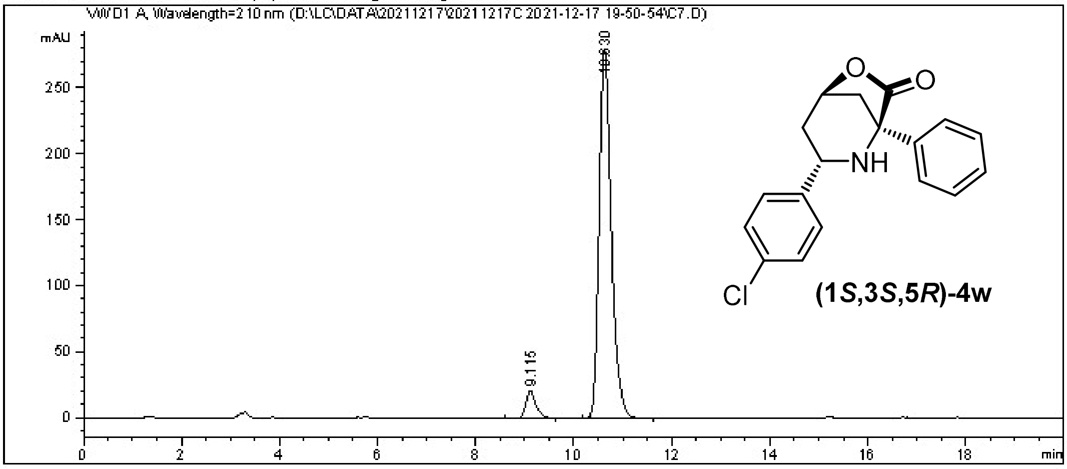

===
Area Percent Report
===

Sorted By : Signal
Multiplier : 1.0000
Dilution : 1.0000
Do not use Multiplier & Dilution Factor with ISTDs

Signal 1: VWD1 A, Wavelength=210 nm

Peak #	RetTime [min]	Type	Width [min]	Area [mAU*s]	Height [mAU]	Area %
1	9.115	BB	0.2252	312.43976	20.91565	6.0754
2	10.630	BB	0.2624	4830.29590	279.97153	93.9246

Totals : 5142.73566 300.88717

===
*** End of Report ***

1200 5/11/2022 10:50:11 AM 系統 Page 1 of 1

Supplementary Figure 113. HPLC spectrum of (1*S*,3*S*,5*R*)-**4w**

Data File D:\LC\DATA...e-rac 2021-11-25 15-00-18\FC04-459-Ph-CH2CH2COOMe-lactone-rac-023.D
Sample Name: CH2CH2COOMe-lactone-rac

===

Acq. Operator : 系统 Seq. Line : 4
Sample Operator : 系统
Acq. Instrument : 1200 Location : 2
Injection Date : 11/25/2021 3:44:32 PM Inj : 1
Inj Volume : 2.000 µl
Acq. Method : D:\LC\DATA\FC\FC-1\FC04-459-Ph-CH2CH2COOMe-lactone-rac 2021-11-25 15-00-18
\ADH-75-25-1ML-2uL-20MIN-210 nm.M
Last changed : 10/15/2021 3:27:40 PM by 系统
Analysis Method : D:\LC\DATA\FC\FC-1\FC04-459-Ph-CH2CH2COOMe-lactone-rac 2021-11-25 15-00-18
\ADH-75-25-1ML-2uL-20MIN-210 nm.M (Sequence Method)
Last changed : 5/6/2022 11:30:30 AM by 系统
(modified after loading)
Additional Info : Peak(s) manually integrated

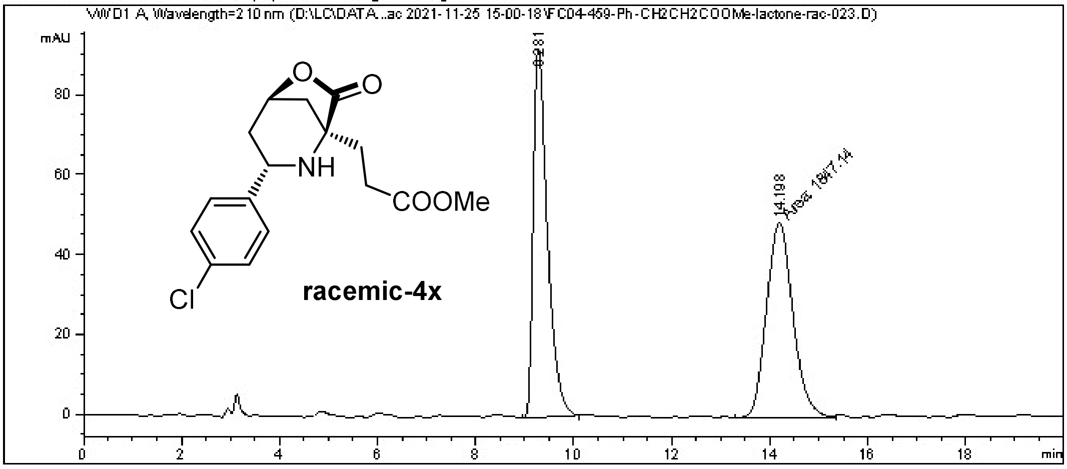

===

Area Percent Report

===

Sorted By : Signal
Multiplier : 1.0000
Dilution : 1.0000
Do not use Multiplier & Dilution Factor with ISTDs

Signal 1: VWD1 A, Wavelength=210 nm

Peak #	RetTime [min]	Type	Width [min]	Area [mAU*s]	Height [mAU]	Area %
1	9.281	BB	0.3001	1853.59143	92.19229	50.0871
2	14.198	MF	0.6287	1847.14246	48.96716	49.9129

Totals : 3700.73389 141.15945

===

*** End of Report ***

1200 5/6/2022 11:30:34 AM 系统 Page 1 of 1

Supplementary Figure 114. HPLC spectrum of racemic-**4x**

```
Data File D:\LC\DATA...-R-S 2021-11-25 11-30-57\FC04-459-Ph-CH2CH2COOMe-pMePh-lactone-R9.D
Sample Name: CH2CH2COOMe-lactone-S

=====================================================================
Acq. Operator   : 系統                          Seq. Line :  10
Sample Operator : 系統
Acq. Instrument : 1200                            Location :   5
Injection Date  : 11/25/2021 1:51:26 PM                Inj :   1
                                                Inj Volume : 2.000 µl
Acq. Method     : D:\LC\DATA\FC\FC-1\FC04-459-Ph-CH2CH2COOMe-pMePh-lactone-R-S 2021-11-25 11-
                  30-57\ADH-75-25-1ML-2uL-20MIN-210 nm.M
Last changed    : 10/15/2021 3:27:40 PM by 系統
Analysis Method : D:\LC\DATA\FC\FC-1\FC04-459-Ph-CH2CH2COOMe-pMePh-lactone-R-S 2021-11-25 11-
                  30-57\ADH-75-25-1ML-2uL-20MIN-210 nm.M (Sequence Method)
Last changed    : 5/6/2022 11:34:26 AM by 系統
                  (modified after loading)
Additional Info : Peak(s) manually integrated
```

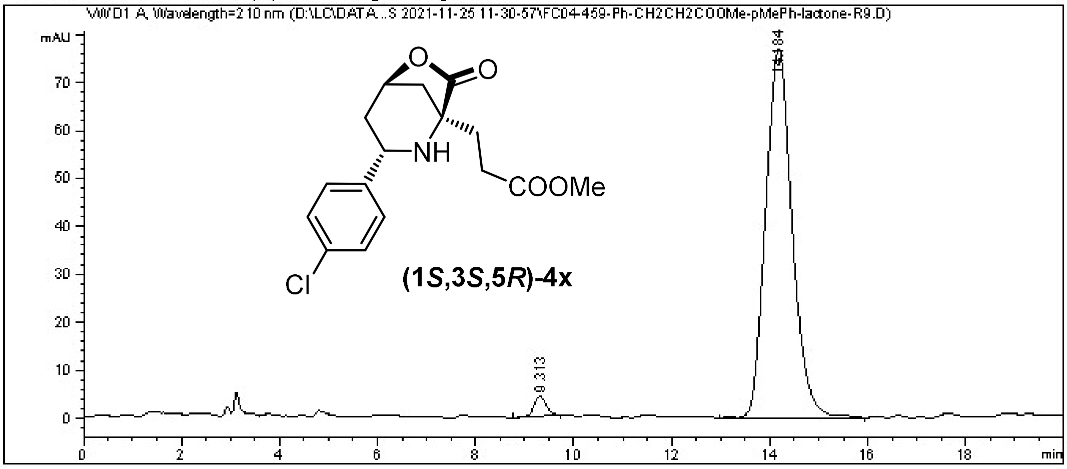


```
=====================================================================
                          Area Percent Report
=====================================================================

Sorted By             :      Signal
Multiplier            :      1.0000
Dilution              :      1.0000
Do not use Multiplier & Dilution Factor with ISTDs


Signal 1: VWD1 A, Wavelength=210 nm

Peak RetTime Type  Width     Area      Height     Area
  #   [min]        [min]   [mAU*s]     [mAU]        %
----|-------|----|-------|----------|----------|--------|
   1   9.313 BB    0.2525   73.79980    4.12354   2.4801
   2  14.184 VV R  0.5855 2901.87598   76.96887  97.5199

Totals :                  2975.67577   81.09241


=====================================================================
                          *** End of Report ***
```

1200 5/6/2022 11:35:14 AM 系統 Page 1 of 1

Supplementary Figure 115. HPLC spectrum of (1*S*,3*S*,5*R*)-**4x**

```
Data File D:\LC\DATA...S-lactone-rac-02 2021-11-17 15-42-12\FC04-451-EtS-lactone-rac-021.D
Sample Name: FC04-451-EtS-lactone-rac

    =====================================================================
    Acq. Operator   : 系統                          Seq. Line :   2
    Sample Operator : 系統
    Acq. Instrument : 1200                            Location :   2
    Injection Date  : 11/17/2021 3:54:26 PM                Inj :   1
                                                    Inj Volume : 2.000 µl
    Acq. Method     : D:\LC\DATA\FC\FC-1\FC04-451-EtS-lactone-rac-02 2021-11-17 15-42-12\ADH-85-
                      15-1ML-2uL-20MIN-210nm.M
    Last changed    : 9/10/2021 8:34:32 PM by 系統
    Analysis Method : D:\LC\DATA\FC\FC-1\FC04-451-EtS-lactone-rac-02 2021-11-17 15-42-12\ADH-85-
                      15-1ML-2uL-20MIN-210nm.M (Sequence Method)
    Last changed    : 5/6/2022 11:06:52 AM by 系統
                      (modified after loading)
    Additional Info : Peak(s) manually integrated
```

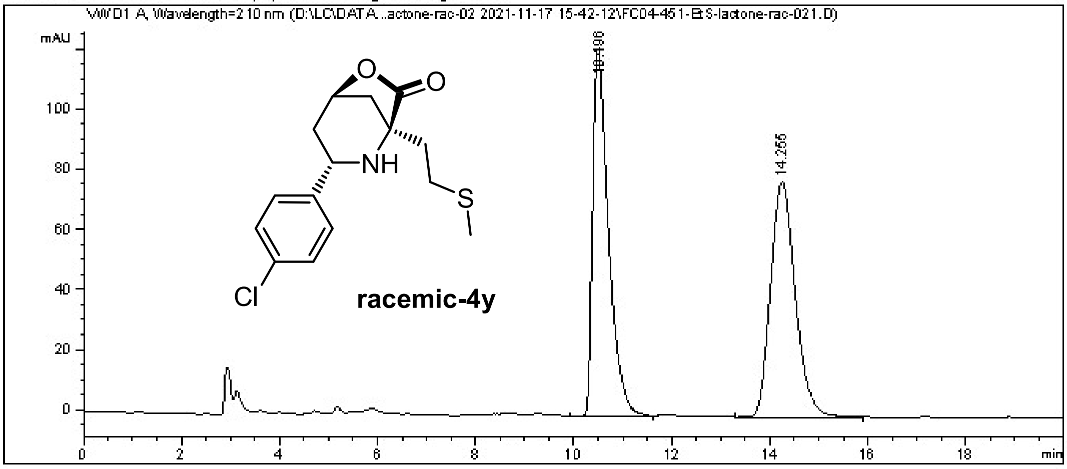


```
    =====================================================================
                         Area Percent Report
    =====================================================================

    Sorted By             :      Signal
    Multiplier            :      1.0000
    Dilution              :      1.0000
    Do not use Multiplier & Dilution Factor with ISTDs


    Signal 1: VWD1 A, Wavelength=210 nm
```

Peak #	RetTime [min]	Type	Width [min]	Area [mAU*s]	Height [mAU]	Area %
1	10.496	BV R	0.3355	2731.52466	121.98933	49.8591
2	14.255	VB R	0.5501	2746.96387	78.38134	50.1409

```
    Totals :                  5478.48853  200.37067


    =====================================================================
                          *** End of Report ***
```

1200 5/6/2022 11:06:55 AM 系統 Page 1 of 1

Supplementary Figure 116. HPLC spectrum of racemic-**4y**

Data File D:\LC\DATA...S-lactone-R-S-02 2021-11-16 21-23-07\FC04-451-EtS-lactone-R-S-022.D
Sample Name: FC04-451-EtS-lactone-S

===

Acq. Operator : 系統 Seq. Line : 3
Sample Operator : 系統
Acq. Instrument : 1200 Location : 3
Injection Date : 11/16/2021 9:56:36 PM Inj : 1
Inj Volume : 2.000 µl
Acq. Method : D:\LC\DATA\FC\FC-1\FC04-451-EtS-lactone-R-S-02 2021-11-16 21-23-07\ADH-85-15-1ML-2uL-20MIN-210nm.M
Last changed : 9/10/2021 8:34:32 PM by 系統
Analysis Method : D:\LC\DATA\FC\FC-1\FC04-451-EtS-lactone-R-S-02 2021-11-16 21-23-07\ADH-85-15-1ML-2uL-20MIN-210nm.M (Sequence Method)
Last changed : 5/6/2022 11:08:55 AM by 系統
(modified after loading)
Additional Info : Peak(s) manually integrated

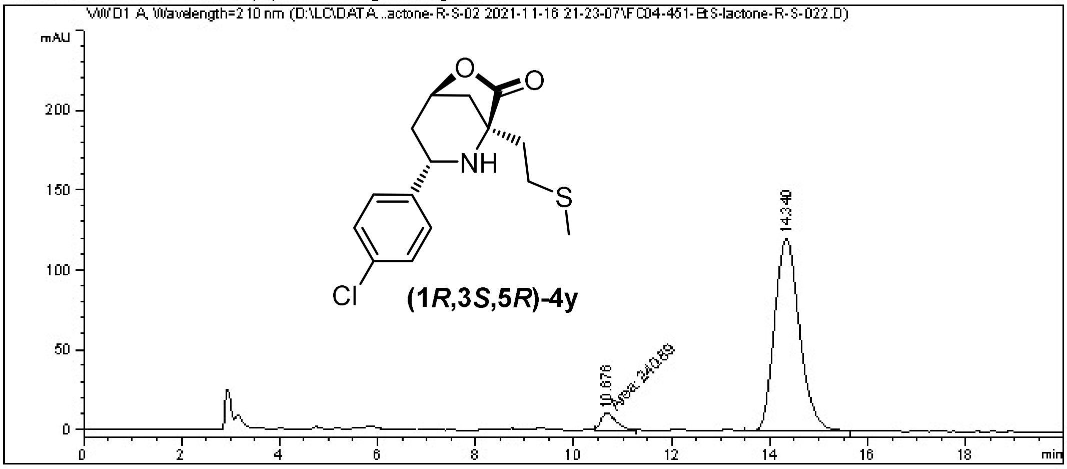

===

Area Percent Report

===

Sorted By : Signal
Multiplier : 1.0000
Dilution : 1.0000
Do not use Multiplier & Dilution Factor with ISTDs

Signal 1: VWD1 A, Wavelength=210 nm

Peak #	RetTime [min]	Type	Width [min]	Area [mAU*s]	Height [mAU]	Area %
1	10.676	MF	0.3643	240.89014	11.02041	5.3656
2	14.340	BB	0.5476	4248.66553	120.67815	94.6344

Totals : 4489.55566 131.69855

===

*** End of Report ***

1200 5/6/2022 11:08:58 AM 系統 Page 1 of 1

Supplementary Figure 117. HPLC spectrum of (1*R*,3*S*,5*R*)-**4y**

Data File D:\LC\DATA\20220310\C 2022-03-10 16-33-37\C1.D
Sample Name: FC04-593-LAH-diol-rac-1-1

===

Acq. Operator : 系统 Seq. Line : 2
Sample Operator : 系统
Acq. Instrument : 1200 Location : 1
Injection Date : 3/10/2022 4:45:33 PM Inj : 1
Inj Volume : 2.000 µl
Acq. Method : D:\LC\DATA\20220310\C 2022-03-10 16-33-37\ADH-75-25-1ML-2uL-10MIN-210 nm.M
Last changed : 10/15/2021 3:26:38 PM by 系统
Analysis Method : D:\LC\DATA\20220310\C 2022-03-10 16-33-37\ADH-75-25-1ML-2uL-10MIN-210 nm.M
(Sequence Method)
Last changed : 5/11/2022 11:26:48 AM by 系统
(modified after loading)
Additional Info : Peak(s) manually integrated

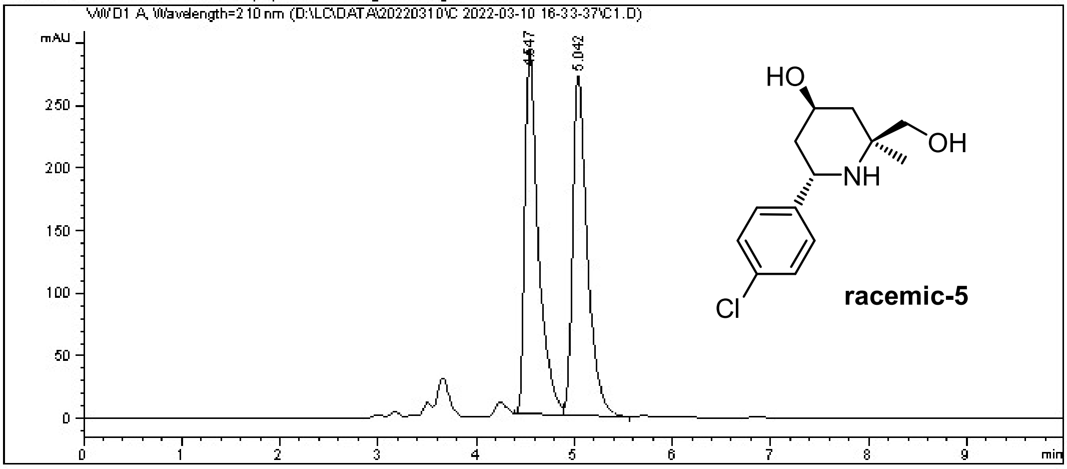

===

Area Percent Report

===

Sorted By : Signal
Multiplier : 1.0000
Dilution : 1.0000
Do not use Multiplier & Dilution Factor with ISTDs

Signal 1: VWD1 A, Wavelength=210 nm

Peak #	RetTime [min]	Type	Width [min]	Area [mAU*s]	Height [mAU]	Area %
1	4.547	BV	0.1411	2798.88037	291.69766	49.8242
2	5.042	VB	0.1530	2818.63550	271.74243	50.1758

Totals : 5617.51587 563.44009

===

*** End of Report ***

1200 5/11/2022 11:26:50 AM 系统 Page 1 of 1

Supplementary Figure 118. HPLC spectrum of racemic-**5**

Data File D:\LC\DATA\20220308\E 2022-03-08 19-22-24\E2.D
Sample Name: FC04-593-LAH-diol-S

===

Acq. Operator : 系統 Seq. Line : 3
Sample Operator : 系統
Acq. Instrument : 1200 Location : 2
Injection Date : 3/8/2022 7:47:23 PM Inj : 1
Inj Volume : 2.000 µl
Acq. Method : D:\LC\DATA\20220308\E 2022-03-08 19-22-24\ADH-75-25-1ML-2uL-10MIN-210 nm.M
Last changed : 10/15/2021 3:26:38 PM by 系統
Analysis Method : D:\LC\DATA\20220308\E 2022-03-08 19-22-24\ADH-75-25-1ML-2uL-10MIN-210 nm.M
(Sequence Method)
Last changed : 5/11/2022 11:25:09 AM by 系統
(modified after loading)
Additional Info : Peak(s) manually integrated

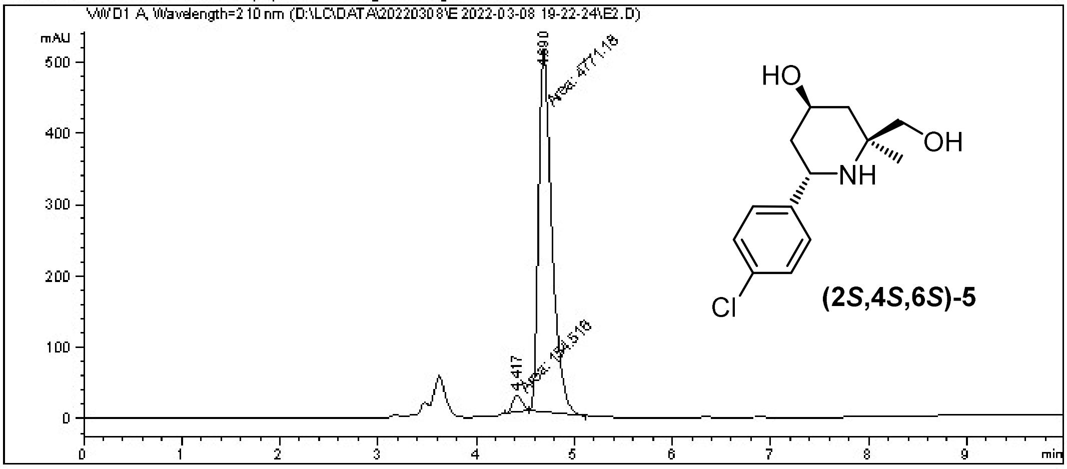

===

Area Percent Report

===

Sorted By : Signal
Multiplier : 1.0000
Dilution : 1.0000
Do not use Multiplier & Dilution Factor with ISTDs

Signal 1: VWD1 A, Wavelength=210 nm

Peak #	RetTime [min]	Type	Width [min]	Area [mAU*s]	Height [mAU]	Area %
1	4.417	MM	0.1120	154.51587	23.00130	3.1369
2	4.690	MM	0.1564	4771.18066	508.39407	96.8631

Totals : 4925.69653 531.39537

===

*** End of Report ***

1200 5/11/2022 11:25:41 AM 系統 Page 1 of 1

Supplementary Figure 119. HPLC spectrum of (2*S*,4*S*,6*S*)-**5**

```
Data File D:\LC\DATA\20220228\D 2022-02-28 21-34-06\D1.D
Sample Name: FC04-578-HEW-rac-1-1

    =====================================================================
    Acq. Operator   : 系統                          Seq. Line :   2
    Sample Operator : 系統
    Acq. Instrument : 1200                            Location :   31
    Injection Date  : 2/28/2022 9:38:48 PM                  Inj :   1
                                                  Inj Volume : 2.000 µl
    Acq. Method     : D:\LC\DATA\20220228\D 2022-02-28 21-34-06\P2-IC-90-10-1ML-20MIN-210NM-2uL.M
    Last changed    : 2/15/2022 11:03:17 AM by 系統
    Analysis Method : D:\LC\DATA\20220228\D 2022-02-28 21-34-06\P2-IC-90-10-1ML-20MIN-210NM-2uL.M
                      (Sequence Method)
    Last changed    : 5/11/2022 11:20:31 AM by 系統
                      (modified after loading)
    Additional Info : Peak(s) manually integrated
```

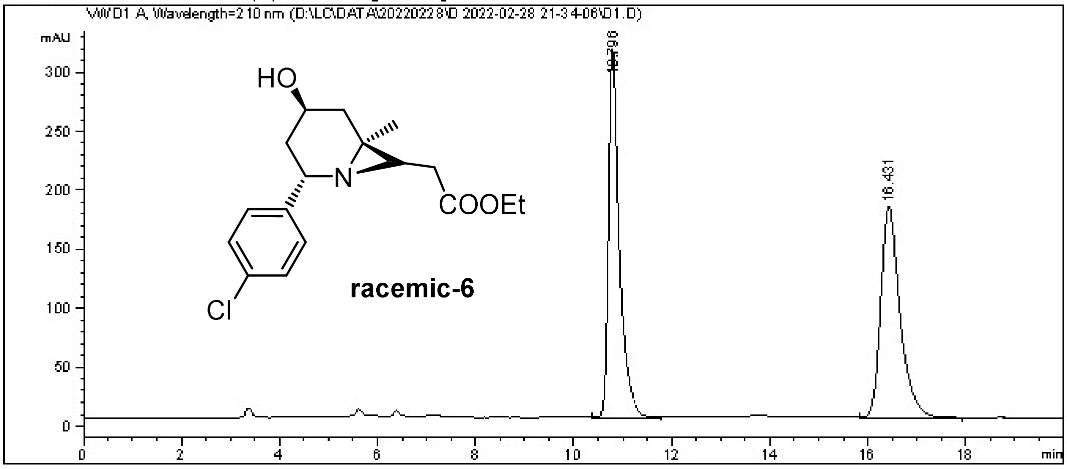


```
    =====================================================================
                         Area Percent Report
    =====================================================================

    Sorted By             :      Signal
    Multiplier            :      1.0000
    Dilution              :      1.0000
    Do not use Multiplier & Dilution Factor with ISTDs


    Signal 1: VWD1 A, Wavelength=210 nm
```

Peak #	RetTime [min]	Type	Width [min]	Area [mAU*s]	Height [mAU]	Area %
1	10.796	VB	0.2243	4849.10547	313.19150	49.6625
2	16.431	VB	0.4135	4915.00879	179.04135	50.3375

Totals :				9764.11426	492.23285	

```
    =====================================================================
                          *** End of Report ***
```


Supplementary Figure 120. HPLC spectrum of racemic-**6**

```
Data File D:\LC\DATA\20220228\C 2022-02-28 20-08-18\C1.D
Sample Name: FC04-578-HEW-S

=====================================================================
Acq. Operator   : 系统                         Seq. Line :   2
Sample Operator : 系统
Acq. Instrument : 1200                          Location :   32
Injection Date  : 2/28/2022 8:15:00 PM               Inj :   1
                                           Inj Volume : 2.000 µl
Acq. Method     : D:\LC\DATA\20220228\C 2022-02-28 20-08-18\P2-IC-90-10-1ML-20MIN-210NM-2uL.M
Last changed    : 2/15/2022 11:03:17 AM by 系统
Analysis Method : D:\LC\DATA\20220228\C 2022-02-28 20-08-18\P2-IC-90-10-1ML-20MIN-210NM-2uL.M
                  (Sequence Method)
Last changed    : 5/11/2022 11:19:02 AM by 系统
                  (modified after loading)
Additional Info : Peak(s) manually integrated
```

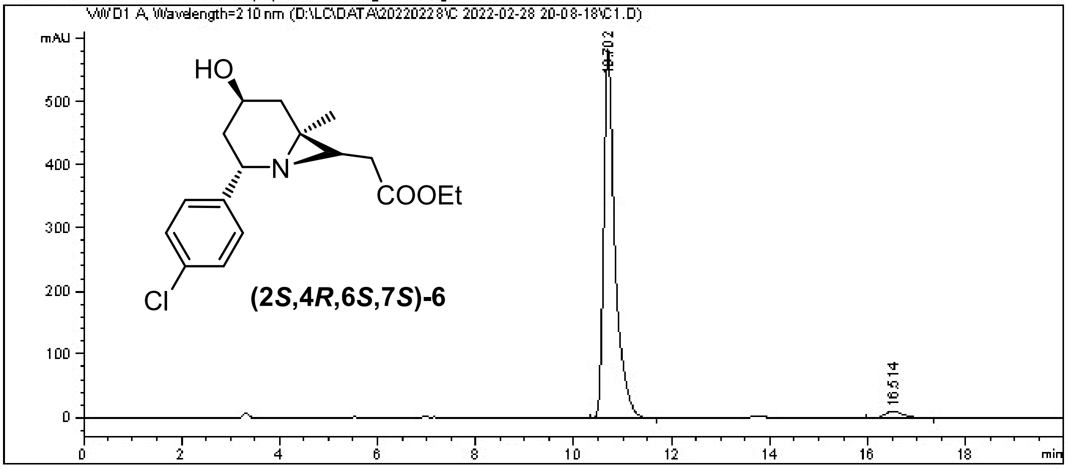


```
=====================================================================
                        Area Percent Report
=====================================================================

Sorted By             :      Signal
Multiplier            :      1.0000
Dilution              :      1.0000
Do not use Multiplier & Dilution Factor with ISTDs


Signal 1: VWD1 A, Wavelength=210 nm

Peak RetTime Type  Width     Area      Height     Area
  #   [min]        [min]   [mAU*s]     [mAU]        %
----|-------|----|-------|----------|----------|--------|
   1  10.702 BB    0.2337 9388.43066  585.52313  96.9058
   2  16.514 BB    0.4145  299.77350   10.88495   3.0942

Totals :                  9688.20416  596.40808

=====================================================================
                          *** End of Report ***
```

1200 5/11/2022 11:19:39 AM 系统 Page 1 of 1

Supplementary Figure 121. HPLC spectrum of (2*S*,4*R*,6*S*,7*S*)-**6**

4. Supplementary References

1. Belokon, Y. N.; Bhave, D.; D'Addario, D.; Groaz, E.; North, M.; Tagliazucca, V. *Tetrahedron*, **60**, 1849-1861 (2004).
2. Wang, C.-J.; Liang, G.; Xue, Z.-Y.; Gao, F. *J. Am. Chem. Soc.* **130**, 17250-17251 (2008).
3. Stanley, L. M.; Hartwig, J. F. *Angew. Chem. Int. Ed.* **48**, 7841-7844 (2009).
4. Trost, B. M.; Miller, J. R.; Hoffman, C. M. *J. Am. Chem. Soc.* **133**, 8165-8167 (2011).
5. Richards, C. J.; Mulvaney, A. W. *Tetrahedron: Asymmetry* **7**, 1419-1430 (1996).
6. Smith, C. R.; Mans, D. J.; Rajanbabu, T. V. *Org. Synth.* **85**, 238-247 (2008).
7. Frisch, M. J.; Trucks, G. W.; Schlegel, H. B.; Scuseria, G. E.; Robb, M. A.; Cheeseman, J. R.; Scalmani, G.; Barone, V.; Mennucci, B.; Petersson, G. A.; Nakatsuji, H.; Caricato, M.; Li, X.; Hratchian, H. P.; Izmaylov, A. F.; Bloino, J.; Zheng, G.; Sonnenberg, J. L.; Hada, M.; Ehara, M.; Toyota, K.; Fukuda, R.; Hasegawa, J.; Ishida, M.; Nakajima, T.; Honda, Y.; Kitao, O.; Nakai, H.; Vreven, T.; Montgomery, J. A., Jr.; Peralta, J. E.; Ogliaro, F.; Bearpark, M.; Heyd, J. J.; Brothers, E.; Kudin, K. N.; Staroverov, V. N.; Kobayashi, R.; Normand, J.; Raghavachari, K.; Rendell, A.; Burant, J. C.; Iyengar, S. S.; Tomasi, J.; Cossi, M.; Rega, N.; Millam, N. J.; Klene, M.; Knox, J. E.; Cross, J. B.; Bakken, V.; Adamo, C.; Jaramillo, J.; Gomperts, R.; Stratmann, R. E.; Yazyev, O.; Austin, A. J.; Cammi, R.; Pomelli, C.; Ochterski, J. W.; Martin, R. L.; Morokuma, K.; Zakrzewski, V. G.; Voth, G. A.; Salvador, P.; Dannenberg, J. J.; Dapprich, S.; Daniels, A. D.; Farkas, Ö .; Foresman, J. B.; Ortiz, J. V.; Cioslowski, J.; Fox, D. J. *Gaussian 09*; Gaussian, Inc.: Wallingford, CT, 2009.
8. Zhao, Y.; Truhlar, D. G. *Acc. Chem. Res.* **41**, 157–167 (2008).
9. Zhao, Y.; Truhlar, D. G. *Theor. Chem. Acc.* **120**, 215–241 (2008).
10. Andrae, D.; Häussermann, U.; Dolg, M.; Stoll, H.; Preuss, H. *Theor. Chim. Acta* **77**, 123–141 (1990).

11. Roy, L. E.; Hay, P. J.; Martin, R. L. *J. Chem. Theory Comput.* **4**, 1029–1031 (2008).

12. Weigend, F.; Ahlrichs, R. Balanced Basis Sets of Split Valence, *Phys. Chem. Chem. Phys.* **7**, 3297–3305 (2005).

13. Marenich, A. V.; Cramer, C. J.; Truhlar, D. G. *J. Phys. Chem. B* **113**, 6378–6396 (2009).

14. Legault, C. Y. *CYLview, 1.0b*; Universite′de Sherbrooke, 2009. http://www.cylview.org.
