# Supplementary material for: Modular access to chiral bridged piperidine-γ-butyrolactones via catalytic asymmetric allylation/aza-Prins cyclization/lactonization sequences
Source: Nat Commun. 2024 Jan 2;15:127. doi: 10.1038/s41467-023-44336-2 (PMC10762176; doi:10.1038/s41467-023-44336-2)
Supplement: Supplementary file 3 — Description of Additional Supplementary Files [file 41467_2023_44336_MOESM3_ESM.pdf]

Description of Additional Supplementary Files

File Name: Supplementary Data 1

Description: Cartesian coordinates of DFT optimized structures.
