# Supplementary material for: Modular access to chiral bridged piperidine-γ-butyrolactones via catalytic asymmetric allylation/aza-Prins cyclization/lactonization sequences
Source: Nat Commun. 2024 Jan 2;15:127. doi: 10.1038/s41467-023-44336-2 (PMC10762176; doi:10.1038/s41467-023-44336-2)
Supplement: Supplementary file 4 — Supplementary Data 1 [file 41467_2023_44336_MOESM4_ESM.docx]

**Cartesian Coordinates (Å) for the Optimized Structures**

**Cu-AY**

C -0.603018 -0.923040 2.152549

C -1.252240 -1.395771 3.340391

H -0.973491 -1.124074 4.352587

C -2.348741 -2.212927 2.949179

H -3.065755 -2.674674 3.619885

C -1.309777 -1.465165 1.006572

C -2.381894 -2.265862 1.526179

H -3.121228 -2.781617 0.921207

C 1.872736 1.727674 1.734791

C 2.362926 0.933306 2.946675

H 2.549989 1.535975 3.840903

N 0.782937 0.882241 1.213561

C 0.494463 0.030925 2.140971

O 1.262995 0.040770 3.252311

H 3.241767 0.311554 2.735263

H 2.650880 1.817043 0.964740

C 1.373024 3.134574 2.102618

C 0.584118 3.782183 0.975860

C 2.557776 4.010001 2.492256

H 0.711069 3.020552 2.981252

H -0.277414 3.178209 0.667741

H 0.219999 4.772282 1.277832

H 1.215958 3.916732 0.087571

H 3.150188 3.586329 3.313284

H 3.230430 4.140923 1.632672

H 2.226940 5.006512 2.806434

P -0.843244 -1.191882 -0.727182

C 0.078095 -2.727296 -1.124067

C 0.586127 -2.869149 -2.424223

C 0.363190 -3.716940 -0.176401

C 1.347413 -3.979175 -2.770418

H 0.389670 -2.092741 -3.165758

C 1.130891 -4.827274 -0.525197

H -0.014464 -3.622619 0.841122

C 1.623575 -4.961462 -1.819831

H 1.736682 -4.073003 -3.782683

H 1.345529 -5.588810 0.222735

H 2.228405 -5.825588 -2.087927

C -2.431811 -1.430138 -1.605810

C -3.095010 -0.305498 -2.111879

C -3.008118 -2.697116 -1.765832

C -4.330303 -0.448214 -2.740546

H -2.629221 0.677024 -2.019370

C -4.240769 -2.834424 -2.396222

H -2.480929 -3.580050 -1.401489

C -4.906632 -1.708724 -2.879121

H -4.840119 0.431215 -3.132275

H -4.680801 -3.823075 -2.516650

H -5.870109 -1.817799 -3.374434

Cu 0.172154 0.752809 -0.794470

O -1.146195 2.246343 -1.561820

C -0.463953 3.072877 -2.227218

O -1.087559 4.139990 -2.803477

C 0.930880 3.011282 -2.420101

N 1.545757 1.947227 -1.832054

C 2.859782 1.864839 -1.759336

H 3.479565 2.698434 -2.108442

C 3.551999 0.743555 -1.170904

C 4.858446 0.908206 -0.667929

C 2.971031 -0.534366 -1.047914

C 5.525719 -0.122796 -0.020799

H 5.345081 1.879521 -0.766941

C 3.622541 -1.574811 -0.402570

H 1.995637 -0.715283 -1.496430

C 4.895163 -1.357791 0.122843

H 6.527088 0.025095 0.377226

H 3.156761 -2.555896 -0.327685

C -2.482440 4.218026 -2.562490

H -3.012461 3.361104 -2.997314

H -2.700745 4.250338 -1.488125

C -2.968061 1.309941 1.055718

C -2.537061 1.670273 2.366918

H -1.652330 2.255711 2.600804

C -3.413253 1.057952 3.311563

H -3.324242 1.109296 4.391651

C -4.115282 0.467304 1.191034

H -4.645600 -0.018591 0.377616

C -4.391110 0.314022 2.584037

H -5.175230 -0.299343 3.016031

H -2.472897 1.592536 0.129256

C 1.705728 4.078992 -3.110449

H 2.385327 3.657738 -3.867333

H 1.041946 4.791130 -3.604943

H 2.344035 4.649461 -2.412805

Fe -2.525301 -0.348361 2.145822

Cl 5.721184 -2.658827 0.952862

H -2.813701 5.141592 -3.041892

**Cu-AY'**

C -0.091879 -2.702752 -0.669472

C 0.401700 -4.026128 -0.929390

H 0.112634 -4.640304 -1.774002

C 1.368011 -4.342664 0.060487

H 1.954147 -5.254386 0.107803

C 0.588250 -2.191627 0.512523

C 1.475185 -3.234454 0.945854

H 2.124906 -3.182614 1.813800

C -2.405111 -0.686635 -2.675071

C -2.408928 -2.076612 -3.322245

H -1.918348 -2.078618 -4.307324

N -1.358175 -0.795911 -1.640535

C -1.030101 -2.042847 -1.565168

O -1.618339 -2.895002 -2.442157

H -3.394822 -2.539958 -3.417022

H -3.350210 -0.484878 -2.143201

C -2.176886 0.429237 -3.696103

C -2.114710 1.802820 -3.051914

C -3.280724 0.381197 -4.748692

H -1.211318 0.222855 -4.192114

H -1.272124 1.895598 -2.361240

H -2.011717 2.585018 -3.813922

H -3.027450 2.011440 -2.475416

H -3.322945 -0.579530 -5.278582

H -4.263534 0.548397 -4.286442

H -3.136941 1.163123 -5.502713

P 0.114283 -0.641845 1.349258

C -0.569074 -1.283085 2.925410

C 0.231865 -1.599907 4.028211

C -1.955002 -1.468657 3.004416

C -0.343959 -2.104594 5.190023

H 1.309272 -1.435386 3.980724

C -2.525110 -1.981090 4.166529

H -2.580965 -1.188348 2.154203

C -1.722135 -2.299563 5.259181

H 0.284474 -2.343640 6.046534

H -3.603968 -2.119476 4.222541

H -2.170364 -2.691455 6.170871

C 1.638628 0.221810 1.870649

C 1.468668 1.551314 2.284869

C 2.931113 -0.311848 1.844038

C 2.562815 2.327565 2.648246

H 0.467972 1.987927 2.282980

C 4.029273 0.473505 2.189799

H 3.092596 -1.333752 1.506926

C 3.848690 1.793922 2.590051

H 2.411248 3.363826 2.945120

H 5.031261 0.048974 2.138843

H 4.709040 2.410369 2.844280

Cu -1.315451 0.419319 0.103947

O -3.418070 0.098549 0.358392

C -3.939796 1.235478 0.510317

O -5.278110 1.329282 0.769749

C -3.261855 2.471616 0.433431

N -1.928464 2.389243 0.160931

C -1.175089 3.470700 0.122266

H -1.614300 4.438370 0.386948

C 0.224420 3.502812 -0.216011

C 0.956128 4.668112 0.100188

C 0.928197 2.462289 -0.861652

C 2.313444 4.780446 -0.154703

H 0.435841 5.496325 0.582454

C 2.282992 2.571703 -1.141244

H 0.400313 1.550148 -1.149645

C 2.976194 3.720492 -0.769388

H 2.860573 5.679672 0.118587

H 2.808380 1.777997 -1.666736

C -5.951178 0.088963 0.895860

H -5.919340 -0.484003 -0.038680

H -5.509544 -0.525505 1.689841

H -6.984904 0.338016 1.144985

C 3.033226 -1.011062 -1.337668

C 2.166974 -1.285778 -2.434991

H 1.375006 -0.632845 -2.794075

C 2.453464 -2.604418 -2.904253

H 1.931120 -3.124817 -3.700630

C 3.852416 -2.157833 -1.120243

H 4.592993 -2.271710 -0.334801

C 3.496747 -3.143575 -2.089957

H 3.915377 -4.141408 -2.168769

H 3.024794 -0.113629 -0.729807

C -3.940630 3.783262 0.627409

H -5.015372 3.651234 0.765551

H -3.555022 4.328349 1.504437

H -3.784533 4.448906 -0.237267

Cl 4.691937 3.832807 -1.082904

Fe 1.883918 -2.653047 -0.966892

**1b**

C -1.334049 0.477852 -0.126195

H -1.135249 1.180743 -0.947072

H -1.387814 1.065825 0.799083

C -2.567324 -0.316952 -0.355322

H -2.532747 -1.014100 -1.194148

C -3.675234 -0.188420 0.370353

H -3.727129 0.495703 1.217077

O -0.225627 -0.442718 -0.043448

C 0.972311 0.155485 -0.017130

O 1.183434 1.346347 -0.065098

O 1.897997 -0.810045 0.072373

C 3.236242 -0.313775 0.111725

H 3.390464 0.314665 0.994481

H 3.458518 0.275156 -0.783395

H 3.872507 -1.198366 0.156429

H -4.576656 -0.753445 0.146339

**Ir-A1**

Ir 1.999790 -1.464630 -0.309047

P 0.270502 0.048720 -0.021019

N 0.952489 1.541017 0.240921

O -0.737166 0.031202 -1.323463

O -0.780040 0.000354 1.227984

C -1.936382 -0.779328 1.187214

C -2.010272 -1.827405 2.123037

H -1.190694 -1.951969 2.829968

C -3.113672 -2.643417 2.134025

H -3.192751 -3.452007 2.859176

C -4.155145 -2.460169 1.193051

C -5.261043 -3.342698 1.141113

H -5.307164 -4.160103 1.859848

C -6.248631 -3.183122 0.198999

H -7.091400 -3.870053 0.166858

C -6.160086 -2.133642 -0.737635

H -6.929288 -2.024548 -1.499149

C -5.108598 -1.247633 -0.702897

H -5.052522 -0.451877 -1.441371

C -4.083975 -1.367573 0.267883

C -2.968122 -0.465570 0.317726

C -2.897146 0.734950 -0.545663

C -3.925322 1.737030 -0.555456

C -5.019314 1.722569 0.345278

H -5.092270 0.926459 1.082241

C -5.980136 2.706665 0.309546

H -6.806050 2.675806 1.017194

C -5.904151 3.755846 -0.628179

H -6.676298 4.521621 -0.649515

C -4.845703 3.814507 -1.501178

H -4.762615 4.629030 -2.219582

C -3.830201 2.828364 -1.482124

C -2.718309 2.912748 -2.354259

H -2.671256 3.731964 -3.070241

C -1.703371 1.992503 -2.285116

H -0.832961 2.041004 -2.935731

C -1.801056 0.937718 -1.362778

C 0.554830 2.584192 1.258250

H 1.149779 3.450968 0.937245

C 1.040817 2.197502 2.641110

C 2.356669 2.507352 3.012593

H 2.976590 3.097194 2.334678

C 2.870644 2.107456 4.243597

H 3.893536 2.363604 4.512767

C 2.066481 1.403854 5.135980

H 2.460886 1.094345 6.101711

C 0.742724 1.127247 4.798108

H 0.097118 0.610159 5.505976

C 0.233841 1.522916 3.564599

H -0.804543 1.309960 3.322607

C -0.897954 3.004265 1.169591

H -1.154609 3.283181 0.141329

H -1.597247 2.230858 1.496991

H -1.050483 3.881316 1.807661

C 2.368325 1.615541 -0.170789

H 2.977747 1.574891 0.748177

C 2.726134 0.418543 -1.043224

H 2.321425 0.570744 -2.055677

H 3.817541 0.361463 -1.146487

C 2.696493 2.906887 -0.885926

C 1.759528 3.562136 -1.688255

H 0.741875 3.173494 -1.739264

C 2.112201 4.711934 -2.387601

H 1.371407 5.219143 -3.003201

C 3.406700 5.218418 -2.294999

H 3.681291 6.118030 -2.841601

C 4.344728 4.575354 -1.491092

H 5.353869 4.972924 -1.404355

C 3.988422 3.429149 -0.786991

H 4.721603 2.930068 -0.149820

C 2.370640 -1.325501 1.874073

H 2.123655 -2.307553 2.280982

H 1.940802 -0.485261 2.424458

C 3.592490 -1.116201 1.188053

H 3.991682 -0.103360 1.140101

C 4.100735 -2.089380 0.294902

H 4.073346 -3.146474 0.545808

C 1.225511 -1.852372 -2.362979

H 1.051676 -0.871593 -2.804118

C 2.567587 -2.276024 -2.303040

H 3.319494 -1.595054 -2.707077

C 3.000687 -3.715451 -2.207252

H 2.939495 -4.173305 -3.207422

H 4.063567 -3.740331 -1.939803

C 2.195609 -4.547641 -1.199962

H 2.820608 -5.372326 -0.838893

H 1.342015 -5.026867 -1.694990

C 1.684417 -3.769864 -0.004747

H 2.173285 -3.976409 0.948095

C 0.429432 -3.185370 0.056514

H 0.019117 -2.978314 1.045919

C -0.560532 -3.174557 -1.075413

H -1.044152 -4.163291 -1.128728

H -1.364015 -2.469978 -0.832122

C 0.042517 -2.801539 -2.434505

H -0.736356 -2.330061 -3.043114

H 0.346110 -3.700306 -2.986686

H 4.921772 -1.798152 -0.360576

**Ir-A2**

Ir 1.989116 -1.546854 -0.227911

O -0.778919 0.003757 1.271491

O -0.757931 -0.065131 -1.273452

C -1.952244 -0.752709 1.253031

C -2.031366 -1.790684 2.199564

H -1.200178 -1.931128 2.888924

C -3.154501 -2.577337 2.247378

H -3.236191 -3.376125 2.983028

C -4.214638 -2.375688 1.331784

C -5.345602 -3.227205 1.321498

H -5.396923 -4.030507 2.055614

C -6.350415 -3.056353 0.400131

H -7.212432 -3.719612 0.399488

C -6.253284 -2.027014 -0.557478

H -7.035597 -1.910091 -1.304349

C -5.176735 -1.170863 -0.563181

H -5.115842 -0.392407 -1.318851

C -4.133948 -1.300211 0.387156

C -2.992072 -0.428786 0.395877

C -2.896779 0.730460 -0.519770

C -3.893545 1.762203 -0.579240

C -4.970437 1.839571 0.338524

H -5.055829 1.088815 1.121236

C -5.898604 2.852609 0.258192

H -6.711282 2.894554 0.980510

C -5.804841 3.838591 -0.744757

H -6.550954 4.628055 -0.800574

C -4.763247 3.804988 -1.639061

H -4.667504 4.569694 -2.408714

C -3.780886 2.788299 -1.574686

C -2.685714 2.780223 -2.471489

H -2.629008 3.547077 -3.242509

C -1.697205 1.836810 -2.355916

H -0.837997 1.814858 -3.022431

C -1.806125 0.852510 -1.360234

P 0.267255 -0.000119 0.014312

N 0.962304 1.494104 0.207630

C 2.806946 0.303162 -0.927680

H 2.500780 0.392994 -1.981050

H 3.902416 0.253426 -0.930450

C 2.383160 1.565282 -0.186942

H 2.975648 1.651364 0.737841

C 2.693799 2.789948 -1.019433

C 1.780272 3.304971 -1.942449

H 0.790057 2.853812 -2.015901

C 2.116084 4.402367 -2.728851

H 1.392424 4.802831 -3.436537

C 3.371626 4.994112 -2.605784

H 3.632132 5.854272 -3.218610

C 4.286810 4.488807 -1.685699

H 5.264542 4.953390 -1.576122

C 3.946117 3.395657 -0.894232

H 4.659335 3.005702 -0.165442

C 0.547989 2.628952 1.115301

H 1.067283 3.483262 0.658208

C 1.140333 2.441621 2.497061

C 2.398304 2.992514 2.769563

H 2.893286 3.588715 1.999932

C 3.012217 2.808381 4.006006

H 3.989050 3.248178 4.197596

C 2.365212 2.077527 4.998077

H 2.835592 1.937509 5.969228

C 1.100581 1.546644 4.749618

H 0.578503 0.998905 5.532259

C 0.492248 1.725891 3.510000

H -0.498176 1.311816 3.331967

C -0.927215 2.971007 1.089951

H -1.274667 3.120727 0.061640

H -1.563177 2.220046 1.565071

H -1.067707 3.912595 1.631804

C 2.552215 -2.349260 -2.215863

H 3.328546 -1.684103 -2.600395

C 1.225509 -1.867677 -2.277165

H 1.110496 -0.874269 -2.710269

C -0.011460 -2.742885 -2.392748

H -0.767205 -2.184608 -2.954437

H 0.234779 -3.614329 -3.013110

C -0.632971 -3.183643 -1.058260

H -1.147019 -4.149908 -1.183608

H -1.415722 -2.473353 -0.769921

C 0.349986 -3.313308 0.073538

H -0.038159 -3.132248 1.077545

C 1.563455 -3.954124 -0.047050

H 2.077906 -4.224857 0.872258

C 2.040593 -4.662342 -1.292186

H 2.595112 -5.557753 -0.989085

H 1.170632 -5.032747 -1.847058

C 2.930022 -3.805110 -2.197800

H 2.903719 -4.192605 -3.228729

H 3.976615 -3.893385 -1.882914

C 2.483142 -0.890411 1.866353

H 2.922730 0.099925 1.774375

H 1.712619 -0.967314 2.633544

C 3.231164 -2.044243 1.553556

H 2.948789 -2.977841 2.038671

C 4.081522 -2.086846 0.423720

H 4.684851 -1.221276 0.156989

H 4.508847 -3.048681 0.142532

**TS1**

C 6.792394 0.225030 -1.089297

C 8.055197 0.027718 -1.741525

H 8.965132 0.570636 -1.511329

C 7.909824 -1.029234 -2.680719

H 8.703127 -1.446481 -3.291763

C 5.850148 -0.731151 -1.648945

C 6.563323 -1.488899 -2.635445

H 6.145176 -2.307791 -3.212722

C 5.879473 2.346225 1.758333

C 6.944973 3.142798 0.992558

H 7.803445 3.444430 1.599942

N 5.636752 1.189316 0.874689

C 6.559194 1.197514 -0.033550

O 7.432910 2.221787 -0.011566

H 6.548561 4.018826 0.466080

H 4.941024 2.915011 1.850570

C 6.340681 1.928009 3.162892

C 5.392627 0.933027 3.813403

C 6.495927 3.162791 4.042891

H 7.329278 1.448723 3.044261

H 5.292446 0.011377 3.229648

H 5.745584 0.665096 4.816677

H 4.388452 1.367547 3.921253

H 7.188249 3.905053 3.627102

H 5.525642 3.659265 4.182824

H 6.870682 2.890404 5.035556

P 4.098796 -0.815670 -1.212864

C 3.310315 0.205717 -2.509777

C 1.909545 0.206789 -2.593734

C 4.038187 1.076123 -3.329394

C 1.254877 1.047423 -3.486249

H 1.330664 -0.460485 -1.946526

C 3.377339 1.924490 -4.217609

H 5.126507 1.089604 -3.278560

C 1.988514 1.914124 -4.297010

H 0.167093 1.040104 -3.545443

H 3.956033 2.593832 -4.851775

H 1.473201 2.580556 -4.985648

C 3.602311 -2.519062 -1.651269

C 3.439113 -3.459861 -0.625560

C 3.383860 -2.910457 -2.979113

C 3.091890 -4.774881 -0.926112

H 3.582852 -3.146829 0.409723

C 3.028959 -4.223220 -3.274817

H 3.489027 -2.179101 -3.781091

C 2.887367 -5.158208 -2.249882

H 2.973028 -5.501762 -0.123523

H 2.861458 -4.518071 -4.309203

H 2.611019 -6.184549 -2.484343

Cu 3.973324 -0.033794 0.843051

O 3.941774 -1.735605 2.198735

C 2.949696 -1.617541 2.947400

O 2.605432 -2.611163 3.796680

C 2.065412 -0.488315 2.959362

N 2.451276 0.552107 2.140319

C 1.838561 1.708279 2.215352

H 1.093535 1.883882 3.000417

C 2.094734 2.825477 1.329606

C 1.713011 4.119261 1.731535

C 2.699097 2.686816 0.066540

C 1.973481 5.231387 0.943571

H 1.227320 4.254602 2.698293

C 2.967023 3.787032 -0.733967

H 2.944670 1.691139 -0.300825

C 2.617686 5.059409 -0.280978

H 1.688453 6.227487 1.271731

H 3.423008 3.659535 -1.713698

C 3.433855 -3.770679 3.736795

C 6.682205 -2.785493 0.731205

C 7.689053 -1.891172 1.198315

H 7.539118 -1.101949 1.929555

C 8.894135 -2.164811 0.485469

H 9.833441 -1.632017 0.588311

C 7.262222 -3.610999 -0.279654

H 6.742446 -4.367347 -0.859418

C 8.630321 -3.229753 -0.428526

H 9.334629 -3.645199 -1.141101

H 5.650396 -2.794247 1.067046

C 1.057341 -0.296131 4.038915

H 0.102677 0.077443 3.640441

H 0.866262 -1.231207 4.569482

H 1.395463 0.447759 4.778852

Fe 7.410050 -1.657092 -0.802834

Cl 2.974969 6.447987 -1.267920

Ir -1.953263 -1.227369 -0.288452

P -3.839442 0.031739 -0.078790

O -4.874573 -0.028330 1.194090

O -4.858018 -0.282237 -1.346525

N -3.425345 1.650338 -0.020446

C -5.843285 -1.023304 1.275597

C -5.699303 -1.958359 2.317073

H -4.857930 -1.849392 3.000041

C -6.621042 -2.965956 2.449718

H -6.528491 -3.693940 3.254497

C -7.692564 -3.088734 1.532865

C -8.611194 -4.162533 1.616625

H -8.486257 -4.890976 2.417142

C -9.630659 -4.293900 0.704764

H -10.327407 -5.126249 0.777609

C -9.764973 -3.353281 -0.336538

H -10.558327 -3.472115 -1.071632

C -8.898749 -2.289565 -0.437164

H -9.012399 -1.576008 -1.249946

C -7.848218 -2.111754 0.496835

C -6.926048 -1.016507 0.411632

C -7.094174 0.084100 -0.562818

C -8.286192 0.879222 -0.615472

C -9.336772 0.736798 0.324495

H -9.244104 -0.000034 1.118493

C -10.460365 1.527518 0.258481

H -11.248459 1.403756 0.998435

C -10.597074 2.500572 -0.751603

H -11.493531 3.115091 -0.795872

C -9.588194 2.680228 -1.667173

H -9.672603 3.441224 -2.442260

C -8.410704 1.895633 -1.618199

C -7.345928 2.110396 -2.526952

H -7.458619 2.869486 -3.299879

C -6.179138 1.395400 -2.422342

H -5.340386 1.557043 -3.096549

C -6.063376 0.410103 -1.425432

C -2.043953 1.866398 -0.492443

H -1.388116 1.977755 0.392139

C -1.900493 3.101521 -1.351093

C -2.951674 3.589018 -2.132281

H -3.935607 3.123697 -2.052631

C -2.750241 4.666285 -2.990687

H -3.578869 5.042867 -3.588294

C -1.495179 5.263553 -3.084488

H -1.339036 6.105113 -3.756589

C -0.443119 4.785380 -2.305899

H 0.540317 5.250385 -2.368289

C -0.648261 3.715834 -1.439774

H 0.177587 3.339911 -0.830999

C -1.584346 0.640204 -1.270557

H -2.059908 0.641860 -2.265976

H -0.504557 0.721988 -1.436735

C -3.952152 2.724276 0.889529

H -3.532385 3.634870 0.436995

C -3.349207 2.604922 2.276323

C -2.098611 3.185765 2.526796

H -1.621277 3.782691 1.746426

C -1.469392 3.025025 3.759276

H -0.502136 3.493178 3.941275

C -2.084228 2.289160 4.768391

H -1.593275 2.162992 5.731869

C -3.344889 1.739516 4.544557

H -3.848463 1.190780 5.338441

C -3.972914 1.900935 3.313049

H -4.958230 1.469100 3.155735

C -5.459538 2.883969 0.849096

H -5.805690 2.986230 -0.185598

H -6.001760 2.052569 1.306620

H -5.732638 3.796919 1.389885

C -1.517528 -0.525046 1.702065

H -1.696215 -1.292368 2.463608

H -1.950565 0.441167 1.971471

C -0.225780 -0.507566 1.059563

H 0.105753 0.427201 0.600661

C 0.738394 -1.503229 1.162844

H 0.544034 -2.436836 1.689070

C -2.541646 -2.089229 -2.231711

H -2.928990 -1.266806 -2.835174

C -1.132527 -2.163102 -2.104047

H -0.552546 -1.416779 -2.654554

C -0.367794 -3.418007 -1.760984

H -0.256777 -4.052318 -2.657044

H 0.653342 -3.123979 -1.479079

C -0.987580 -4.227712 -0.613756

H -1.674210 -4.990630 -1.002831

H -0.191529 -4.786255 -0.104037

C -1.720771 -3.386697 0.415808

H -1.264967 -3.341272 1.405446

C -3.086020 -3.094214 0.377073

H -3.557583 -2.855182 1.330912

C -4.038305 -3.485913 -0.721419

H -4.944316 -2.874749 -0.618437

H -4.361676 -4.529337 -0.571620

C -3.463454 -3.290890 -2.127201

H -2.928831 -4.191112 -2.457593

H -4.291264 -3.162600 -2.833987

H 1.633905 -1.470264 0.540158

H 3.052960 -4.442608 4.506401

H 3.364175 -4.247711 2.751361

H 4.480314 -3.517311 3.931778

**TS1'**

C -4.978269 1.947129 -0.937125

C -5.538571 3.215721 -1.315349

H -5.290617 3.763976 -2.217175

C -6.504562 3.581660 -0.339263

H -7.131467 4.466162 -0.369179

C -5.619441 1.525079 0.298478

C -6.559465 2.553180 0.643247

H -7.215418 2.532300 1.508109

C -3.013275 -0.416942 -2.897543

C -2.646696 0.949895 -3.472145

H -2.743219 1.034694 -4.558226

N -3.716358 -0.054941 -1.655234

C -4.090484 1.179562 -1.787714

O -3.625580 1.835617 -2.878078

H -1.650045 1.301282 -3.165071

H -2.122878 -1.011051 -2.640821

C -3.905410 -1.239924 -3.843942

C -4.603702 -2.388457 -3.135319

C -3.086358 -1.750817 -5.021926

H -4.672488 -0.544703 -4.229598

H -5.202214 -2.043024 -2.284457

H -5.262922 -2.924284 -3.828548

H -3.875290 -3.106546 -2.737711

H -2.568287 -0.947713 -5.562348

H -2.324824 -2.466881 -4.679427

H -3.721368 -2.272700 -5.746072

P -5.164862 0.079689 1.295406

C -4.608759 0.820734 2.871380

C -4.365932 -0.047878 3.947050

C -4.354731 2.186886 3.034889

C -3.910440 0.444665 5.164234

H -4.544396 -1.118058 3.827626

C -3.899621 2.676605 4.257320

H -4.503140 2.876788 2.204949

C -3.681466 1.811371 5.324751

H -3.740392 -0.239955 5.993566

H -3.711088 3.743865 4.369050

H -3.330873 2.197928 6.279933

C -6.747028 -0.716649 1.747306

C -7.060951 -1.932769 1.129202

C -7.645230 -0.166896 2.670789

C -8.269219 -2.570309 1.398186

H -6.342420 -2.378132 0.436408

C -8.849684 -0.809035 2.943900

H -7.389060 0.755574 3.193536

C -9.166040 -2.005962 2.302247

H -8.506199 -3.514069 0.910245

H -9.541581 -0.377959 3.665024

H -10.107768 -2.506392 2.519647

Cu -3.715693 -1.085744 0.145050

O -3.689724 -3.169728 -0.220069

C -2.774806 -3.716415 0.428653

O -2.636913 -5.057305 0.440521

C -1.768709 -3.008070 1.173366

N -1.956671 -1.644258 1.190668

C -1.285060 -0.883514 2.017326

H -0.671497 -1.320258 2.815212

C -1.272196 0.559157 1.903564

C -0.875907 1.351479 2.992673

C -1.593626 1.207865 0.695558

C -0.852801 2.736285 2.905542

H -0.618709 0.866959 3.934054

C -1.583791 2.591560 0.594054

H -1.837627 0.601867 -0.176426

C -1.227850 3.348525 1.710867

H -0.574641 3.344522 3.762882

H -1.837687 3.087558 -0.341961

C -3.593218 -5.778444 -0.336674

H -4.612904 -5.523284 -0.034065

H -3.473951 -5.555530 -1.402659

C -7.739261 -0.038305 -1.666336

C -7.306949 0.649364 -2.837302

H -6.533510 0.315108 -3.522948

C -8.015272 1.883619 -2.914497

H -7.880285 2.650717 -3.669023

C -8.717838 0.771906 -1.013898

H -9.209568 0.540358 -0.074155

C -8.890037 1.958357 -1.789224

H -9.534600 2.794402 -1.540022

H -7.362046 -0.990155 -1.309063

C -0.904719 -3.691498 2.176383

H -0.822212 -4.759873 1.963524

H 0.106211 -3.257694 2.195124

H -1.306259 -3.580912 3.196689

Fe -6.965317 1.780085 -1.177138

Cl -1.261746 5.088084 1.613749

Ir 2.396237 -1.455677 -1.540047

P 4.023210 -0.472350 -0.282078

O 3.774082 0.759480 0.776920

O 5.172632 0.180546 -1.273866

N 4.672129 -1.594545 0.773421

C 3.616391 2.063426 0.323948

C 2.343984 2.641470 0.495933

H 1.563327 2.054134 0.981493

C 2.124568 3.922196 0.056622

H 1.149411 4.389999 0.190030

C 3.152957 4.648605 -0.591983

C 2.916981 5.941107 -1.118571

H 1.925699 6.376617 -0.995352

C 3.908071 6.628631 -1.777093

H 3.713015 7.619778 -2.181084

C 5.178972 6.040969 -1.940145

H 5.955945 6.577283 -2.481048

C 5.446649 4.793385 -1.426467

H 6.430179 4.350977 -1.565865

C 4.454992 4.063226 -0.726066

C 4.700553 2.754471 -0.191924

C 6.047744 2.140804 -0.192588

C 7.178131 2.789230 0.409972

C 7.064078 3.994698 1.146193

H 6.085146 4.451697 1.267611

C 8.166676 4.583653 1.720765

H 8.049027 5.504691 2.288284

C 9.444532 4.004531 1.587198

H 10.309030 4.485792 2.039532

C 9.588646 2.823738 0.900624

H 10.566505 2.352848 0.805641

C 8.472680 2.180302 0.313691

C 8.611542 0.935548 -0.345579

H 9.602547 0.491665 -0.429772

C 7.513941 0.282654 -0.845015

H 7.591664 -0.685303 -1.335531

C 6.247476 0.884656 -0.740173

C 4.221761 -2.966992 0.456523

H 3.366435 -3.203263 1.115118

C 5.301470 -3.991824 0.711103

C 6.640142 -3.731038 0.403920

H 6.914542 -2.741890 0.033542

C 7.609205 -4.711654 0.587283

H 8.649519 -4.495598 0.350762

C 7.252085 -5.967759 1.074582

H 8.011271 -6.733643 1.218508

C 5.921225 -6.236153 1.382837

H 5.635703 -7.212787 1.769307

C 4.953990 -5.250125 1.206367

H 3.909962 -5.456706 1.452089

C 3.742953 -3.028140 -0.986643

H 4.615686 -3.020679 -1.658531

H 3.230805 -3.985055 -1.150723

C 5.068957 -1.386252 2.209076

H 5.580795 -2.330112 2.446843

C 3.851666 -1.309706 3.112121

C 3.241093 -2.498489 3.533828

H 3.701368 -3.452919 3.271210

C 2.078844 -2.479193 4.301482

H 1.623078 -3.415858 4.619212

C 1.519428 -1.262188 4.683094

H 0.617920 -1.242568 5.295299

C 2.143541 -0.072531 4.310436

H 1.738191 0.884482 4.636351

C 3.298480 -0.096189 3.535315

H 3.775681 0.840863 3.258732

C 6.090002 -0.283598 2.407022

H 6.966868 -0.455324 1.773340

H 5.703222 0.714185 2.182932

H 6.420567 -0.288225 3.451578

C 1.309019 -1.322111 0.324116

H 0.585397 -0.506124 0.263970

H 1.903882 -1.286994 1.241935

C 0.897754 -2.624531 -0.139788

H 1.474868 -3.481193 0.212647

C -0.314394 -2.921771 -0.754069

H -0.966472 -2.137928 -1.135856

C 3.465210 -1.274008 -3.428026

H 4.487912 -1.618100 -3.264310

C 2.483257 -2.297465 -3.557460

H 2.847065 -3.326663 -3.510111

C 1.177352 -2.129000 -4.296279

H 1.333793 -2.234286 -5.383415

H 0.513459 -2.956326 -4.008927

C 0.484737 -0.800233 -3.987379

H 0.819048 -0.018325 -4.681705

H -0.591670 -0.907465 -4.172760

C 0.702474 -0.321790 -2.564753

H -0.155380 -0.386104 -1.889616

C 1.687713 0.606673 -2.219952

H 1.517018 1.201730 -1.321470

C 2.702052 1.163683 -3.186293

H 3.474430 1.683422 -2.605961

H 2.224586 1.939911 -3.807140

C 3.369769 0.100438 -4.072987

H 2.851206 0.015084 -5.037553

H 4.382999 0.439069 -4.317805

H -3.388200 -6.832203 -0.146212

H -0.513861 -3.930607 -1.113287

**2a**

O -3.457658 0.045945 1.415362

C -3.431442 -0.155388 0.221916

O -3.999870 -1.212690 -0.382601

C -2.717473 0.745357 -0.783793

N -1.331235 0.284982 -0.792316

C -0.453045 0.962117 -0.157905

H -0.681912 1.900763 0.376177

C 0.946306 0.543412 -0.097477

C 1.864346 1.302519 0.636331

C 1.395018 -0.607574 -0.760990

C 3.200461 0.930462 0.717825

H 1.523854 2.198543 1.155922

C 2.724345 -0.990720 -0.693001

H 0.674538 -1.191710 -1.329853

C 3.617669 -0.216171 0.049106

H 3.913840 1.516828 1.290825

H 3.078142 -1.881818 -1.205356

C -4.634924 -2.130951 0.513853

H -3.912154 -2.520130 1.236906

H -5.445390 -1.636313 1.057695

C -2.976137 2.203118 -0.440173

H -2.366872 2.864349 -1.066430

H -4.029402 2.450113 -0.611982

H -2.761135 2.409594 0.613001

Cl 5.289177 -0.698161 0.142711

H -5.024693 -2.932986 -0.113589

H -3.116699 0.513492 -1.779421

(*S*)-**3a**

O 2.330981 -0.211737 1.855622

C 2.828178 -0.400748 0.772379

O 4.157718 -0.558939 0.570674

C 2.080551 -0.518114 -0.562270

N 0.708196 -0.110568 -0.303022

C -0.259419 -0.915507 -0.498394

H -0.126596 -1.952561 -0.856740

C -1.650573 -0.528122 -0.263107

C -2.675649 -1.435027 -0.551963

C -1.985069 0.732482 0.251225

C -4.008181 -1.103253 -0.342454

H -2.423273 -2.418916 -0.948855

C -3.308752 1.079043 0.467932

H -1.181313 1.428549 0.482577

C -4.311347 0.155372 0.167059

H -4.806004 -1.805917 -0.567618

H -3.575059 2.053970 0.868665

C 4.948300 -0.407970 1.751312

H 4.831286 0.601700 2.158306

H 4.645945 -1.131907 2.514036

C 2.287345 -1.927117 -1.120303

H 1.779703 -2.039637 -2.086317

H 3.353839 -2.115164 -1.272494

H 1.904609 -2.698003 -0.440817

Cl -5.977615 0.586960 0.435285

H 5.979862 -0.580905 1.441551

C 2.672256 0.522499 -1.549485

C 2.673418 1.914184 -1.008156

H 2.067922 0.465198 -2.467923

H 3.693259 0.216267 -1.808204

C 3.776722 2.624254 -0.776702

H 1.695050 2.333294 -0.767951

H 4.767697 2.220572 -0.989453

H 3.738883 3.632664 -0.370258

**Int-I**

C 1.471622 1.970181 -0.715192

C -0.912819 1.597096 -0.262111

C -0.411284 5.155107 -0.332976

C 0.593783 4.350511 0.016244

C 1.345256 3.495945 -0.954712

H 0.290204 0.297274 -1.264574

H -0.774477 5.206150 -1.360171

H -0.901545 5.805657 0.387823

H 0.932335 3.628754 -1.964995

H 2.383510 3.858576 -1.003800

H -0.903478 2.553570 0.257414

C -2.118432 0.822172 -0.197522

C -3.193307 1.388445 0.513534

C -2.261563 -0.466196 -0.749955

C -4.379790 0.697902 0.678378

H -3.081881 2.380890 0.948418

C -3.440363 -1.167838 -0.579577

H -1.445492 -0.935595 -1.296932

C -4.491558 -0.580947 0.129718

H -5.210509 1.129186 1.229570

H -3.544232 -2.172090 -0.980118

C 1.878264 1.679890 0.748950

O 1.171395 1.981549 1.686096

O 3.066744 1.093068 0.825422

N 0.180761 1.266868 -0.880443

C 3.398680 0.574443 2.125924

H 4.403226 0.164238 2.022895

H 3.369957 1.370805 2.874200

H 2.678336 -0.209317 2.377450

O 0.782348 -0.921474 0.893078

S 0.799398 -1.959825 -0.157919

O 0.752374 -1.356979 -1.534248

O -0.070166 -3.117740 0.027278

C 2.519006 -2.622140 -0.071914

F 3.408670 -1.647060 -0.340922

F 2.777318 -3.076924 1.159682

F 2.709027 -3.614023 -0.943616

Cl -5.968882 -1.458256 0.337374

H 0.928003 4.326932 1.053320

C 2.433412 1.392577 -1.743325

H 3.420434 1.846850 -1.627592

H 2.538526 0.311265 -1.633502

H 2.070246 1.608101 -2.755042

**TS2**

C -1.767862 1.904488 0.796291

C 0.406640 1.732448 -0.299846

C 0.549559 3.569787 -0.212828

C -0.803874 3.916785 -0.280412

C -1.710905 3.517320 0.785123

H -0.242417 0.514269 1.216411

H 1.044827 3.626592 0.755920

H 1.191784 3.859178 -1.044903

H -1.342945 3.783014 1.781620

H -2.728877 3.897486 0.658105

H -0.038325 1.656876 -1.294761

C 1.758686 1.151517 -0.212636

C 2.350878 0.631101 -1.368009

C 2.438672 1.061514 1.007317

C 3.591504 0.013453 -1.310231

H 1.807556 0.666079 -2.311085

C 3.679512 0.449677 1.080895

H 1.977493 1.457925 1.911988

C 4.246489 -0.068426 -0.083524

H 4.043673 -0.417986 -2.198877

H 4.207661 0.364110 2.026555

C -2.472173 1.497216 -0.508770

O -2.197820 2.010660 -1.579624

O -3.345259 0.522615 -0.321350

N -0.418838 1.442256 0.768230

C -3.808658 -0.132654 -1.518353

H -4.539437 -0.864197 -1.174714

H -4.259529 0.593990 -2.199187

H -2.954554 -0.628675 -1.986613

O -0.758801 -0.804328 -1.023821

S -0.308995 -1.782594 -0.006620

O -0.138095 -1.174811 1.350970

O 0.742462 -2.709988 -0.409253

C -1.808347 -2.836270 0.228559

F -2.816218 -2.115576 0.761752

F -2.234890 -3.310283 -0.951570

F -1.565322 -3.868042 1.039291

Cl 5.807608 -0.823912 0.001931

H -1.224857 4.235786 -1.230658

C -2.451001 1.392290 2.048986

H -3.472234 1.777518 2.125127

H -2.499821 0.299397 2.027258

H -1.890297 1.701296 2.937296

**TS2'**

C -2.289422 -0.557997 -0.646682

C -0.235303 -0.029071 0.351394

C -0.434314 -1.395154 1.613529

C -1.829733 -1.490187 1.764520

C -2.598213 -1.924607 0.678066

H 0.069812 -2.190328 1.061927

H 0.153628 -1.036074 2.459973

H -2.205698 -2.738324 0.066827

H -3.684780 -1.957725 0.783273

H -0.637339 0.787518 0.967902

C 1.239583 0.074936 0.151068

C 1.997618 0.895873 0.988740

C 1.891953 -0.654401 -0.846620

C 3.376293 1.000799 0.839435

H 1.497497 1.470101 1.770039

C 3.267652 -0.563277 -1.011925

H 1.292768 -1.282026 -1.503854

C 3.999110 0.265582 -0.163705

H 3.964614 1.645713 1.486929

H 3.776858 -1.123644 -1.792167

C -3.048306 0.593344 -0.013416

O -2.797518 1.112102 1.057328

O -4.035280 1.030229 -0.825717

N -0.941201 -0.475725 -0.715454

C -4.754534 2.162557 -0.329208

H -5.533867 2.361274 -1.065698

H -5.191028 1.941685 0.649520

H -4.088836 3.025147 -0.229655

Cl 5.729543 0.382764 -0.366349

H -2.309938 -0.888518 2.534218

C -2.921556 -1.130493 -1.898447

H -3.955264 -1.448472 -1.728618

H -2.934387 -0.383691 -2.699030

H -2.330455 -1.985620 -2.238877

**Int-II**

C -0.914322 2.301809 1.021505

C 0.871338 1.288409 -0.443035

C 0.998208 2.654628 -1.122230

C -0.258240 3.459776 -0.889906

C -0.485272 3.707771 0.588266

H -0.015487 0.481820 1.213680

H 1.837061 3.236950 -0.716343

H 1.192441 2.510937 -2.190855

H 0.424802 4.019102 1.108538

H -1.279206 4.444103 0.765348

H 0.229639 0.672225 -1.088988

C 2.185820 0.568769 -0.296935

C 2.336925 -0.717515 -0.816695

C 3.257232 1.158970 0.378218

C 3.544217 -1.398584 -0.689282

H 1.492219 -1.204456 -1.304277

C 4.470025 0.497239 0.511666

H 3.135526 2.147260 0.823419

C 4.601844 -0.778795 -0.033520

H 3.659942 -2.403139 -1.086827

H 5.306453 0.954773 1.033925

C -1.842570 2.019860 -0.131958

O -1.450295 2.574282 -1.237927

O -2.910646 1.332662 -0.063311

N 0.253926 1.424104 0.896936

C -3.634026 1.010631 -1.293061

H -4.600286 0.649786 -0.949611

H -3.726314 1.911611 -1.902196

H -3.064230 0.222428 -1.789747

O -1.300123 -0.773732 -1.053169

S -1.229129 -1.748569 0.070292

O -1.086864 -1.096678 1.395291

O -0.393937 -2.921397 -0.175964

C -2.955346 -2.398036 0.116610

F -3.821394 -1.408722 0.432750

F -3.311724 -2.871213 -1.085281

F -3.097530 -3.371167 1.017560

Cl 6.125040 -1.616012 0.124707

H -0.383643 4.317874 -1.553445

C -1.538457 2.148770 2.386135

H -2.393793 2.821480 2.513430

H -1.875060 1.116555 2.530576

H -0.790662 2.377886 3.150856

**TS3**

C -1.594359 -3.157981 -0.551156

C 0.088976 -1.503395 0.283868

C -0.290539 -1.949235 1.704264

C -1.654534 -2.612542 1.702883

C -1.668290 -3.839344 0.810145

H -0.134594 -2.199227 -1.627210

H 0.442177 -2.670031 2.091632

H -0.279048 -1.069994 2.357648

H -0.817595 -4.503845 0.983935

H -2.602670 -4.403787 0.916861

H -0.492285 -0.583332 0.086150

C 1.543748 -1.127264 0.175904

C 1.929054 0.172025 0.515655

C 2.515961 -2.041411 -0.232274

C 3.259547 0.563916 0.436083

H 1.173335 0.883887 0.845152

C 3.852228 -1.663829 -0.317886

H 2.219720 -3.054768 -0.499075

C 4.211201 -0.360725 0.014282

H 3.551865 1.581003 0.685269

H 4.612044 -2.369149 -0.644812

C -2.529883 -1.982139 -0.298487

O -2.582666 -1.693323 0.985764

O -3.035273 -1.249920 -1.166609

N -0.248534 -2.573055 -0.685393

C -3.103067 0.516020 -0.719037

H -4.142672 0.709194 -0.949271

H -2.800138 0.406680 0.310651

H -2.367081 0.768554 -1.467254

O -1.536570 3.986970 1.418330

S -1.585062 2.764805 0.630658

O -2.848237 2.592224 -0.168043

O -1.133024 1.507731 1.267806

C -0.323891 3.006153 -0.695219

F -0.623130 4.036163 -1.484280

F 0.891808 3.208285 -0.171139

F -0.252369 1.894817 -1.469294

Cl 5.883215 0.120350 -0.108933

H -2.092034 -2.735807 2.695592

C -1.934381 -3.990696 -1.760572

H -2.903964 -4.484838 -1.640923

H -1.984593 -3.368959 -2.661567

H -1.167644 -4.757165 -1.909974

**4a**

C 2.728299 0.847455 -0.022709

C 2.971724 0.444321 1.423906

C 2.534155 -1.008526 1.334093

C 1.021516 -1.084619 1.193547

C 0.572253 -0.382325 -0.097587

H 4.036208 0.522552 1.677945

H 2.921446 -1.660855 2.122194

H 0.675294 -2.124860 1.169891

H 0.556658 -0.602954 2.065126

H 0.841081 -1.062532 -0.929474

H 2.382036 1.034736 2.132645

C -0.924051 -0.197275 -0.121914

C -1.743817 -1.213545 -0.616907

C -1.521722 0.953379 0.396758

C -3.129077 -1.097081 -0.595296

H -1.290512 -2.115069 -1.031459

C -2.905283 1.092496 0.419288

H -0.883542 1.750121 0.775358

C -3.697979 0.061820 -0.075822

H -3.764682 -1.889285 -0.982692

H -3.369560 1.991467 0.817307

Cl -5.435458 0.228874 -0.054410

N 1.262203 0.911886 -0.221371

H 1.080893 1.274970 -1.156856

C 3.271663 -0.394676 -0.747404

O 3.143622 -1.465956 0.090471

O 3.675851 -0.492835 -1.877581

C 3.362983 2.130297 -0.494829

H 2.882394 2.991863 -0.019061

H 4.431788 2.149381 -0.257494

H 3.268086 2.226397 -1.582721

**TfOH**

S 0.850822 -0.145651 0.082805

O 1.214013 -1.372452 -0.577962

O 1.238584 0.210286 1.432194

C -0.993923 0.004515 -0.003287

O 1.243450 1.071079 -0.918565

H 1.397125 1.866471 -0.376138

F -1.417013 -0.267896 -1.229815

F -1.527534 -0.851334 0.860202

F -1.346020 1.248735 0.323573

**TfOMe**

S -0.437628 0.535589 0.024195

O -0.460460 1.519035 -1.038131

O -0.767979 0.874911 1.395032

C 1.231406 -0.272552 0.005133

O -1.239292 -0.805185 -0.417597

F 1.488167 -0.757443 -1.202287

F 2.125488 0.662311 0.311520

F 1.278611 -1.248558 0.904358

C -2.629627 -0.830228 -0.033621

H -2.975415 -1.825636 -0.310819

H -2.730783 -0.674164 1.044120

H -3.190964 -0.069833 -0.586255

**TS4**

C -1.544885 1.750529 0.793549

C 0.588462 1.788744 -0.407956

C 0.700070 3.399749 -0.368199

C -0.650763 3.940864 -0.283849

C -1.414145 3.765585 0.855652

H 0.064121 0.611438 1.269375

H 1.302866 3.642243 0.512530

H 1.239274 3.703706 -1.269180

H -0.929773 3.735652 1.830274

H -2.440992 4.132015 0.871377

H 0.098990 1.551592 -1.358710

C 1.924840 1.108869 -0.312883

C 2.209622 0.045968 -1.172634

C 2.857155 1.458745 0.666998

C 3.406019 -0.652956 -1.065702

H 1.465956 -0.270604 -1.901615

C 4.064525 0.781002 0.777076

H 2.645720 2.268078 1.366604

C 4.327986 -0.270174 -0.097233

H 3.610604 -1.500696 -1.713264

H 4.793682 1.054036 1.535115

C -2.363745 1.585894 -0.487775

O -2.084064 2.074154 -1.561569

O -3.385066 0.775564 -0.239909

N -0.240520 1.413968 0.691694

C -3.985518 0.179860 -1.405578

H -4.810282 -0.421249 -1.022901

H -4.338081 0.955104 -2.091094

H -3.233274 -0.452562 -1.885739

O -0.978558 -0.734342 -0.999768

S -0.508783 -1.757481 -0.032920

O -0.194366 -1.199789 1.315984

O 0.443459 -2.734941 -0.553241

C -2.049292 -2.718856 0.293015

F -2.978299 -1.931168 0.878062

F -2.575005 -3.168274 -0.855130

F -1.824241 -3.759136 1.096663

Cl 5.838405 -1.127129 0.038288

H -1.137177 4.262062 -1.201037

C -2.190494 1.310541 2.072804

H -3.179690 1.756911 2.198141

H -2.309434 0.220357 2.051322

H -1.563564 1.570868 2.930715

**Int-III**

C 1.962944 -1.078232 -0.963868

C 0.070397 -1.594121 0.578205

C 0.515662 -0.897181 1.890572

C 1.928233 -1.234211 2.229800

C 2.963114 -0.402502 2.075946

H 0.388835 0.188742 1.788708

H -0.172774 -1.244864 2.672556

H 2.824134 0.615510 1.712952

H 3.976994 -0.711583 2.327558

H 0.417597 -2.629396 0.581944

C -1.415881 -1.563764 0.329385

C -2.076022 -2.747376 -0.006746

C -2.152129 -0.379907 0.430786

C -3.447126 -2.762671 -0.236124

H -1.510150 -3.675347 -0.090068

C -3.521602 -0.377426 0.197567

H -1.658134 0.559641 0.667594

C -4.157484 -1.570858 -0.133352

H -3.962135 -3.684210 -0.494630

H -4.093437 0.543863 0.266944

C 2.773002 -2.280055 -0.542180

O 2.316515 -3.356328 -0.223301

O 4.073617 -1.992153 -0.637089

N 0.765276 -0.895009 -0.520322

C 4.948945 -3.043461 -0.201183

H 5.957814 -2.658236 -0.343076

H 4.760531 -3.266026 0.853821

H 4.783111 -3.945958 -0.794938

Cl -5.874429 -1.572514 -0.426748

H 2.103309 -2.255934 2.580855

C 2.552255 -0.124474 -1.932684

H 3.273751 0.508257 -1.399660

H 3.101805 -0.654205 -2.716800

H 1.794870 0.530653 -2.369396

H 0.297049 0.005419 -0.835713

O 1.310867 3.439195 -1.625607

S 0.746686 2.508326 -0.655374

O 1.681703 1.869317 0.294995

O -0.224001 1.514766 -1.226841

C -0.337438 3.528424 0.431018

F 0.369279 4.484058 1.036327

F -1.323315 4.095889 -0.265760

F -0.894030 2.754137 1.388790

**TS5**

C -2.094371 0.738989 0.534703

C -0.074586 1.900157 -0.362059

C 0.095278 3.434796 -0.353143

C 0.594195 3.971419 0.948255

C 1.829254 4.423663 1.161427

H 0.787540 3.702490 -1.162907

H -0.885660 3.863623 -0.598474

H 2.571918 4.446539 0.363239

H 2.150543 4.793257 2.132830

H -0.563467 1.634042 -1.302256

C 1.257208 1.187659 -0.317667

C 1.891495 0.900118 -1.529181

C 1.925626 0.876838 0.867433

C 3.147719 0.308276 -1.569766

H 1.386853 1.139491 -2.466123

C 3.176947 0.271030 0.850769

H 1.476842 1.088607 1.836645

C 3.778918 -0.007675 -0.371119

H 3.630051 0.081859 -2.517203

H 3.683713 0.017228 1.777981

C -2.992871 1.167773 -0.627566

O -2.801334 2.138323 -1.325700

O -4.053932 0.364683 -0.715020

N -0.989631 1.502354 0.722051

C -4.973181 0.698596 -1.763635

H -5.769801 -0.041511 -1.695503

H -5.363764 1.710136 -1.620172

H -4.472567 0.642666 -2.734141

Cl 5.347397 -0.768039 -0.402372

H -0.119142 3.960765 1.777064

C -2.866267 0.437262 1.794503

H -3.582936 -0.367463 1.625055

H -2.195368 0.151797 2.609419

H -3.415846 1.336768 2.095153

O -1.653976 -0.796916 -0.146079

S -1.136397 -1.871187 0.869690

O -2.193675 -2.772757 1.296929

O -0.248842 -1.273260 1.865389

C -0.064642 -2.835854 -0.297518

F -0.747206 -3.144542 -1.398714

F 1.012256 -2.129747 -0.628467

F 0.316799 -3.957749 0.311297

H -0.521801 1.288372 1.596491

**Int-IV**

C 1.670966 1.089242 -0.434870

C -0.546983 1.975808 0.246356

C -1.075511 3.406365 0.019653

C -1.611614 3.655010 -1.350985

C -2.889677 3.895475 -1.640759

H -1.850730 3.608580 0.771308

H -0.232303 4.082341 0.219487

H -3.652407 3.928853 -0.862415

H -3.227685 4.073740 -2.659456

H -0.030770 1.977666 1.210734

C -1.647538 0.943835 0.322939

C -2.034441 0.455784 1.572931

C -2.330529 0.485665 -0.807344

C -3.066381 -0.464617 1.705942

H -1.504592 0.791864 2.464809

C -3.358291 -0.443573 -0.698657

H -2.061528 0.846648 -1.799548

C -3.718941 -0.908630 0.561481

H -3.352862 -0.848797 2.681308

H -3.876966 -0.805814 -1.582124

C 2.425461 1.858878 0.658696

O 2.101794 2.946019 1.079450

O 3.527602 1.197614 1.024117

N 0.473266 1.663525 -0.775388

C 4.317350 1.860175 2.020620

H 5.143133 1.182092 2.234465

H 4.686333 2.816763 1.639407

H 3.719756 2.041284 2.918122

Cl -5.011851 -2.071087 0.709006

H -0.877280 3.628325 -2.161159

C 2.561018 0.793346 -1.616751

H 3.453097 0.254939 -1.295216

H 2.031415 0.199424 -2.365670

H 2.867840 1.741832 -2.071181

O 1.468829 -0.324602 0.398527

S 0.711632 -1.541695 -0.260598

O 0.266856 -1.229573 -1.613746

H 0.081782 1.235365 -1.609113

C 2.127678 -2.721601 -0.451538

F 2.994920 -2.256810 -1.356915

F 1.657719 -3.895540 -0.866881

F 2.757107 -2.882737 0.709011

O -0.169688 -2.151379 0.711304

**Int-IV'**

C -1.151556 1.082784 -0.105053

C 1.162811 0.859251 0.810577

C 1.089023 0.673579 2.339323

C 2.235654 1.318120 3.046642

C 2.127276 2.386376 3.835237

H 0.144286 1.079614 2.717784

H 1.056397 -0.409098 2.534197

H 1.160166 2.849470 4.036039

H 2.989781 2.836804 4.321576

H 1.228350 1.940792 0.590621

C 2.399759 0.199972 0.250863

C 3.503789 0.957103 -0.139847

C 2.467637 -1.193706 0.147380

C 4.656082 0.348130 -0.626247

H 3.462612 2.043588 -0.063981

C 3.607562 -1.819083 -0.344463

H 1.609545 -1.798120 0.444214

C 4.695232 -1.038645 -0.726354

H 5.514170 0.939625 -0.933353

H 3.656133 -2.901184 -0.432484

C -0.985123 1.852464 -1.438705

O -0.005501 1.749719 -2.141427

O -2.041100 2.629797 -1.699957

N -0.016662 0.257140 0.160731

C -1.962186 3.343238 -2.943187

H -2.884248 3.920465 -3.008124

H -1.088692 4.001131 -2.947562

H -1.883622 2.639999 -3.776769

Cl 6.129256 -1.810897 -1.350559

H 3.222757 0.883778 2.867901

C -1.580176 1.993878 1.023633

H -2.472755 2.543004 0.716291

H -1.811902 1.408561 1.915722

H -0.794266 2.721036 1.260851

O -2.277425 0.175392 -0.437212

S -2.350791 -1.223841 0.408769

O -2.289511 -0.991648 1.839903

O -1.597548 -2.274711 -0.245596

C -4.132232 -1.509706 -0.028809

F -4.900110 -0.574206 0.520062

F -4.292951 -1.503638 -1.347142

F -4.470257 -2.702755 0.454828

H 0.280472 -0.138285 -0.730187

**TS6**

C -0.839098 0.784461 0.913544

C 1.157978 -0.783722 1.179040

C 0.784454 -2.275213 1.298125

C -0.081992 -2.627314 2.463646

C -1.172733 -3.390445 2.417367

H 0.319984 -2.610644 0.362497

H 1.728728 -2.832110 1.409066

H -1.555415 -3.777542 1.475249

H -1.729230 -3.646499 3.316582

H 1.212179 -0.361190 2.196772

C 2.513244 -0.568282 0.537594

C 3.286196 0.537120 0.901837

C 2.981788 -1.403208 -0.479244

C 4.505712 0.799335 0.289208

H 2.922932 1.211767 1.678017

C 4.197837 -1.156513 -1.107709

H 2.390287 -2.261659 -0.793836

C 4.951851 -0.055800 -0.714295

H 5.105545 1.657295 0.580673

H 4.558883 -1.808092 -1.899107

C -0.565226 2.281961 0.660950

O 0.377215 2.683582 0.015699

O -1.490098 3.058527 1.232113

N 0.190398 -0.004218 0.373464

C -1.327799 4.462078 0.980721

H -2.151246 4.949612 1.502098

H -0.363922 4.808997 1.363925

H -1.376390 4.659265 -0.093774

Cl 6.478189 0.260162 -1.495207

H 0.260437 -2.247009 3.432792

C -1.255978 0.472450 2.325836

H -2.137781 1.062553 2.580693

H -1.490916 -0.592102 2.422081

H -0.457607 0.726830 3.033356

O -2.120473 0.631944 0.074174

S -2.220667 -0.632438 -0.920877

O -2.177688 -1.892932 -0.201179

O -1.468156 -0.419639 -2.142428

C -4.002670 -0.311988 -1.322232

F -4.738206 -0.324671 -0.215164

F -4.141583 0.858077 -1.938446

F -4.408963 -1.289776 -2.130718

H 0.663201 0.512251 -0.363218

**Int-V**

C 0.683511 -2.298362 -0.110055

C -1.162763 -1.192932 1.140574

C -0.522323 -0.332393 2.254091

C 0.475612 -1.107520 3.051552

C 1.798785 -0.996199 2.930260

H -0.064795 0.551386 1.789766

H -1.344870 0.021619 2.890080

H 2.255819 -0.301620 2.226778

H 2.470319 -1.603909 3.534809

H -1.379541 -2.193403 1.535324

C -2.433658 -0.615402 0.568049

C -3.606304 -1.371342 0.607241

C -2.468969 0.672429 0.022917

C -4.804797 -0.861335 0.120992

H -3.588842 -2.376845 1.028754

C -3.658007 1.190134 -0.476557

H -1.561561 1.272976 -0.032907

C -4.817047 0.419911 -0.420203

H -5.718093 -1.448984 0.154132

H -3.687613 2.186466 -0.908902

C 1.595116 -2.093411 -1.298851

O 1.239249 -1.487237 -2.280918

O 2.765774 -2.689223 -1.096399

N -0.167402 -1.347805 0.066813

C 3.765780 -2.326022 -2.070850

H 4.680719 -2.815878 -1.739638

H 3.467327 -2.675705 -3.062547

H 3.873563 -1.237475 -2.072810

Cl -6.308361 1.070304 -1.041273

H 0.056766 -1.831853 3.757752

C 0.793309 -3.535596 0.689363

H 0.701521 -4.409176 0.032054

H 1.801686 -3.576714 1.115186

H 0.067114 -3.605664 1.500564

H -0.003037 -0.469660 -0.497848

O 2.651738 1.604478 -1.963483

S 2.052244 1.191091 -0.700809

O 2.663501 0.020627 -0.027144

O 0.553383 1.134476 -0.676429

C 2.380998 2.582517 0.463423

F 3.692130 2.816052 0.561567

F 1.775071 3.695850 0.045305

F 1.915795 2.290835 1.695312

**TS7**

C 0.960714 -2.297605 0.074317

C -1.196221 -1.513241 0.797344

C -0.534754 -0.631628 2.040059

C 0.640365 -1.363243 2.434657

C 1.751493 -1.403285 1.605095

H -0.317369 0.341395 1.587321

H -1.312605 -0.561044 2.804662

H 2.048422 -0.507129 1.050303

H 2.587359 -2.044125 1.884479

H -1.422999 -2.501493 1.211491

C -2.470342 -0.854496 0.357125

C -3.681237 -1.505121 0.601485

C -2.479025 0.403153 -0.255333

C -4.890827 -0.927305 0.235447

H -3.683260 -2.484467 1.080998

C -3.681915 0.988193 -0.631241

H -1.545522 0.934461 -0.445608

C -4.878293 0.320049 -0.380746

H -5.833215 -1.435094 0.422057

H -3.693469 1.962261 -1.112976

C 1.996224 -2.046439 -1.021619

O 1.699574 -1.593232 -2.099812

O 3.206192 -2.446544 -0.633745

N -0.196964 -1.626274 -0.200479

C 4.253859 -2.110386 -1.567307

H 5.170833 -2.492167 -1.118887

H 4.062121 -2.585199 -2.533211

H 4.283936 -1.023514 -1.678268

Cl -6.385000 1.060714 -0.837077

H 0.538219 -2.108255 3.229293

C 0.869874 -3.739806 0.517692

H 0.539779 -4.352149 -0.330800

H 1.850143 -4.103764 0.833691

H 0.165428 -3.888372 1.340659

H -0.024679 -0.755187 -0.730130

O 2.147605 2.394112 -1.880864

S 1.962043 1.557552 -0.703668

O 3.014865 0.558597 -0.405719

O 0.584387 1.007903 -0.499619

C 2.094621 2.701259 0.738142

F 3.340833 3.158600 0.875751

F 1.268976 3.744888 0.614554

F 1.764606 2.046237 1.874656

(*S*)-**IM1**

C -0.628733 -1.224885 2.036838

C -1.222886 -1.878266 3.169915

H -0.865757 -1.801419 4.190703

C -2.374456 -2.579894 2.723084

H -3.064055 -3.130644 3.353626

C -1.435702 -1.541730 0.869062

C -2.503861 -2.386002 1.319463

H -3.302930 -2.765059 0.690061

C 2.043040 1.283029 1.911070

C 2.401196 0.379889 3.098499

H 2.519335 0.916504 4.045047

N 0.883896 0.598828 1.303863

C 0.513065 -0.330941 2.129451

O 1.257502 -0.493293 3.238731

H 3.279570 -0.254646 2.934432

H 2.857039 1.311830 1.170440

C 1.731873 2.723022 2.347004

C 1.043611 3.523551 1.255114

C 3.019560 3.401993 2.796366

H 1.046968 2.657594 3.210983

H 0.069799 3.096937 0.993112

H 0.889608 4.562209 1.572319

H 1.657165 3.545883 0.340651

H 3.534104 2.850072 3.592905

H 3.720755 3.494318 1.955015

H 2.824025 4.410993 3.174615

P -1.083168 -0.997848 -0.816374

C -0.191615 -2.417071 -1.542942

C 0.105752 -2.389451 -2.913660

C 0.322019 -3.453205 -0.754882

C 0.891704 -3.383544 -3.484175

H -0.285921 -1.582003 -3.535156

C 1.117727 -4.443095 -1.329779

H 0.096790 -3.492110 0.310673

C 1.405317 -4.409490 -2.690939

H 1.108438 -3.357374 -4.550292

H 1.507440 -5.248167 -0.709228

H 2.026158 -5.183680 -3.136831

C -2.706480 -0.984926 -1.640243

C -3.312197 0.246137 -1.919562

C -3.371509 -2.171626 -1.975663

C -4.577612 0.288841 -2.499584

H -2.784261 1.169986 -1.677321

C -4.634945 -2.124773 -2.555410

H -2.891991 -3.133526 -1.790681

C -5.241030 -0.895372 -2.811697

H -5.045355 1.248440 -2.712006

H -5.147574 -3.049475 -2.813015

H -6.228920 -0.861397 -3.266630

Cu 0.206713 0.772847 -0.640072

O -0.950037 2.757323 -1.140624

C -0.268010 3.370196 -1.950034

O -0.465159 4.635197 -2.288962

C 0.865945 2.740911 -2.729872

N 1.490864 1.714748 -1.897395

C 2.772011 1.587356 -2.004210

H 3.335089 2.300430 -2.626340

C 3.551313 0.574008 -1.319706

C 4.910592 0.820170 -1.068596

C 2.986948 -0.633279 -0.874175

C 5.673296 -0.076635 -0.335604

H 5.367293 1.740500 -1.431788

C 3.741722 -1.545210 -0.154997

H 1.950861 -0.867910 -1.116390

C 5.078423 -1.251433 0.126534

H 6.720172 0.121485 -0.123341

H 3.307150 -2.484468 0.179758

C -1.569424 5.291407 -1.635016

H -2.507478 4.793778 -1.894050

H -1.429105 5.265334 -0.551295

C -2.966357 1.239584 1.512490

C -2.419148 1.361041 2.824351

H -1.501634 1.881552 3.083285

C -3.243648 0.622166 3.721564

H -3.070573 0.488337 4.783869

C -4.135771 0.421199 1.599549

H -4.757300 0.106559 0.766835

C -4.305582 0.042650 2.965356

H -5.080831 -0.608818 3.354087

H -2.533457 1.656266 0.605864

Fe -2.509511 -0.591531 2.261478

Cl 6.015980 -2.371929 1.054169

H 1.589962 3.520448 -3.009972

C 0.282556 2.104619 -3.993294

H -0.197746 2.861888 -4.621409

H -0.459744 1.343069 -3.724588

H 1.078350 1.624811 -4.571796

H -1.556967 6.314701 -2.006211

(*R*)-**IM1**

C -0.647764 -1.791781 1.614862

C -1.276591 -2.714535 2.518933

H -0.915797 -2.956625 3.512278

C -2.462078 -3.199060 1.904506

H -3.180644 -3.872614 2.358588

C -1.467840 -1.717257 0.418285

C -2.578931 -2.601303 0.619203

H -3.394432 -2.744407 -0.082583

C 2.292929 0.355643 2.085555

C 2.541562 -0.873574 2.970944

H 2.708387 -0.634389 4.026047

N 1.037874 0.018070 1.377237

C 0.577343 -1.069181 1.915506

O 1.313607 -1.629081 2.891311

H 3.350340 -1.524738 2.619644

H 3.088975 0.471363 1.335236

C 2.209154 1.655454 2.897838

C 1.714076 2.825636 2.066062

C 3.577730 1.964711 3.493402

H 1.492729 1.481036 3.719587

H 0.712691 2.642736 1.662094

H 1.680257 3.742861 2.665907

H 2.393825 3.009824 1.220636

H 3.964002 1.152890 4.121708

H 4.313401 2.140097 2.695306

H 3.543616 2.866847 4.113327

P -1.097473 -0.716957 -1.038864

C -0.291442 -1.905149 -2.168101

C -0.203112 -1.604320 -3.534892

C 0.366423 -3.038924 -1.676131

C 0.523961 -2.424468 -4.390479

H -0.716330 -0.726510 -3.930260

C 1.096161 -3.856323 -2.537050

H 0.301562 -3.290626 -0.617143

C 1.180151 -3.549356 -3.892345

H 0.577228 -2.186159 -5.450950

H 1.594103 -4.742060 -2.145875

H 1.750798 -4.188999 -4.562115

C -2.729059 -0.409416 -1.790744

C -3.298693 0.864373 -1.679984

C -3.440494 -1.423162 -2.446143

C -4.567666 1.116157 -2.195023

H -2.738712 1.653972 -1.177388

C -4.707003 -1.168811 -2.961851

H -2.994874 -2.411725 -2.559751

C -5.273518 0.098852 -2.832993

H -5.006317 2.108476 -2.102822

H -5.253288 -1.960962 -3.469628

H -6.263735 0.294740 -3.239077

Cu 0.279236 0.870516 -0.359354

O -0.914852 2.839528 -0.048578

C -0.552661 3.656213 -0.882290

O -1.229007 4.762232 -1.165847

C 0.683572 3.510339 -1.756762

N 1.391857 2.290066 -1.351724

C 2.666392 2.224360 -1.541660

H 3.226882 3.094088 -1.905969

C 3.452654 1.040896 -1.238720

C 4.782180 1.200076 -0.818296

C 2.912143 -0.252780 -1.310567

C 5.532183 0.109332 -0.401890

H 5.221788 2.196912 -0.784374

C 3.655900 -1.353216 -0.912655

H 1.909066 -0.401089 -1.711009

C 4.955237 -1.161227 -0.439219

H 6.554033 0.232218 -0.053081

H 3.238451 -2.355411 -0.977221

C -2.439724 4.962254 -0.411818

H -3.136777 4.138104 -0.590374

H -2.212251 5.019574 0.655384

C -2.895897 0.825794 1.856271

C -2.323759 0.547492 3.133122

H -1.383416 0.943666 3.505623

C -3.163089 -0.391337 3.801918

H -2.980458 -0.829696 4.776961

C -4.090524 0.051014 1.732376

H -4.736117 0.008676 0.860453

C -4.256097 -0.697065 2.937127

H -5.047839 -1.409865 3.140552

H -2.467836 1.480655 1.102678

Fe -2.496892 -1.159033 2.040015

Cl 5.875531 -2.527874 0.093694

H 0.284570 3.313925 -2.766823

C 1.498185 4.797691 -1.805278

H 1.994182 4.986441 -0.845618

H 0.841939 5.642120 -2.024506

H 2.258755 4.767448 -2.590192

H -2.851401 5.902664 -0.773999

(*S*)-**IM1’**

C 0.472417 2.200344 1.505501

C 1.203833 3.060018 2.396755

H 1.273884 4.137186 2.303852

C 1.849694 2.249343 3.366242

H 2.511922 2.605474 4.147888

C 0.679268 0.826809 1.946739

C 1.532494 0.890064 3.097646

H 1.878831 0.029751 3.663033

C -1.375294 3.112692 -1.543124

C -0.654302 4.382174 -1.087791

H 0.204241 4.628796 -1.729636

N -0.832003 2.084271 -0.624773

C -0.200711 2.712478 0.322503

O -0.132904 4.054576 0.214073

H -1.287683 5.265778 -0.984337

H -2.459370 3.180395 -1.346981

C -1.175987 2.817971 -3.032679

C -2.001876 1.631931 -3.507127

C -1.535233 4.052484 -3.855619

H -0.103629 2.593497 -3.177924

H -1.785281 0.714796 -2.947694

H -1.816200 1.429454 -4.568264

H -3.074883 1.841897 -3.392557

H -0.914197 4.923792 -3.617140

H -2.583877 4.336190 -3.691544

H -1.416075 3.853915 -4.925901

P -0.303414 -0.562340 1.335145

C -1.472985 -0.863038 2.707684

C -1.274129 -1.831284 3.697952

C -2.619001 -0.056545 2.752794

C -2.210846 -1.988495 4.716129

H -0.388496 -2.465771 3.669795

C -3.545002 -0.208632 3.779053

H -2.780686 0.692085 1.973693

C -3.343495 -1.178466 4.759407

H -2.052607 -2.745256 5.481862

H -4.428915 0.425370 3.810517

H -4.071726 -1.304848 5.557872

C 0.718199 -2.060330 1.259820

C 0.053746 -3.248103 0.914192

C 2.104036 -2.089723 1.442153

C 0.759734 -4.433808 0.756557

H -1.029704 -3.237677 0.784148

C 2.812647 -3.276277 1.263256

H 2.626719 -1.179590 1.729300

C 2.144655 -4.447989 0.918005

H 0.229279 -5.350074 0.501399

H 3.892307 -3.284450 1.403953

H 2.700177 -5.374149 0.784482

Cu -1.315224 0.111711 -0.505597

O -3.594152 0.495742 -0.695396

C -4.097411 -0.488114 -1.218275

O -5.374156 -0.582572 -1.562613

C -3.347469 -1.777382 -1.481706

N -1.930495 -1.453939 -1.660308

C -1.175131 -2.386074 -2.145022

H -1.594840 -3.380338 -2.364496

C 0.237949 -2.213953 -2.413479

C 1.039764 -3.348971 -2.608693

C 0.836044 -0.944317 -2.491919

C 2.405490 -3.235042 -2.817930

H 0.584992 -4.338465 -2.565978

C 2.191493 -0.815898 -2.742592

H 0.222640 -0.047433 -2.390787

C 2.975962 -1.964127 -2.875363

H 3.028503 -4.116855 -2.939931

H 2.654090 0.163646 -2.833863

C -6.189556 0.566399 -1.265423

H -5.817784 1.439678 -1.807649

H -6.174679 0.770267 -0.191753

H -7.192115 0.302098 -1.597723

C 3.411707 0.681968 0.032012

C 3.037835 1.968597 -0.454098

H 2.365115 2.167218 -1.285084

C 3.635369 2.952406 0.390129

H 3.509456 4.026826 0.307193

C 4.236613 0.866849 1.181064

H 4.663346 0.080507 1.795787

C 4.377103 2.269281 1.401357

H 4.917289 2.735768 2.217785

H 3.080370 -0.271370 -0.365674

Cl 4.682267 -1.798892 -3.113785

Fe 2.421581 1.744987 1.468931

H -3.753296 -2.245278 -2.389131

C -3.573464 -2.706473 -0.287324

H -3.046027 -3.654395 -0.442521

H -4.638298 -2.925424 -0.159381

H -3.197529 -2.242949 0.635009

(*R*)-**IM1’**

C -1.246071 2.537329 0.278577

C -1.296264 3.955162 0.499733

H -1.970011 4.640031 -0.002098

C -0.275441 4.294166 1.425907

H -0.029387 5.296668 1.758463

C -0.164728 1.991508 1.089883

C 0.408490 3.103391 1.794430

H 1.238703 3.037716 2.491174

C -2.858318 0.520141 -2.314292

C -3.737957 1.767776 -2.190024

H -3.896855 2.300947 -3.132191

N -1.934635 0.656404 -1.176617

C -2.041200 1.870745 -0.736405

O -2.980121 2.642376 -1.320344

H -4.705616 1.585978 -1.709165

H -3.444768 -0.401669 -2.176978

C -2.123823 0.449484 -3.664790

C -0.928716 -0.490468 -3.641056

C -3.108270 0.061759 -4.760425

H -1.752741 1.468869 -3.876089

H -0.167193 -0.152667 -2.928558

H -0.461029 -0.551908 -4.630381

H -1.220266 -1.509061 -3.348093

H -3.982745 0.723732 -4.795060

H -3.478101 -0.961767 -4.606460

H -2.635866 0.094386 -5.747798

P 0.069810 0.216134 1.400439

C -0.629188 0.052115 3.083056

C 0.159687 0.109232 4.237428

C -2.017896 -0.092917 3.203787

C -0.432599 0.014042 5.493510

H 1.240128 0.222981 4.153667

C -2.605194 -0.175832 4.462297

H -2.635889 -0.145682 2.306607

C -1.813505 -0.127440 5.608020

H 0.187964 0.053785 6.386415

H -3.685390 -0.280587 4.550004

H -2.273282 -0.200404 6.591506

C 1.849386 -0.104322 1.620313

C 2.212708 -1.411240 1.982817

C 2.856123 0.828477 1.358724

C 3.550364 -1.771926 2.078207

H 1.436450 -2.152642 2.181308

C 4.198622 0.459411 1.440702

H 2.589390 1.844856 1.080171

C 4.548792 -0.837917 1.799585

H 3.817113 -2.788628 2.361290

H 4.973568 1.194108 1.226421

H 5.596636 -1.124586 1.857271

Cu -1.006784 -0.878204 -0.205331

O -3.079556 -1.715869 0.523812

C -3.075699 -2.913688 0.287038

O -3.923945 -3.785280 0.815966

C -2.139194 -3.571700 -0.702261

N -0.883518 -2.814073 -0.766898

C 0.161486 -3.533346 -1.030452

H 0.038028 -4.623837 -1.118878

C 1.521057 -3.065217 -1.182832

C 2.542436 -4.031293 -1.158927

C 1.879278 -1.712999 -1.317566

C 3.877943 -3.668574 -1.219872

H 2.278598 -5.085084 -1.072574

C 3.209676 -1.337940 -1.390576

H 1.100166 -0.949403 -1.355646

C 4.202614 -2.317025 -1.328320

H 4.664744 -4.416913 -1.185568

H 3.496838 -0.293932 -1.488548

C -4.886837 -3.230711 1.733410

H -5.537659 -2.524391 1.211608

H -4.370428 -2.718235 2.549741

H -5.455699 -4.082014 2.103302

C 1.869840 2.508054 -1.460410

C 0.742897 2.967623 -2.200708

H 0.066877 2.343320 -2.778378

C 0.612202 4.371622 -1.983442

H -0.173719 5.007295 -2.377265

C 2.439917 3.628602 -0.782829

H 3.301741 3.610495 -0.123580

C 1.662667 4.779607 -1.107163

H 1.818709 5.781664 -0.722917

H 2.205698 1.478268 -1.381656

Cl 5.864652 -1.841876 -1.396703

Fe 0.498652 3.369424 -0.227146

H -1.950625 -4.603929 -0.372516

C -2.821522 -3.604990 -2.069996

H -3.044578 -2.586454 -2.411118

H -3.756870 -4.171989 -2.023690

H -2.163644 -4.076857 -2.807352

**IM2**

Ir 1.605152 -1.769286 -0.549346

O -0.776678 0.121107 1.199450

O -0.806297 0.231254 -1.356686

C -1.931083 -0.643670 1.176102

C -2.016344 -1.718472 2.082163

H -1.189602 -1.880349 2.770230

C -3.130789 -2.518062 2.089141

H -3.207694 -3.349586 2.788832

C -4.185780 -2.292625 1.173059

C -5.308692 -3.152380 1.112929

H -5.352290 -3.996015 1.801785

C -6.313863 -2.942685 0.199547

H -7.168382 -3.615603 0.160046

C -6.229366 -1.858904 -0.698168

H -7.013955 -1.707390 -1.437255

C -5.160065 -0.994926 -0.654539

H -5.102269 -0.168472 -1.359533

C -4.112987 -1.172129 0.284097

C -2.980266 -0.296524 0.339966

C -2.902855 0.945500 -0.456198

C -3.896953 1.972177 -0.351590

C -4.967390 1.907009 0.575649

H -5.041531 1.047389 1.237180

C -5.897457 2.916299 0.662237

H -6.703557 2.842593 1.390037

C -5.812148 4.047174 -0.174605

H -6.556928 4.837134 -0.099178

C -4.776596 4.153354 -1.071211

H -4.685754 5.030107 -1.712519

C -3.793334 3.139931 -1.176433

C -2.696498 3.264823 -2.062614

H -2.630778 4.149043 -2.695858

C -1.714752 2.308233 -2.104424

H -0.851531 2.402823 -2.758984

C -1.813197 1.166423 -1.282807

P 0.272830 -0.030704 -0.090673

N 1.214093 1.327121 0.136631

C 2.792744 -0.124710 -1.153488

H 2.504209 0.067743 -2.202748

H 3.868163 -0.359457 -1.181057

C 2.618993 1.166971 -0.349742

H 3.258987 1.113520 0.546683

C 3.027847 2.381321 -1.152642

C 2.286455 2.751181 -2.279997

H 1.409335 2.158288 -2.545703

C 2.655125 3.850070 -3.044866

H 2.067060 4.126794 -3.918993

C 3.780681 4.597455 -2.695599

H 4.072532 5.458563 -3.294306

C 4.526554 4.237514 -1.577522

H 5.404605 4.817666 -1.297506

C 4.147600 3.137965 -0.807736

H 4.721749 2.863582 0.078722

C 1.028498 2.550816 0.950295

H 1.446122 3.359280 0.330139

C 1.866195 2.486298 2.214185

C 2.635580 3.589590 2.587084

H 2.649914 4.468961 1.940524

C 3.388724 3.571329 3.758259

H 3.987076 4.438729 4.032518

C 3.384677 2.439812 4.569015

H 3.977409 2.418550 5.481961

C 2.624391 1.330645 4.201304

H 2.621760 0.440610 4.828903

C 1.870346 1.352980 3.032222

H 1.286627 0.480024 2.737708

C -0.401885 2.964060 1.267729

H -1.014991 3.021314 0.363338

H -0.888522 2.298198 1.983214

H -0.365740 3.966151 1.710916

C 3.408259 -2.929980 -0.072353

H 4.209087 -2.196646 0.040641

C 3.008947 -3.254301 -1.378615

H 3.510472 -2.716840 -2.187082

C 2.313214 -4.539639 -1.773236

H 2.656240 -4.838102 -2.771853

H 2.623841 -5.351795 -1.103715

C 0.781126 -4.406597 -1.790916

H 0.315157 -5.403554 -1.711452

H 0.471634 -4.009964 -2.767493

C 0.244702 -3.475393 -0.732992

H -0.762493 -3.096632 -0.933723

C 0.694868 -3.427632 0.601298

H 0.011191 -2.990791 1.327671

C 1.732854 -4.361272 1.190821

H 1.472749 -4.576144 2.235227

H 1.700832 -5.331994 0.679806

C 3.150308 -3.772269 1.146289

H 3.902573 -4.576206 1.219958

H 3.304351 -3.135247 2.027595

**IM3**

M06-L SCF energy in solvent: -5551.161136 a.u

Ir -1.759943 -0.205278 -1.656995

O 0.750116 -0.419024 0.882695

O 1.345986 0.829113 -1.252856

C 1.678172 -1.396031 0.566733

C 1.308708 -2.735962 0.801550

H 0.330631 -2.947953 1.234287

C 2.193488 -3.739842 0.500075

H 1.923642 -4.779509 0.681347

C 3.457421 -3.447287 -0.066052

C 4.348203 -4.478507 -0.449639

H 4.043896 -5.512008 -0.284528

C 5.560346 -4.189726 -1.028622

H 6.232340 -4.993135 -1.324590

C 5.925213 -2.846980 -1.254276

H 6.874515 -2.619163 -1.735942

C 5.089940 -1.820587 -0.878659

H 5.380851 -0.789998 -1.068598

C 3.840651 -2.080381 -0.261465

C 2.942122 -1.032079 0.126038

C 3.315833 0.397442 0.038142

C 4.476051 0.929104 0.692412

C 5.286188 0.154065 1.560062

H 5.021845 -0.884806 1.742867

C 6.388404 0.695853 2.179787

H 6.988199 0.077668 2.845304

C 6.744374 2.042765 1.966501

H 7.622597 2.458078 2.457300

C 5.969182 2.829988 1.149540

H 6.220156 3.878308 0.987621

C 4.820569 2.308593 0.507196

C 4.001141 3.127171 -0.306331

H 4.285247 4.167502 -0.462530

C 2.856078 2.626816 -0.871002

H 2.203017 3.237738 -1.490777

C 2.508944 1.276928 -0.666088

P 0.001260 0.442165 -0.325929

N -0.473898 1.763440 0.590016

C -2.048635 1.892010 -1.321381

H -1.440231 2.444433 -2.057606

H -3.095545 2.151431 -1.527895

C -1.704724 2.403897 0.080501

H -2.531960 2.131750 0.758270

C -1.589333 3.912674 0.082359

C -0.360208 4.556483 -0.081344

H 0.544688 3.951303 -0.141767

C -0.287149 5.944612 -0.141035

H 0.680720 6.429610 -0.262209

C -1.444729 6.712498 -0.040430

H -1.388084 7.798628 -0.085242

C -2.674769 6.080893 0.129307

H -3.584600 6.672626 0.217998

C -2.743567 4.692500 0.195887

H -3.707425 4.199039 0.335063

C -0.184568 2.060303 2.030417

H -0.559031 3.089159 2.137508

C -1.022247 1.202309 2.962399

C -2.297423 1.647532 3.332971

H -2.641298 2.622767 2.982350

C -3.121729 0.877773 4.149902

H -4.112132 1.241842 4.417800

C -2.675314 -0.350893 4.627312

H -3.321379 -0.959890 5.257199

C -1.397075 -0.793182 4.294024

H -1.032211 -1.749487 4.666623

C -0.577960 -0.023018 3.473379

H 0.417310 -0.383563 3.225945

C 1.292346 2.116463 2.374154

H 1.822440 2.780691 1.681572

H 1.783816 1.141036 2.348380

H 1.405274 2.520319 3.386976

C -2.159585 0.217105 -3.743043

H -2.642538 1.192893 -3.845733

C -0.745282 0.241359 -3.508313

H -0.303980 1.238527 -3.441027

C 0.231262 -0.830179 -3.979113

H 1.193251 -0.351724 -4.200544

H -0.108286 -1.252761 -4.934907

C 0.468590 -1.940230 -2.944747

H 0.776727 -2.877233 -3.439846

H 1.311652 -1.652056 -2.302015

C -0.721495 -2.205988 -2.056407

H -0.494718 -2.665672 -1.092169

C -2.036538 -2.306609 -2.515271

H -2.745714 -2.810512 -1.857911

C -2.428506 -2.280070 -3.980067

H -3.229528 -3.012855 -4.141360

H -1.585145 -2.633308 -4.586866

C -2.890786 -0.899467 -4.445602

H -2.791746 -0.809075 -5.541597

H -3.962803 -0.788770 -4.230318

C -2.498402 -1.920247 0.811954

H -1.527342 -1.817714 1.311576

H -2.441112 -2.770168 0.120009

C -2.886740 -0.642543 0.154971

H -2.849092 0.192085 0.859412

C -3.794556 -0.522374 -0.944521

H -4.269538 -1.414034 -1.356516

O -3.476551 -2.257574 1.836758

C -3.040418 -3.156102 2.715721

O -1.967347 -3.725306 2.711765

O -4.009325 -3.334862 3.631273

C -3.673141 -4.285299 4.637992

H -2.802726 -3.953082 5.214740

H -4.551528 -4.351460 5.281441

H -3.446980 -5.260558 4.195372

H -4.415297 0.372810 -1.011391

**TS-OA**

Ir 1.479434 -1.298901 -1.376376

O -0.784219 0.123581 0.964160

O -1.053454 0.819514 -1.468931

C -1.946665 -0.642801 0.904080

C -1.912509 -1.885802 1.560955

H -1.015230 -2.157354 2.130547

C -3.032082 -2.678197 1.509558

H -3.041246 -3.640660 2.020445

C -4.182566 -2.276516 0.787536

C -5.306959 -3.128108 0.666604

H -5.271018 -4.101674 1.155220

C -6.411915 -2.748014 -0.056670

H -7.266793 -3.415852 -0.144660

C -6.431017 -1.492133 -0.696080

H -7.297091 -1.199965 -1.287058

C -5.361989 -0.632709 -0.585681

H -5.388748 0.330366 -1.091280

C -4.213472 -0.985048 0.166125

C -3.072184 -0.123248 0.285503

C -3.062372 1.265252 -0.225965

C -4.036457 2.234027 0.190520

C -5.006785 1.958921 1.186905

H -5.015513 0.979886 1.659724

C -5.918448 2.912675 1.575140

H -6.644975 2.676841 2.350421

C -5.914690 4.193969 0.987929

H -6.644585 4.937775 1.301425

C -4.974566 4.503334 0.035662

H -4.943130 5.496661 -0.411922

C -4.010938 3.551249 -0.375643

C -3.007393 3.885109 -1.316307

H -3.003262 4.884321 -1.750504

C -2.035821 2.978245 -1.651174

H -1.240654 3.220509 -2.352779

C -2.061896 1.689535 -1.085223

P 0.118413 0.321141 -0.389593

N 1.092473 1.576691 0.097767

C 2.602524 0.501105 -1.559029

H 2.291658 0.987755 -2.496818

H 3.665518 0.249065 -1.679550

C 2.480198 1.506450 -0.418628

H 3.119494 1.187270 0.421020

C 2.974550 2.860364 -0.876474

C 2.194980 3.662954 -1.714897

H 1.196469 3.319656 -1.992499

C 2.673173 4.887459 -2.166802

H 2.053668 5.507730 -2.812568

C 3.942756 5.323622 -1.789505

H 4.317245 6.283167 -2.141212

C 4.726611 4.530025 -0.956794

H 5.715857 4.867926 -0.652473

C 4.241588 3.306516 -0.500033

H 4.844641 2.690019 0.168391

C 0.972740 2.521868 1.259715

H 1.417122 3.449804 0.869756

C 1.849882 2.055497 2.404546

C 2.965553 2.815142 2.765655

H 3.173162 3.743892 2.230197

C 3.814779 2.392089 3.785957

H 4.680974 2.994884 4.055344

C 3.560828 1.194163 4.447501

H 4.229489 0.851171 5.235248

C 2.448767 0.430009 4.096733

H 2.244566 -0.511360 4.603227

C 1.593267 0.860608 3.086516

H 0.727835 0.245325 2.840252

C -0.436182 2.880067 1.696879

H -1.054394 3.194359 0.849063

H -0.943150 2.071446 2.226700

H -0.359405 3.729357 2.385100

C 1.841181 -1.502525 -3.518372

H 2.690252 -0.863833 -3.774344

C 0.592634 -0.849283 -3.325770

H 0.600689 0.235251 -3.444172

C -0.753830 -1.499325 -3.603893

H -1.446381 -0.724574 -3.952552

H -0.645285 -2.197130 -4.445658

C -1.378132 -2.203243 -2.392713

H -2.051902 -3.012296 -2.720608

H -2.010971 -1.492738 -1.849205

C -0.371852 -2.766744 -1.425444

H -0.700645 -2.845279 -0.388860

C 0.749102 -3.492931 -1.795427

H 1.230798 -4.083423 -1.016315

C 1.056856 -3.900031 -3.220243

H 1.496897 -4.904578 -3.211010

H 0.114030 -3.998625 -3.773233

C 2.004841 -2.938895 -3.942708

H 1.876399 -3.023444 -5.034810

H 3.042711 -3.233788 -3.740798

C 1.782974 -2.144469 0.868748

H 1.320225 -1.550715 1.651428

H 1.357533 -3.130393 0.724723

C 2.958766 -1.741447 0.224128

H 3.454933 -0.837730 0.578501

C 3.382821 -2.347656 -0.998165

H 3.268208 -3.421876 -1.133279

O 2.351488 -3.239065 2.896067

C 1.507731 -2.809012 3.714390

O 0.451181 -2.160866 3.523405

O 1.849210 -3.094676 5.041405

C 0.890046 -2.695271 5.996862

H 0.735054 -1.606878 5.998288

H 1.279069 -3.012466 6.970069

H -0.086220 -3.164571 5.817611

H 4.261983 -1.930780 -1.491893

**IM3'**

Ir -1.881620 0.083025 -1.478160

O 0.807757 -0.513387 0.798570

O 1.242674 0.833905 -1.303794

C 1.651824 -1.496701 0.324961

C 1.241960 -2.834718 0.494087

H 0.306863 -3.039201 1.014347

C 2.054451 -3.847668 0.051178

H 1.755797 -4.886649 0.187751

C 3.282245 -3.565497 -0.593772

C 4.100492 -4.601042 -1.105571

H 3.765259 -5.631241 -0.984513

C 5.281716 -4.320414 -1.748916

H 5.899045 -5.126757 -2.140772

C 5.686077 -2.979922 -1.914132

H 6.609702 -2.755920 -2.445167

C 4.920974 -1.950425 -1.417936

H 5.241346 -0.920805 -1.561326

C 3.707528 -2.203408 -0.729347

C 2.888310 -1.152248 -0.203608

C 3.305168 0.266928 -0.228316

C 4.526304 0.726030 0.367207

C 5.380172 -0.124930 1.113650

H 5.107475 -1.170779 1.235651

C 6.532185 0.352633 1.694629

H 7.162849 -0.321917 2.271068

C 6.898619 1.706860 1.559587

H 7.815583 2.071246 2.018960

C 6.085517 2.564482 0.858896

H 6.344396 3.618894 0.760823

C 4.885116 2.110613 0.262329

C 4.023027 3.003269 -0.419797

H 4.316564 4.048406 -0.516549

C 2.825568 2.566411 -0.925046

H 2.136402 3.234079 -1.438435

C 2.463780 1.210900 -0.795006

P -0.033408 0.468459 -0.262108

N -0.248171 1.772181 0.793273

C -1.887427 2.209321 -0.978826

H -1.218792 2.727861 -1.681614

H -2.899271 2.604984 -1.145917

C -1.449316 2.551247 0.443166

H -2.262609 2.267493 1.128987

C -1.241195 4.040875 0.610061

C 0.007892 4.648558 0.460329

H 0.879434 4.020970 0.273423

C 0.141951 6.029652 0.573335

H 1.123918 6.487219 0.459929

C -0.970525 6.824614 0.838416

H -0.863882 7.904064 0.929981

C -2.219258 6.227405 0.998046

H -3.093376 6.838351 1.217901

C -2.348659 4.846804 0.889871

H -3.325512 4.377352 1.022623

C 0.141364 1.857591 2.236436

H -0.168425 2.879650 2.502329

C -0.684740 0.920644 3.102619

C -1.965127 1.321322 3.509482

H -2.308424 2.329320 3.269749

C -2.796225 0.467888 4.231174

H -3.791433 0.799770 4.522782

C -2.347981 -0.799868 4.590120

H -2.996148 -1.473023 5.149658

C -1.056122 -1.191385 4.244694

H -0.682375 -2.171306 4.537436

C -0.234617 -0.340553 3.509199

H 0.765711 -0.670052 3.239306

C 1.639949 1.797382 2.471704

H 2.151288 2.529568 1.835611

H 2.071426 0.815255 2.263162

H 1.852046 2.045369 3.518455

C -3.387813 0.377100 -2.992541

H -4.123807 1.124173 -2.676845

C -2.132084 0.886499 -3.473943

H -2.022721 1.971866 -3.434203

C -1.328377 0.228834 -4.584983

H -0.891955 1.004982 -5.226331

H -1.991527 -0.350314 -5.241500

C -0.217584 -0.640990 -4.012277

H 0.143615 -1.382761 -4.746582

H 0.643668 -0.008700 -3.764750

C -0.630071 -1.346027 -2.746044

H 0.209493 -1.705942 -2.145765

C -1.867626 -1.969689 -2.520153

H -1.864845 -2.743919 -1.752262

C -2.991343 -2.076001 -3.536781

H -3.519222 -3.023820 -3.370087

H -2.561087 -2.162545 -4.543031

C -3.998297 -0.918365 -3.474587

H -4.491145 -0.791362 -4.454501

H -4.806732 -1.187680 -2.778182

C -2.317591 -2.069763 0.830103

H -1.634540 -1.445263 1.423389

H -1.718764 -2.814455 0.290743

C -3.175829 -1.258917 -0.074696

H -3.836007 -1.825181 -0.732790

C -3.462418 0.070809 0.198688

H -3.064944 0.515181 1.110540

O -3.187947 -2.782390 1.754000

C -2.549331 -3.667292 2.517695

O -1.376769 -3.976903 2.452550

O -3.439156 -4.171671 3.386937

C -2.881393 -5.126459 4.287253

H -2.133057 -4.656450 4.935009

H -3.720326 -5.491284 4.881023

H -2.407649 -5.948813 3.742650

H -4.340161 0.554271 -0.227044

**TS-OA'**

Ir 1.248311 -2.008629 -0.714154

O -0.754491 0.410022 0.959315

O -1.036835 0.209844 -1.560398

C -1.991341 -0.192195 1.162096

C -2.071239 -1.131675 2.208115

H -1.189223 -1.287725 2.833918

C -3.267439 -1.767471 2.429410

H -3.359473 -2.490342 3.239465

C -4.395668 -1.511191 1.611724

C -5.610697 -2.215361 1.791687

H -5.663882 -2.958214 2.587417

C -6.696027 -1.978144 0.982757

H -7.622865 -2.528817 1.132377

C -6.602331 -1.025517 -0.051709

H -7.454898 -0.852774 -0.706104

C -5.440821 -0.315133 -0.247126

H -5.380588 0.411154 -1.054691

C -4.309053 -0.518050 0.582210

C -3.079683 0.196999 0.395240

C -2.946658 1.295325 -0.588824

C -3.797601 2.450948 -0.568993

C -4.764010 2.669797 0.444963

H -4.865637 1.941610 1.245938

C -5.560919 3.791543 0.439103

H -6.289015 3.936355 1.235001

C -5.437489 4.757426 -0.579905

H -6.077463 5.637428 -0.575818

C -4.493062 4.591011 -1.563534

H -4.368461 5.339503 -2.345795

C -3.647355 3.456370 -1.579516

C -2.641314 3.311434 -2.563771

H -2.541152 4.077154 -3.332082

C -1.784607 2.242166 -2.532548

H -0.986094 2.125161 -3.261990

C -1.935895 1.259852 -1.535973

P 0.121402 0.017234 -0.374304

N 1.228326 1.248322 -0.369562

C 2.636534 -0.542842 -1.409183

H 2.456236 -0.466129 -2.491458

H 3.662449 -0.918271 -1.295988

C 2.589871 0.869685 -0.820745

H 3.241008 0.924889 0.065617

C 3.130564 1.851833 -1.837147

C 2.379167 2.194459 -2.965967

H 1.379287 1.769742 -3.081409

C 2.886000 3.074320 -3.914841

H 2.287268 3.340521 -4.784609

C 4.160152 3.618135 -3.750846

H 4.557627 4.309858 -4.491335

C 4.918312 3.276076 -2.634942

H 5.911908 3.699765 -2.499222

C 4.403382 2.399551 -1.681911

H 4.985187 2.147999 -0.794398

C 1.170931 2.648058 0.175310

H 1.472992 3.278078 -0.676156

C 2.210808 2.820439 1.263858

C 3.193104 3.802556 1.126765

H 3.206043 4.422576 0.228430

C 4.158243 3.979406 2.115037

H 4.920700 4.748232 1.996600

C 4.153529 3.162671 3.242572

H 4.916814 3.287618 4.008921

C 3.174860 2.180406 3.391938

H 3.172213 1.525996 4.261783

C 2.200597 2.018147 2.409390

H 1.427328 1.257301 2.538065

C -0.176698 3.165395 0.649778

H -0.956615 3.086834 -0.112324

H -0.517223 2.673398 1.563282

H -0.042191 4.229488 0.875489

C 1.572309 -2.932073 -2.661519

H 2.503555 -2.533709 -3.070568

C 0.436518 -2.074030 -2.731723

H 0.619951 -1.101016 -3.193269

C -1.005169 -2.547277 -2.848823

H -1.568124 -1.784575 -3.398205

H -1.032704 -3.446386 -3.479880

C -1.720231 -2.800617 -1.509513

H -2.475188 -3.595551 -1.627023

H -2.277864 -1.902638 -1.224183

C -0.800367 -3.171778 -0.375627

H -1.123139 -2.890132 0.627561

C 0.189143 -4.131375 -0.494920

H 0.614801 -4.529328 0.424672

C 0.406329 -4.973686 -1.730337

H 0.651731 -5.996072 -1.417465

H -0.537136 -5.054932 -2.284331

C 1.517180 -4.436319 -2.631789

H 1.407235 -4.830808 -3.655785

H 2.486789 -4.804649 -2.271858

C 1.999122 -1.297762 1.519332

H 2.767114 -0.582554 1.242103

H 1.178031 -0.965719 2.152489

C 2.130710 -2.654256 1.217900

H 1.466079 -3.338944 1.746223

C 2.943745 -3.125700 0.143677

H 3.862355 -2.591863 -0.100504

O 2.771007 -1.039838 3.750909

C 1.727602 -0.699938 4.356950

O 0.529073 -0.749978 3.994190

O 1.997545 -0.150497 5.614800

C 0.865144 0.339649 6.300733

H 0.379441 1.156889 5.748801

H 1.229477 0.712717 7.263479

H 0.114810 -0.443852 6.464977

H 2.991793 -4.201593 -0.028183
