# Supplementary material for: Modular access to chiral bridged piperidine-γ-butyrolactones via catalytic asymmetric allylation/aza-Prins cyclization/lactonization sequences
Source: Nat Commun. 2024 Jan 2;15:127. doi: 10.1038/s41467-023-44336-2 (PMC10762176; doi:10.1038/s41467-023-44336-2)
Supplement: Supplementary file 5 — Reporting Summary [file 41467_2023_44336_MOESM5_ESM.pdf]

nature portfolio

Corresponding author(s): CHUN-JIANG WANG

Last updated by author(s): Oct 5, 2023

Reporting Summary

Nature Portfolio wishes to improve the reproducibility of the work that we publish. This form provides structure for consistency and transparency in reporting. For further information on Nature Portfolio policies, see our Editorial Policies and the Editorial Policy Checklist.

Statistics

For all statistical analyses, confirm that the following items are present in the figure legend, table legend, main text, or Methods section.

n/a	Confirmed	
☐	☒	The exact sample size (*n*) for each experimental group/condition, given as a discrete number and unit of measurement
☐	☒	A statement on whether measurements were taken from distinct samples or whether the same sample was measured repeatedly
☒	☐	The statistical test(s) used AND whether they are one- or two-sided<br>*Only common tests should be described solely by name; describe more complex techniques in the Methods section.*
☒	☐	A description of all covariates tested
☒	☐	A description of any assumptions or corrections, such as tests of normality and adjustment for multiple comparisons
☐	☒	A full description of the statistical parameters including central tendency (e.g. means) or other basic estimates (e.g. regression coefficient) AND variation (e.g. standard deviation) or associated estimates of uncertainty (e.g. confidence intervals)
☒	☐	For null hypothesis testing, the test statistic (e.g. *F*, *t*, *r*) with confidence intervals, effect sizes, degrees of freedom and *P* value noted<br>*Give P values as exact values whenever suitable.*
☒	☐	For Bayesian analysis, information on the choice of priors and Markov chain Monte Carlo settings
☒	☐	For hierarchical and complex designs, identification of the appropriate level for tests and full reporting of outcomes
☒	☐	Estimates of effect sizes (e.g. Cohen's *d*, Pearson's *r*), indicating how they were calculated

Our web collection on statistics for biologists contains articles on many of the points above.

Software and code

Policy information about availability of computer code

Data collection	No software was used
Data analysis	We used Reed and Muench's method for IC50 calculation and statistical analysis.

For manuscripts utilizing custom algorithms or software that are central to the research but not yet described in published literature, software must be made available to editors and reviewers. We strongly encourage code deposition in a community repository (e.g. GitHub). See the Nature Portfolio guidelines for submitting code & software for further information.

Data

Policy information about availability of data

All manuscripts must include a data availability statement. This statement should provide the following information, where applicable:

- Accession codes, unique identifiers, or web links for publicly available datasets
- A description of any restrictions on data availability
- For clinical datasets or third party data, please ensure that the statement adheres to our policy

The authors declare that all the other data supporting the findings of this study are available within the paper and its supplementary information files, and from the corresponding author upon reasonable request. A reporting summary for this Article is available as a Supplementary Information file.

Research involving human participants, their data, or biological material

Policy information about studies with human participants or human data. See also policy information about sex, gender (identity/presentation), and sexual orientation and race, ethnicity and racism.

Reporting on sex and gender	The work did not include any studies with human research participants
Reporting on race, ethnicity, or other socially relevant groupings	The work did not include any studies with human research participants
Population characteristics	The work did not include any studies with human research participants
Recruitment	The work did not include any studies with human research participants
Ethics oversight	The work did not include any studies with human research participants

Note that full information on the approval of the study protocol must also be provided in the manuscript.

Field-specific reporting

Please select the one below that is the best fit for your research. If you are not sure, read the appropriate sections before making your selection.

[x] Life sciences [ ] Behavioural & social sciences [ ] Ecological, evolutionary & environmental sciences

For a reference copy of the document with all sections, see nature.com/documents/nr-reporting-summary-flat.pdf

Life sciences study design

All studies must disclose on these points even when the disclosure is negative.

Sample size	For the in vitro cytotoxicity test, prepare a single cell suspension using a culture medium containing 10% fetal bovine serum (DMEM or RMPI1640), and inoculate 3000-15000 cells per well onto a 96-wells plate with a volume of 100 µL cells per well (HL-60 cancer cells have 15000 cells, A549 lung carcinoma cells have 3000 cells, SMMC-7721 hepatoma cells, MDA-MB-231 breast adenocarcinoma cells, and SW480 cells colon cancer cells have 5000 cells). Cells are inoculated and cultured 12-24 hours in advance.
Data exclusions	No data were excluded
Replication	In vitro cytotoxicity in triplicate (with untreated as the negative control and doxorubicin treatment as the positive control). All attempts at replication are successful.
Randomization	No randomization was used for in vitro experiments. In this study, randomization is not relevant as we did not use any animal models.
Blinding	No blinding was used for in vitro experiments. Blinding is not not applicable, since our experiments are based on cell culture.

Reporting for specific materials, systems and methods

We require information from authors about some types of materials, experimental systems and methods used in many studies. Here, indicate whether each material, system or method listed is relevant to your study. If you are not sure if a list item applies to your research, read the appropriate section before selecting a response.

Materials & experimental systems

n/a	Involved in the study	
[x]	[ ]	Antibodies
[ ]	[x]	Eukaryotic cell lines
[x]	[ ]	Palaeontology and archaeology
[x]	[ ]	Animals and other organisms
[x]	[ ]	Clinical data
[x]	[ ]	Dual use research of concern
[x]	[ ]	Plants

Methods

n/a	Involved in the study	
[x]	[ ]	ChIP-seq
[x]	[ ]	Flow cytometry
[x]	[ ]	MRI-based neuroimaging

Eukaryotic cell lines

Policy information about cell lines and Sex and Gender in Research

Cell line source(s)	The human tumor cell lines HL-60, A549, SMMC-7721, MDA-MB-231, and SW480 were used in the cytotoxic assay. These cell lines were obtained from ATCC (Manassas, VA, USA) and BeNa Culture Collection. Cells were cultured in RMPI-1640 or DMEM medium (Biological Industries, Kibbutz Beit-Haemek, Israel) supplemented with 10% fetal bovine serum (FBS, Biological Industries) at 37 °C in a humidified atmosphere with 5% CO2.
Authentication	Cell lines were stored in liquid nitrogen, and were only used within passage 20. None of the cell lines used were authenticated.
Mycoplasma contamination	All the cell lines used were negative for mycoplasma contamination.
Commonly misidentified lines (See ICLAC register)	No cell lines used in this manuscript were misidentified
